# Supplementary figures and images for: Negative regulation of APC/C activation by MAPK-mediated attenuation of Cdc20Slp1 under stress (part 1 of 2)
Source: eLife. 2024 Oct 16;13:RP97896. doi: 10.7554/eLife.97896 (PMC11483130; doi:10.7554/eLife.97896)

Figure 1-figure supplement 1A.

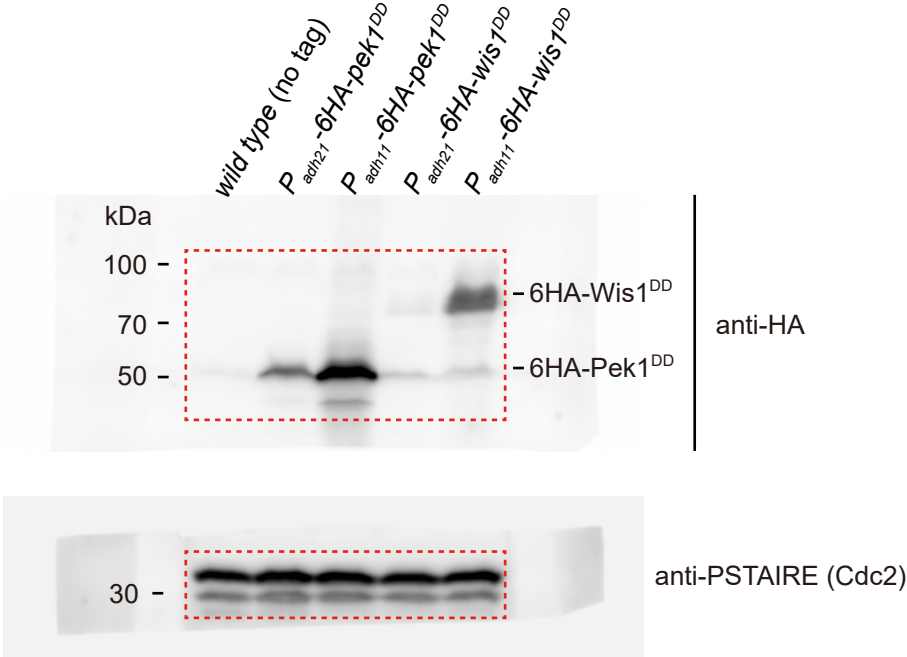

Supplement: Figure 1—figure supplement 1—source data 1. [file elife-97896-fig1-figsupp1-data1.pdf]

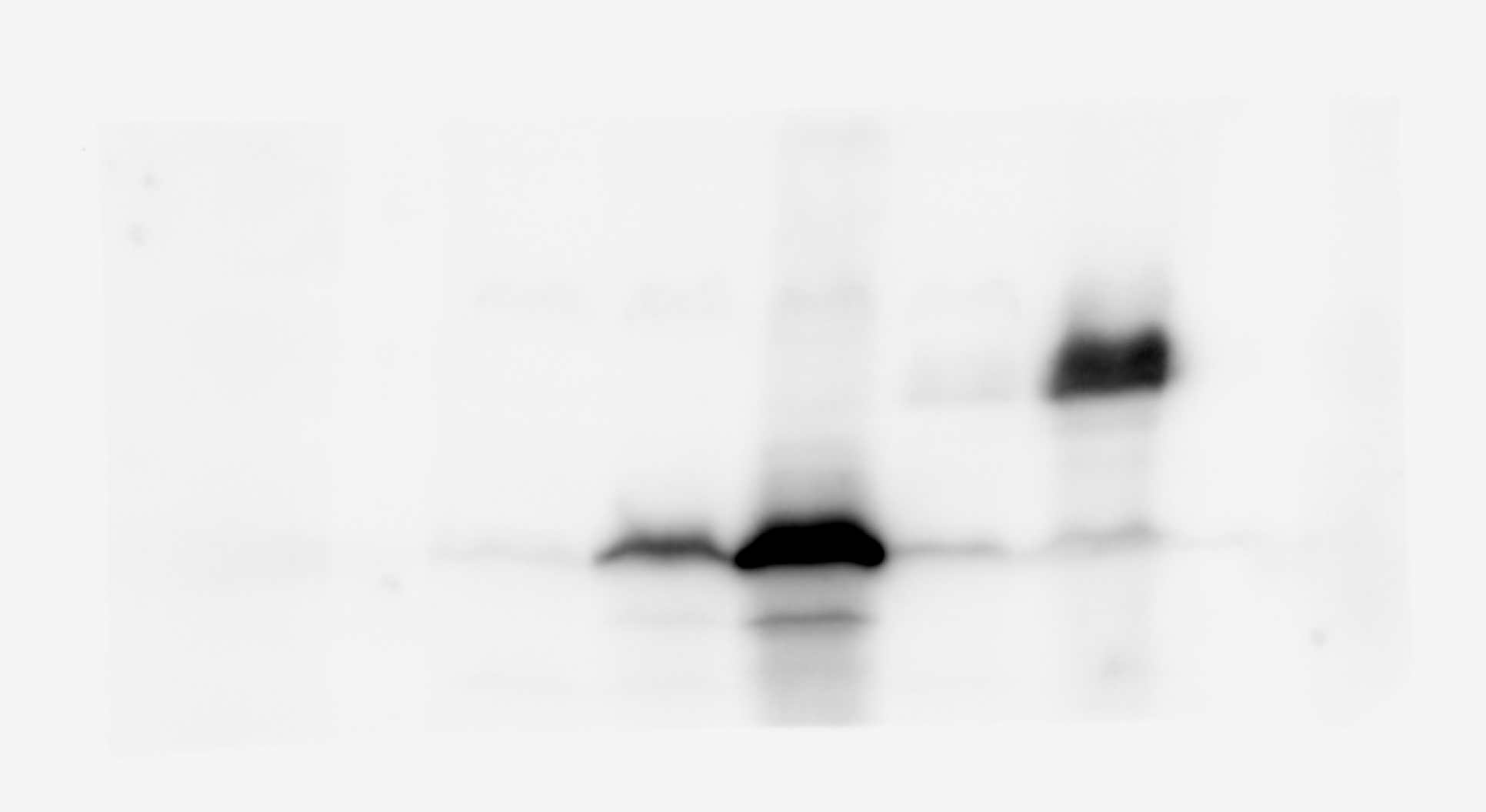

Supplement: Figure 1—figure supplement 1—source data 3. [file elife-97896-fig1-figsupp1-data3.zip › Figure 1-figure supplement 1-Source Data 3. Full raw unedited blot (anti-HA) for Figure 1-figure supplement 1A.tif]

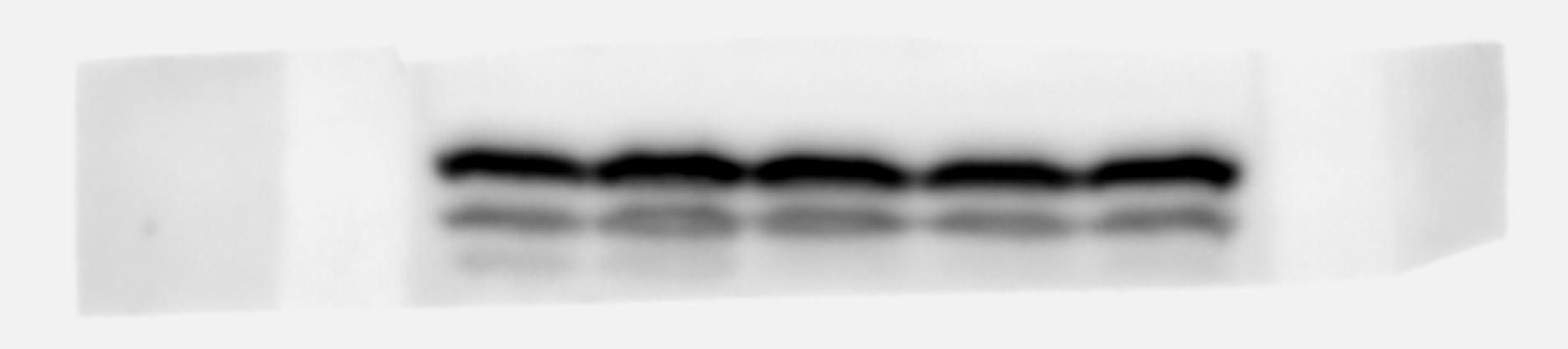

Supplement: Figure 1—figure supplement 1—source data 4. [file elife-97896-fig1-figsupp1-data4.zip › Figure 1-figure supplement 1-Source Data 4. Full raw unedited blot (Cdc2) for Figure 1-figure supplement 1A.tif]

Figure 1-figure supplement 2A

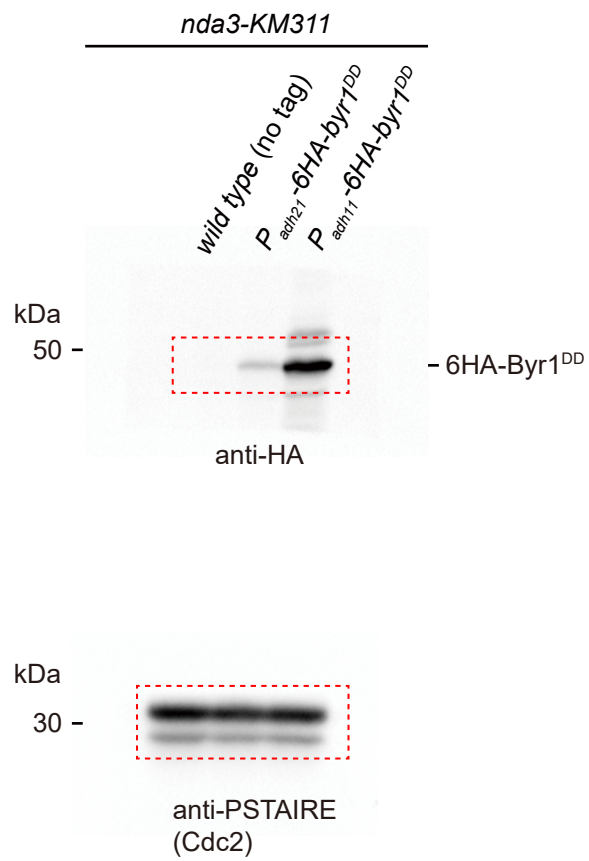

Supplement: Figure 1—figure supplement 2—source data 1. [file elife-97896-fig1-figsupp2-data1.pdf]

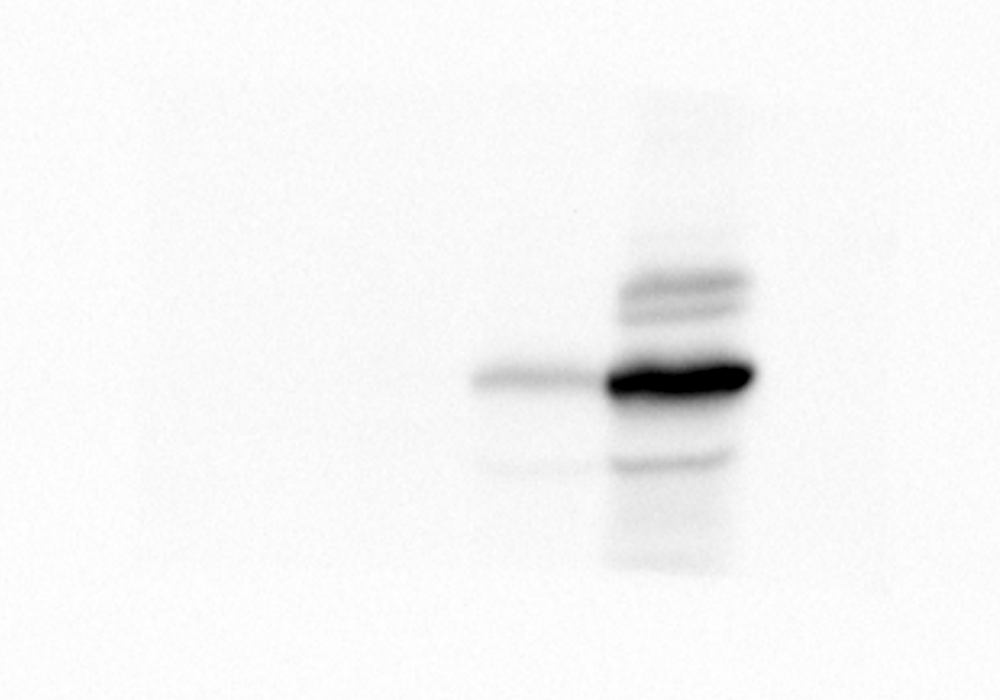

Supplement: Figure 1—figure supplement 2—source data 3. [file elife-97896-fig1-figsupp2-data3.zip › Figure 1-figure supplement 2-Source Data 3. Full raw unedited blot (anti-HA) for Figure 1-figure supplement 2A.tif]

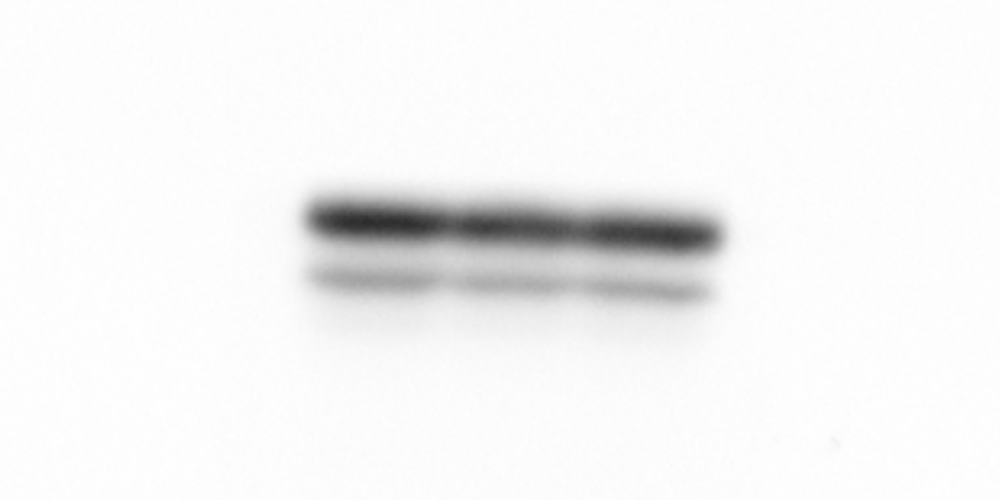

Supplement: Figure 1—figure supplement 2—source data 4. [file elife-97896-fig1-figsupp2-data4.zip › Figure 1-figure supplement 2-Source Data 4. Full raw unedited blot (Cdc2) for Figure 1-figure supplement 2A.tif]

Figure 2A.

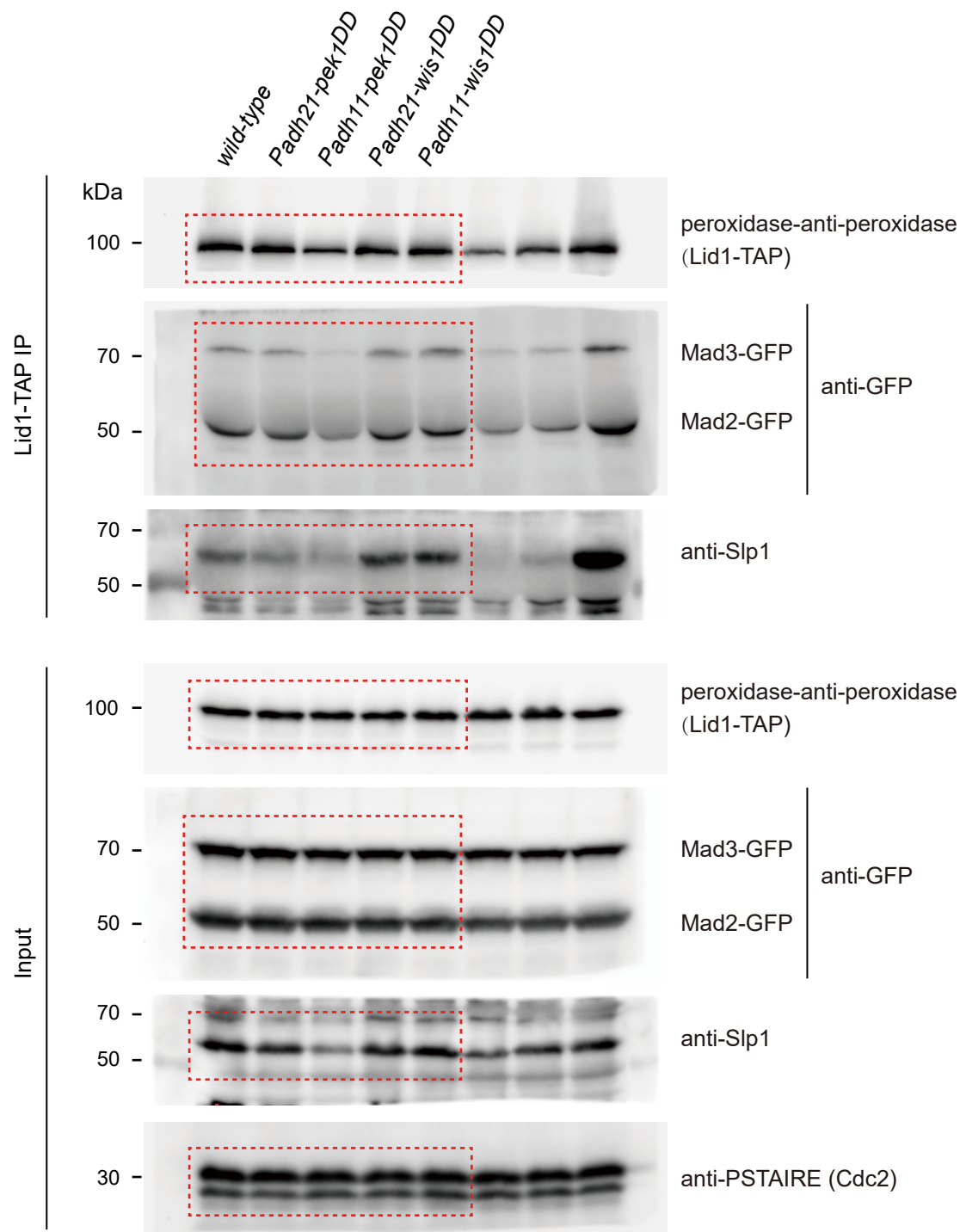

Figure 2B.

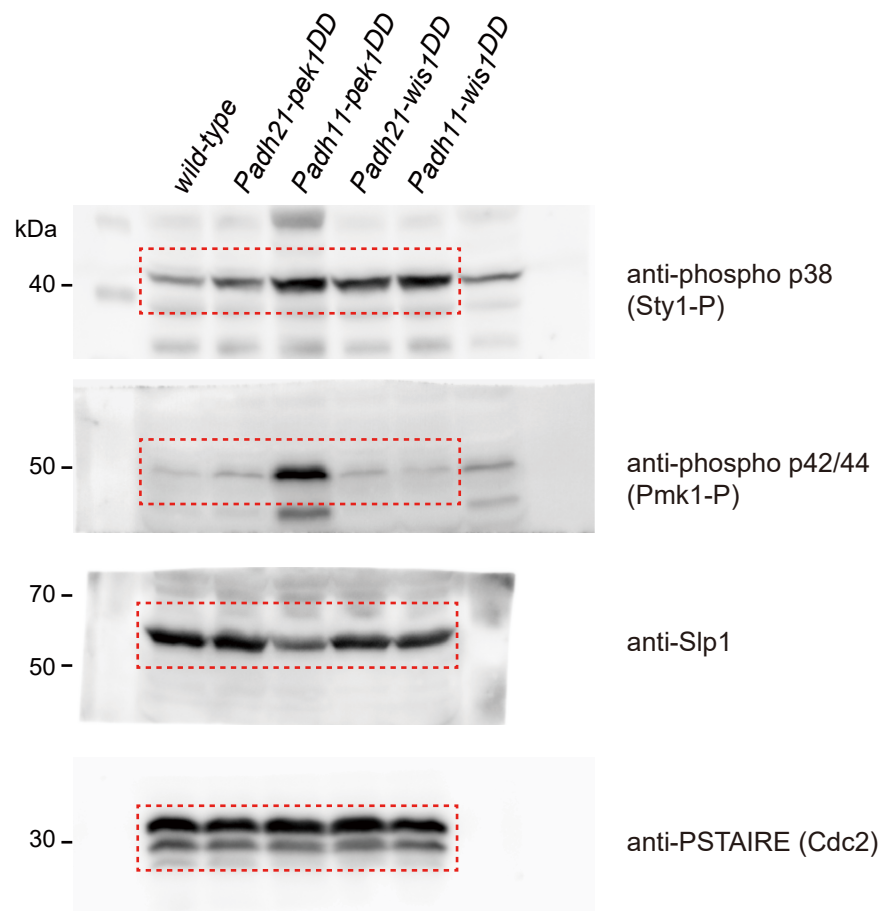

Figure 2C.

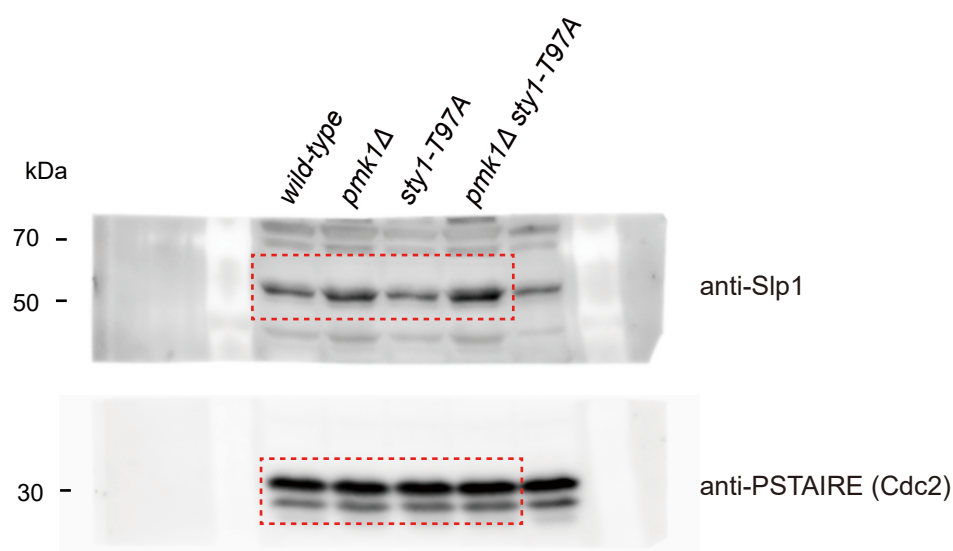

Supplement: Figure 2—source data 1. [file elife-97896-fig2-data1.pdf]

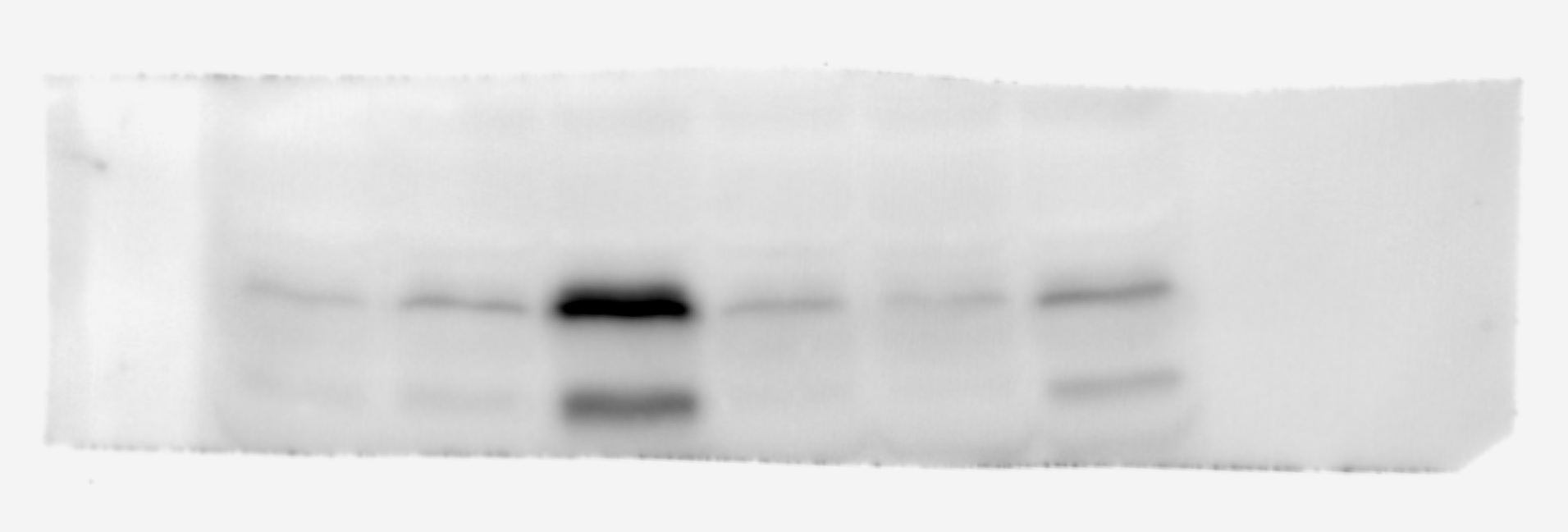

Supplement: Figure 2—source data 3. [file elife-97896-fig2-data3.zip › Figure 2-Source Data 3-15. /Figure 2-Source Data 11. Full raw unedited blot (phosphorylated Pmk1) for Figure 2B.tif]

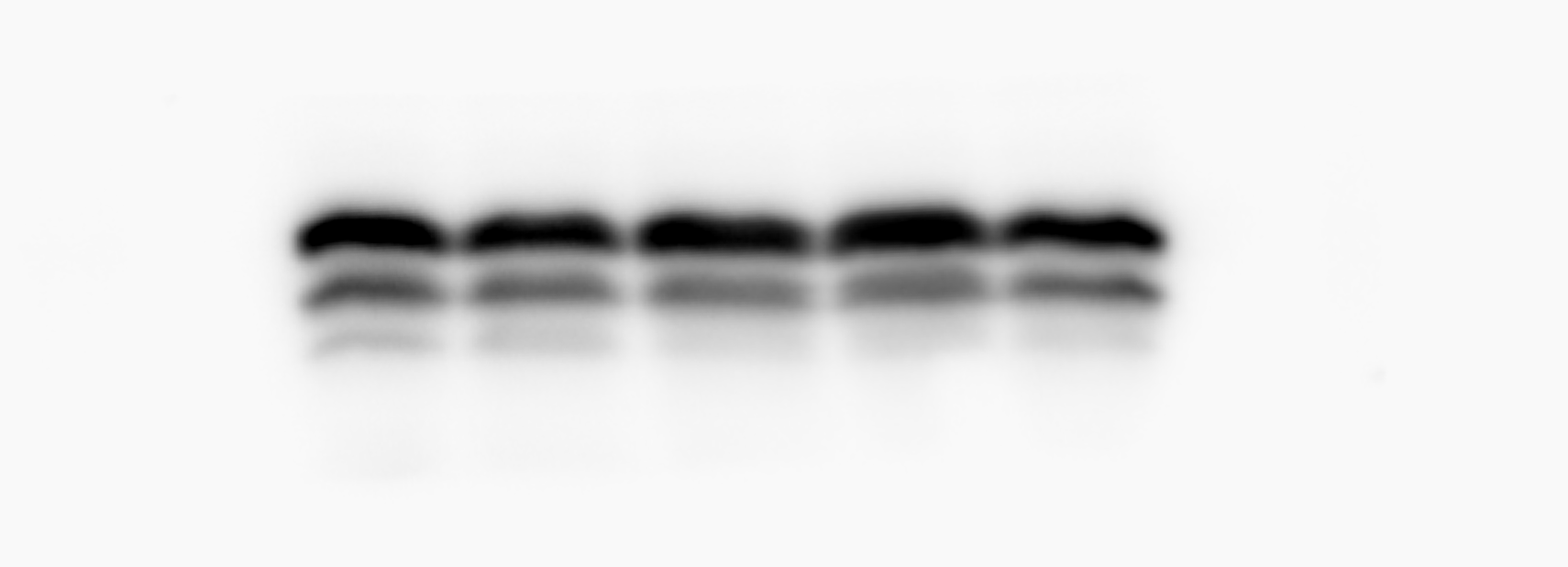

Supplement: Figure 2—source data 3. [file elife-97896-fig2-data3.zip › Figure 2-Source Data 3-15. /Figure 2-Source Data 13. Full raw unedited blot (Cdc2) for Figure 2B.tif]

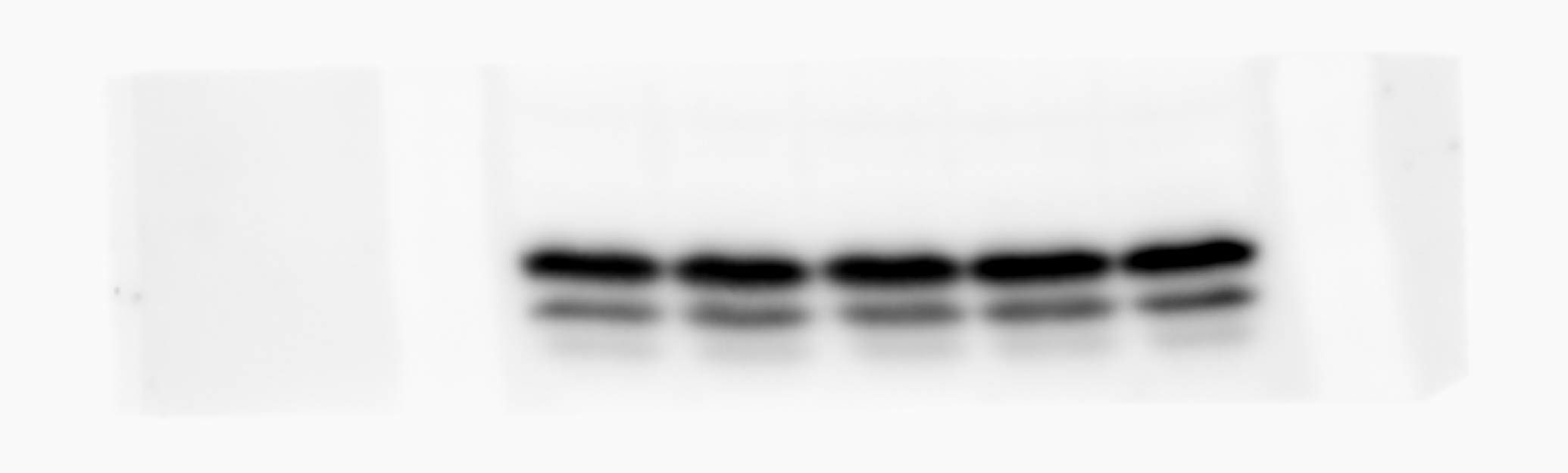

Supplement: Figure 2—source data 3. [file elife-97896-fig2-data3.zip › Figure 2-Source Data 3-15. /Figure 2-Source Data 15. Full raw unedited blot (Cdc2) for Figure 2C.tif]

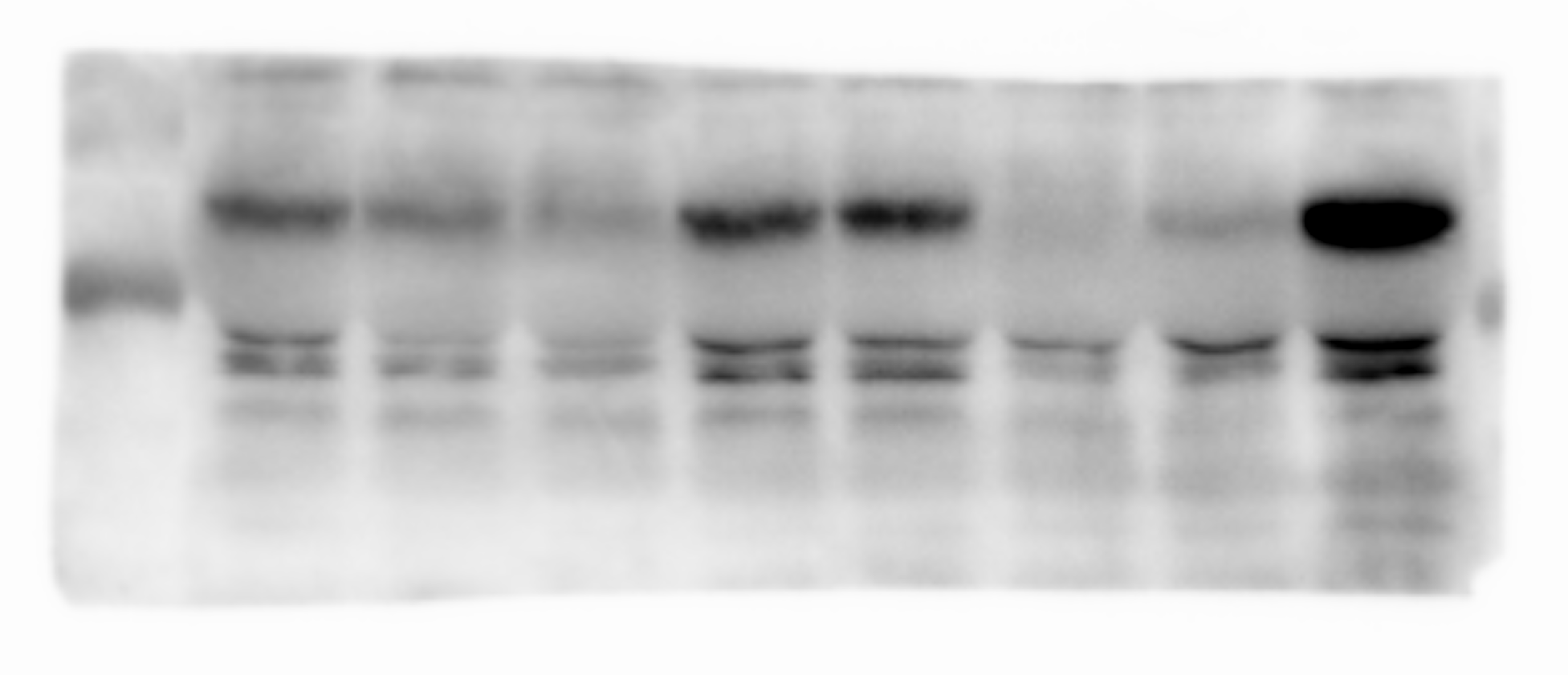

Supplement: Figure 2—source data 3. [file elife-97896-fig2-data3.zip › Figure 2-Source Data 3-15. /Figure 2-Source Data 5. Full raw unedited blot (co-IPed Slp1) for Figure 2A.tif]

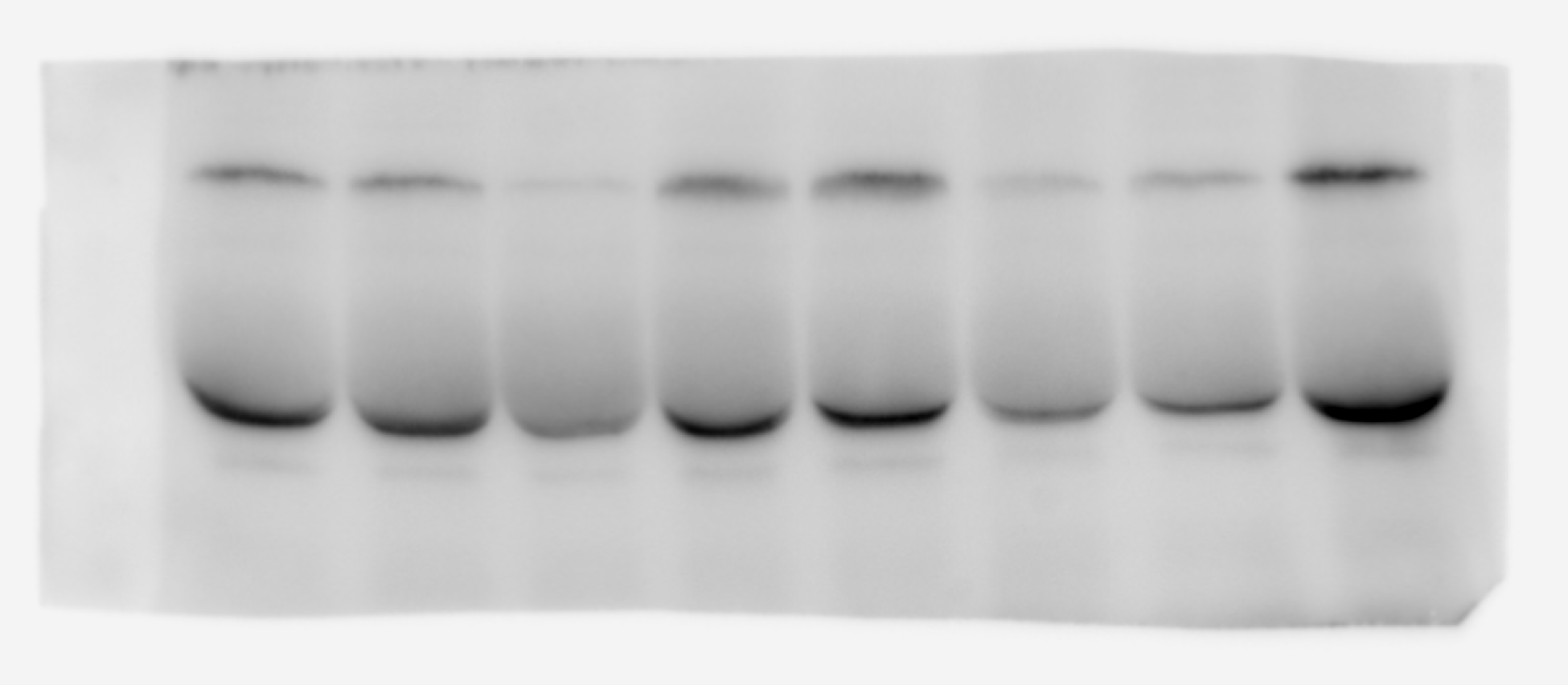

Supplement: Figure 2—source data 3. [file elife-97896-fig2-data3.zip › Figure 2-Source Data 3-15. /Figure 2-Source Data 4. Full raw unedited blot (co-IPed Mad2-GFPMad3-GFP) for Figure 2A.tif]

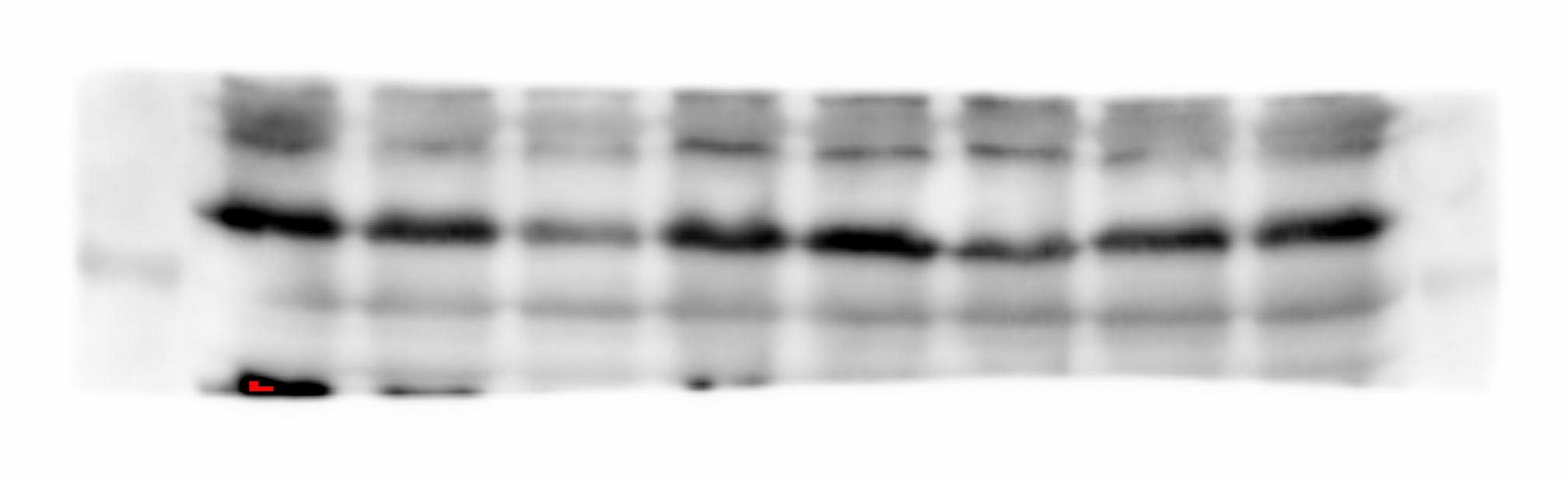

Supplement: Figure 2—source data 3. [file elife-97896-fig2-data3.zip › Figure 2-Source Data 3-15. /Figure 2-Source Data 8. Full raw unedited blot (Slp1 input) for Figure 2A.tif]

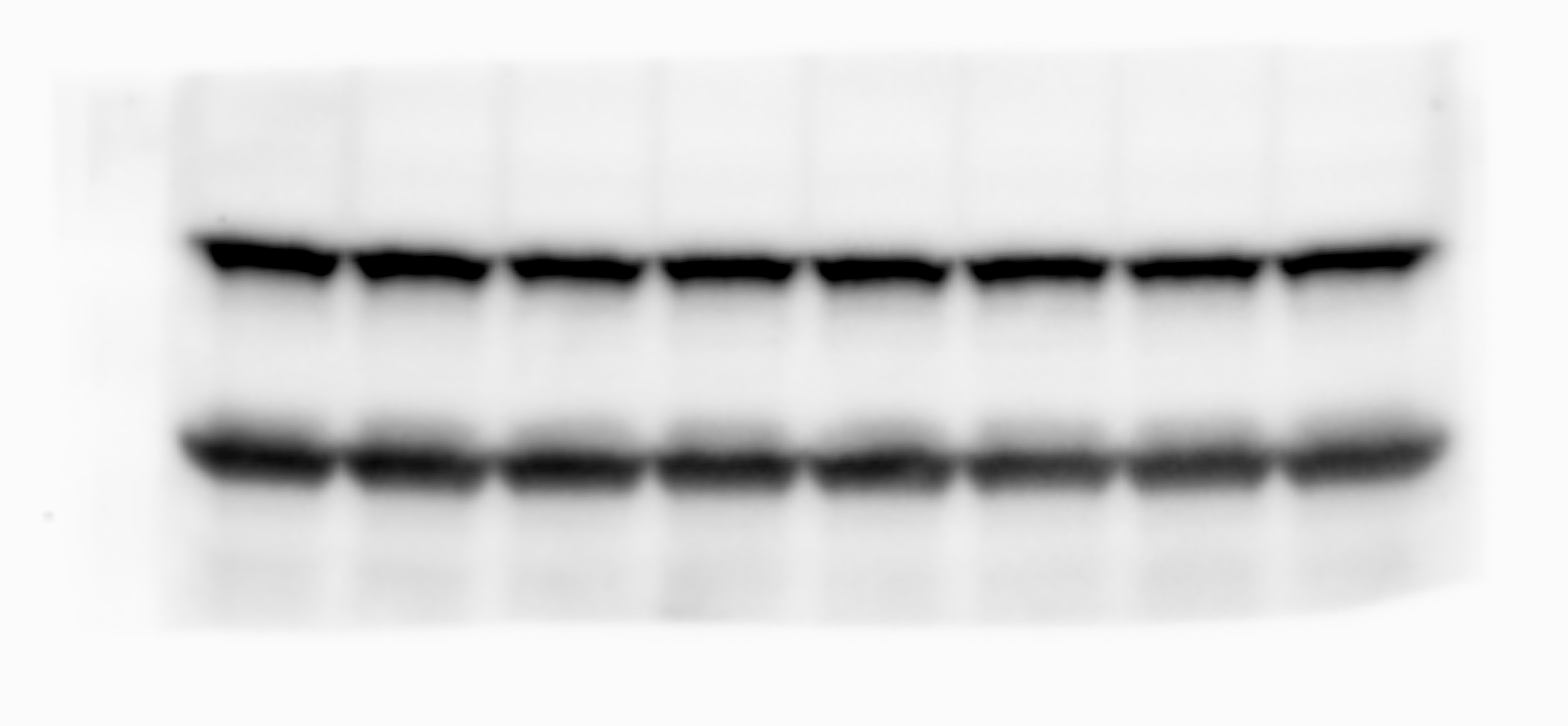

Supplement: Figure 2—source data 3. [file elife-97896-fig2-data3.zip › Figure 2-Source Data 3-15. /Figure 2-Source Data 7. Full raw unedited blot (Mad2-GFP & Mad3-GFP input) for Figure 2A.tif]

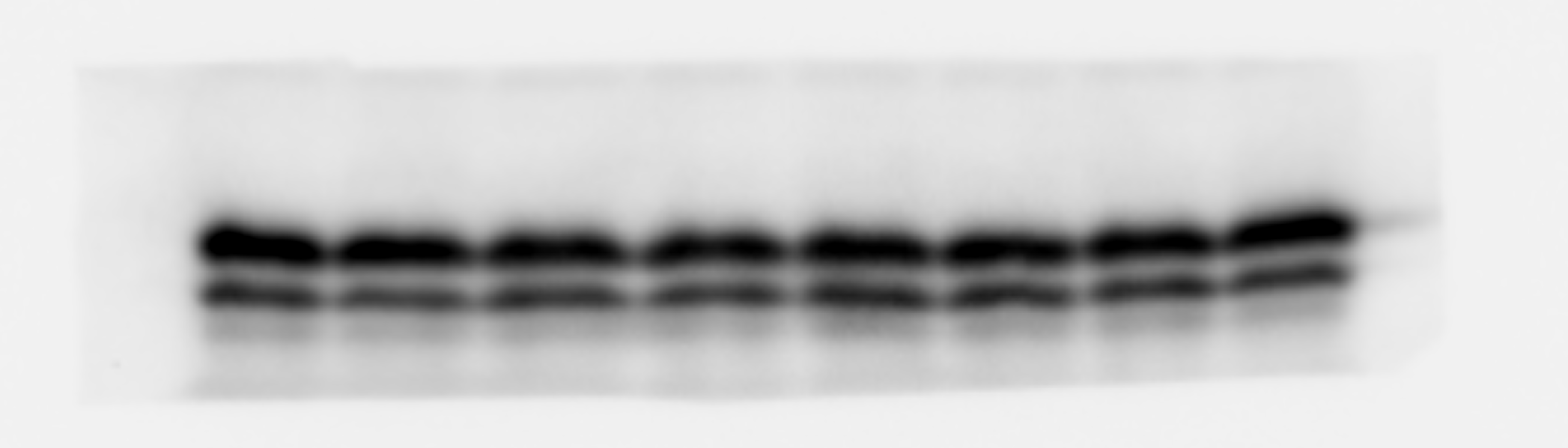

Supplement: Figure 2—source data 3. [file elife-97896-fig2-data3.zip › Figure 2-Source Data 3-15. /Figure 2-Source Data 9. Full raw unedited blot (Cdc2 input) for Figure 2A.tif]

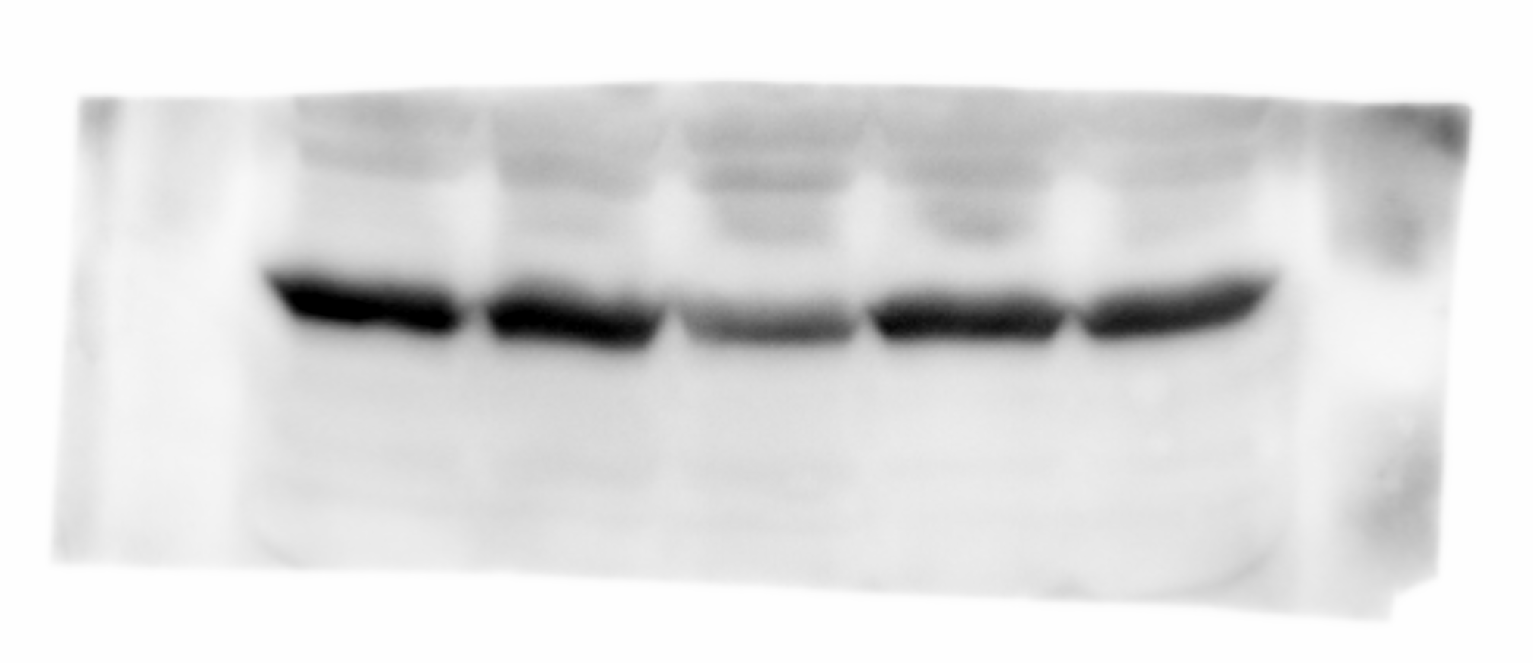

Supplement: Figure 2—source data 3. [file elife-97896-fig2-data3.zip › Figure 2-Source Data 3-15. /Figure 2-Source Data 12. Full raw unedited blot (Slp1) for Figure 2B.tif]

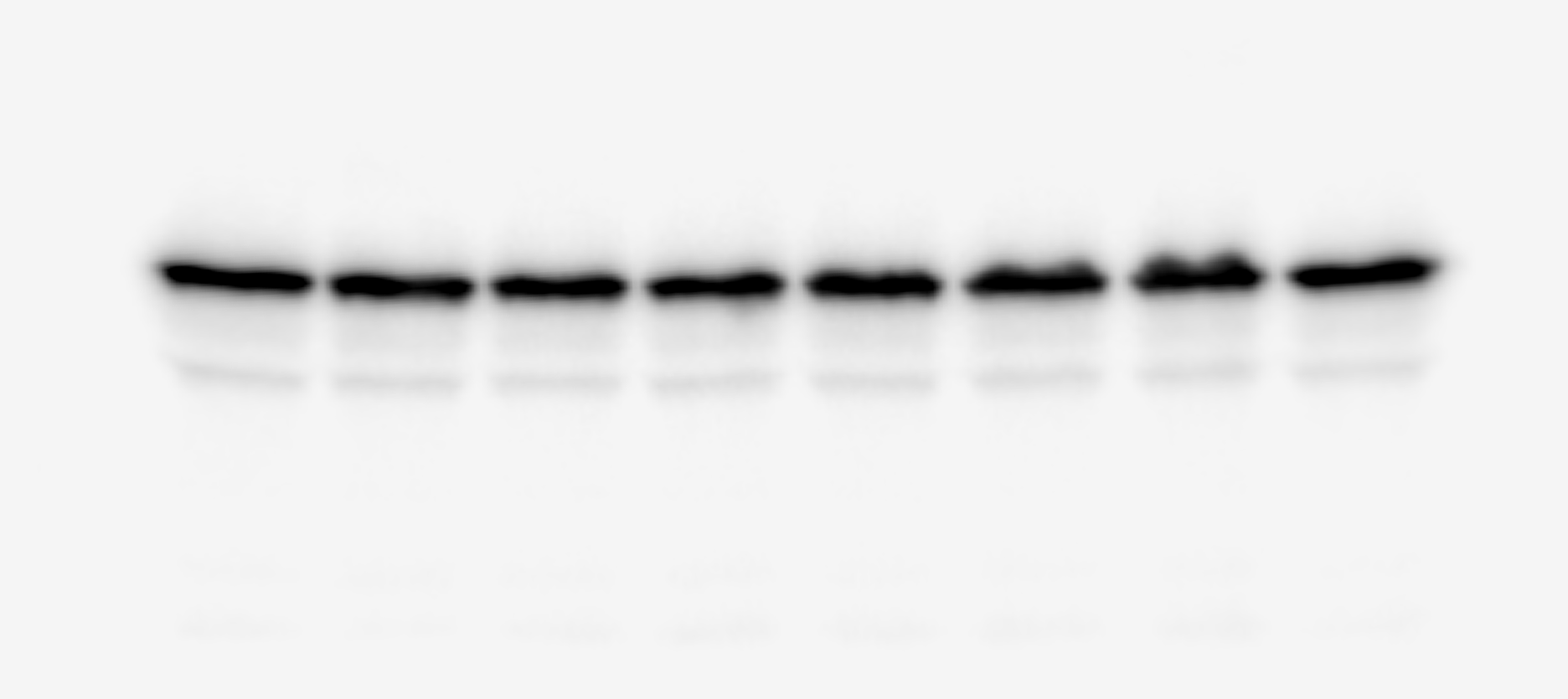

Supplement: Figure 2—source data 3. [file elife-97896-fig2-data3.zip › Figure 2-Source Data 3-15. /Figure 2-Source Data 6. Full raw unedited blot (Lid1-TAP input) for Figure 2A.tif]

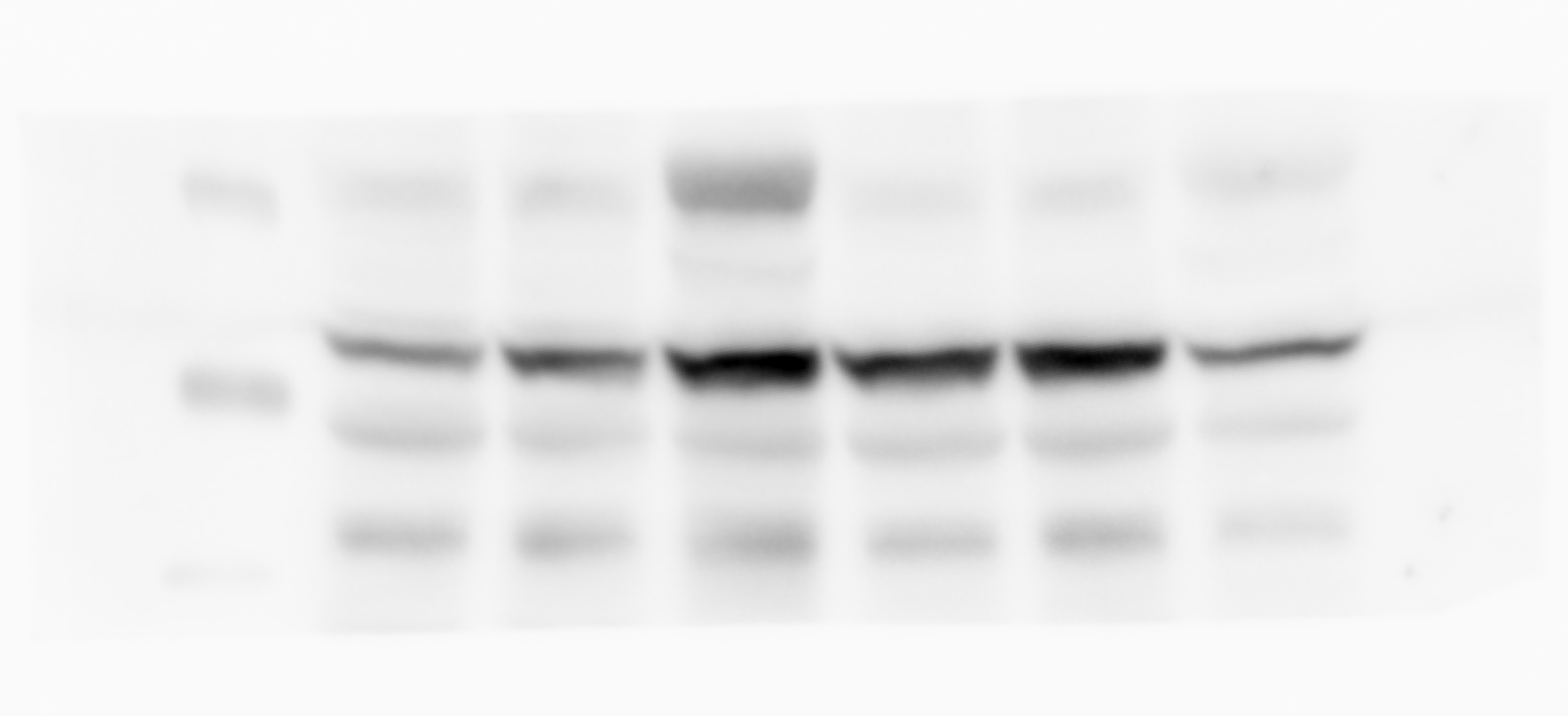

Supplement: Figure 2—source data 3. [file elife-97896-fig2-data3.zip › Figure 2-Source Data 3-15. /Figure 2-Source Data 10. Full raw unedited blot (phosphorylated Sty1) for Figure 2B.tif]

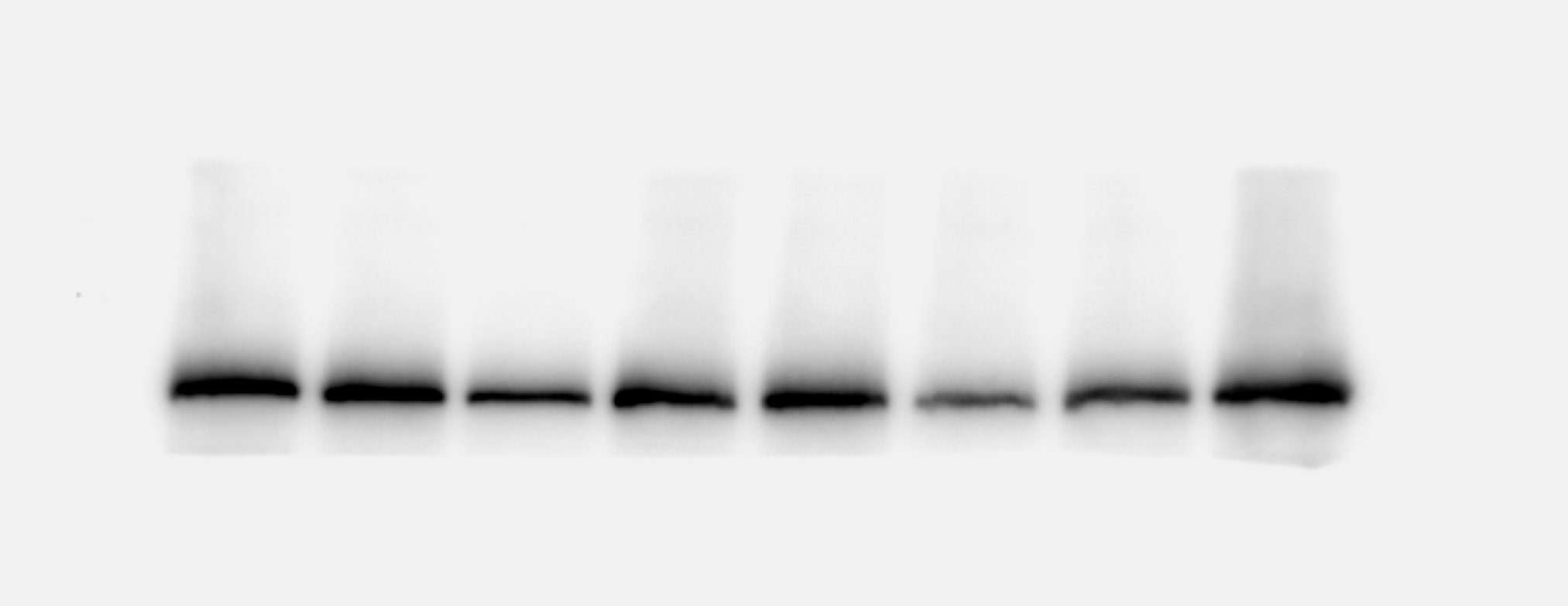

Supplement: Figure 2—source data 3. [file elife-97896-fig2-data3.zip › Figure 2-Source Data 3-15. /Figure 2-Source Data 3. Full raw unedited blot (IPed Lid1-TAP) for Figure 2A.tif]

Figure 2-figure supplement 1C

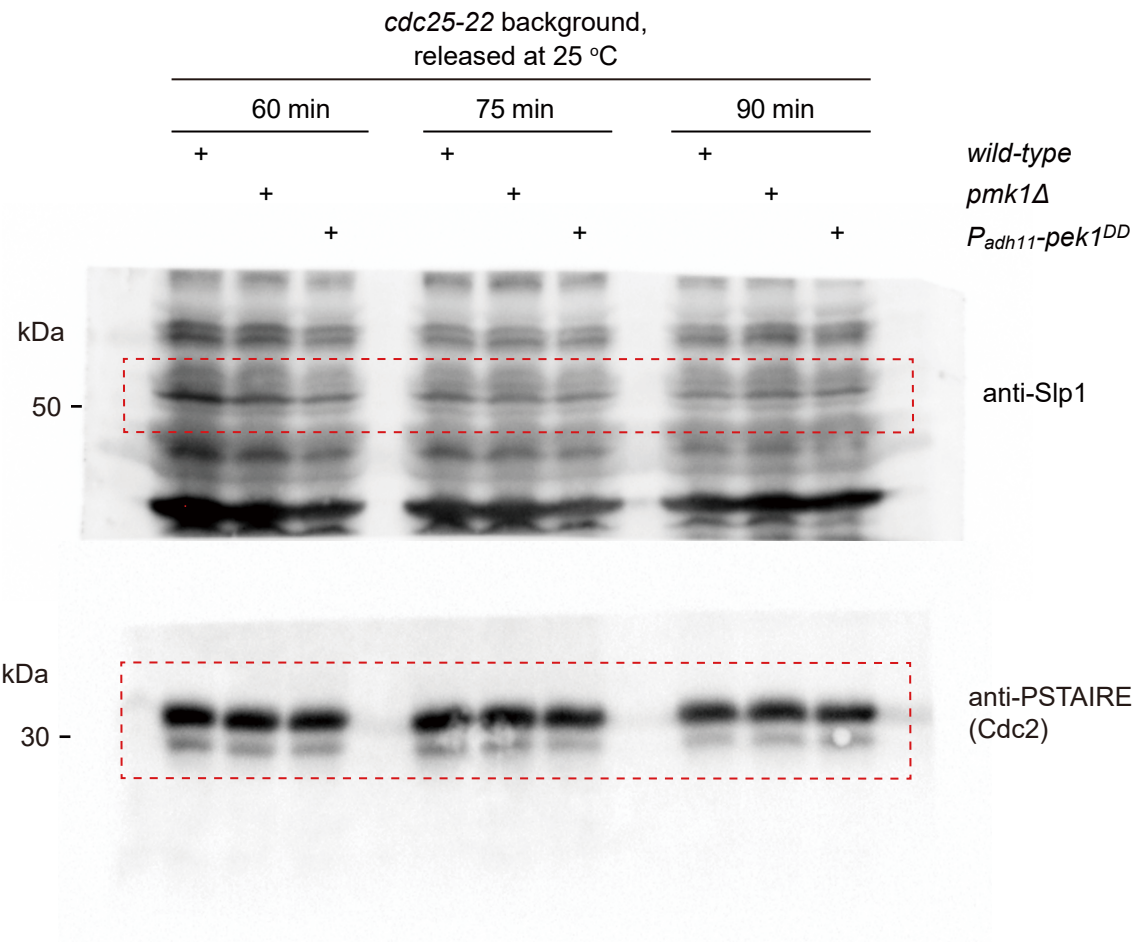

Supplement: Figure 2—figure supplement 1—source data 1. [file elife-97896-fig2-figsupp1-data1.pdf]

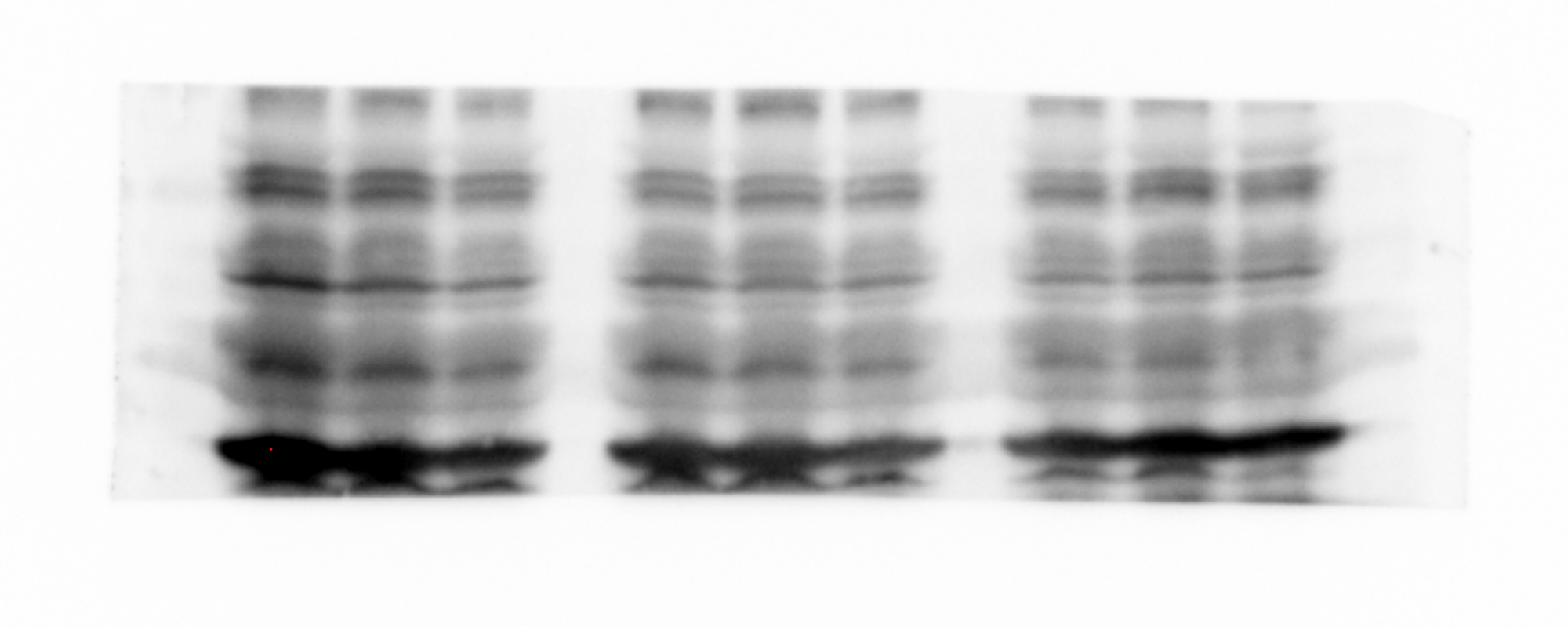

Supplement: Figure 2—figure supplement 1—source data 3. [file elife-97896-fig2-figsupp1-data3.zip › Figure 2-figure supplement 1-Source Data 3. Full raw unedited blot (Slp1) for Figure 2-figure supplement 1C.tif]

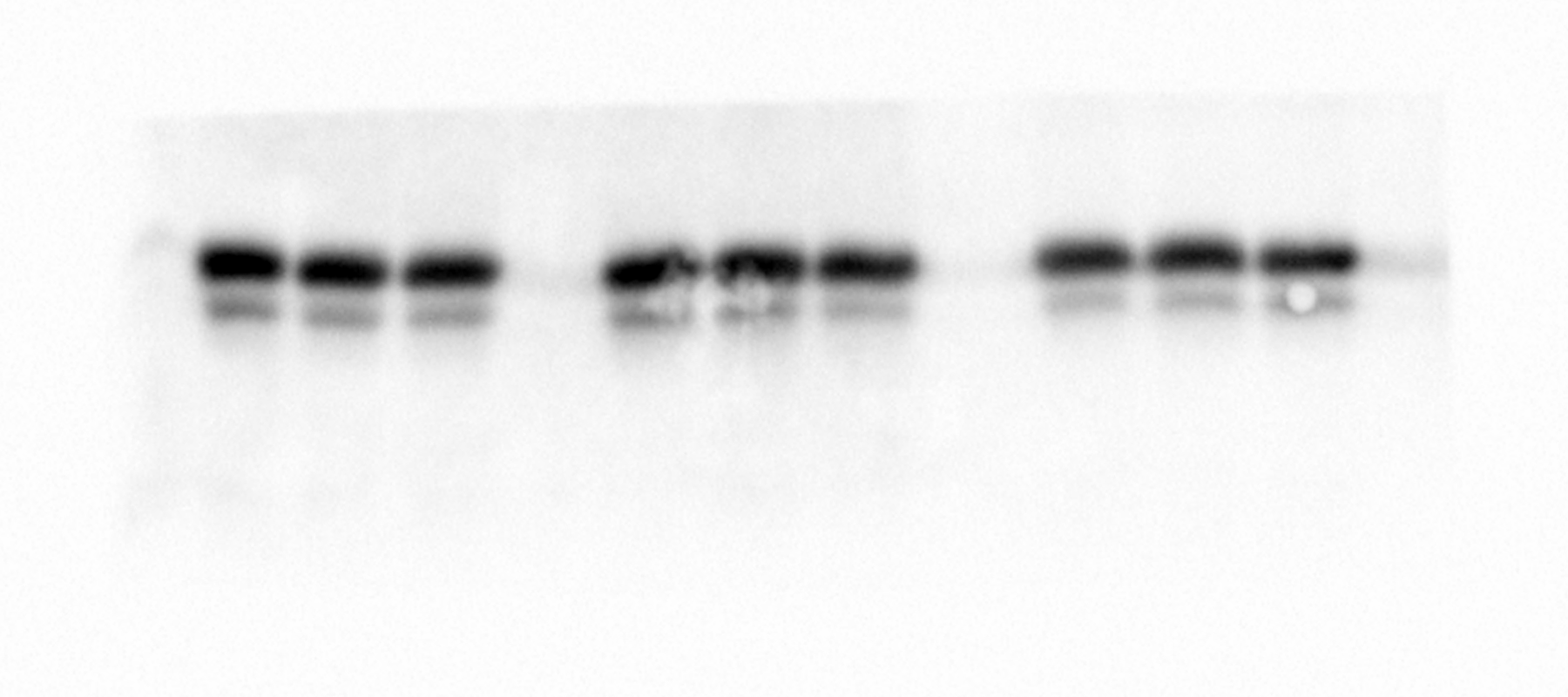

Supplement: Figure 2—figure supplement 1—source data 4. [file elife-97896-fig2-figsupp1-data4.zip › Figure 2-figure supplement 1-Source Data 4. Full raw unedited blot (Cdc2) for Figure 2-figure supplement 1C.tif]

Figure 3A.

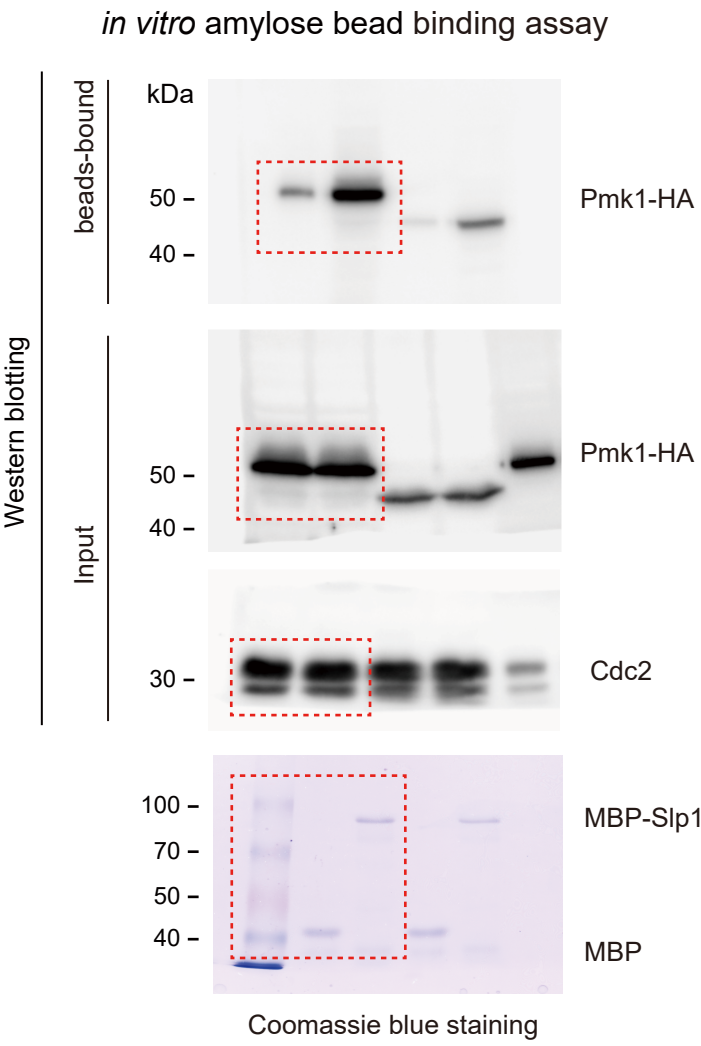

Figure 3C.

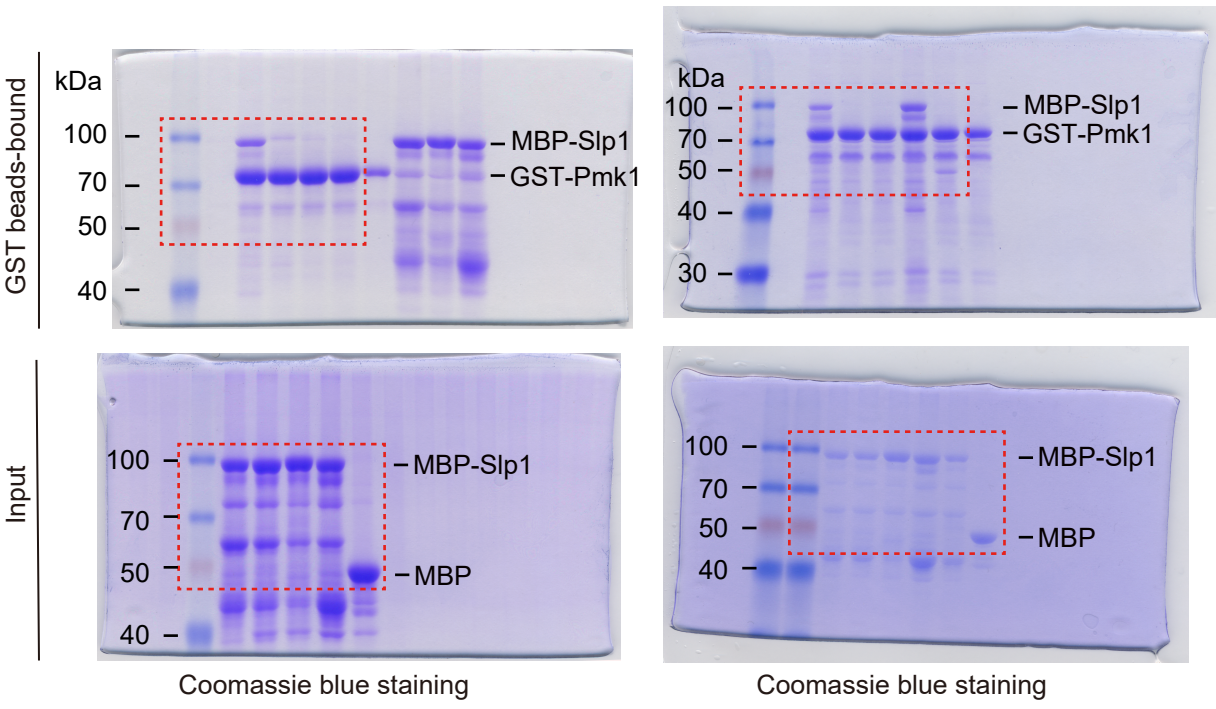

Figure 3D.

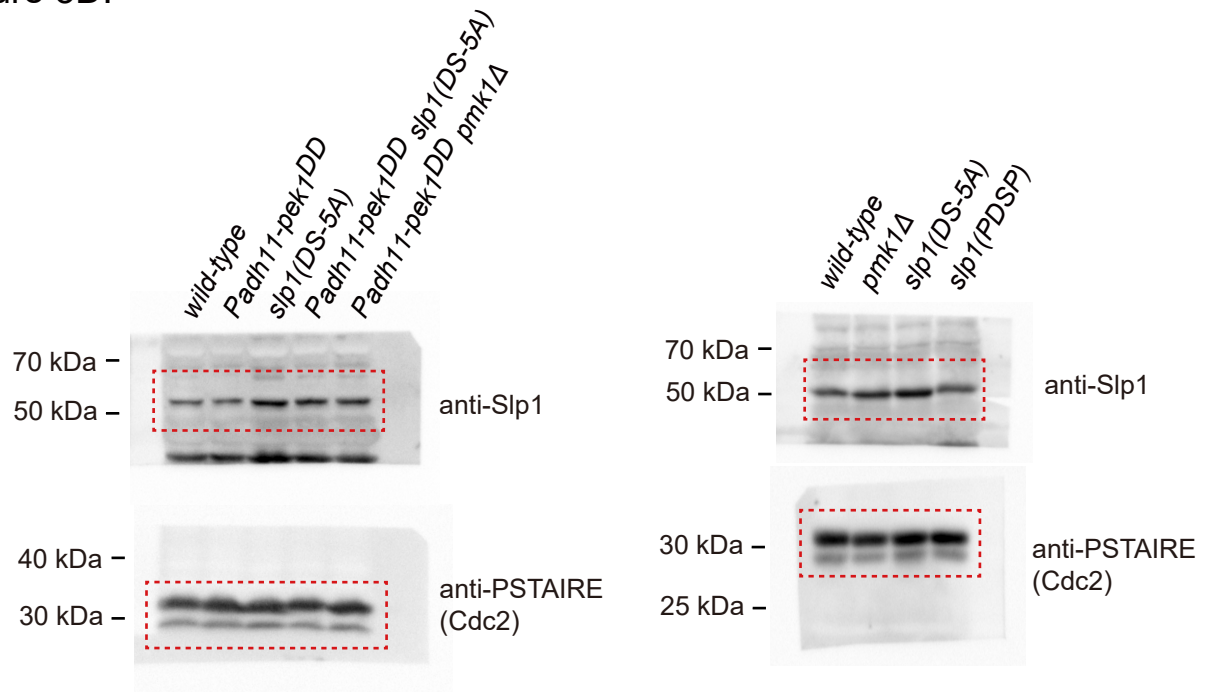

Supplement: Figure 3—source data 1. [file elife-97896-fig3-data1.pdf]

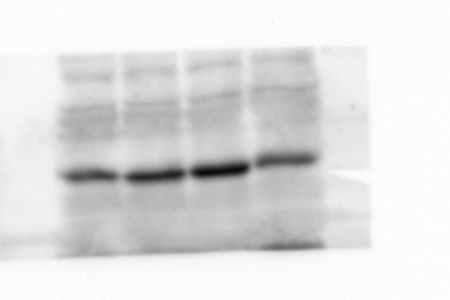

Supplement: Figure 3—source data 3. [file elife-97896-fig3-data3.zip › Figure 3-Source Data 3-14. /Figure 3-Source Data 12. Full raw unedited blot (Slp1, right) for Figure 3D.tif]

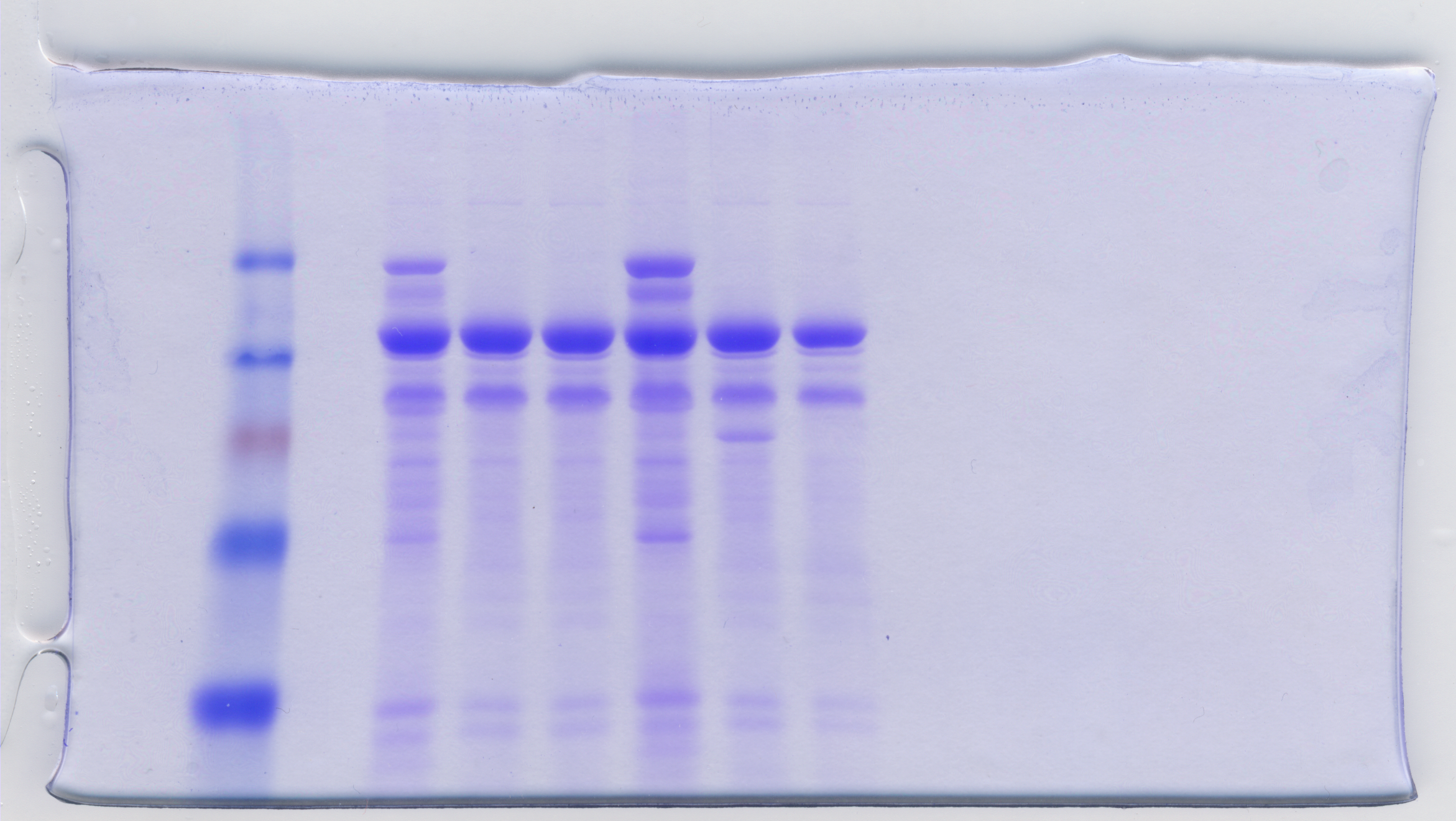

Supplement: Figure 3—source data 3. [file elife-97896-fig3-data3.zip › Figure 3-Source Data 3-14. /Figure 3-Source Data 8. Full raw unedited Coomassie gel (bead-bound, right) for Figure 3C.tif]

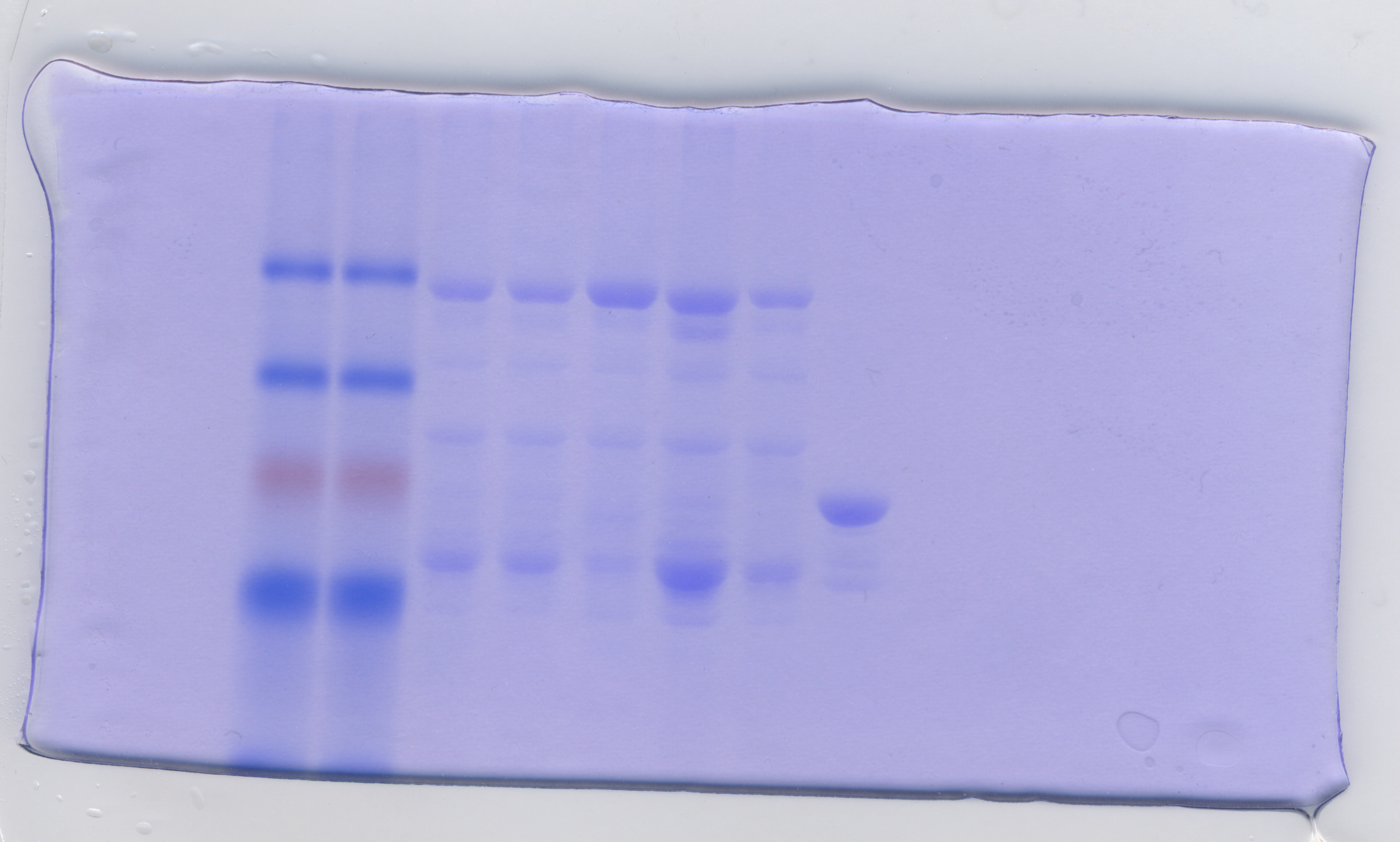

Supplement: Figure 3—source data 3. [file elife-97896-fig3-data3.zip › Figure 3-Source Data 3-14. /Figure 3-Source Data 10. Full raw unedited Coomassie gel (input, right) for Figure 3C.tif]

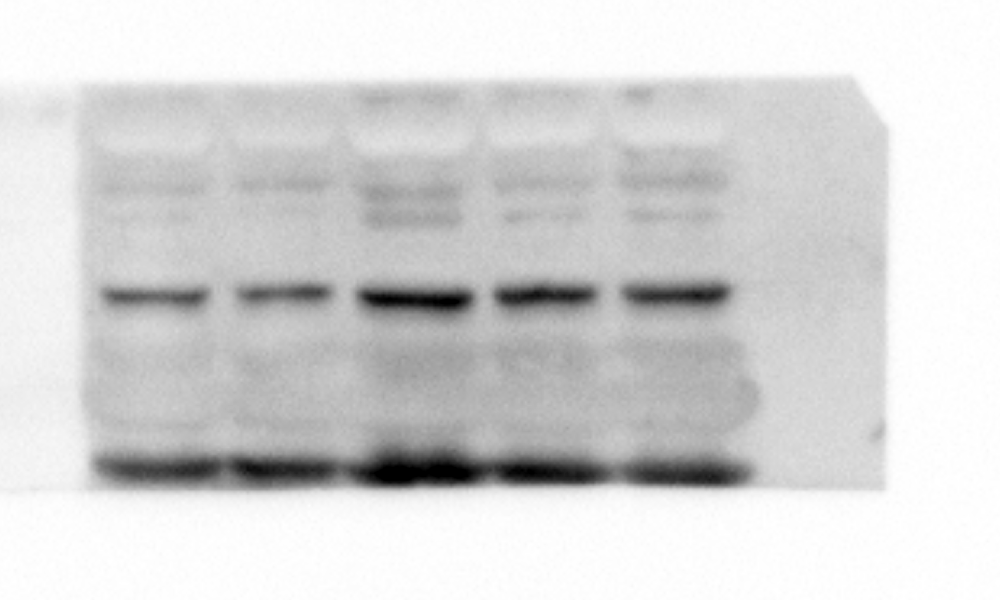

Supplement: Figure 3—source data 3. [file elife-97896-fig3-data3.zip › Figure 3-Source Data 3-14. /Figure 3-Source Data 11. Full raw unedited blot (Slp1, left) for Figure 3D.tif]

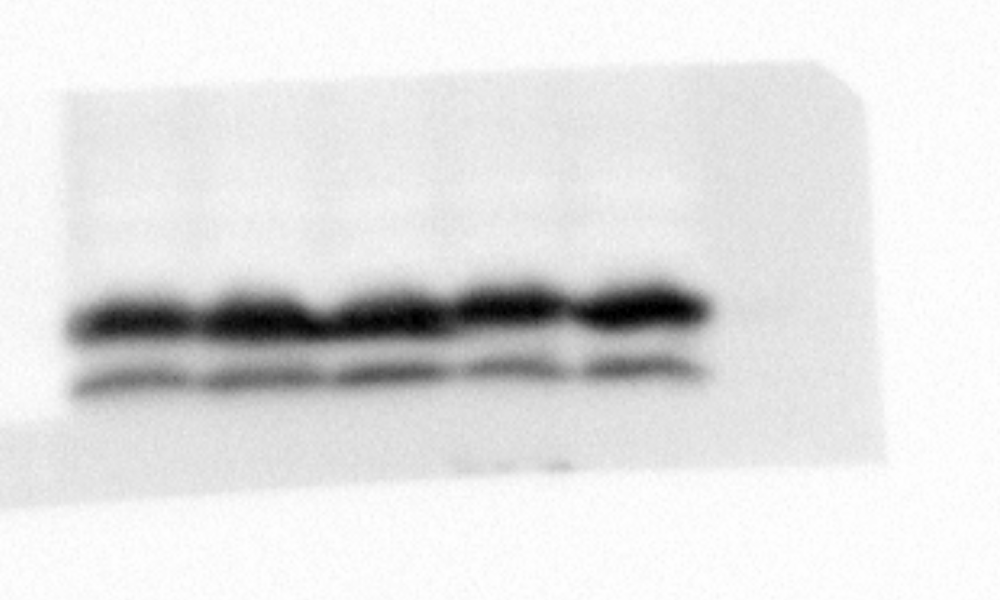

Supplement: Figure 3—source data 3. [file elife-97896-fig3-data3.zip › Figure 3-Source Data 3-14. /Figure 3-Source Data 13. Full raw unedited blot (Cdc2, left) for Figure 3D.tif]

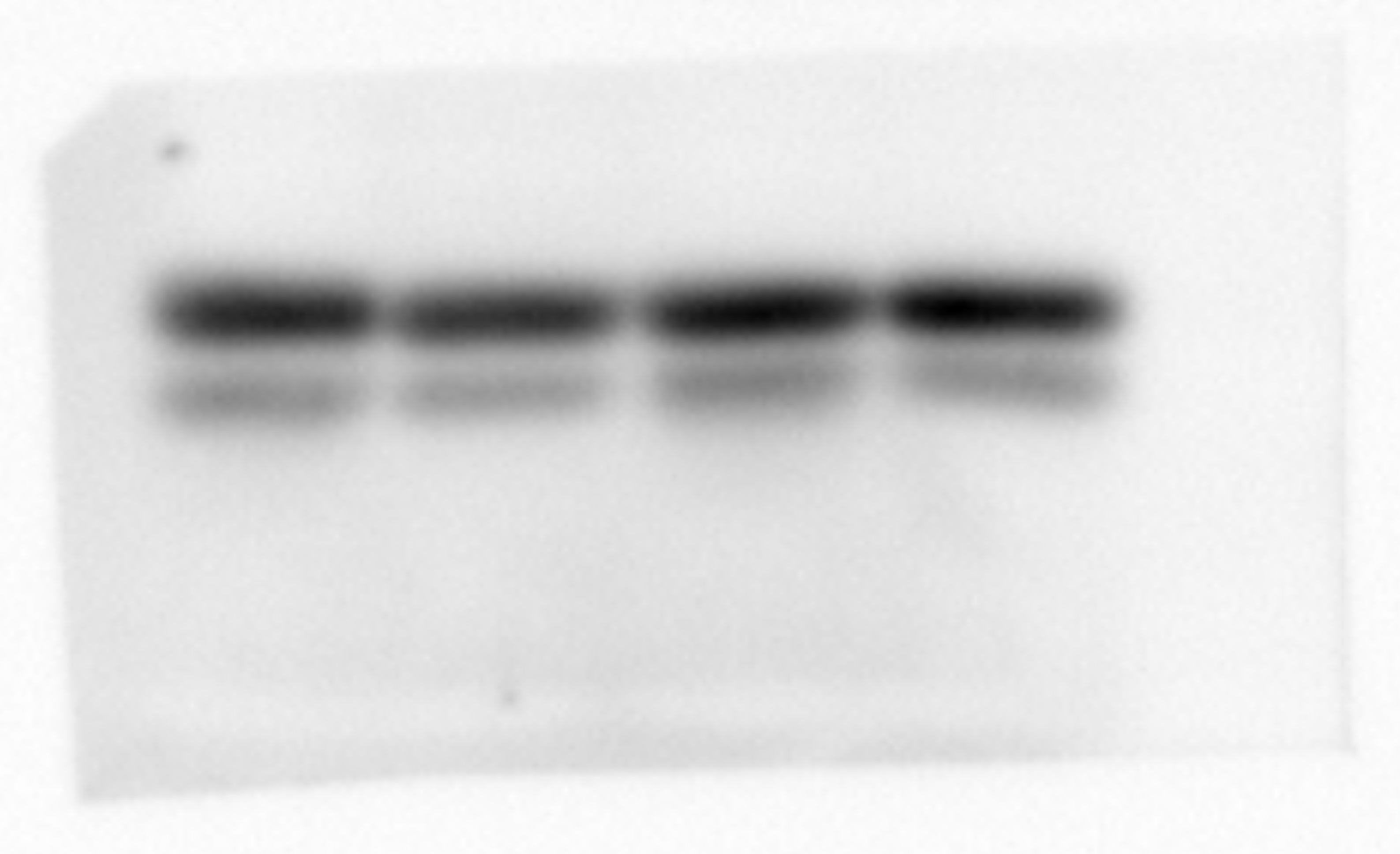

Supplement: Figure 3—source data 3. [file elife-97896-fig3-data3.zip › Figure 3-Source Data 3-14. /Figure 3-Source Data 14. Full raw unedited blot (Cdc2, right) for Figure 3D.tif]

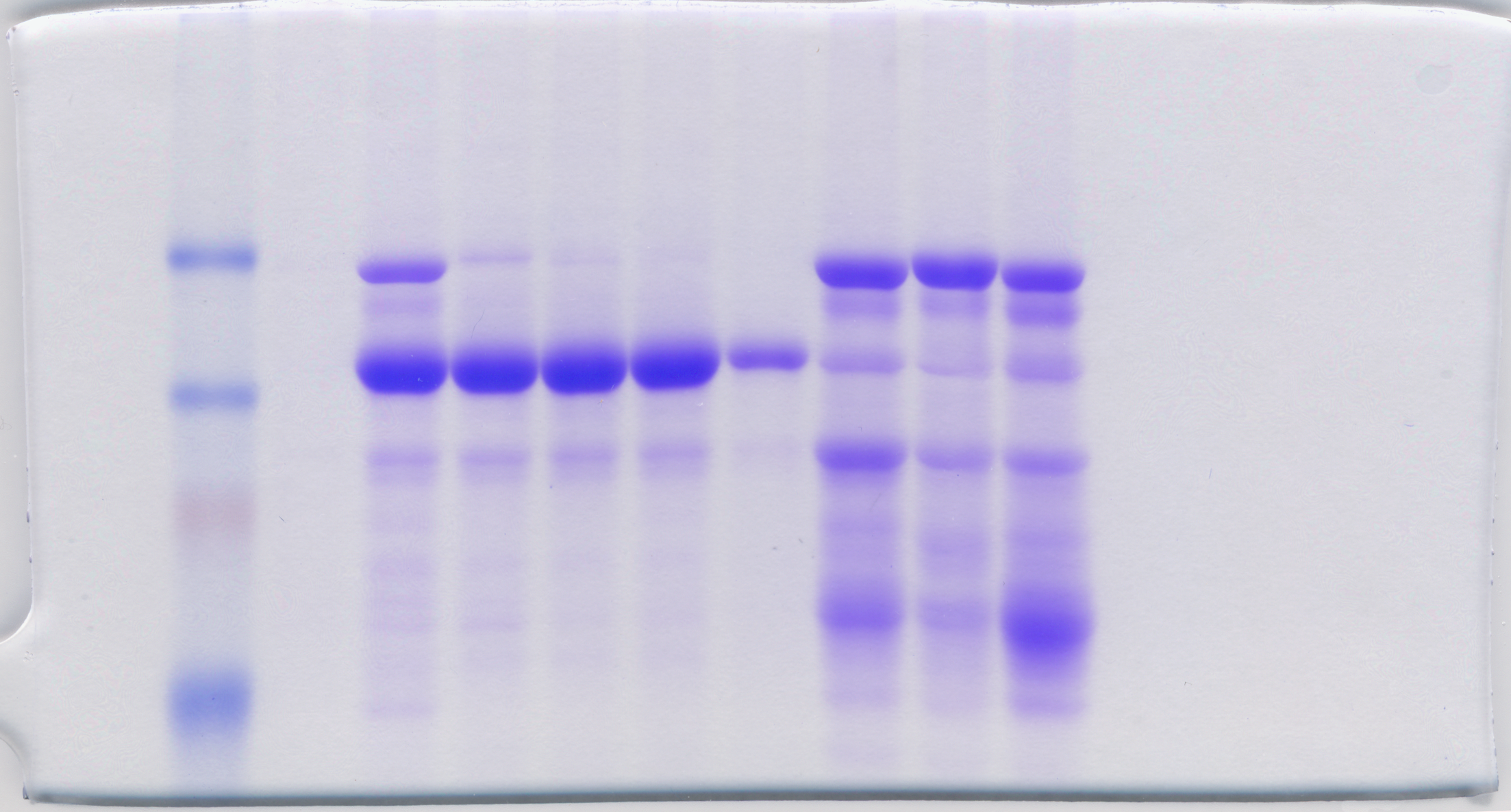

Supplement: Figure 3—source data 3. [file elife-97896-fig3-data3.zip › Figure 3-Source Data 3-14. /Figure 3-Source Data 7. Full raw unedited Coomassie gel (bead-bound, left) for Figure 3C.tif]

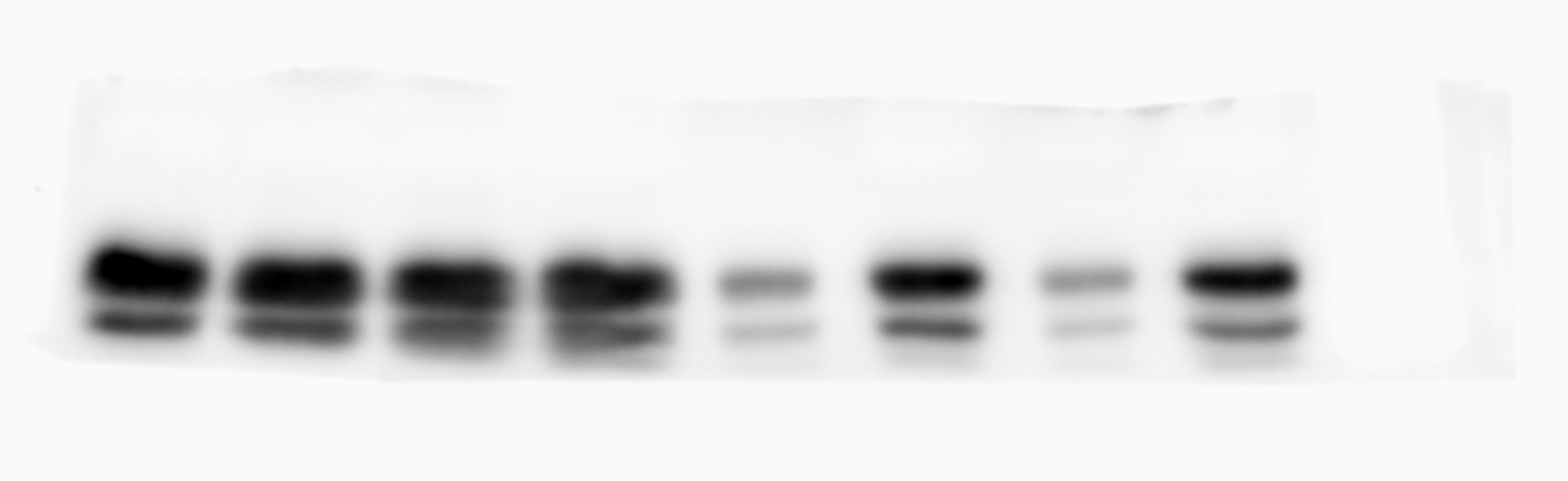

Supplement: Figure 3—source data 3. [file elife-97896-fig3-data3.zip › Figure 3-Source Data 3-14. /Figure 3-Source Data 5. Full raw unedited blot (Cdc2 input) for Figure 3A.tif]

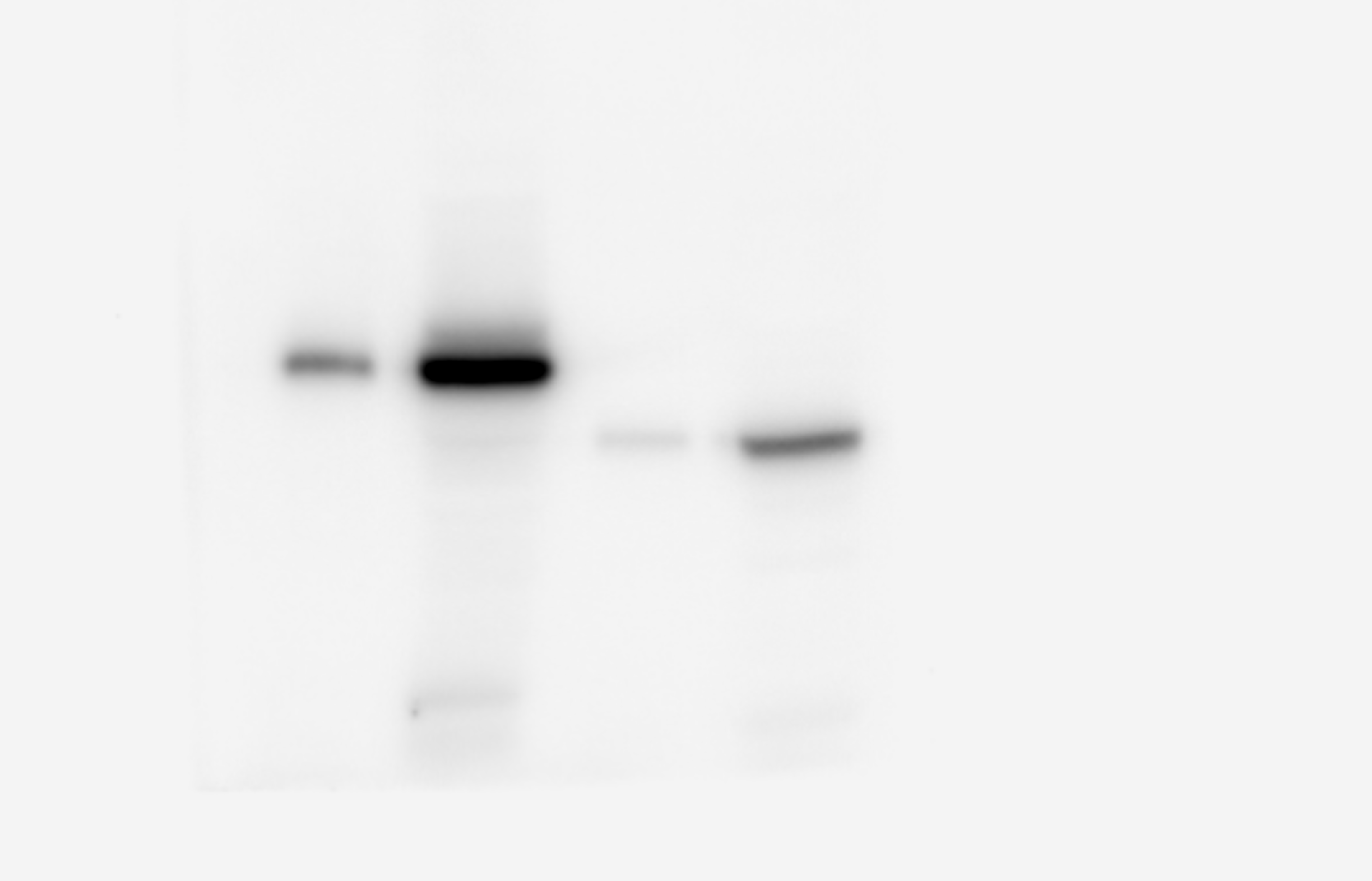

Supplement: Figure 3—source data 3. [file elife-97896-fig3-data3.zip › Figure 3-Source Data 3-14. /Figure 3-Source Data 3. Full raw unedited blot (bead-bound Pmk1-HA) for Figure 3A.tif]

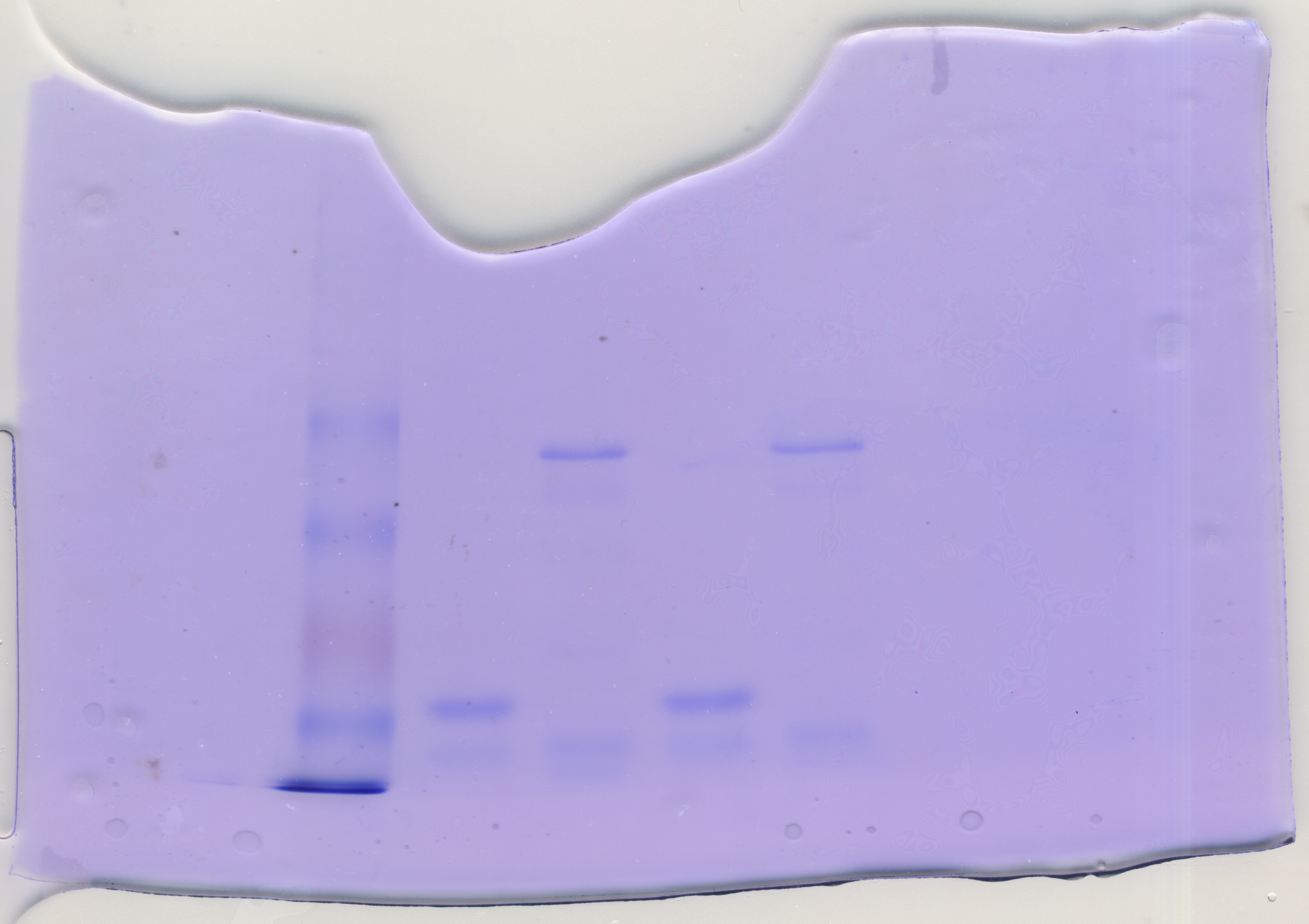

Supplement: Figure 3—source data 3. [file elife-97896-fig3-data3.zip › Figure 3-Source Data 3-14. /Figure 3-Source Data 6. Full raw unedited Coomassie gel (MBP & MBP-Slp1) for Figure 3A.tif]

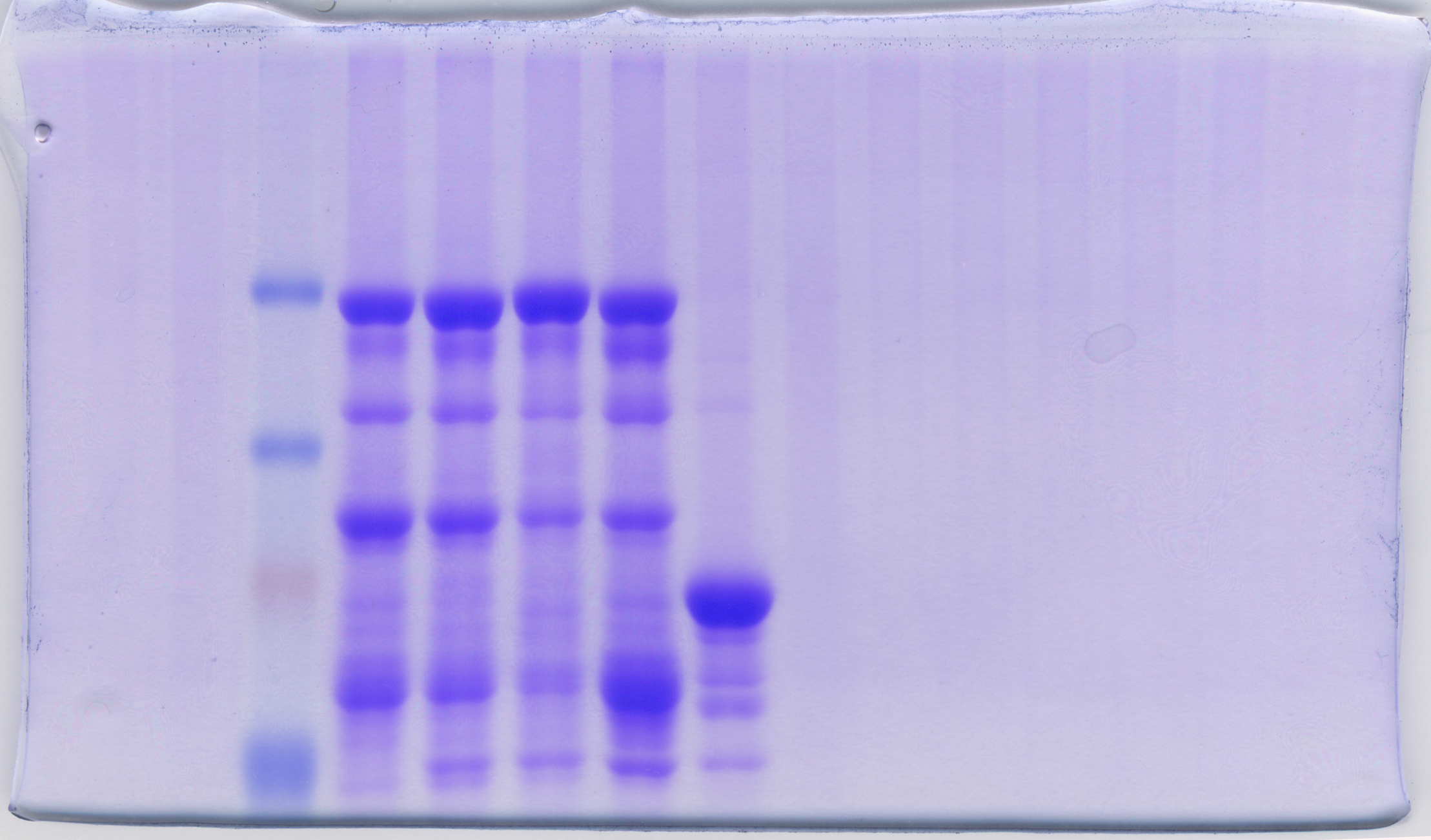

Supplement: Figure 3—source data 3. [file elife-97896-fig3-data3.zip › Figure 3-Source Data 3-14. /Figure 3-Source Data 9. Full raw unedited Coomassie gel (input, left) for Figure 3C.tif]

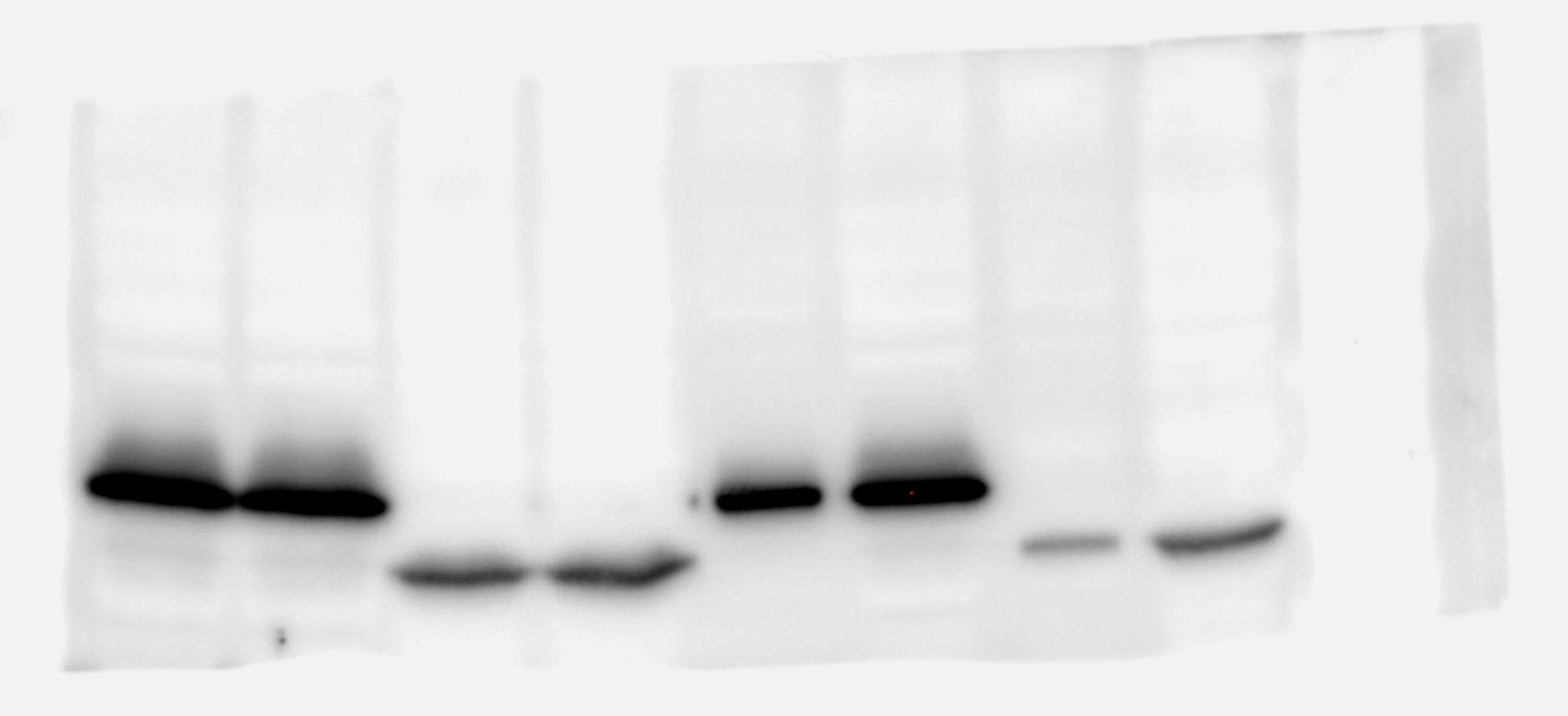

Supplement: Figure 3—source data 3. [file elife-97896-fig3-data3.zip › Figure 3-Source Data 3-14. /Figure 3-Source Data 4. Full raw unedited blot (Pmk1-HA input) for Figure 3A.tif]

Figure 3-figure supplement 2B.

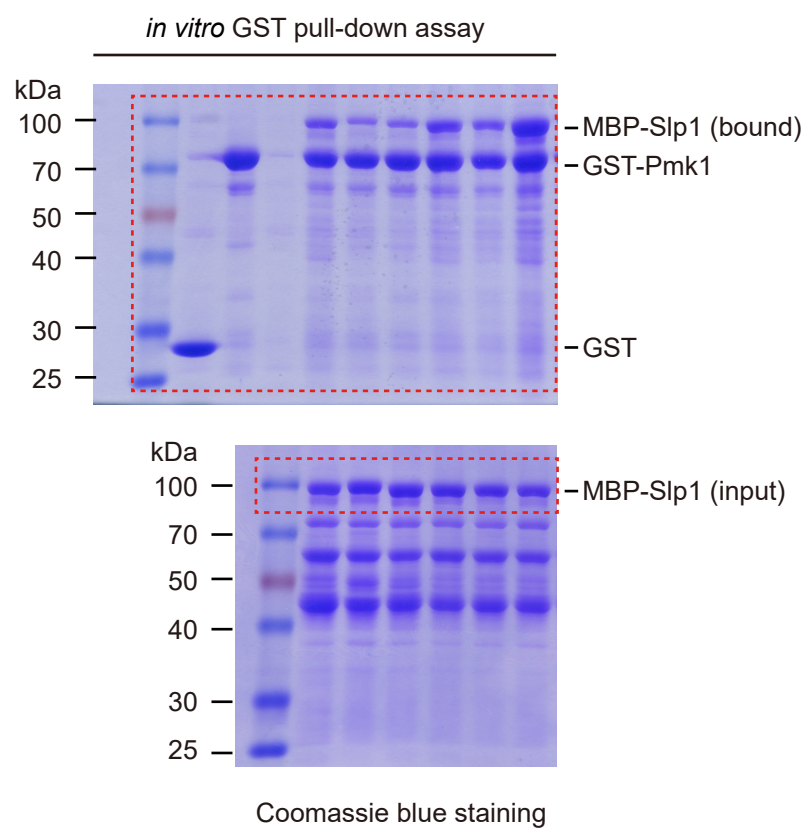

Supplement: Figure 3—figure supplement 2—source data 1. [file elife-97896-fig3-figsupp2-data1.pdf]

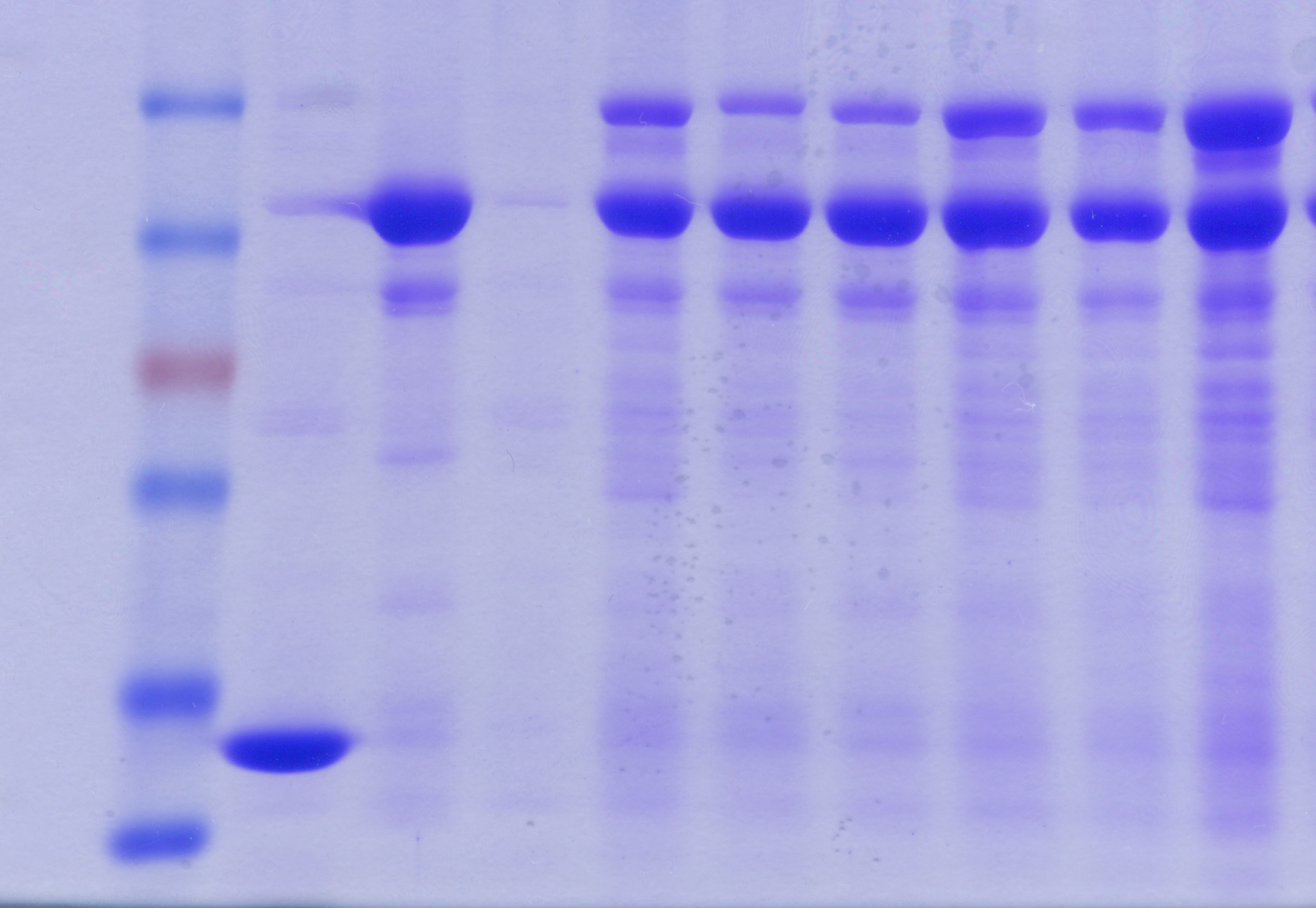

Supplement: Figure 3—figure supplement 2—source data 2. [file elife-97896-fig3-figsupp2-data2.zip › Figure 3-figure supplement 2-Source Data 2. Full raw unedited Coomassie gel #1 for Fig 3-figure suppl 2B.tif]

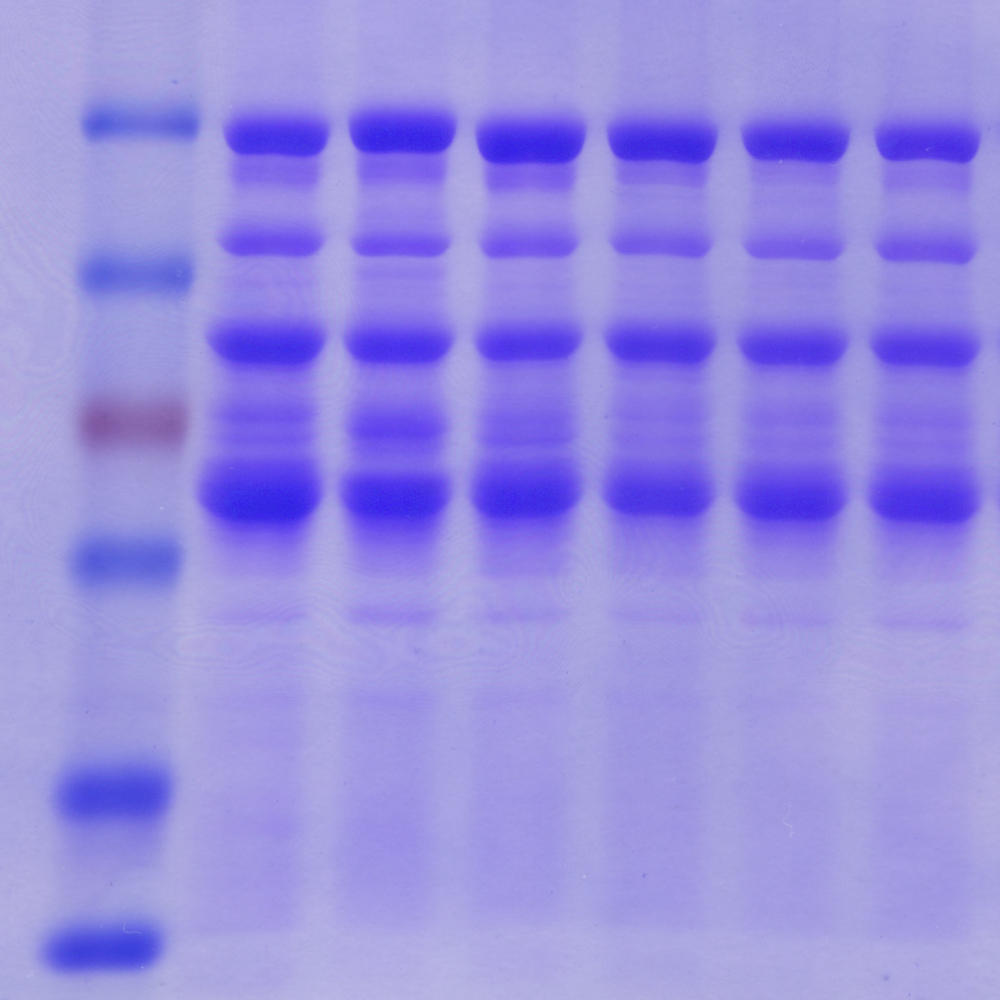

Supplement: Figure 3—figure supplement 2—source data 3. [file elife-97896-fig3-figsupp2-data3.zip › Figure 3-figure supplement 2-Source Data 3. Full raw unedited Coomassie gel #2 for Fig 3-figure suppl 2B.tif]

Figure 4A.

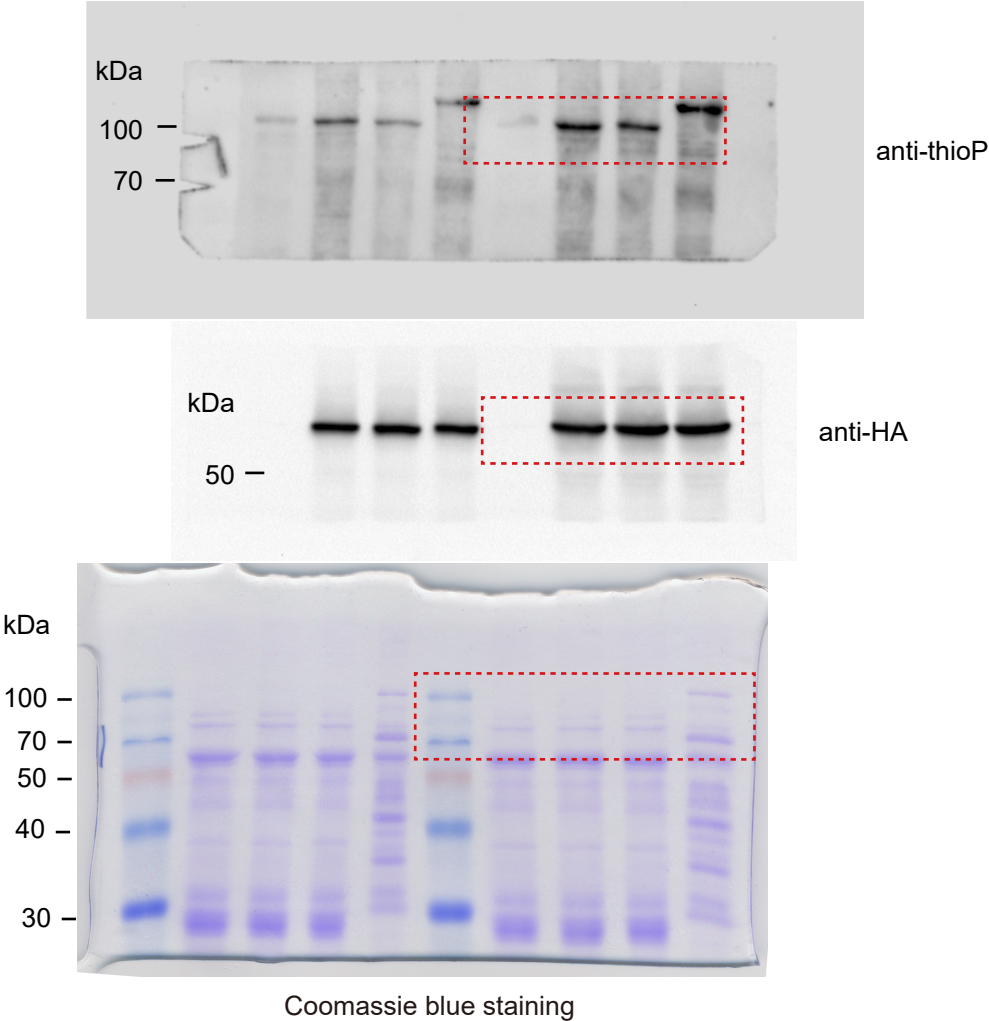

Figure 4C. *in vitro* kinase assay

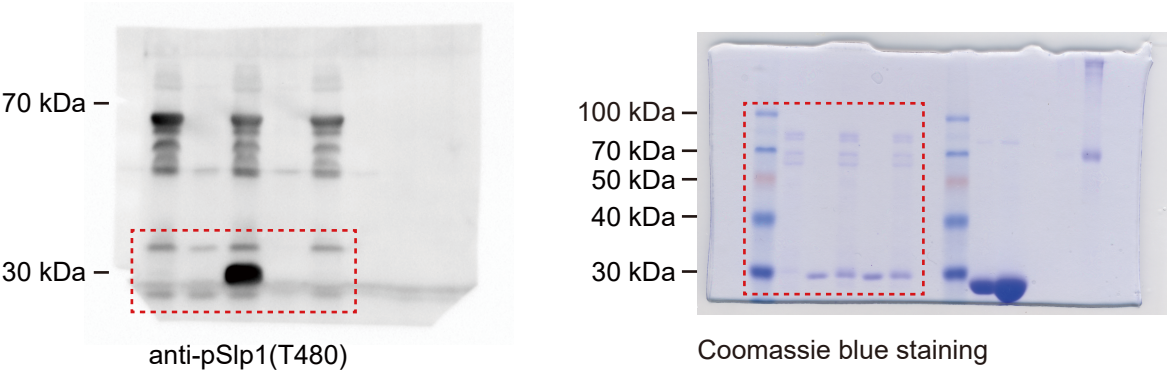

Figure 4D.

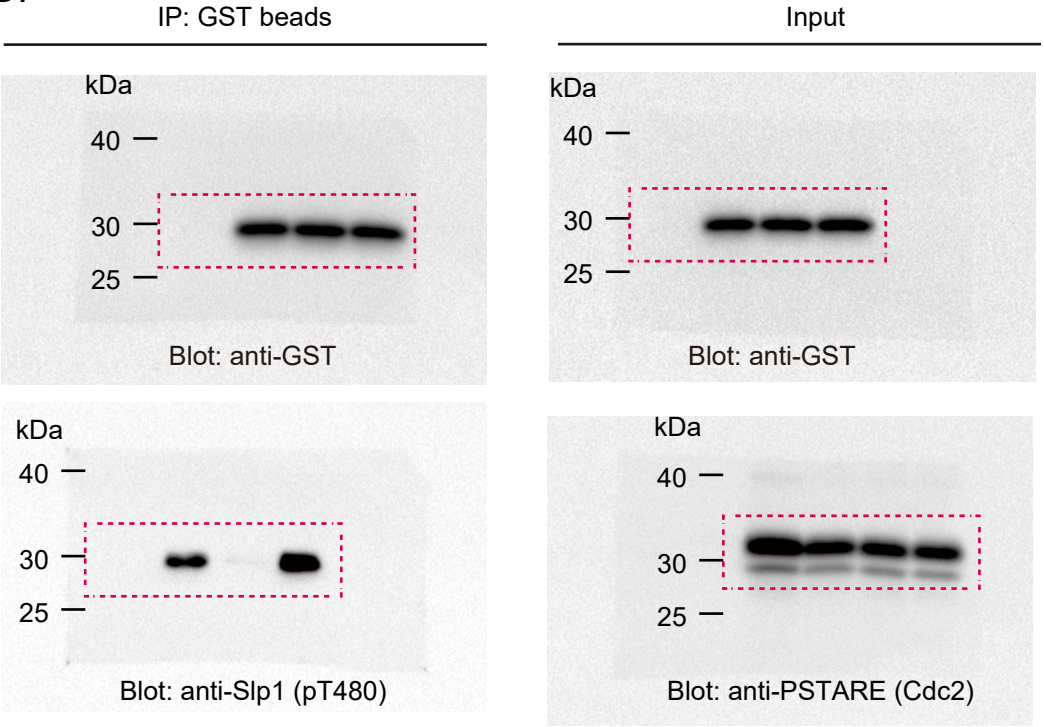

Figure 4E.

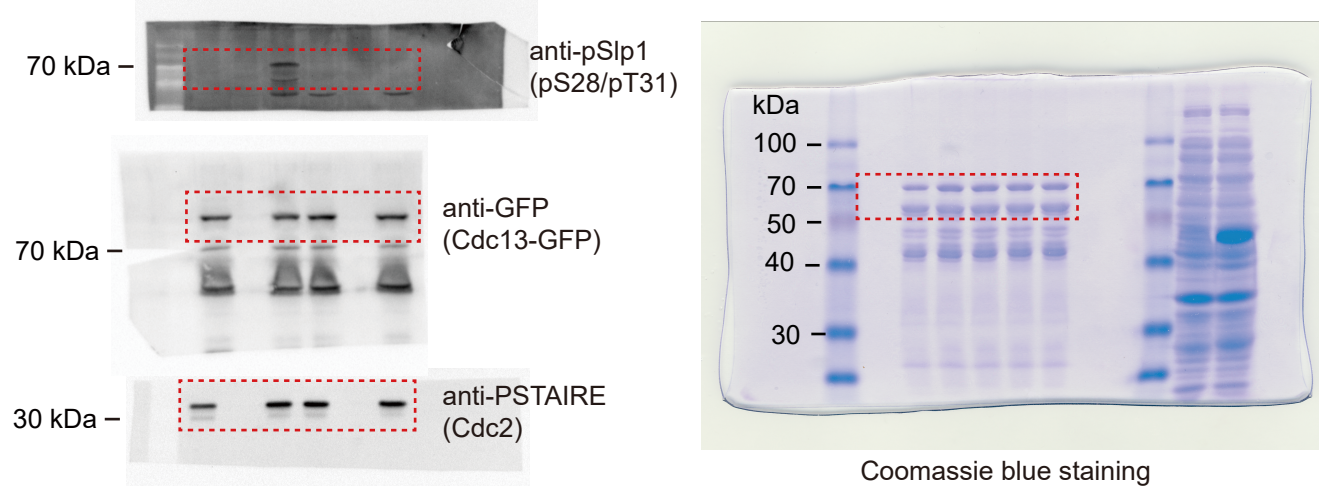

Figure 4F.

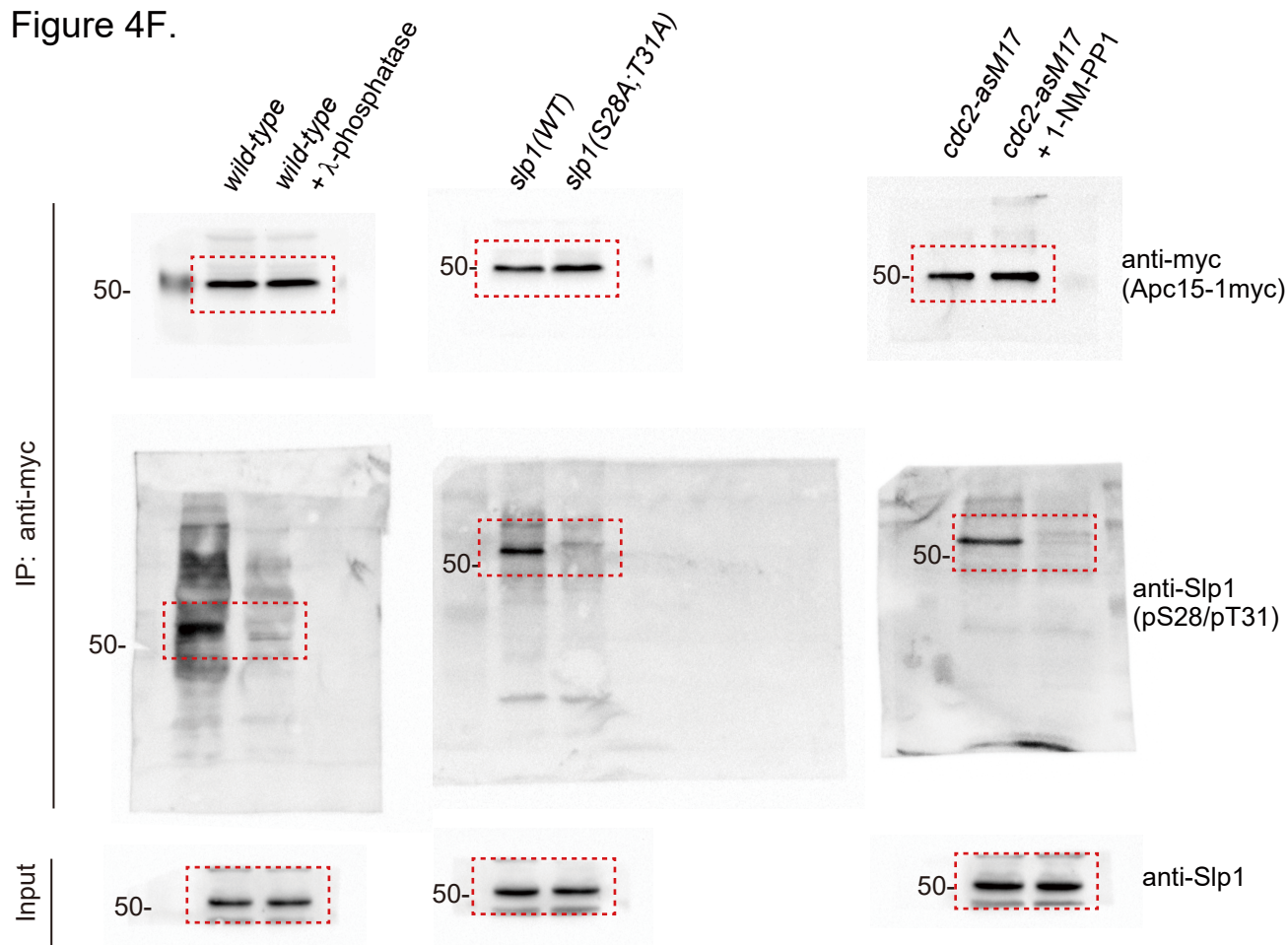

Figure 4G.

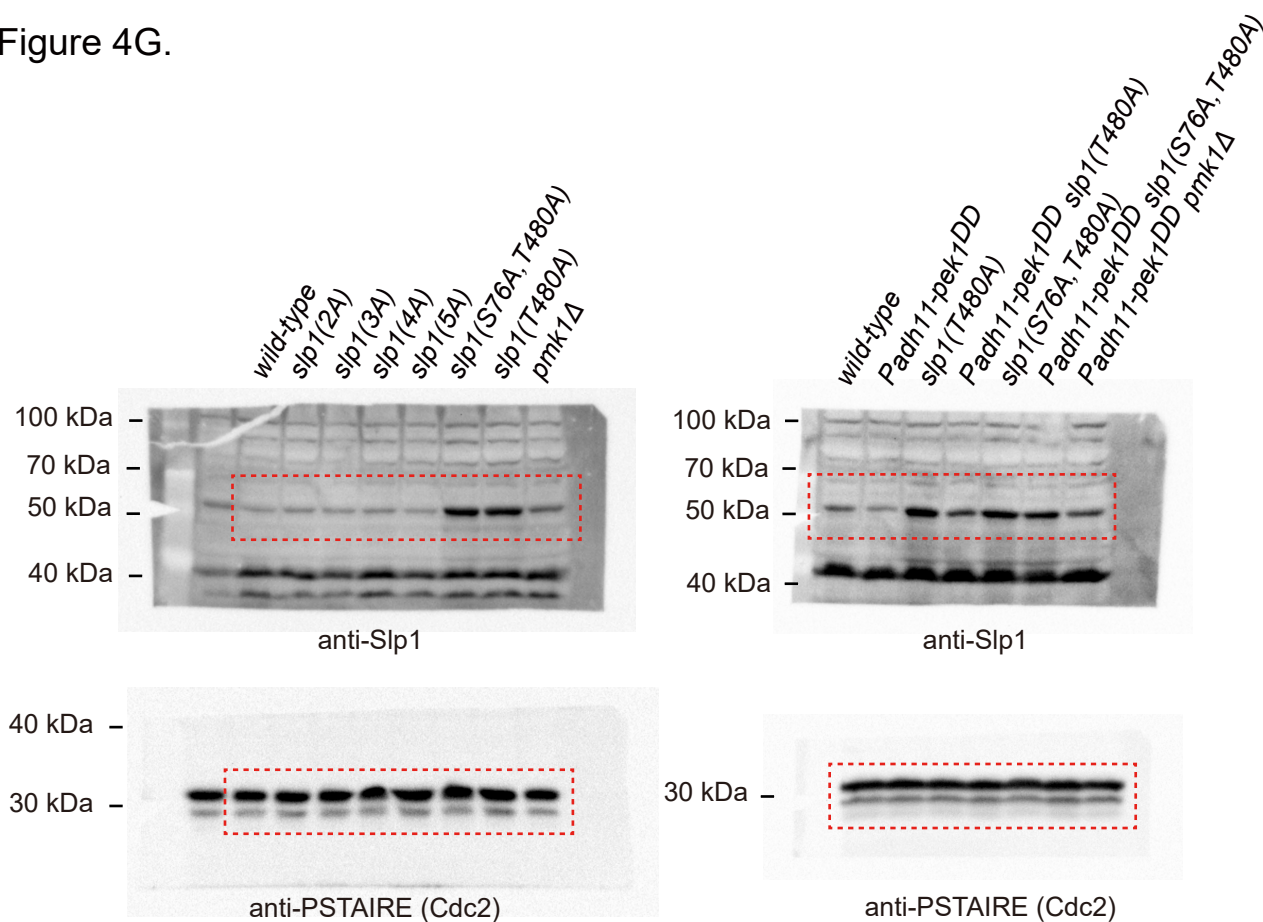

Supplement: Figure 4—source data 1. [file elife-97896-fig4-data1.pdf]

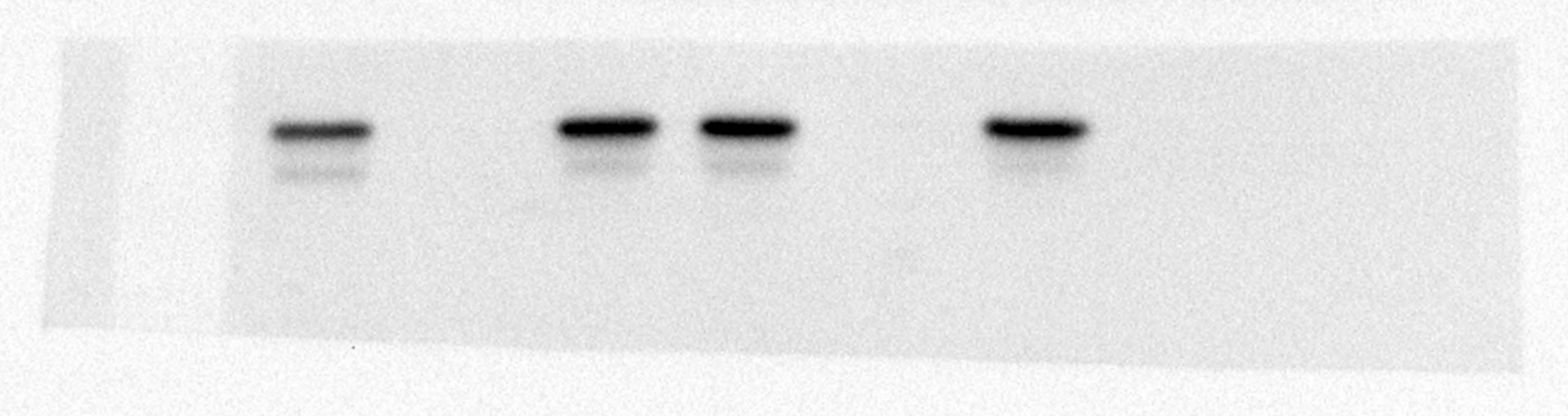

Supplement: Figure 4—source data 3. [file elife-97896-fig4-data3.zip › Figure 4-Source Data 3-28. /Figure 4-Source Data 14. Full raw unedited blot (Cdc2 input) for Figure 4E.tif]

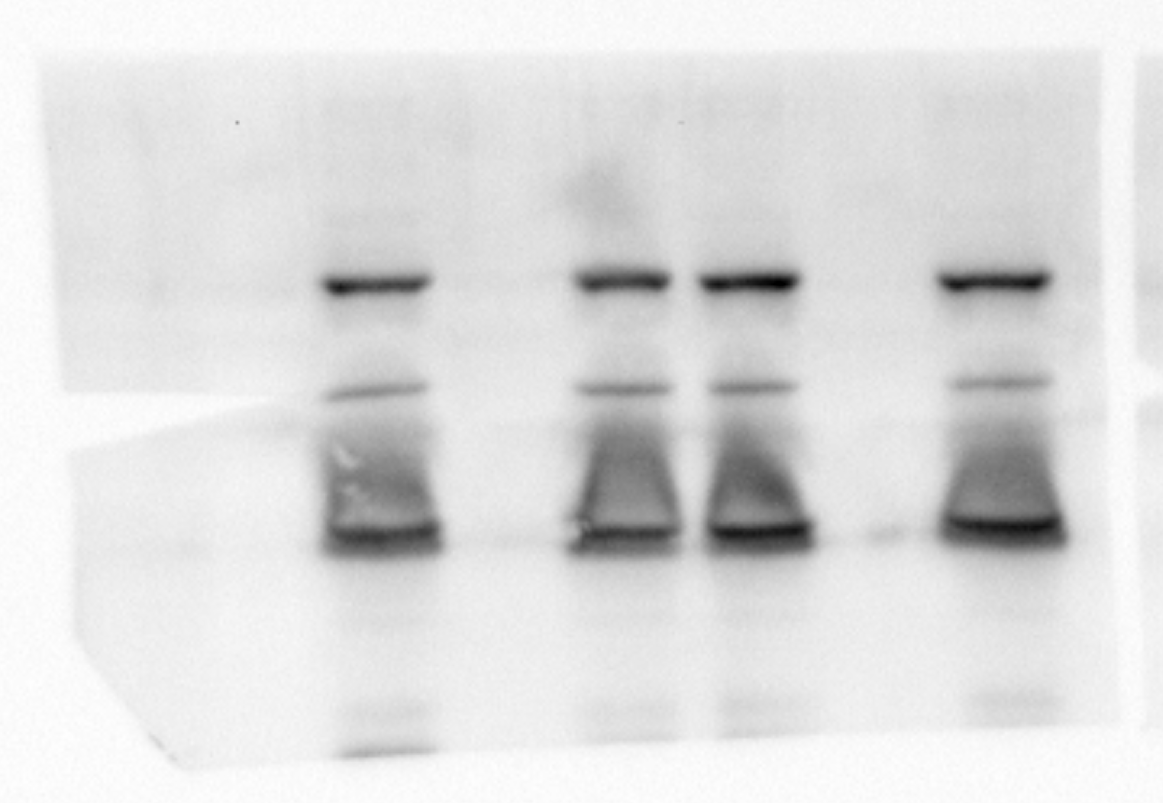

Supplement: Figure 4—source data 3. [file elife-97896-fig4-data3.zip › Figure 4-Source Data 3-28. /Figure 4-Source Data 13. Full raw unedited blot (anti-GFP) for Figure 4E.tif]

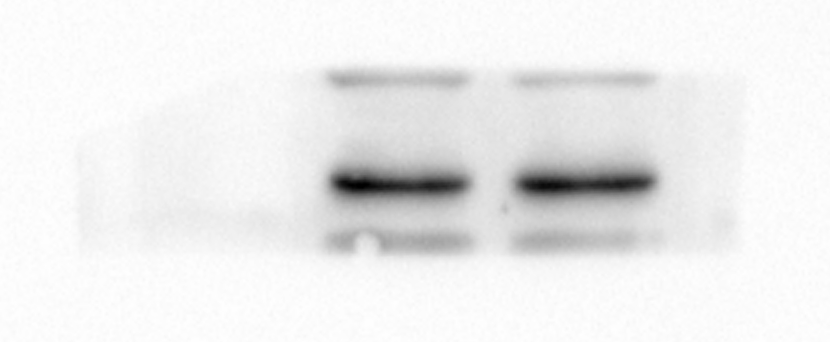

Supplement: Figure 4—source data 3. [file elife-97896-fig4-data3.zip › Figure 4-Source Data 3-28. /Figure 4-Source Data 22. Full raw unedited blot (Slp1 input, left) for Figure 4F.tif]

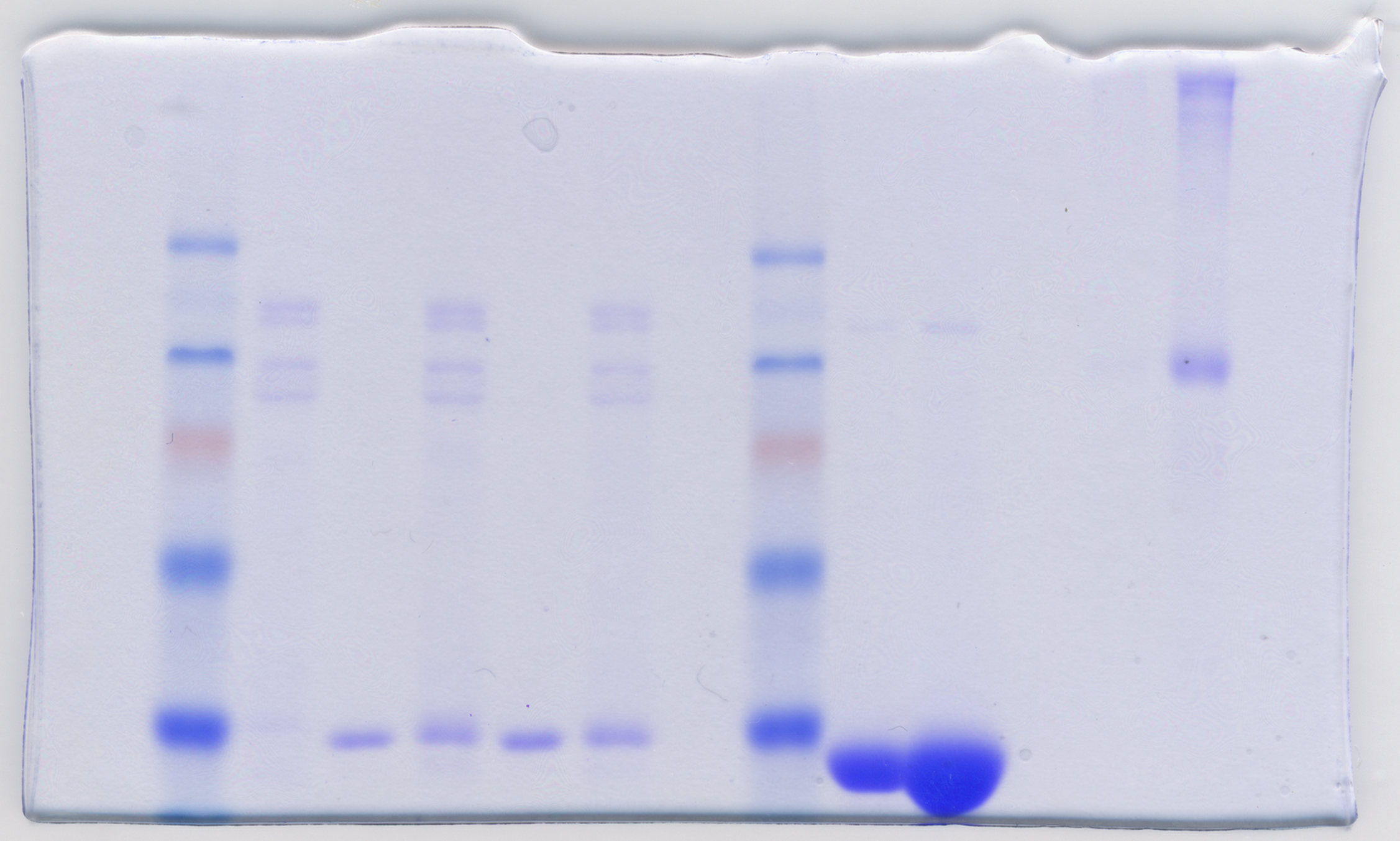

Supplement: Figure 4—source data 3. [file elife-97896-fig4-data3.zip › Figure 4-Source Data 3-28. /Figure 4-Source Data 7. Full raw unedited Coomassie gel (GST-fusions) for Figure 4C.tif]

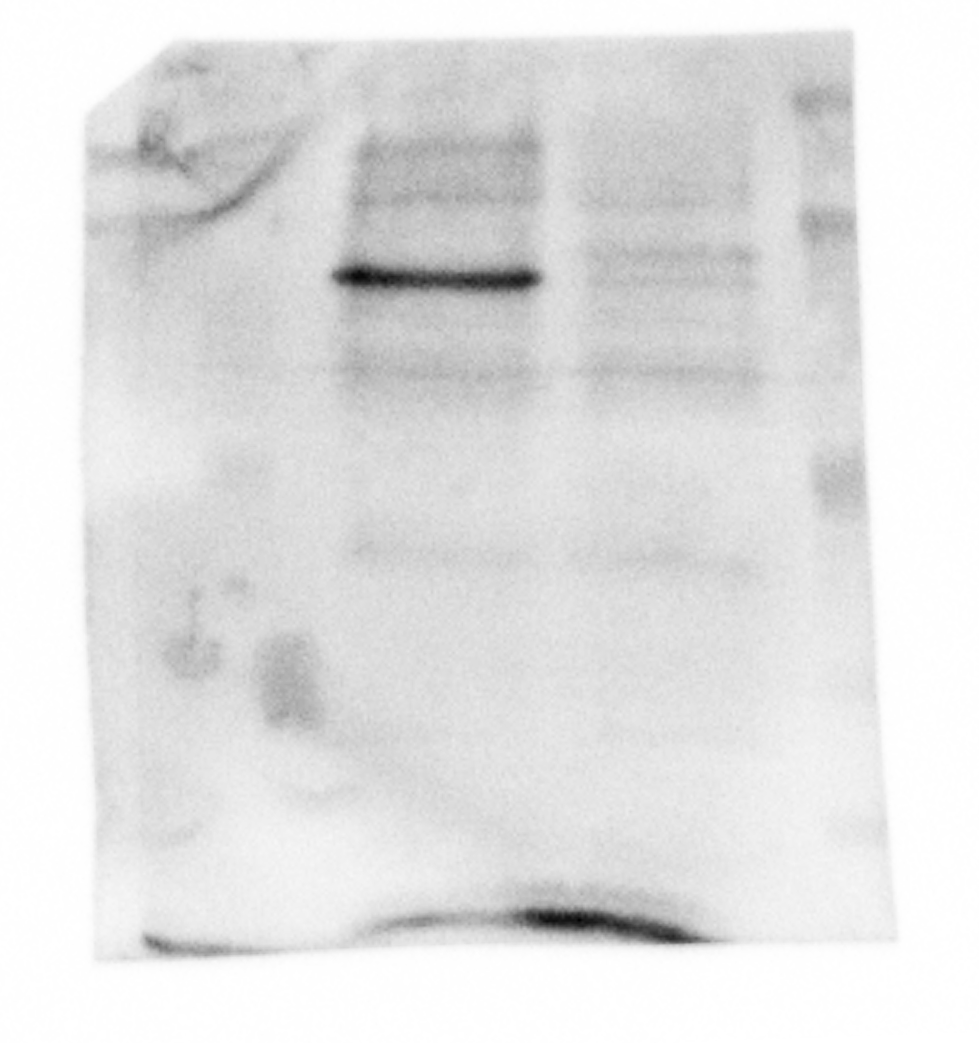

Supplement: Figure 4—source data 3. [file elife-97896-fig4-data3.zip › Figure 4-Source Data 3-28. /Figure 4-Source Data 21. Full raw unedited blot (anti-pS28;pT31 after IP, right) for Figure 4F.tif]

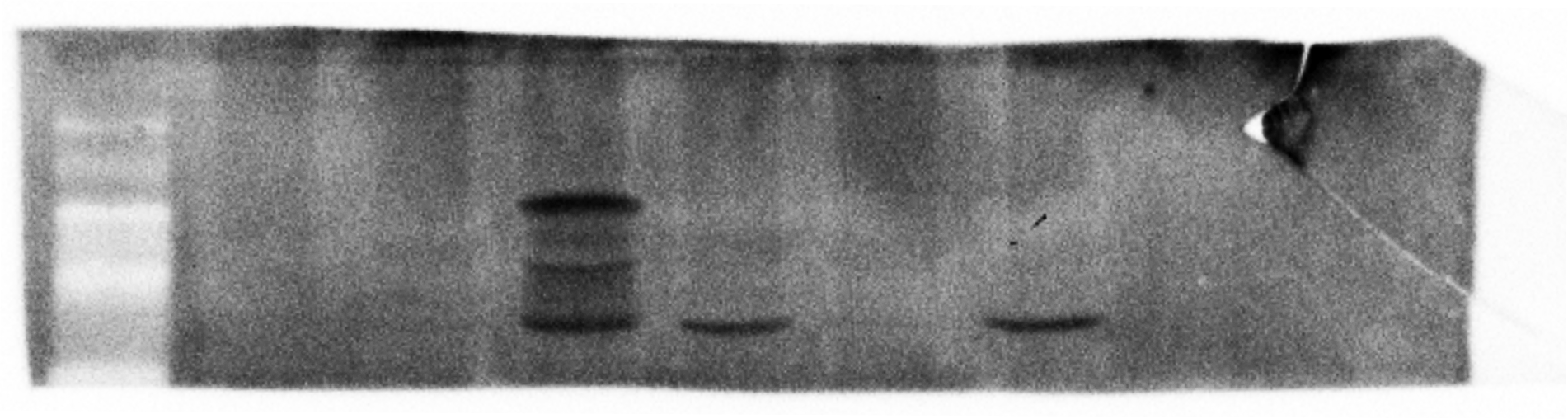

Supplement: Figure 4—source data 3. [file elife-97896-fig4-data3.zip › Figure 4-Source Data 3-28. /Figure 4-Source Data 12. Full raw unedited blot (anti-pS28;pT31) for Figure 4E.tif]

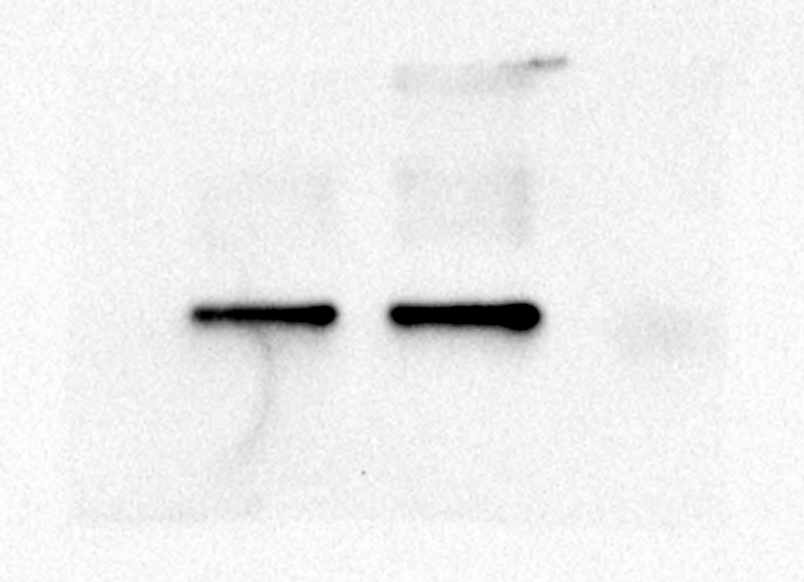

Supplement: Figure 4—source data 3. [file elife-97896-fig4-data3.zip › Figure 4-Source Data 3-28. /Figure 4-Source Data 18. Full raw unedited blot (anti-myc IP, right) for Figure 4F.tif]

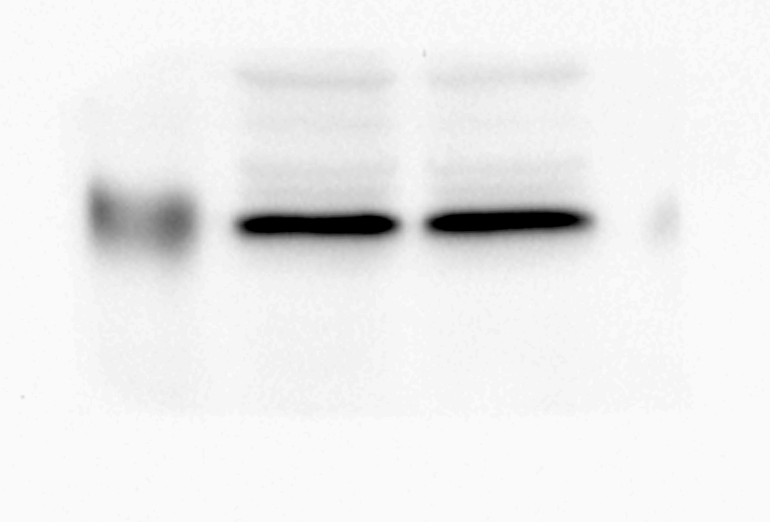

Supplement: Figure 4—source data 3. [file elife-97896-fig4-data3.zip › Figure 4-Source Data 3-28. /Figure 4-Source Data 16. Full raw unedited blot (anti-myc IP, left) for Figure 4F.tif]

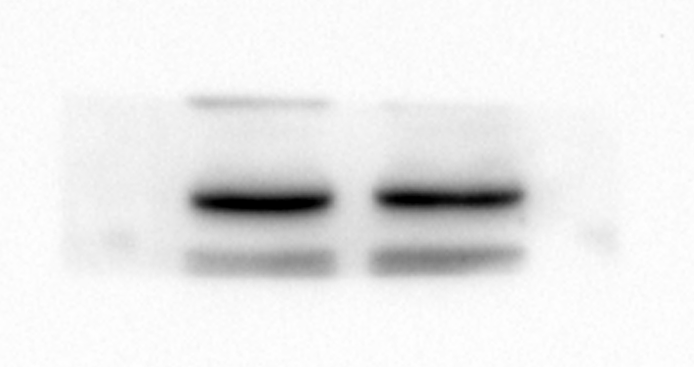

Supplement: Figure 4—source data 3. [file elife-97896-fig4-data3.zip › Figure 4-Source Data 3-28. /Figure 4-Source Data 23. Full raw unedited blot (Slp1 input, middle) for Figure 4F.tif]

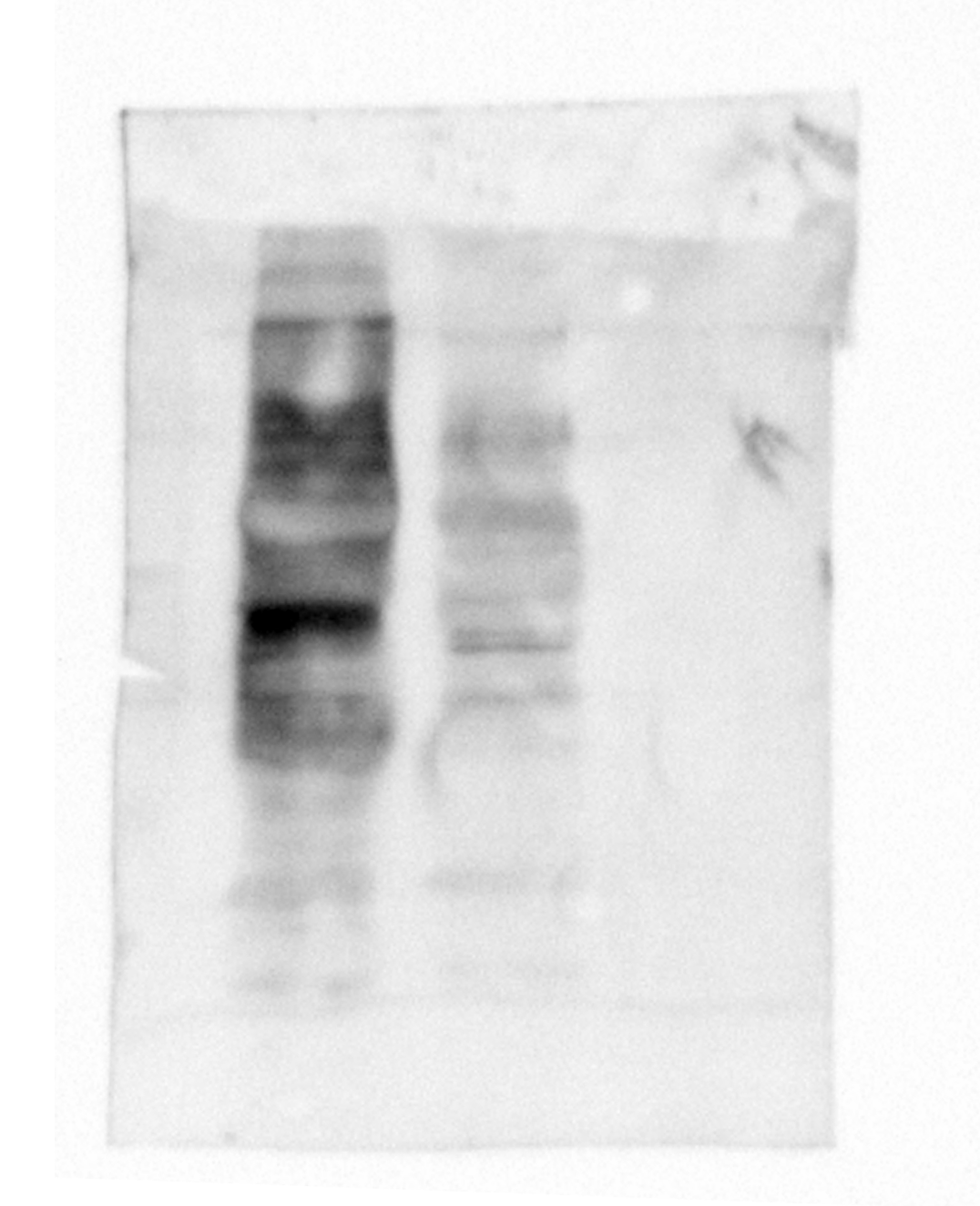

Supplement: Figure 4—source data 3. [file elife-97896-fig4-data3.zip › Figure 4-Source Data 3-28. /Figure 4-Source Data 19. Full raw unedited blot (anti-pS28;pT31 after IP, left) for Figure 4F.tif]

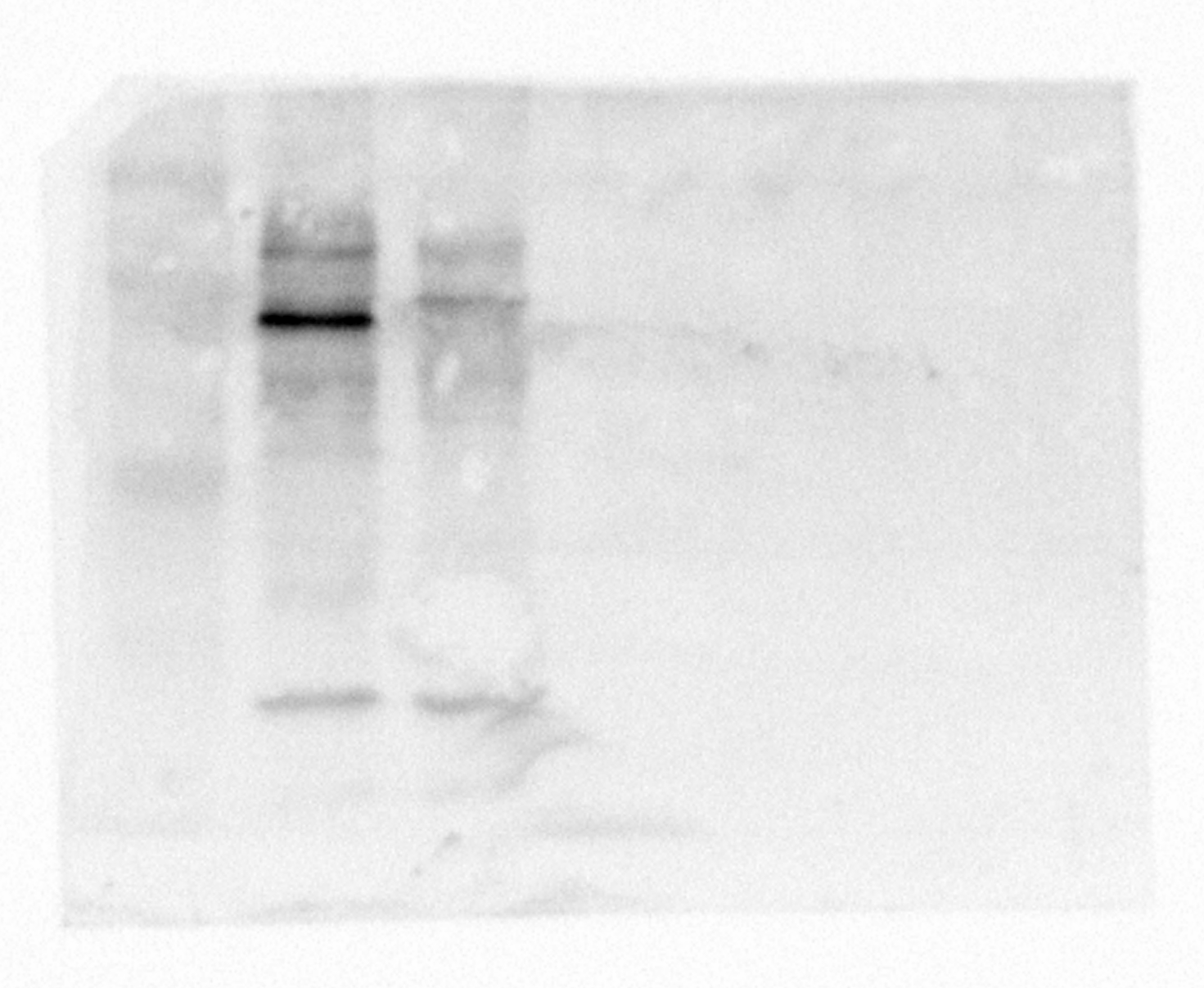

Supplement: Figure 4—source data 3. [file elife-97896-fig4-data3.zip › Figure 4-Source Data 3-28. /Figure 4-Source Data 20. Full raw unedited blot (anti-pS28;pT31 after IP, middle) for Figure 4F.tif]

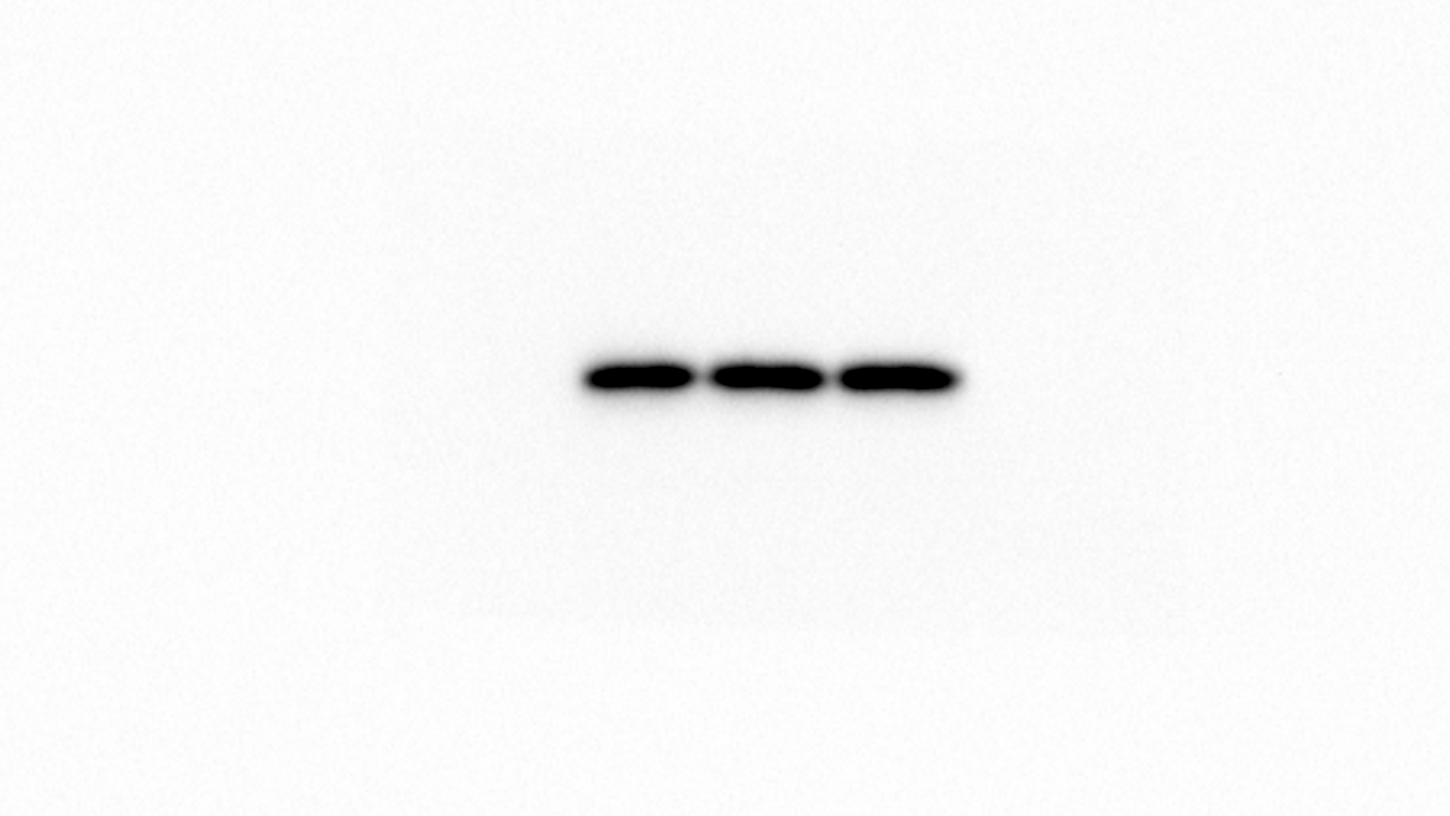

Supplement: Figure 4—source data 3. [file elife-97896-fig4-data3.zip › Figure 4-Source Data 3-28. /Figure 4-Source Data 10. Full raw unedited blot (anti-GST, input) for Figure 4D.tif]

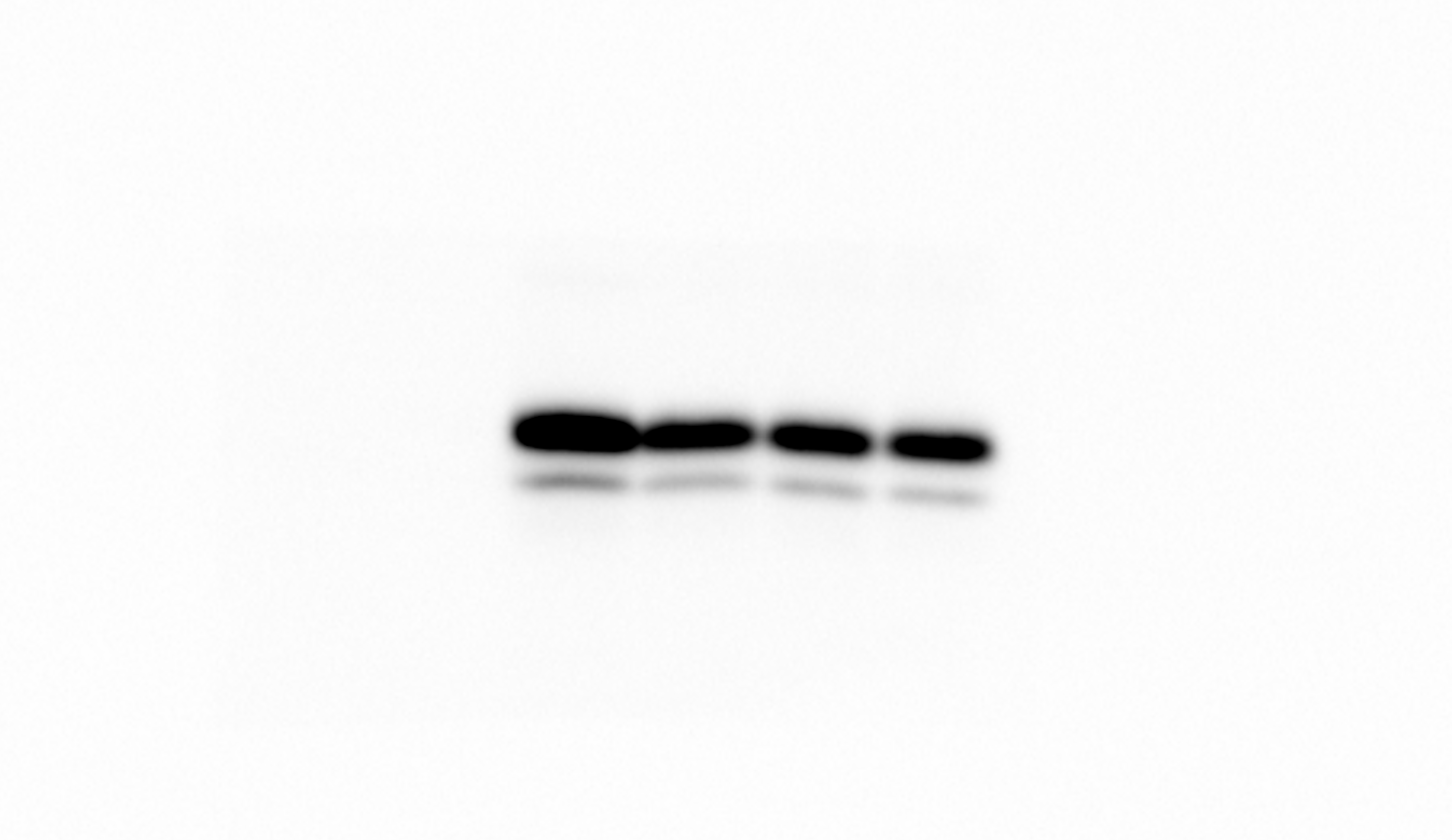

Supplement: Figure 4—source data 3. [file elife-97896-fig4-data3.zip › Figure 4-Source Data 3-28. /Figure 4-Source Data 11. Full raw unedited blot (Cdc2, input) for Figure 4D.tif]

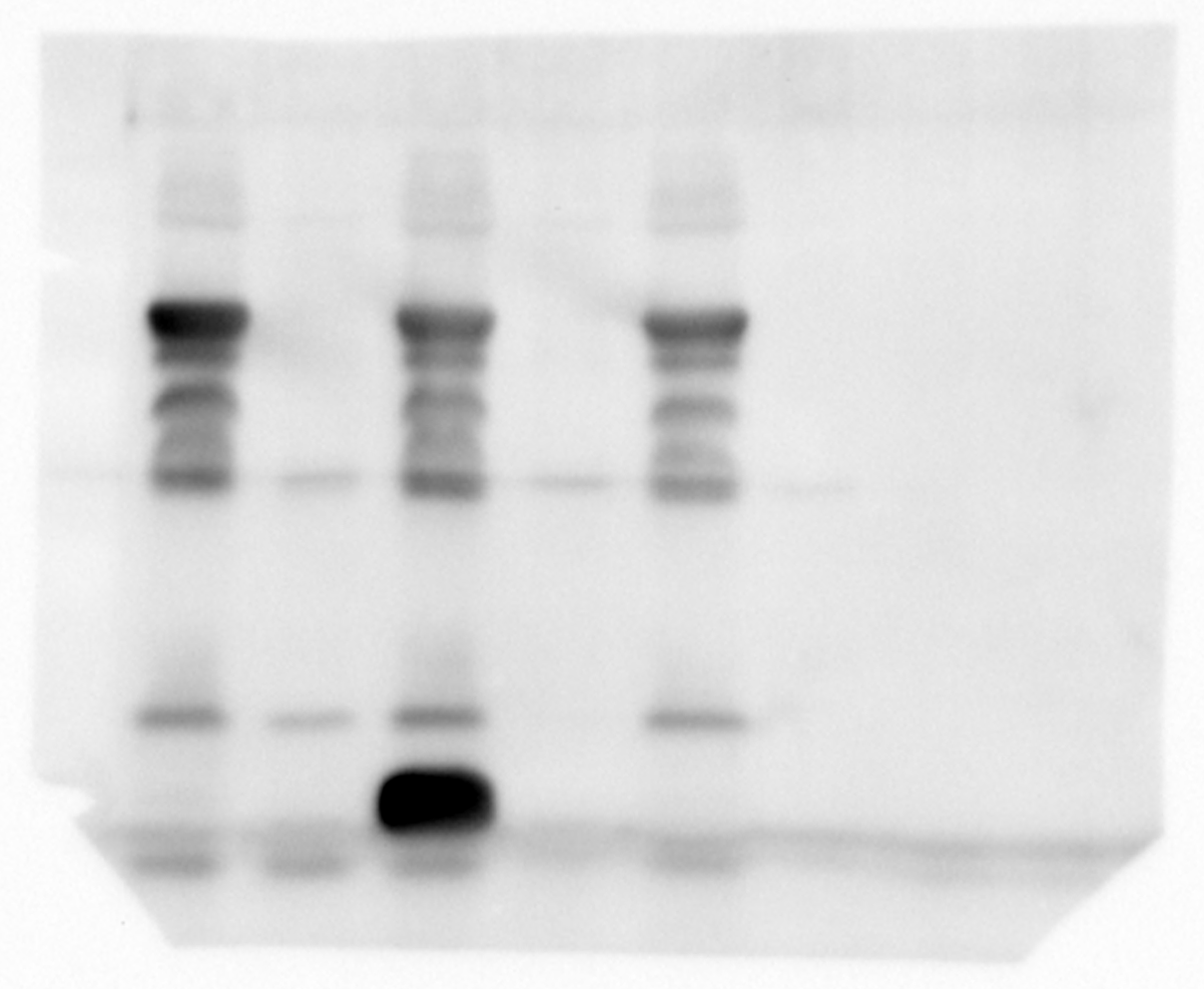

Supplement: Figure 4—source data 3. [file elife-97896-fig4-data3.zip › Figure 4-Source Data 3-28. /Figure 4-Source Data 6. Full raw unedited blot (anti-pT480) for Figure 4C.tif]

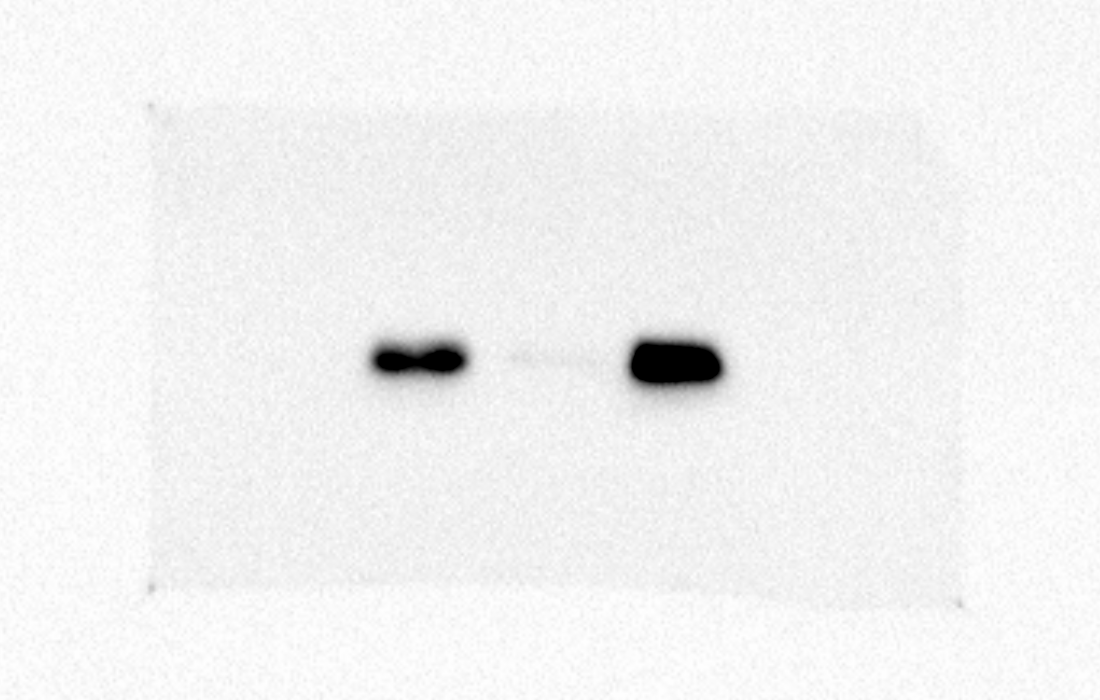

Supplement: Figure 4—source data 3. [file elife-97896-fig4-data3.zip › Figure 4-Source Data 3-28. /Figure 4-Source Data 8. Full raw unedited blot (bead-bound, anti-pT480) for Figure 4D.tif]

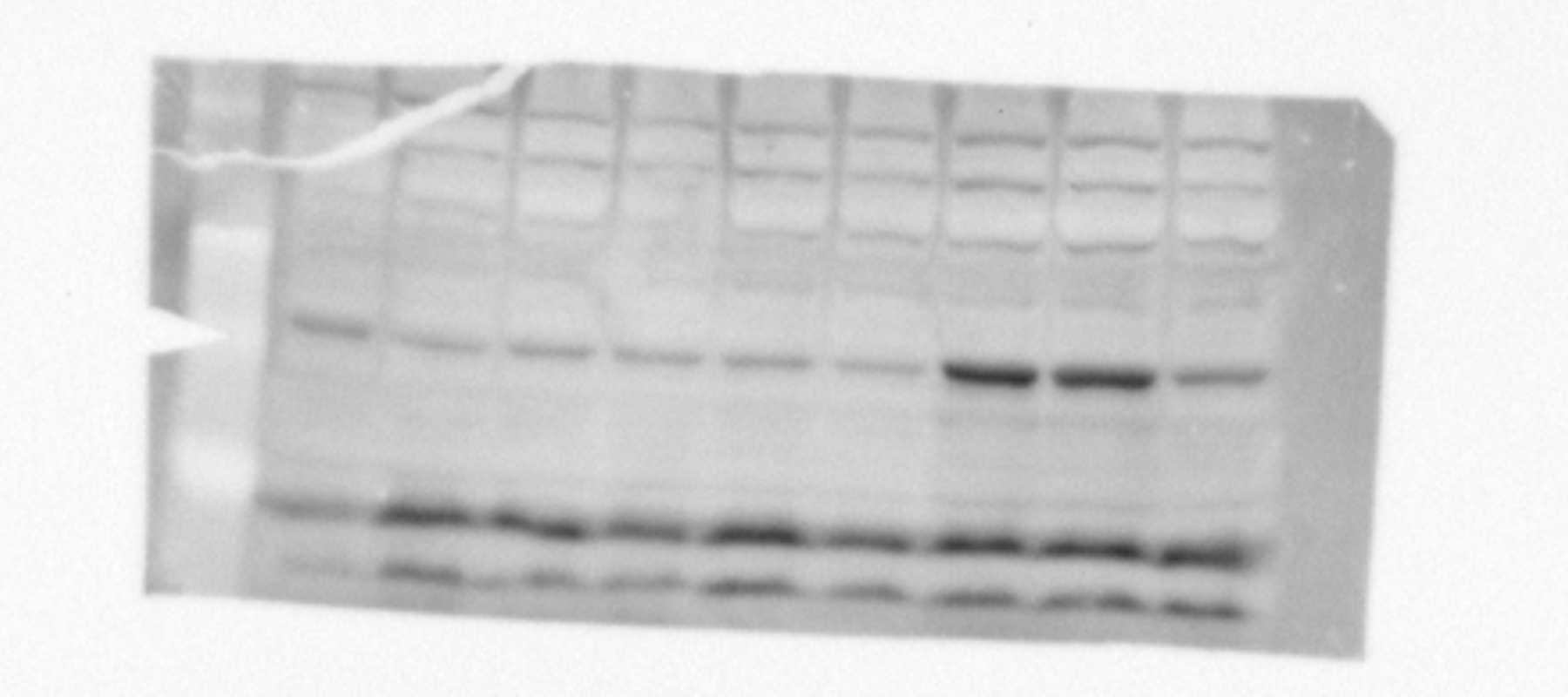

Supplement: Figure 4—source data 3. [file elife-97896-fig4-data3.zip › Figure 4-Source Data 3-28. /Figure 4-Source Data 25. Full raw unedited blot (Slp1, left) for Figure 4G.tif]

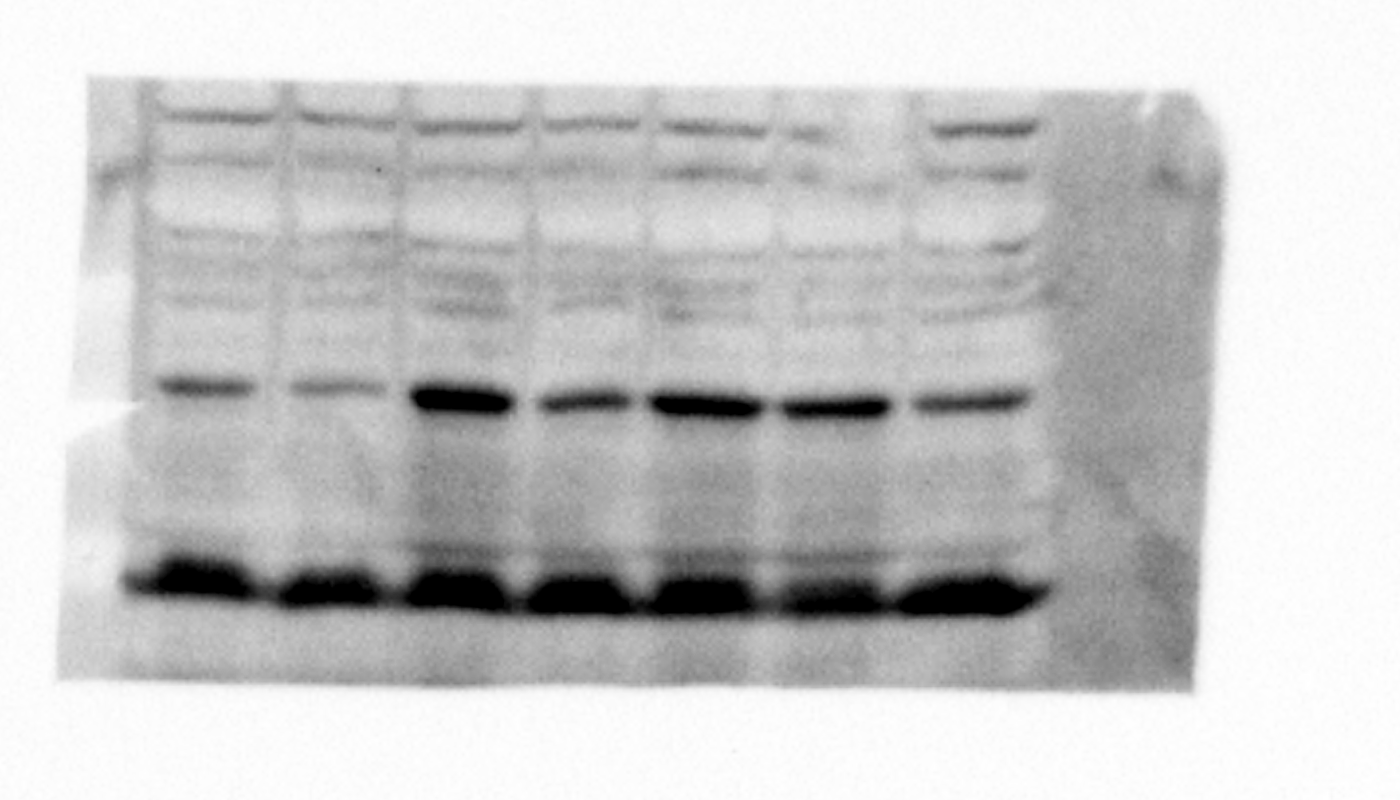

Supplement: Figure 4—source data 3. [file elife-97896-fig4-data3.zip › Figure 4-Source Data 3-28. /Figure 4-Source Data 26. Full raw unedited blot (Slp1, right) for Figure 4G.tif]

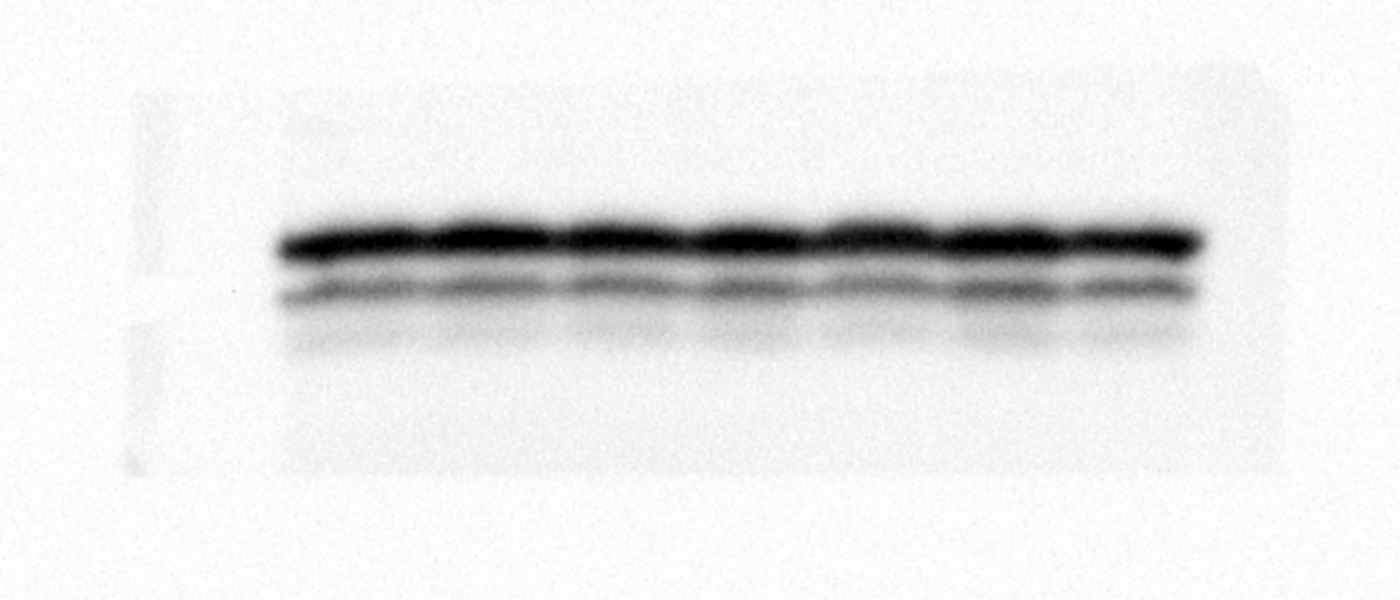

Supplement: Figure 4—source data 3. [file elife-97896-fig4-data3.zip › Figure 4-Source Data 3-28. /Figure 4-Source Data 28. Full raw unedited blot (Cdc2, right) for Figure 4G.tif]

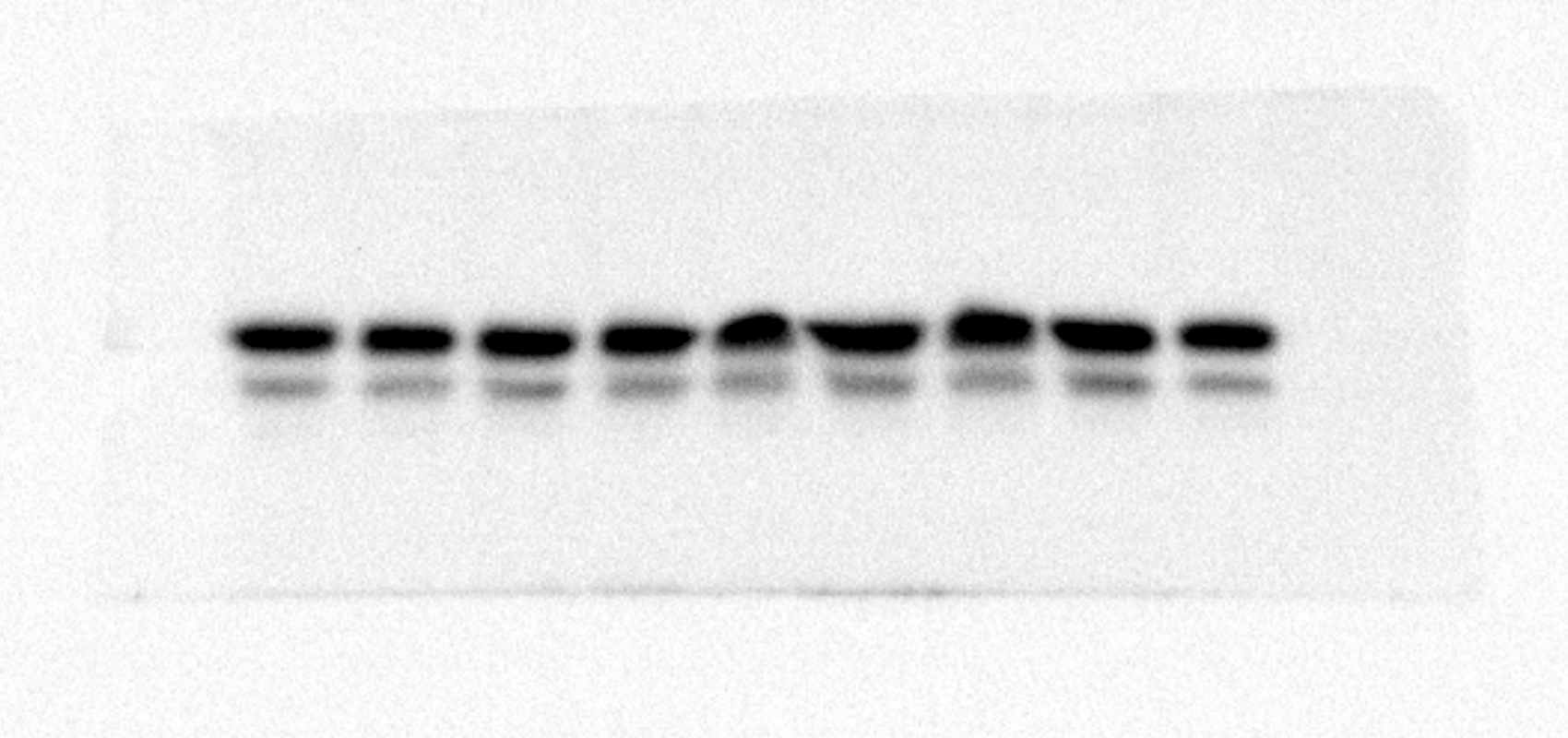

Supplement: Figure 4—source data 3. [file elife-97896-fig4-data3.zip › Figure 4-Source Data 3-28. /Figure 4-Source Data 27. Full raw unedited blot (Cdc2, left) for Figure 4G.tif]

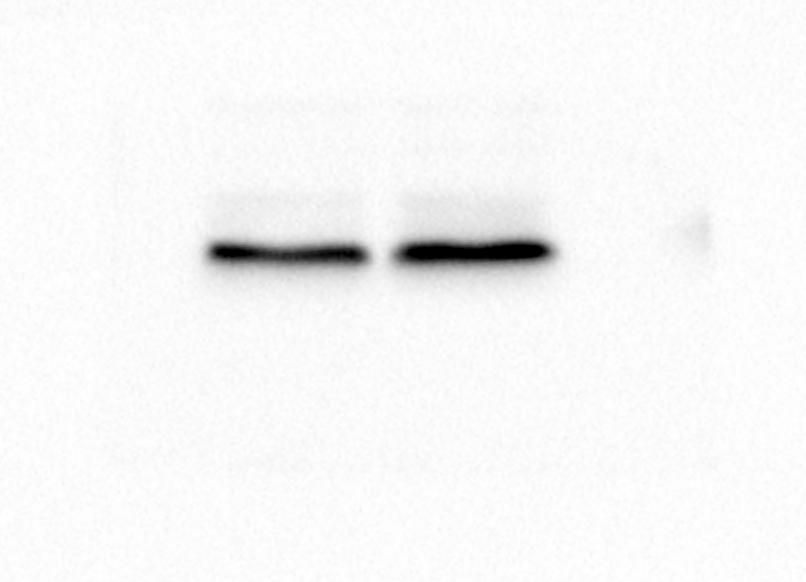

Supplement: Figure 4—source data 3. [file elife-97896-fig4-data3.zip › Figure 4-Source Data 3-28. /Figure 4-Source Data 17. Full raw unedited blot (anti-myc IP, middle) for Figure 4F.tif]

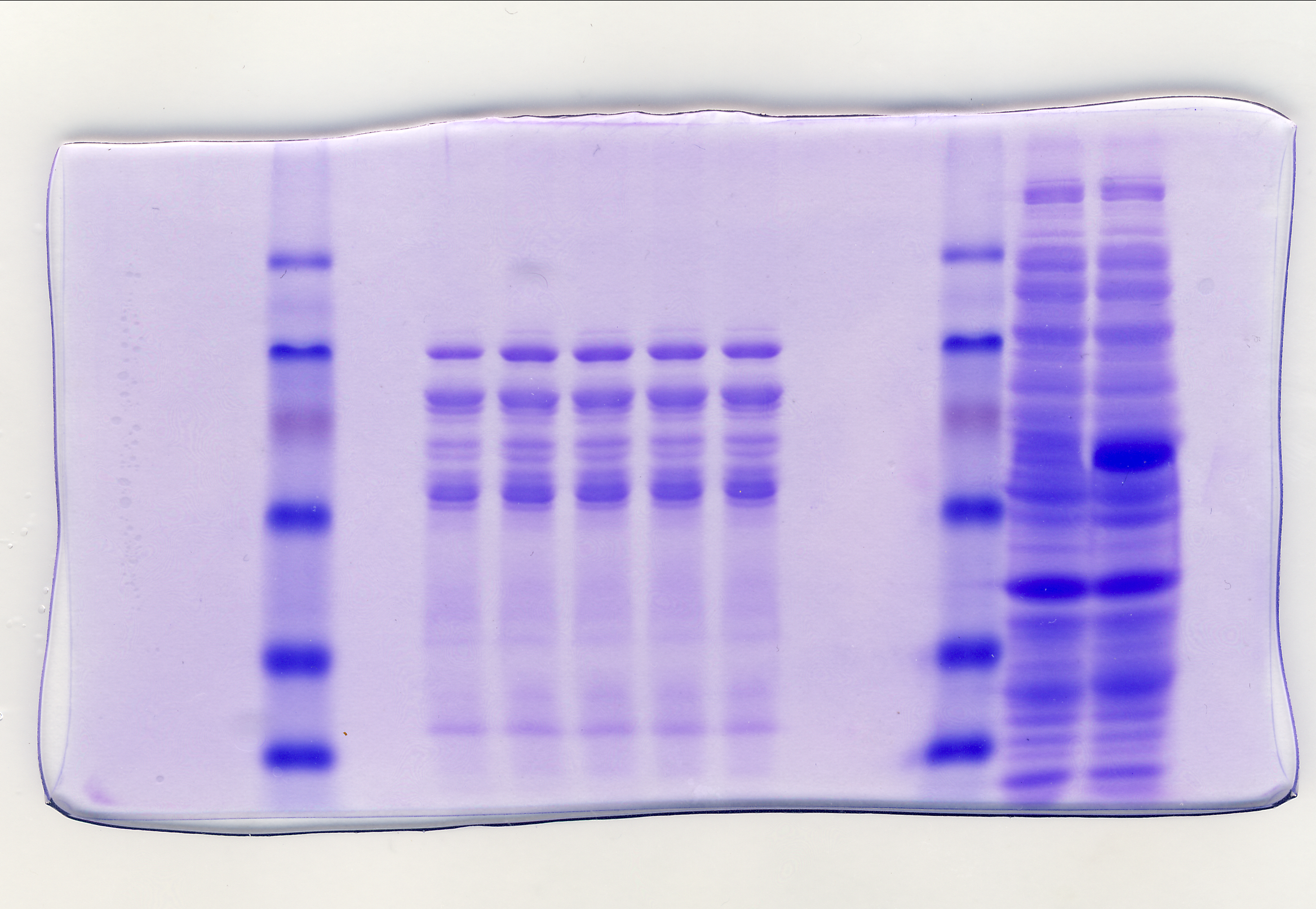

Supplement: Figure 4—source data 3. [file elife-97896-fig4-data3.zip › Figure 4-Source Data 3-28. /Figure 4-Source Data 15. Full raw unedited Coomassie gel (MBP-Slp1(1-190aa)) for Figure 4E.tif]

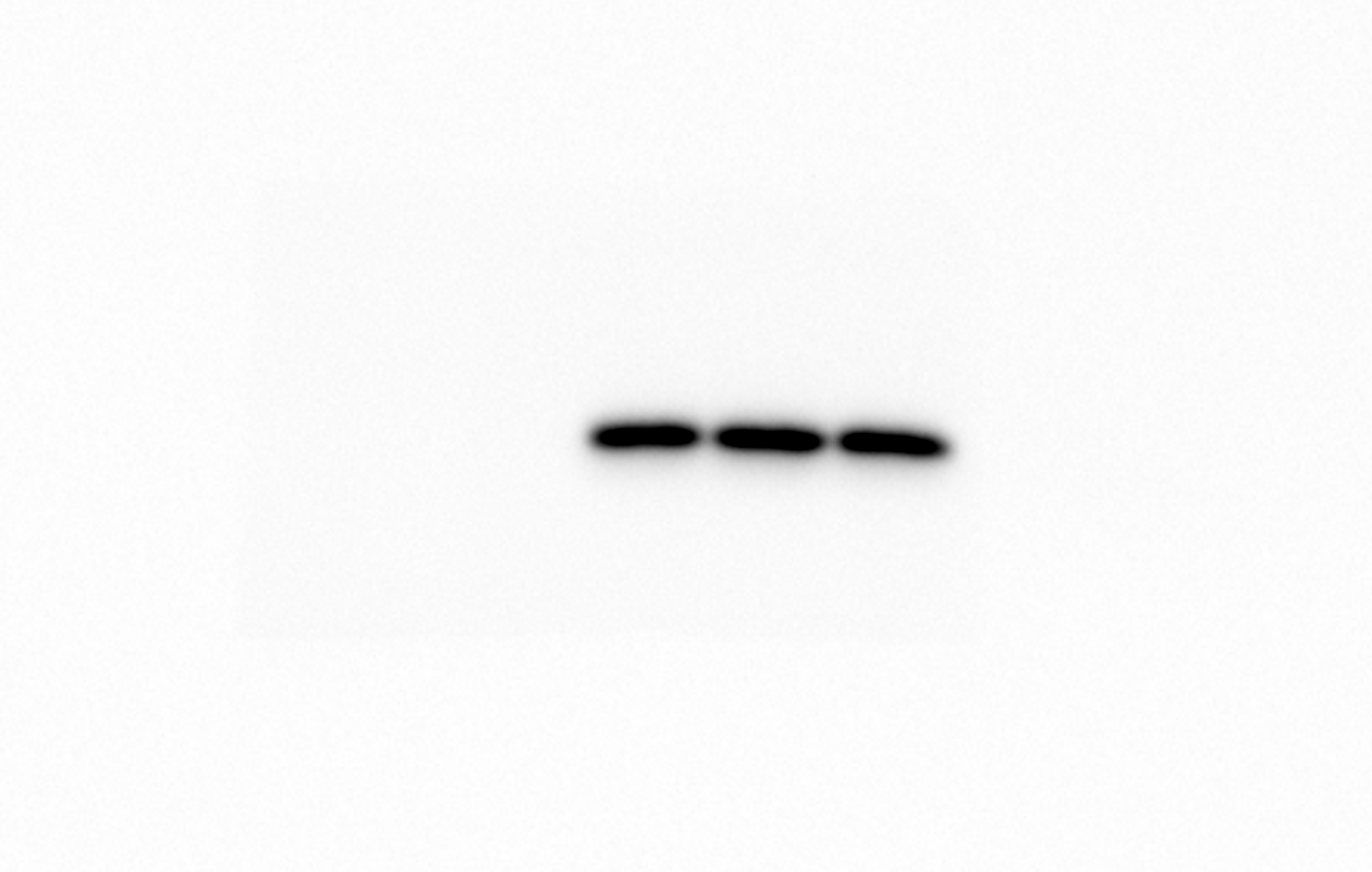

Supplement: Figure 4—source data 3. [file elife-97896-fig4-data3.zip › Figure 4-Source Data 3-28. /Figure 4-Source Data 9. Full raw unedited blot (bead-bound, anti-GST) for Figure 4D.tif]

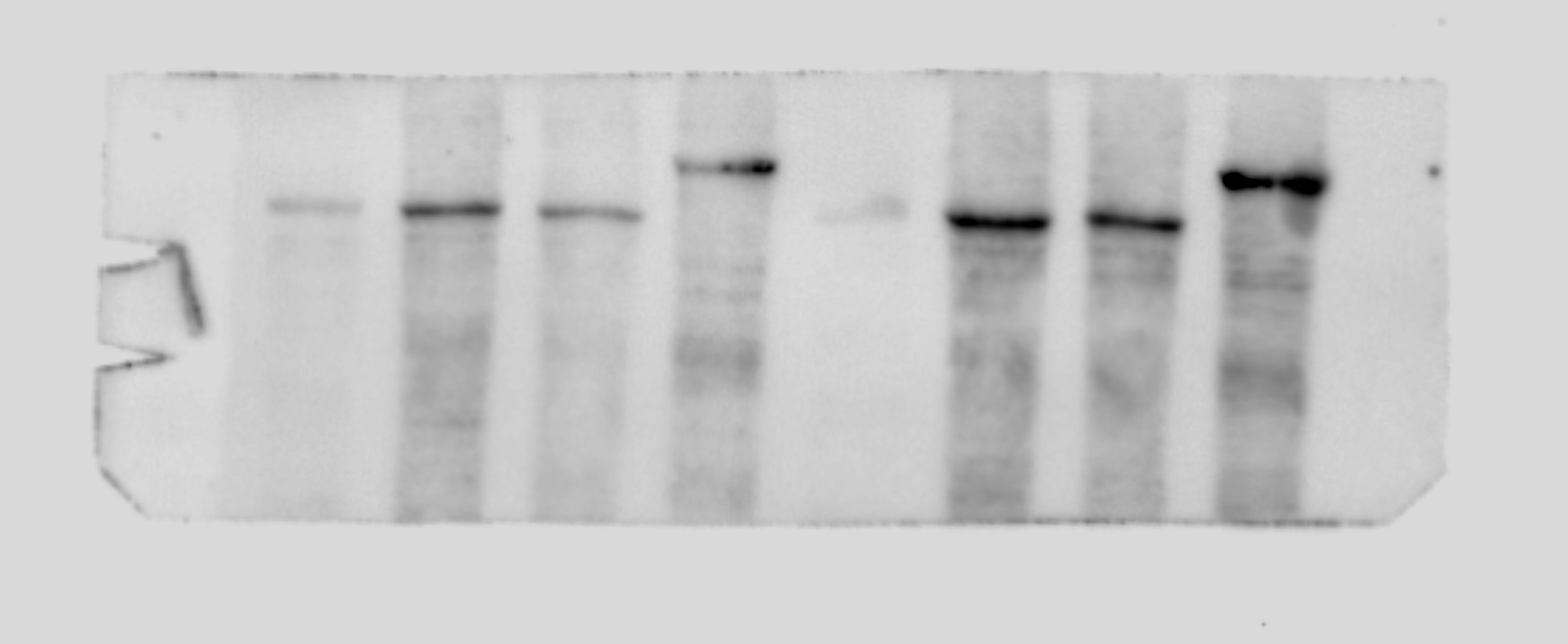

Supplement: Figure 4—source data 3. [file elife-97896-fig4-data3.zip › Figure 4-Source Data 3-28. /Figure 4-Source Data 3. Full raw unedited blot (anti-thioP) for Figure 4A.tif]

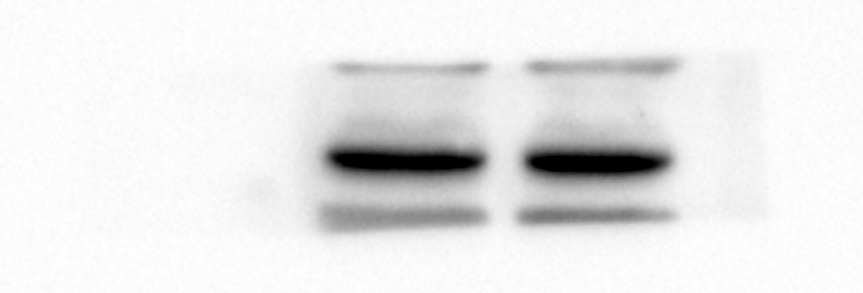

Supplement: Figure 4—source data 3. [file elife-97896-fig4-data3.zip › Figure 4-Source Data 3-28. /Figure 4-Source Data 24. Full raw unedited blot (Slp1 input, right) for Figure 4F.tif]

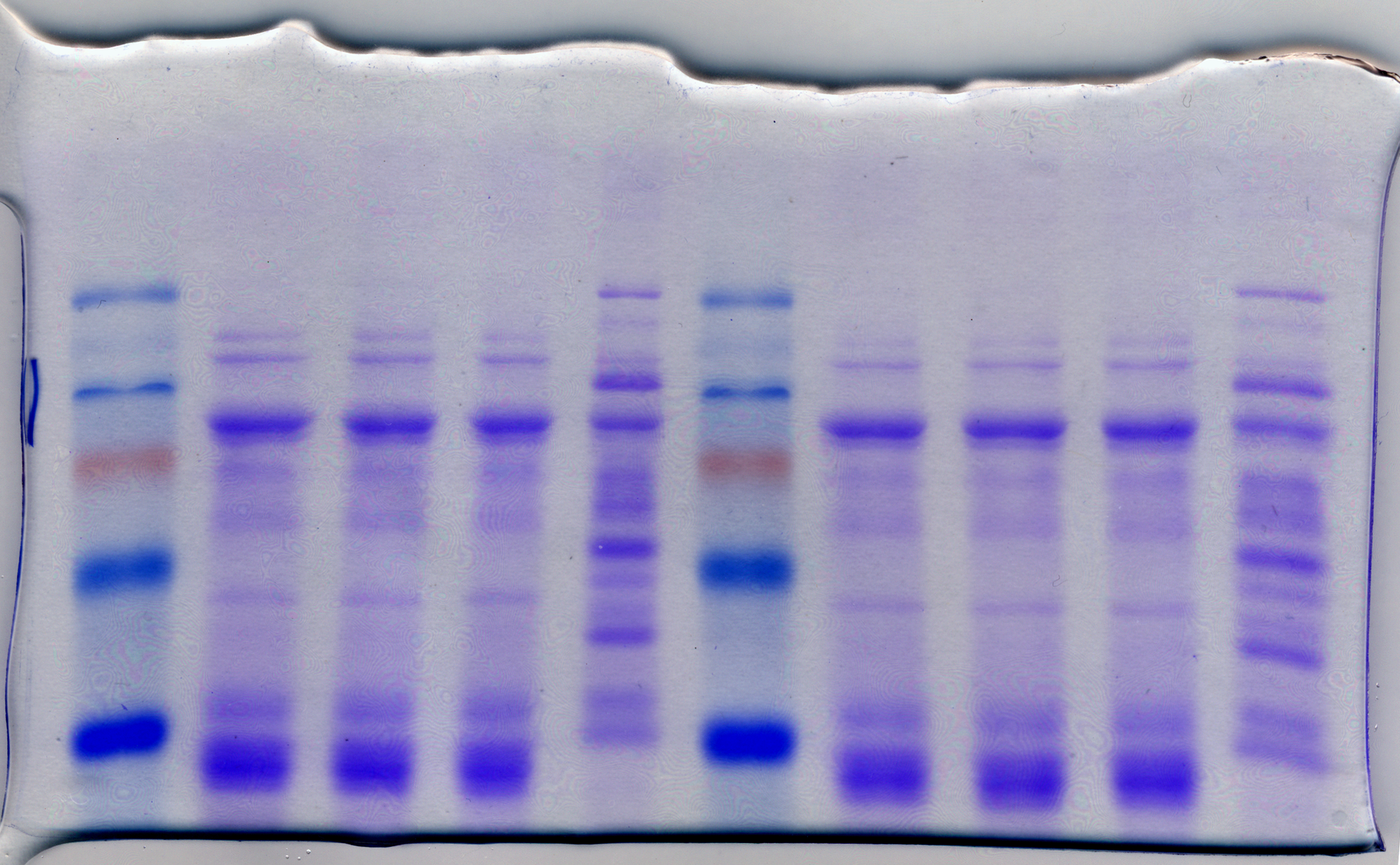

Supplement: Figure 4—source data 3. [file elife-97896-fig4-data3.zip › Figure 4-Source Data 3-28. /Figure 4-Source Data 5. Full raw unedited Coomassie gel (GST-Atf1 & GST-Slp1) for Figure 4A.tif]

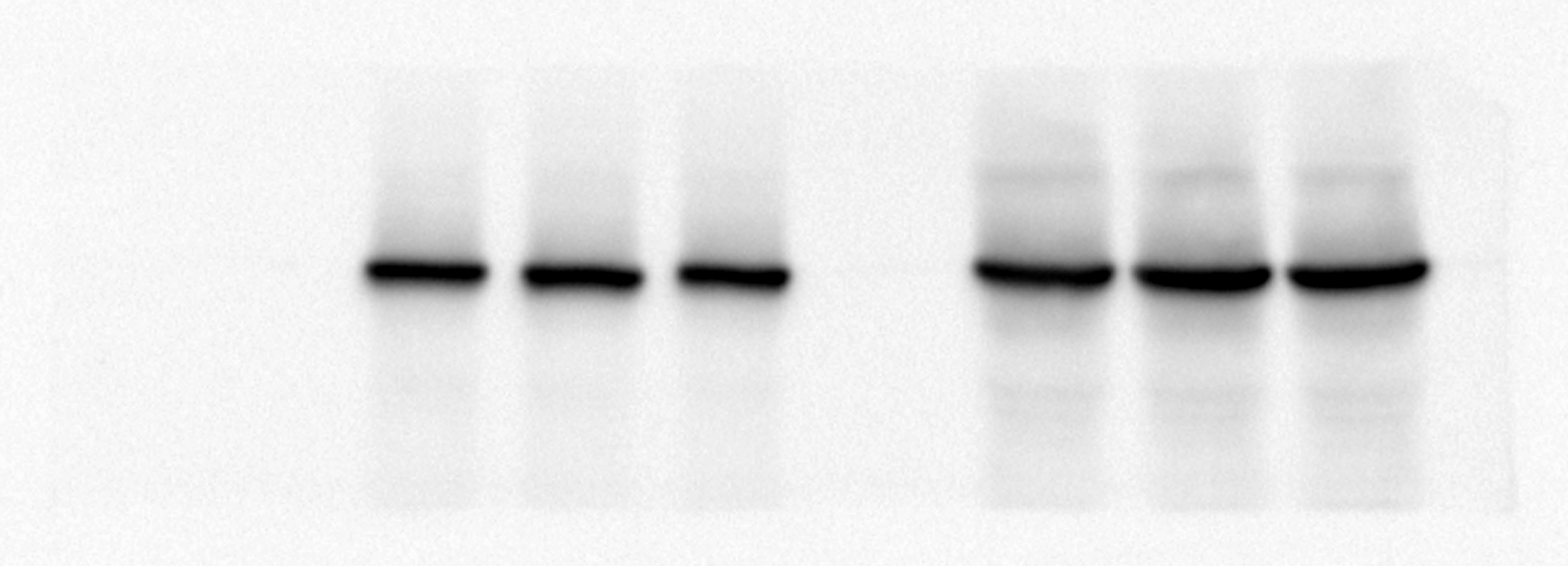

Supplement: Figure 4—source data 3. [file elife-97896-fig4-data3.zip › Figure 4-Source Data 3-28. /Figure 4-Source Data 4. Full raw unedited blot (anti-HA) for Figure 4A.tif]

Figure 4-figure supplement 1A.

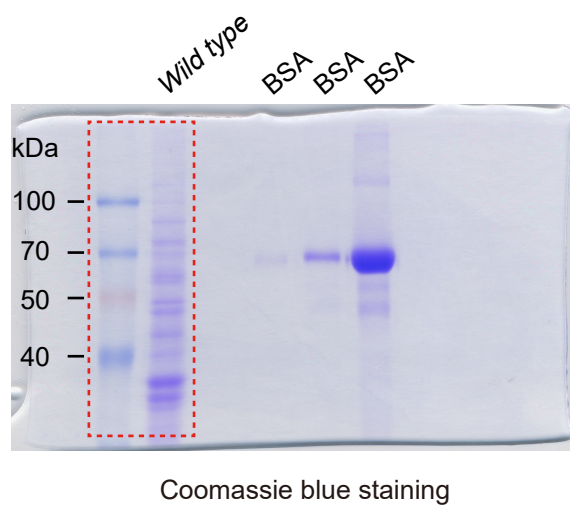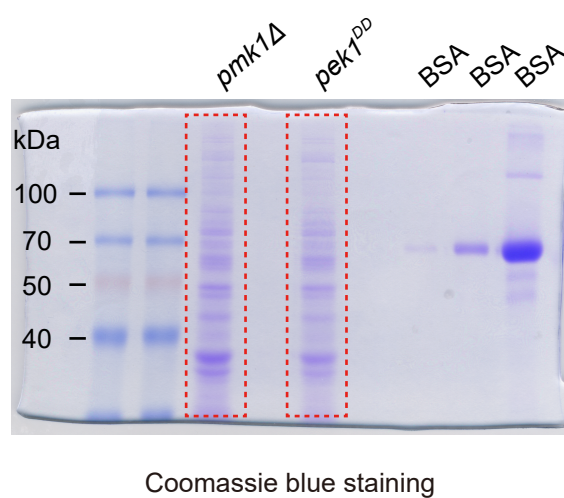

Supplement: Figure 4—figure supplement 1—source data 1. [file elife-97896-fig4-figsupp1-data1.pdf]

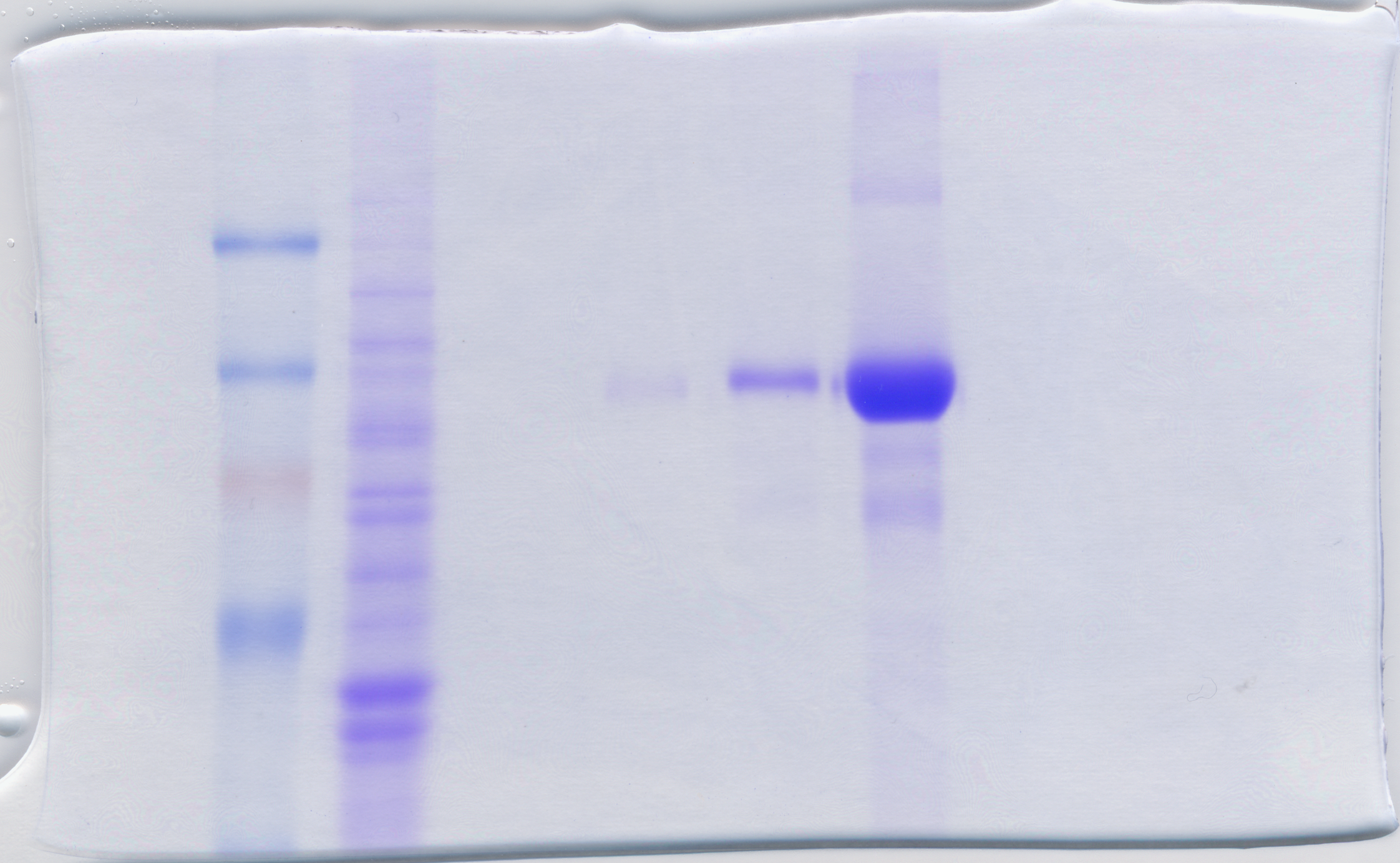

Supplement: Figure 4—figure supplement 1—source data 2. [file elife-97896-fig4-figsupp1-data2.zip › Figure 4-figure supplement 1-Source Data 2. Full raw unedited Coomassie gel #1 for Fig 4-figure suppl 1A.tif]

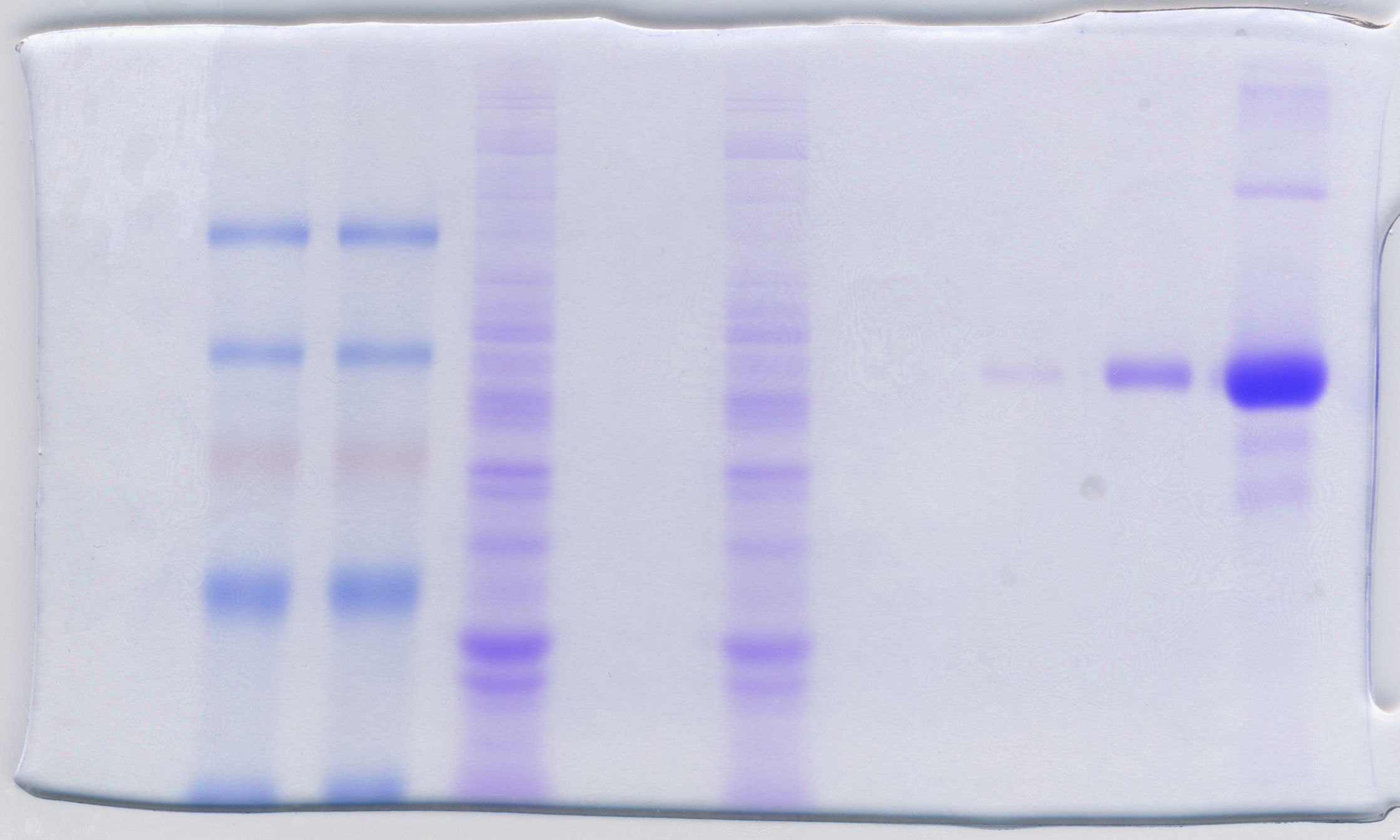

Supplement: Figure 4—figure supplement 1—source data 3. [file elife-97896-fig4-figsupp1-data3.zip › Figure 4-figure supplement 1-Source Data 3. Full raw unedited Coomassie gel #2 for Fig 4-figure suppl 1A.tif]

Figure 4-figure supplement 3C

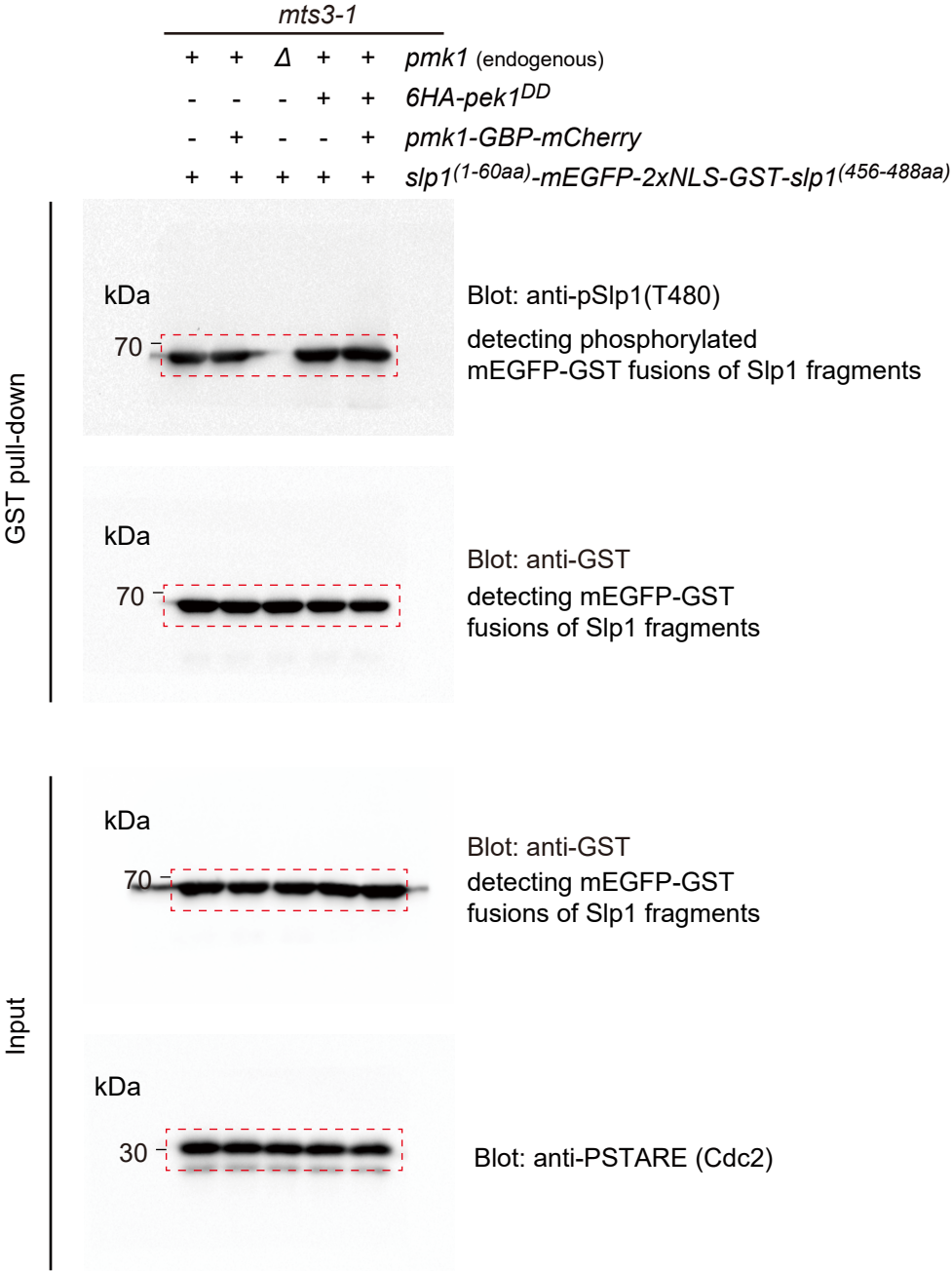

Supplement: Figure 4—figure supplement 3—source data 1. [file elife-97896-fig4-figsupp3-data1.pdf]

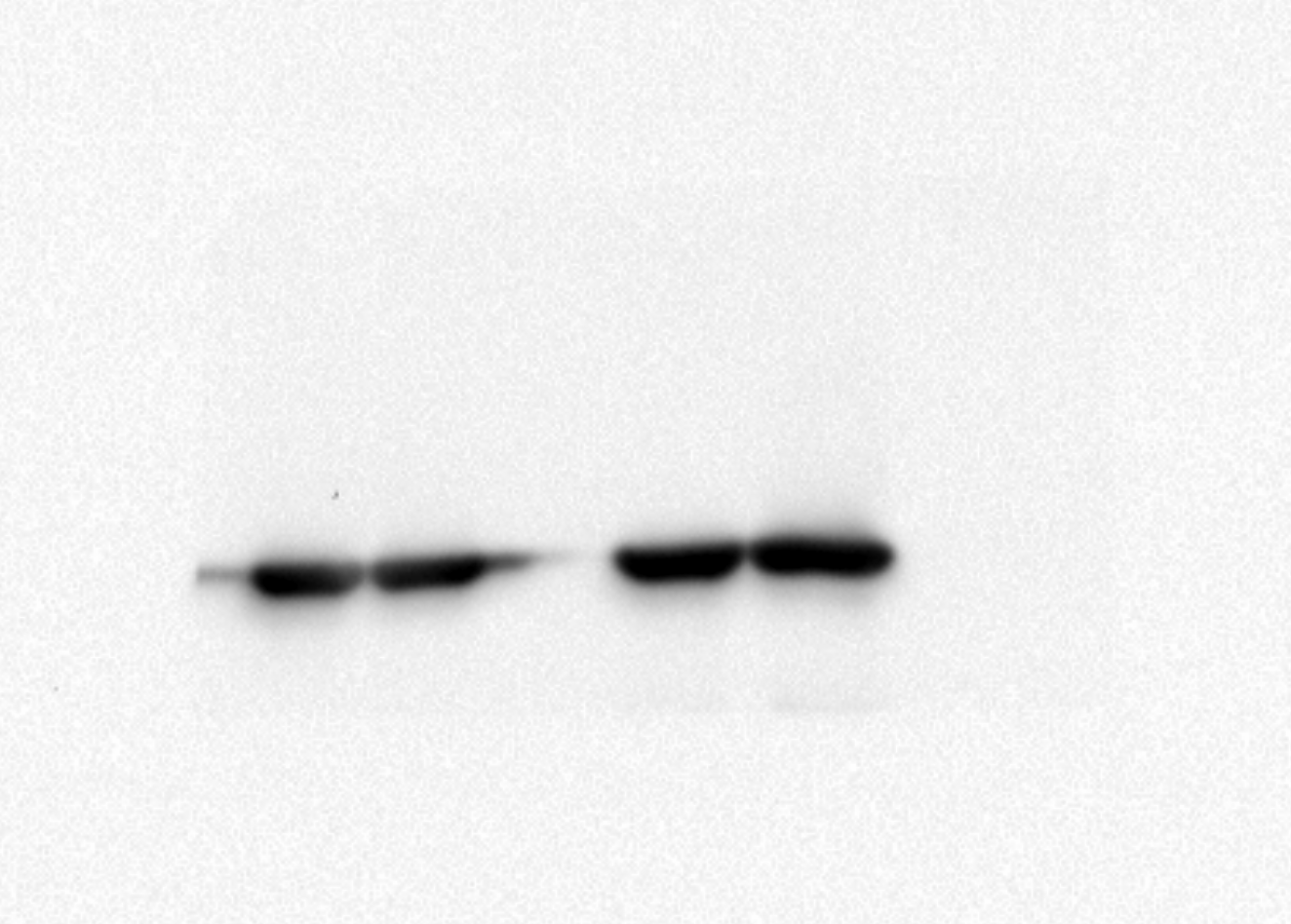

Supplement: Figure 4—figure supplement 3—source data 3. [file elife-97896-fig4-figsupp3-data3.zip › Figure 4-figure supplement 3-Source Data 3. Full raw unedited blot (bead-bound, anti-pT480) for Figure 4-figure supplement 3C.tif]

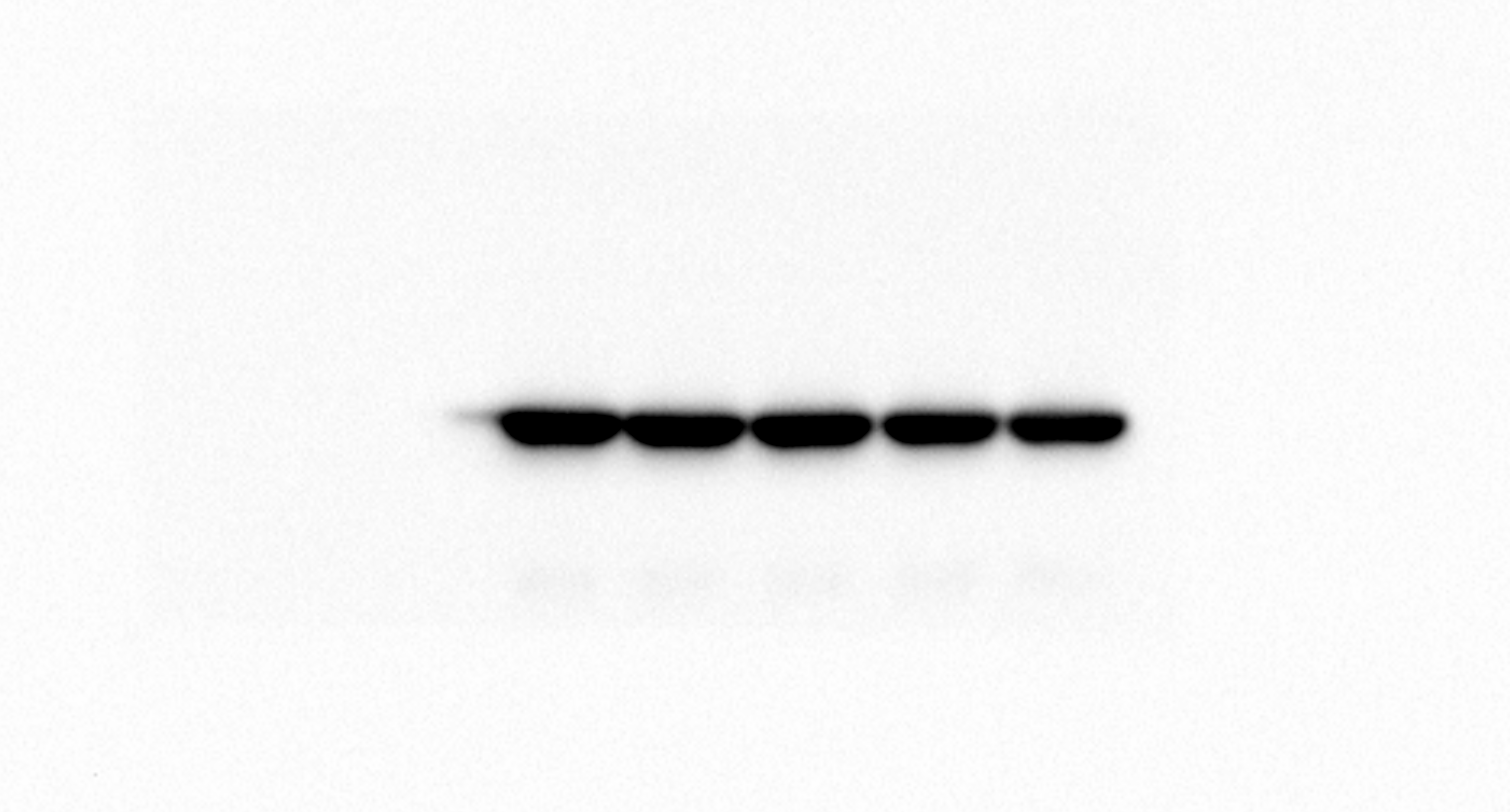

Supplement: Figure 4—figure supplement 3—source data 4. [file elife-97896-fig4-figsupp3-data4.zip › Figure 4-figure supplement 3-Source Data 4. Full raw unedited blot (bead-bound, anti-GST) for Figure 4-figure supplement 3C.tif]

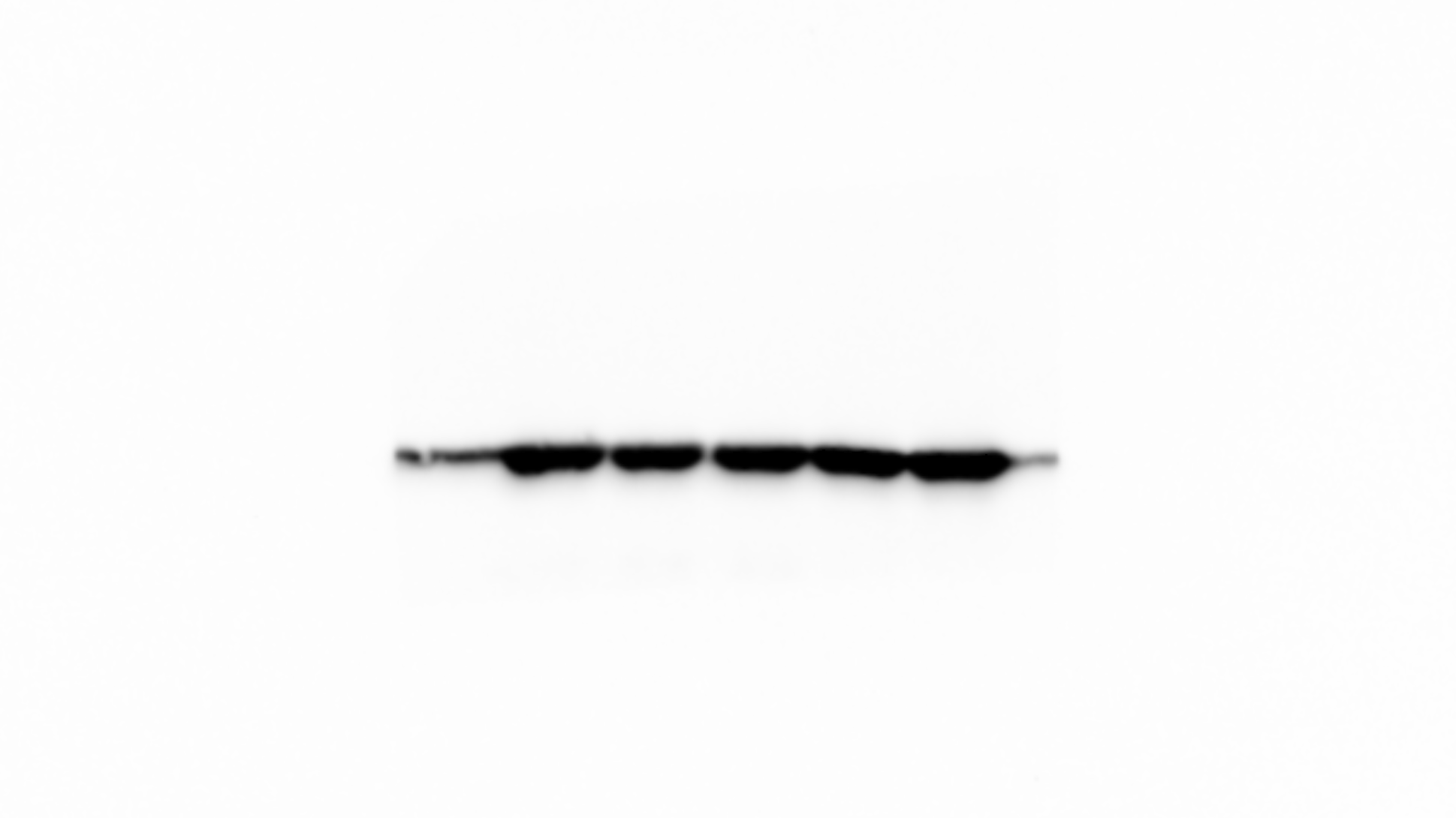

Supplement: Figure 4—figure supplement 3—source data 5. [file elife-97896-fig4-figsupp3-data5.zip › Figure 4-figure supplement 3-Source Data 5. Full raw unedited blot (input, anti-GST) for Figure 4-figure supplement 3C.tif]

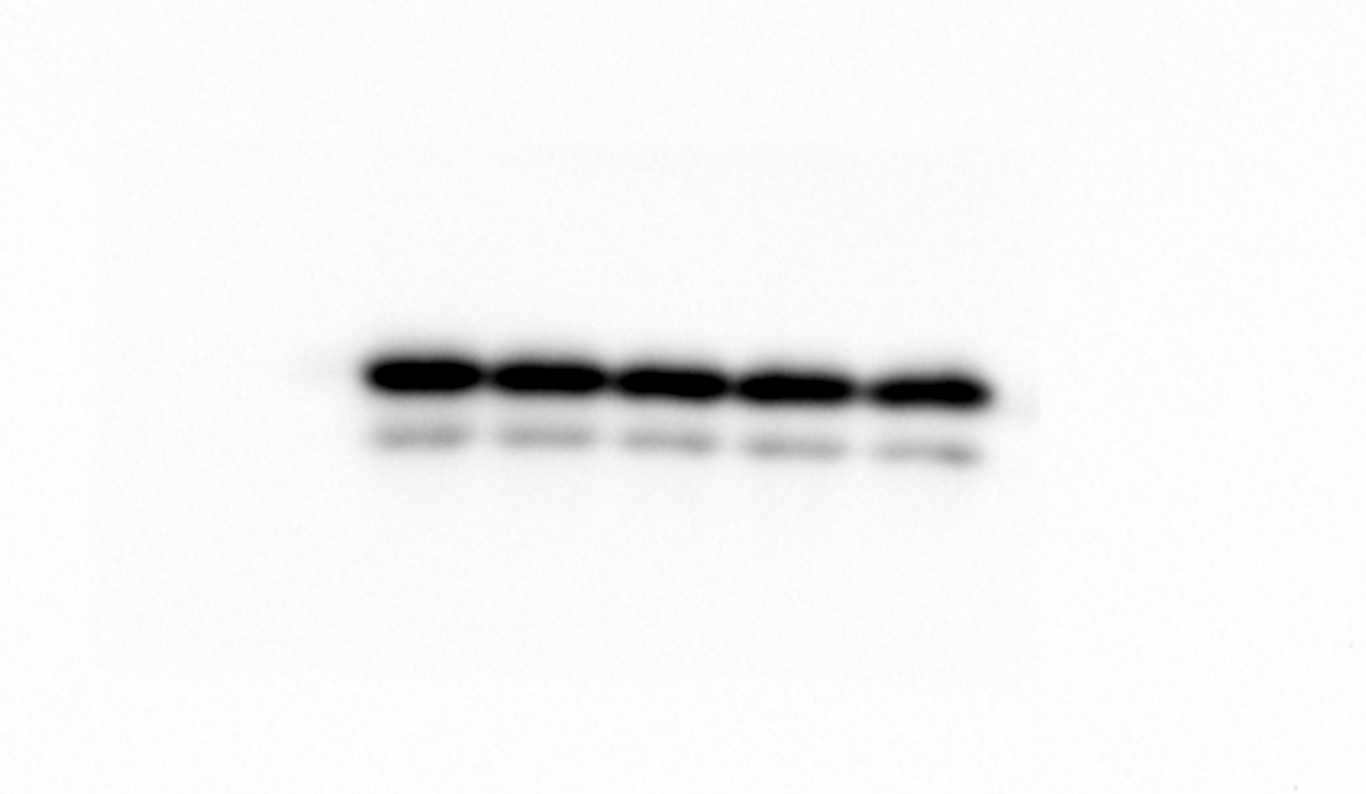

Supplement: Figure 4—figure supplement 3—source data 6. [file elife-97896-fig4-figsupp3-data6.zip › Figure 4-figure supplement 3-Source Data 6. Full raw unedited blot (input, Cdc2) Figure 4-figure supplement 3C.tif]

Figure 4-figure supplement 5.

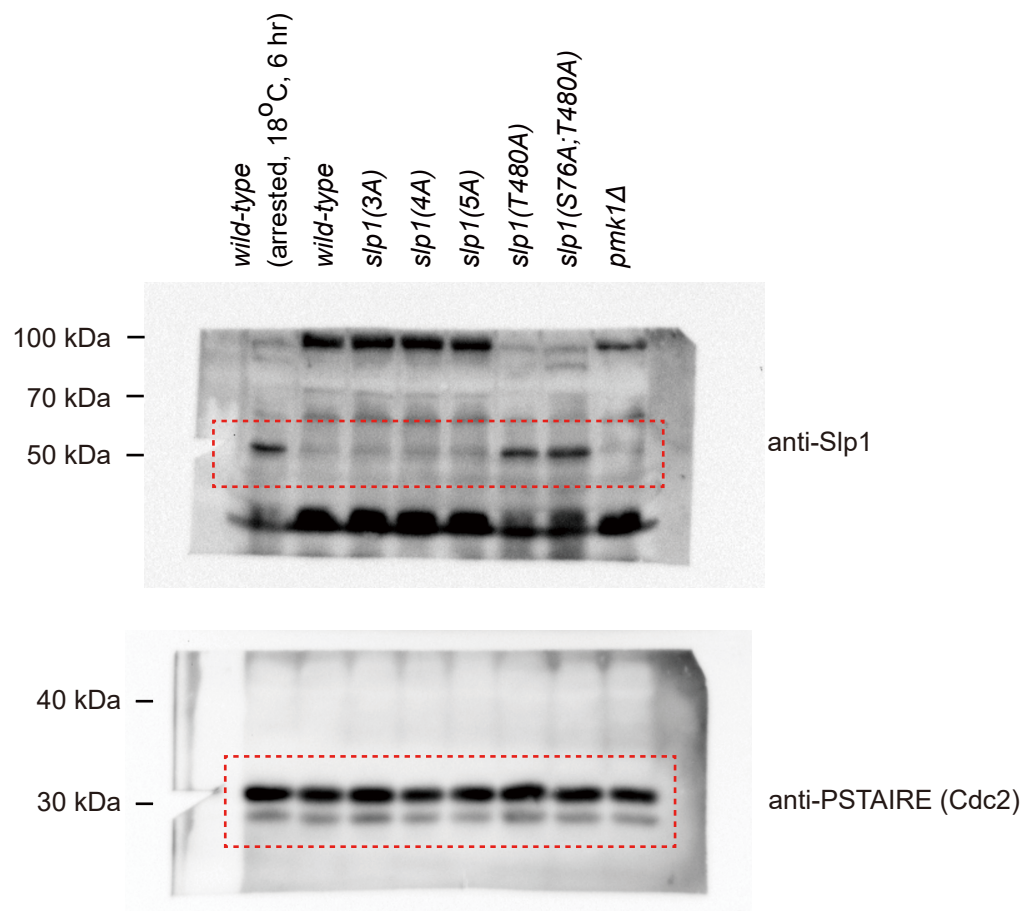

Supplement: Figure 4—figure supplement 5—source data 1. [file elife-97896-fig4-figsupp5-data1.pdf]

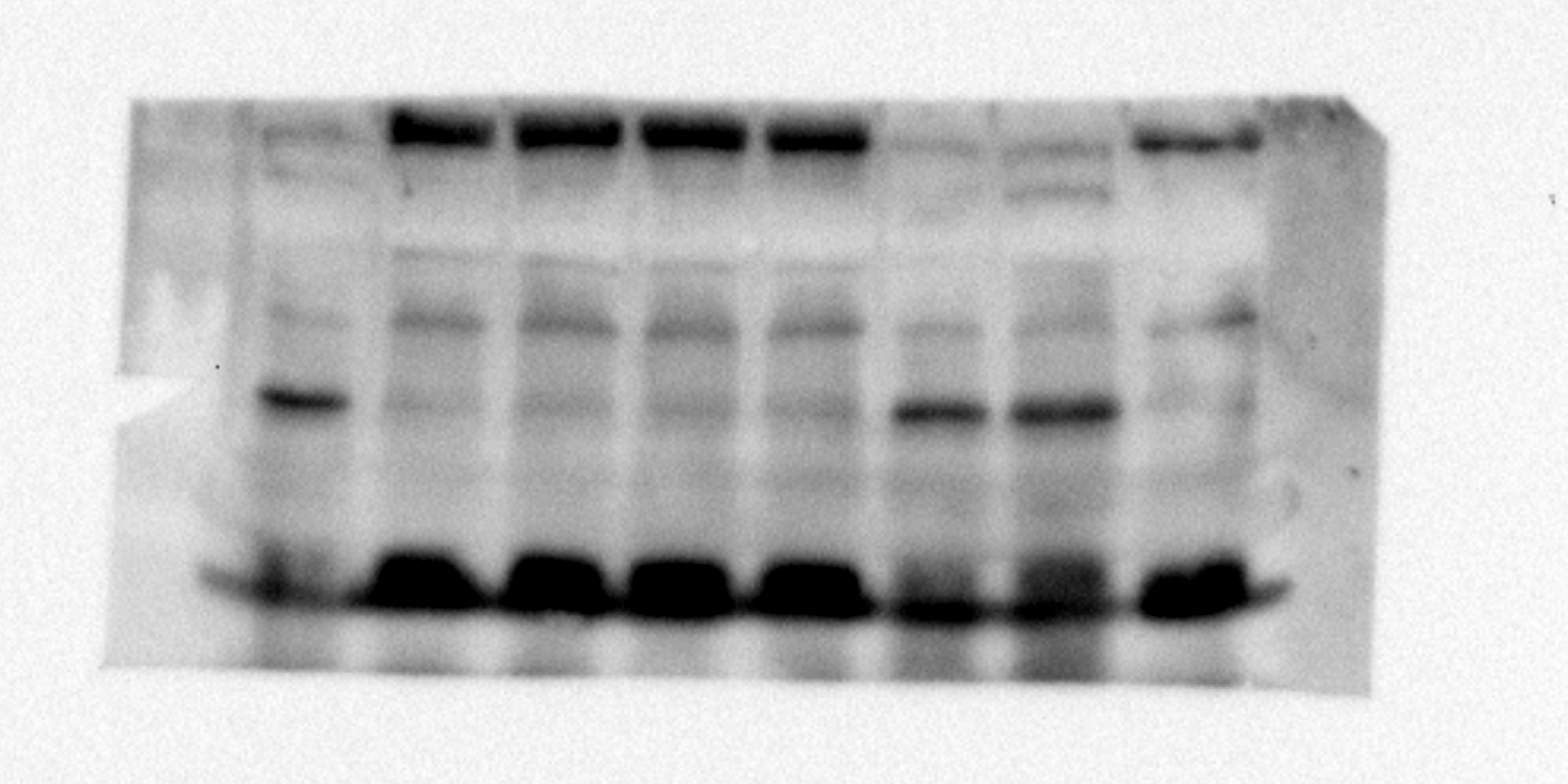

Supplement: Figure 4—figure supplement 5—source data 3. [file elife-97896-fig4-figsupp5-data3.zip › Figure 4-figure supplement 5-Source Data 3. Full raw unedited blot (Slp1) for Figure 4-figure supplement 5.tif]

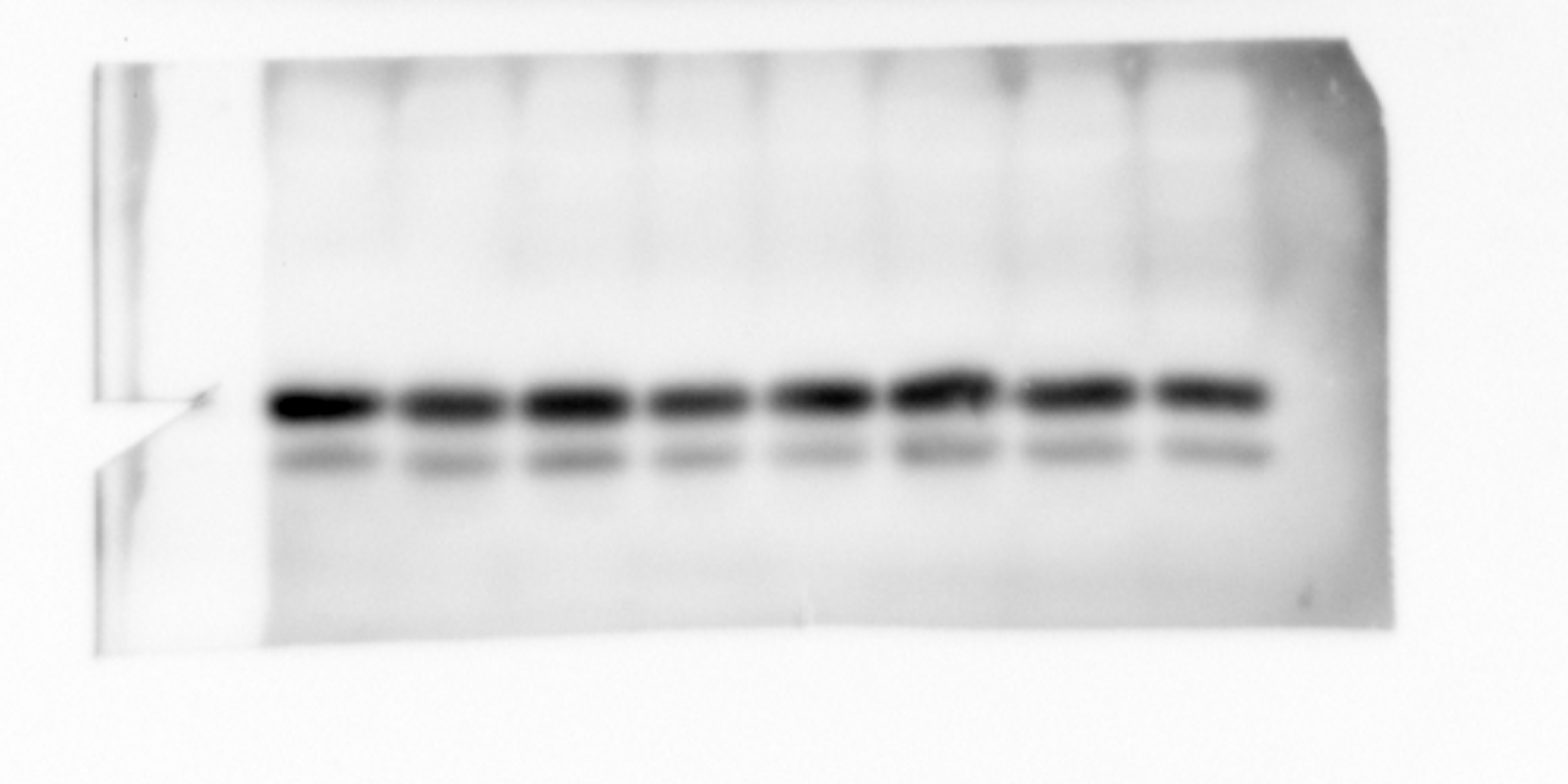

Supplement: Figure 4—figure supplement 5—source data 4. [file elife-97896-fig4-figsupp5-data4.zip › Figure 4-figure supplement 5-Source Data 4. Full raw unedited blot (Cdc2) for Figure 4-figure supplement 5.tif]

Figure 5A.

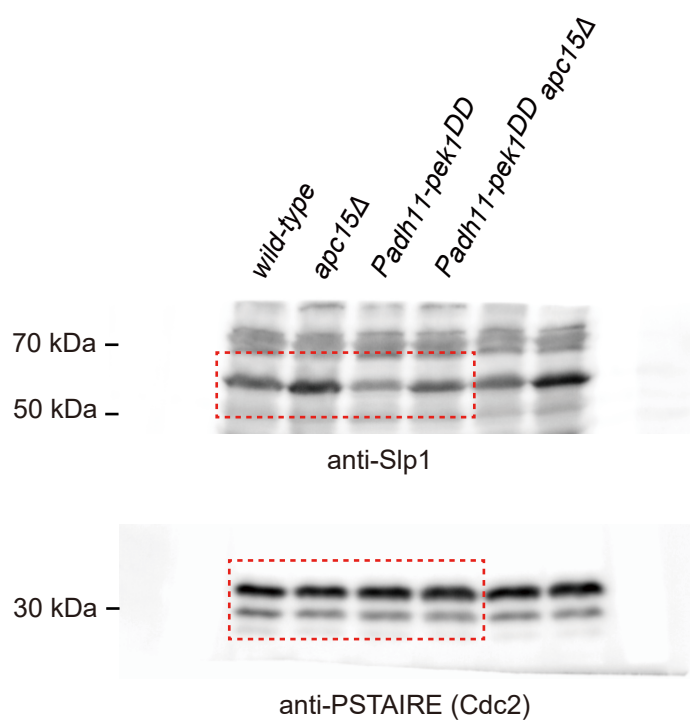

Figure 5C.

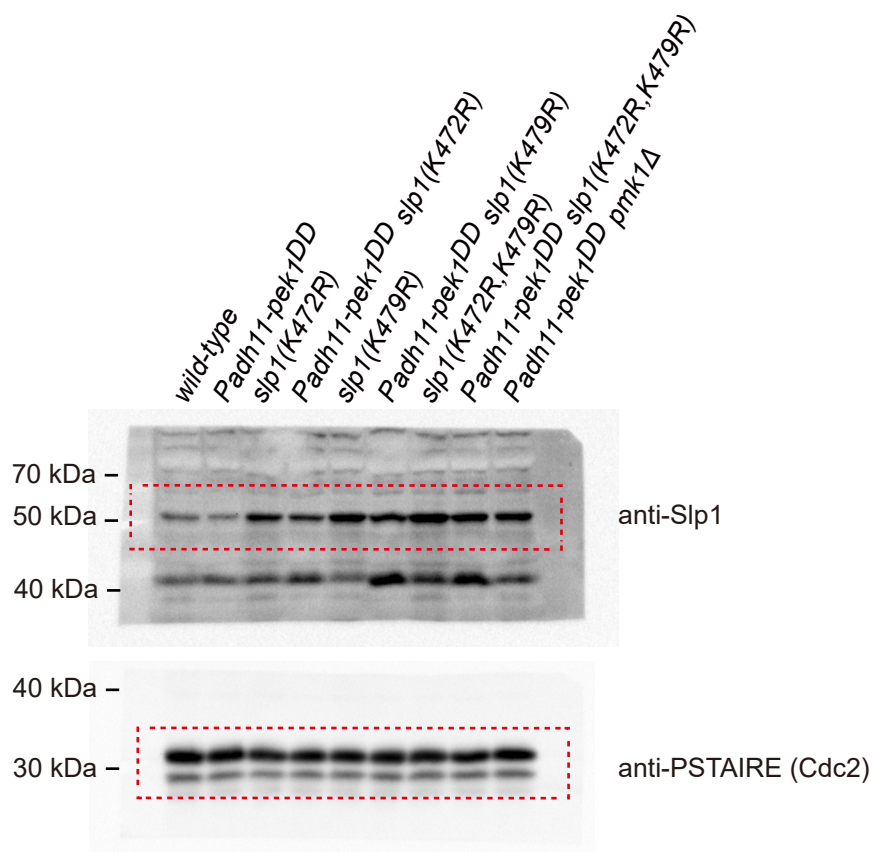

Figure 5F.

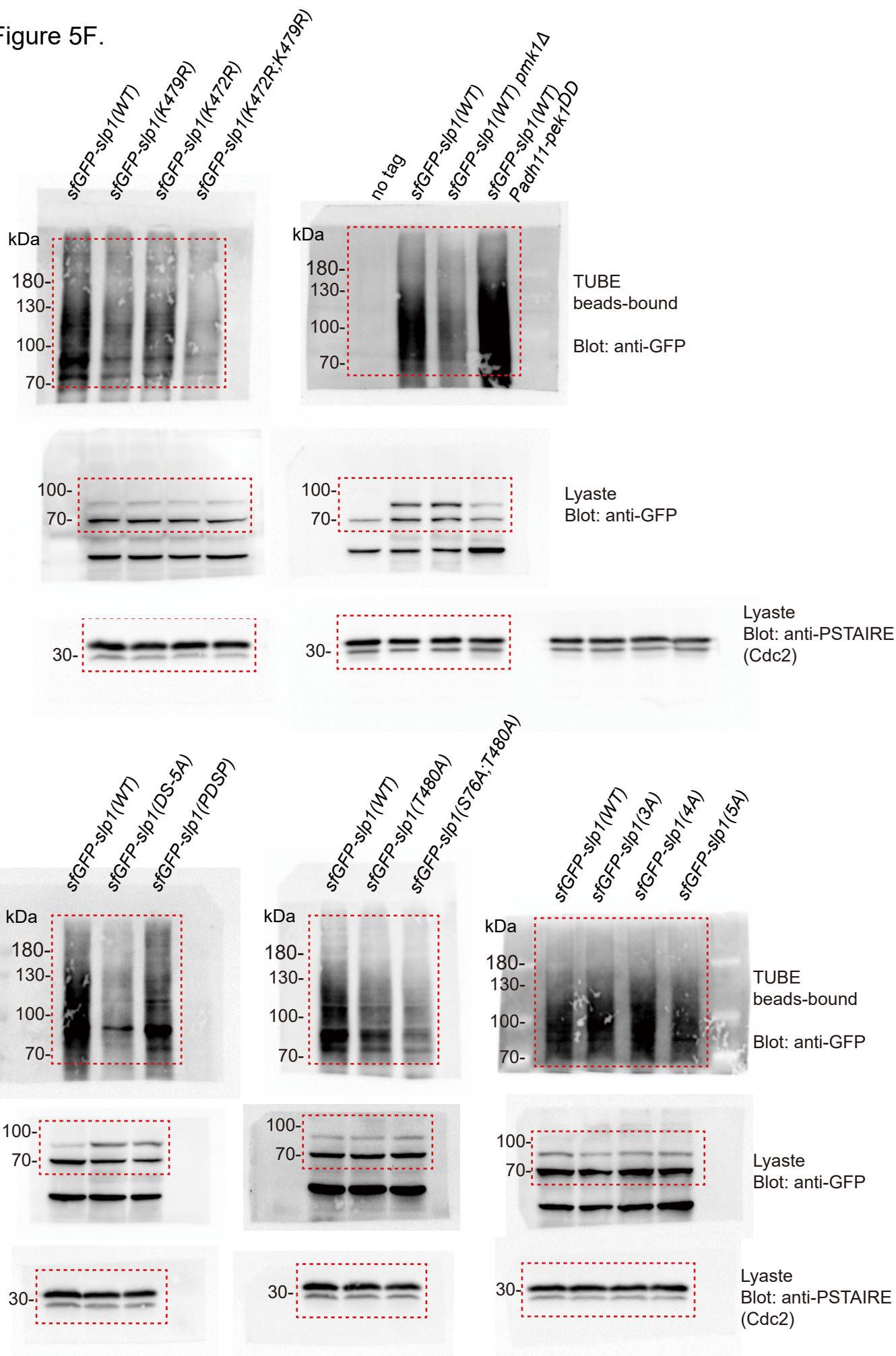

Supplement: Figure 5—source data 1. [file elife-97896-fig5-data1.pdf]

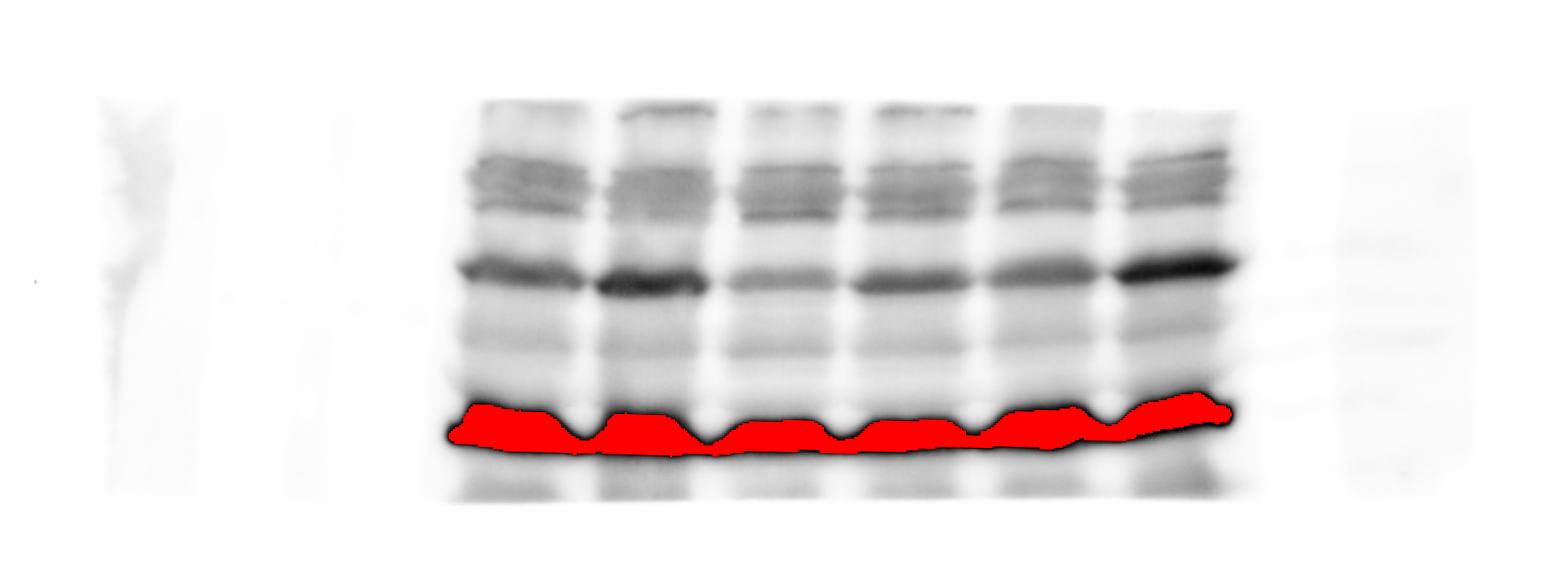

Supplement: Figure 5—source data 3. [file elife-97896-fig5-data3.zip › Figure 5-Source Data 3-21. /Figure 5-Source Data 3. Full raw unedited blot (Slp1) for Figure 5A.tif]

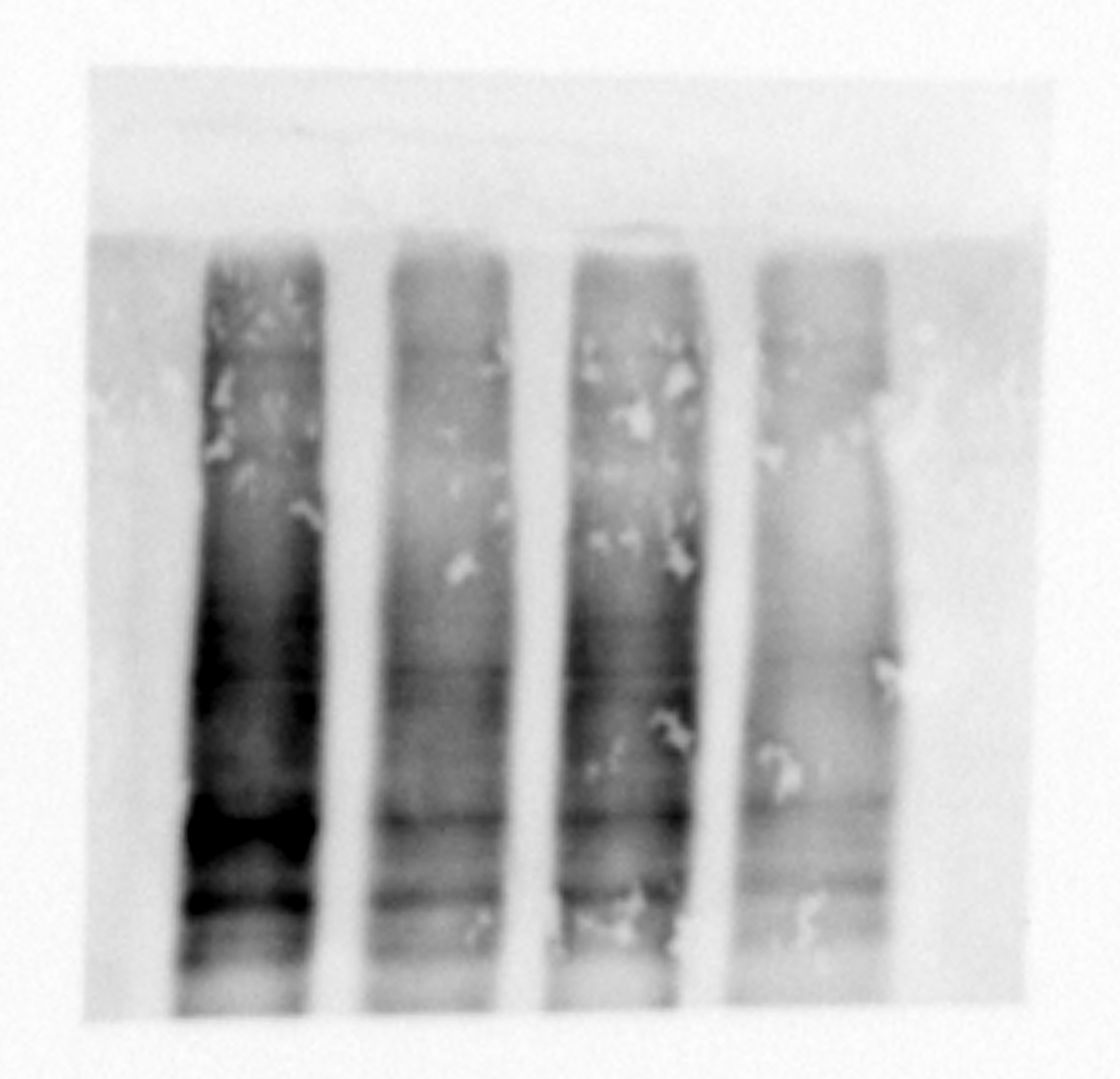

Supplement: Figure 5—source data 3. [file elife-97896-fig5-data3.zip › Figure 5-Source Data 3-21. /Figure 5-Source Data 7. Full raw unedited blot (bead-bound sfGFP-Slp1, blot 1) for Figure 5F.tif]

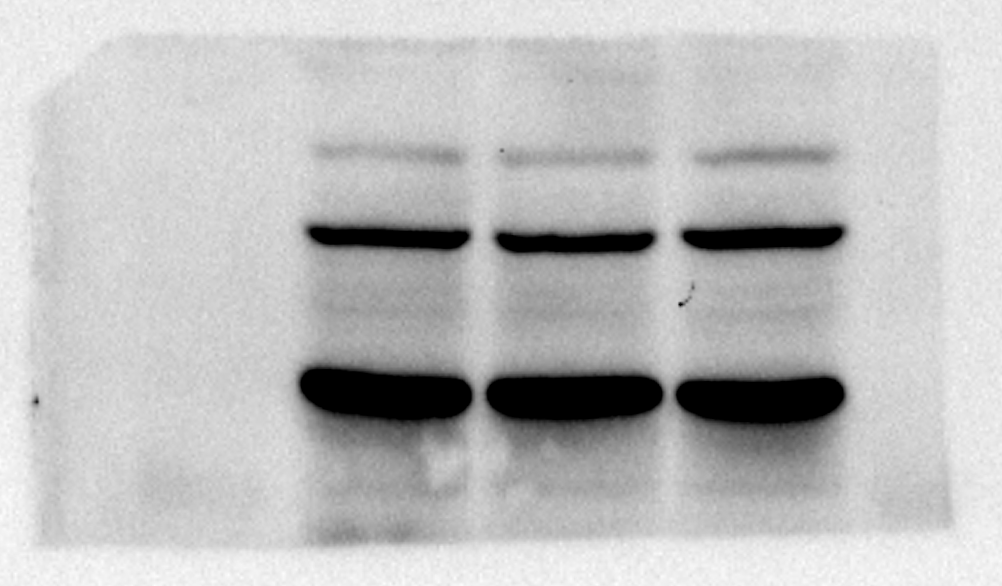

Supplement: Figure 5—source data 3. [file elife-97896-fig5-data3.zip › Figure 5-Source Data 3-21. /Figure 5-Source Data 15. Full raw unedited blot (sfGFP-Slp1 input, blot 4) for Figure 5F.tif]

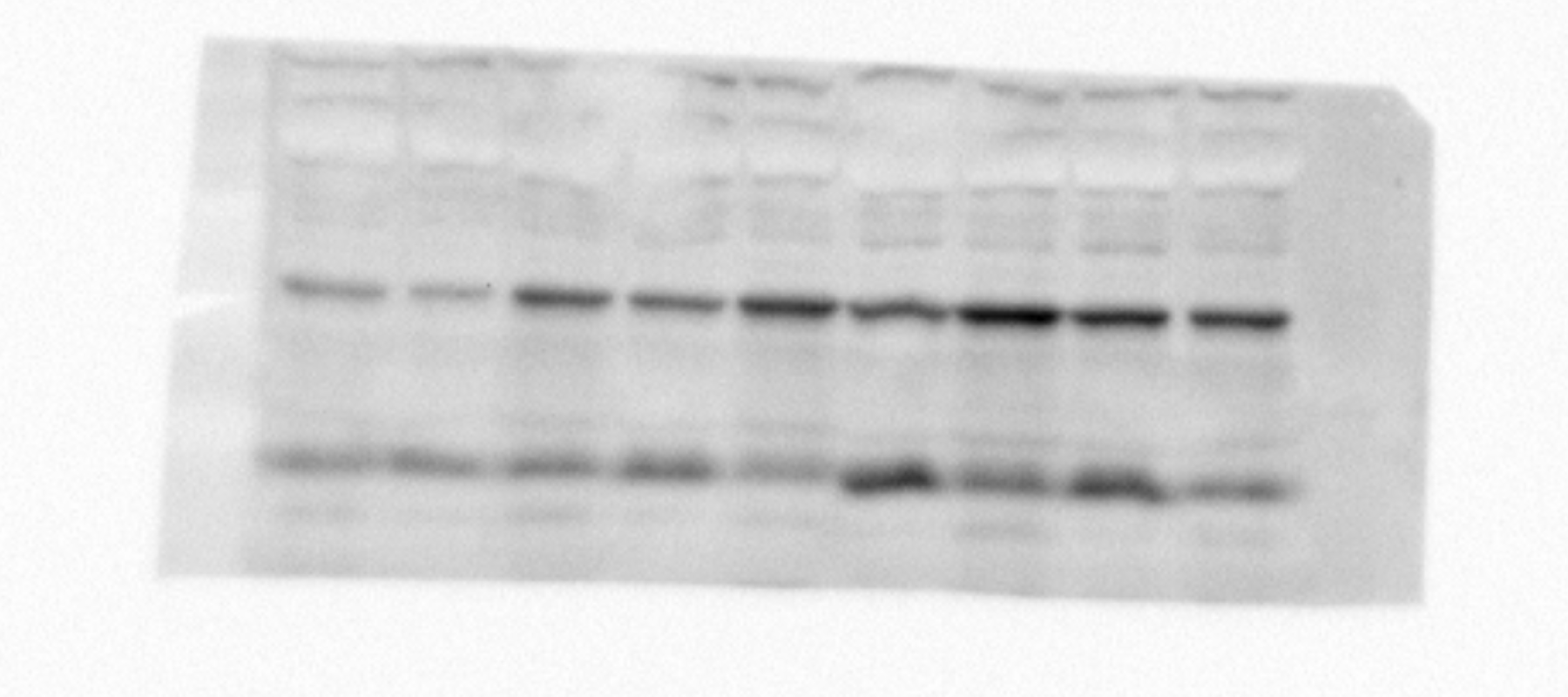

Supplement: Figure 5—source data 3. [file elife-97896-fig5-data3.zip › Figure 5-Source Data 3-21. /Figure 5-Source Data 5. Full raw unedited blot (Slp1) for Figure 5C.tif]

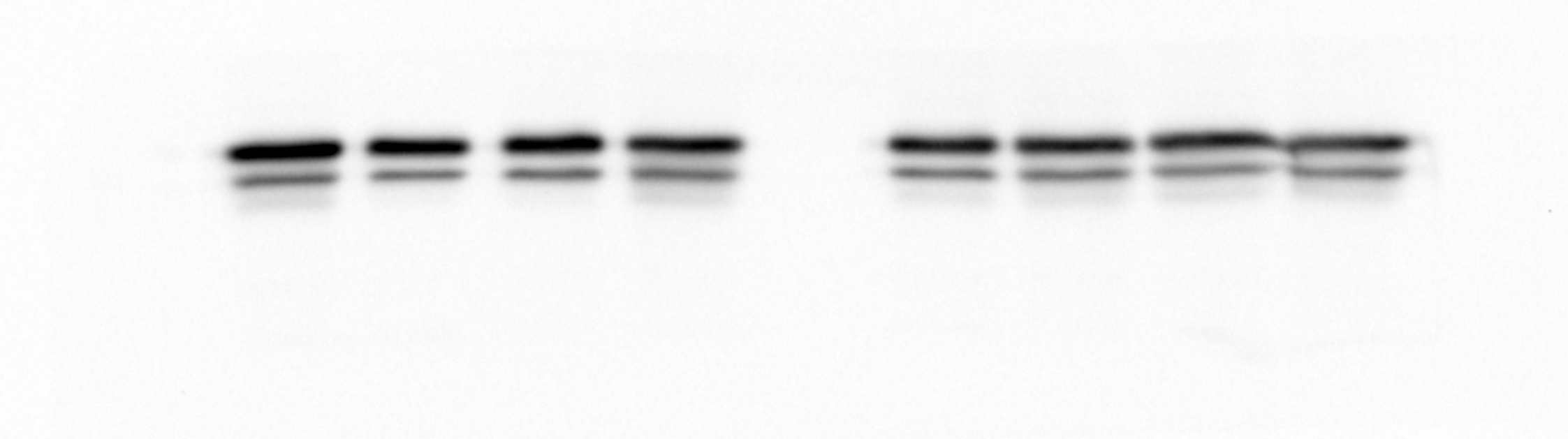

Supplement: Figure 5—source data 3. [file elife-97896-fig5-data3.zip › Figure 5-Source Data 3-21. /Figure 5-Source Data 18. Full raw unedited blot (Cdc2 input, blot 2) for Figure 5F.tif]

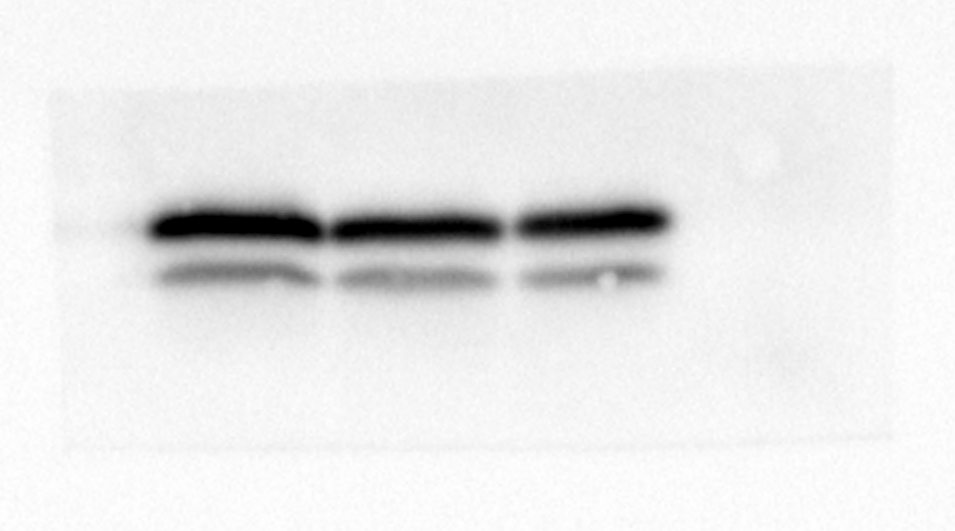

Supplement: Figure 5—source data 3. [file elife-97896-fig5-data3.zip › Figure 5-Source Data 3-21. /Figure 5-Source Data 19. Full raw unedited blot (Cdc2 input, blot 3) for Figure 5F.tif]

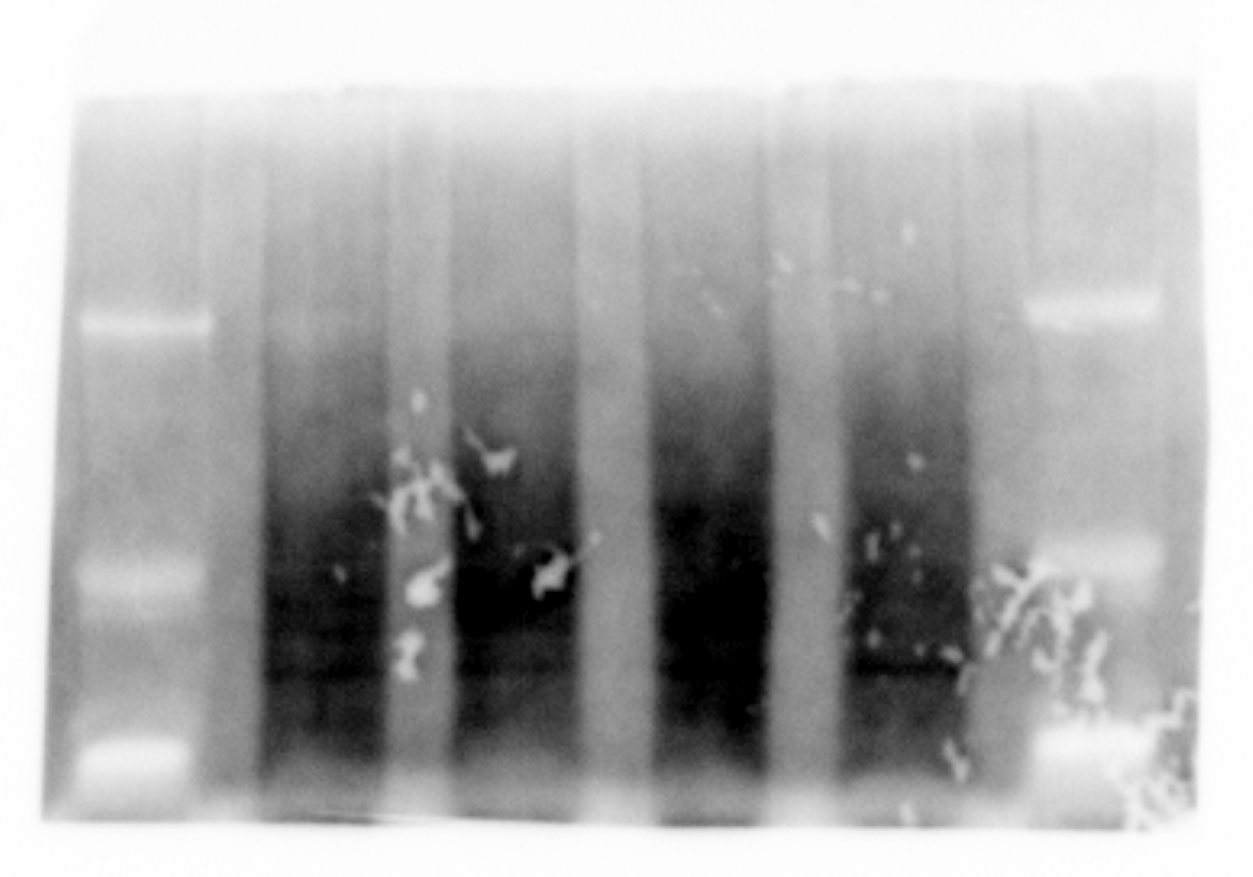

Supplement: Figure 5—source data 3. [file elife-97896-fig5-data3.zip › Figure 5-Source Data 3-21. /Figure 5-Source Data 11. Full raw unedited blot (bead-bound sfGFP-Slp1, blot 5) for Figure 5F.tif]

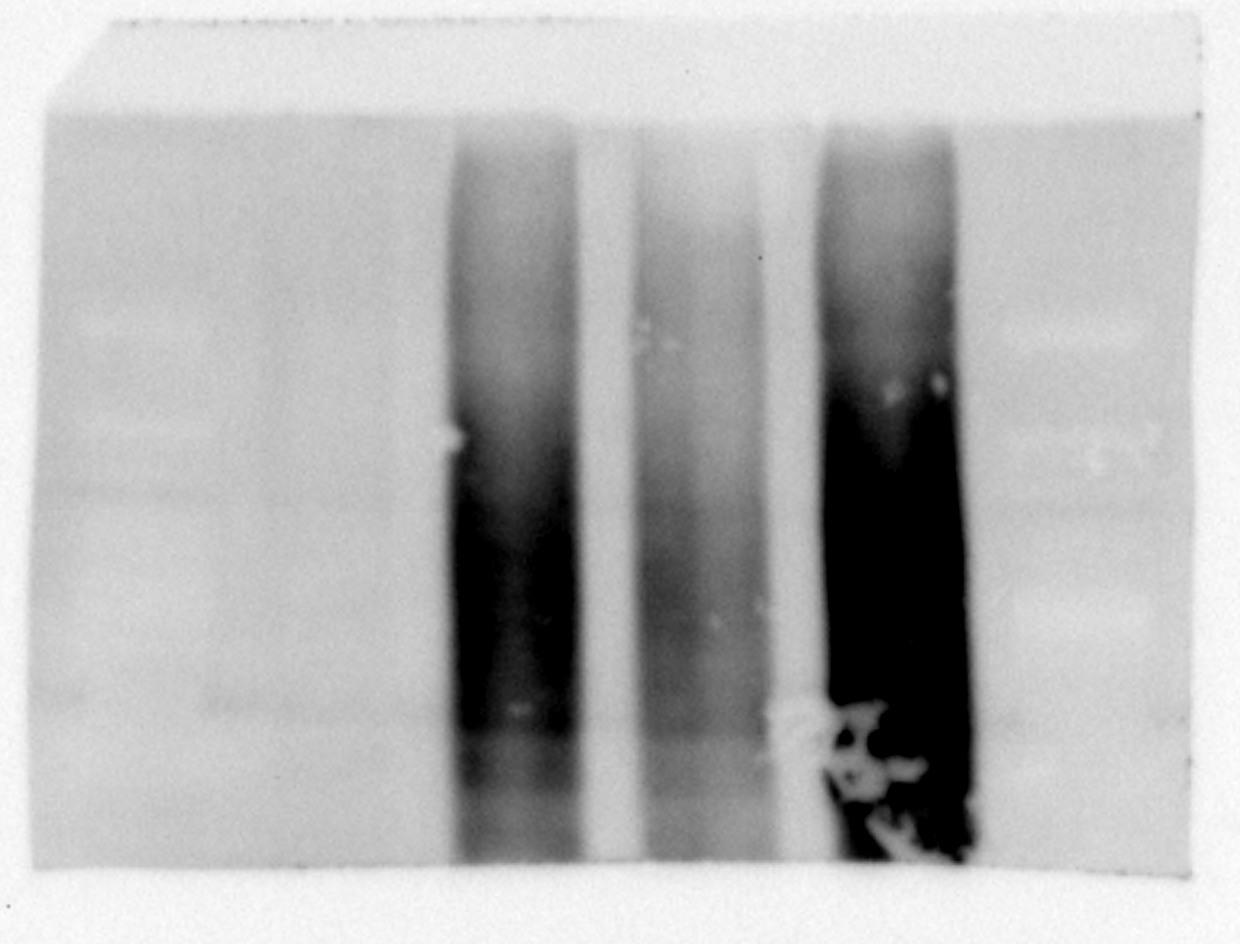

Supplement: Figure 5—source data 3. [file elife-97896-fig5-data3.zip › Figure 5-Source Data 3-21. /Figure 5-Source Data 8. Full raw unedited blot (bead-bound sfGFP-Slp1, blot 2) for Figure 5F.tif]

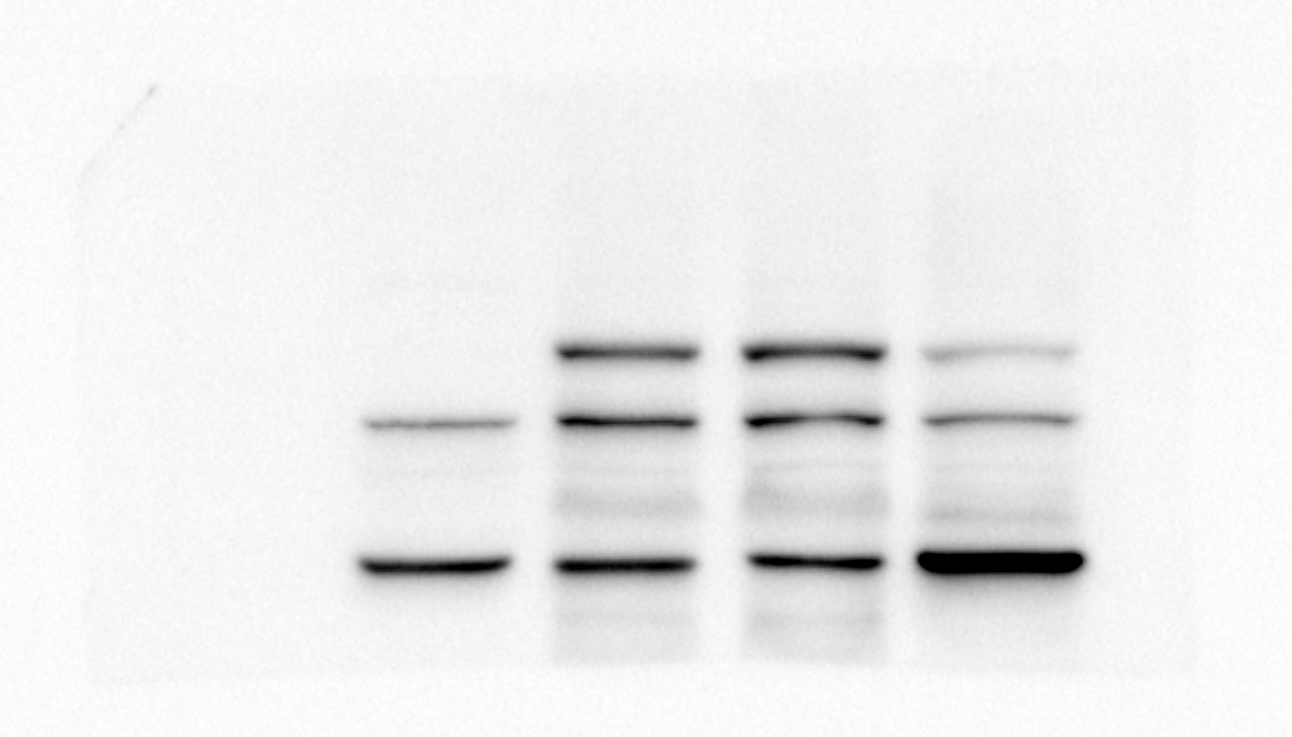

Supplement: Figure 5—source data 3. [file elife-97896-fig5-data3.zip › Figure 5-Source Data 3-21. /Figure 5-Source Data 13. Full raw unedited blot (sfGFP-Slp1 input, blot 2) for Figure 5F.tif]

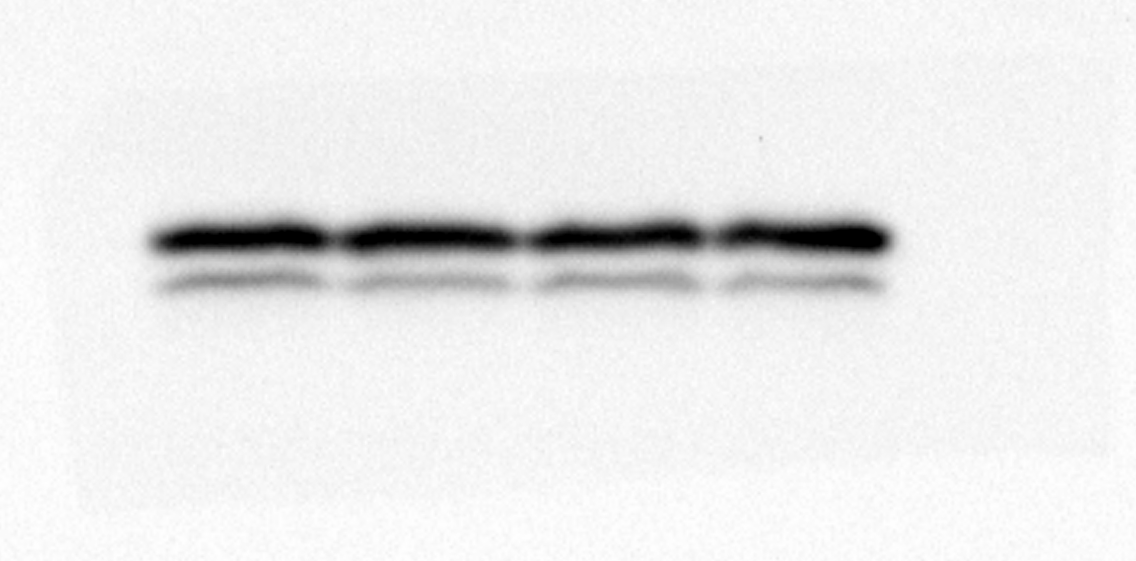

Supplement: Figure 5—source data 3. [file elife-97896-fig5-data3.zip › Figure 5-Source Data 3-21. /Figure 5-Source Data 21. Full raw unedited blot (Cdc2 input, blot 5) for Figure 5F.tif]

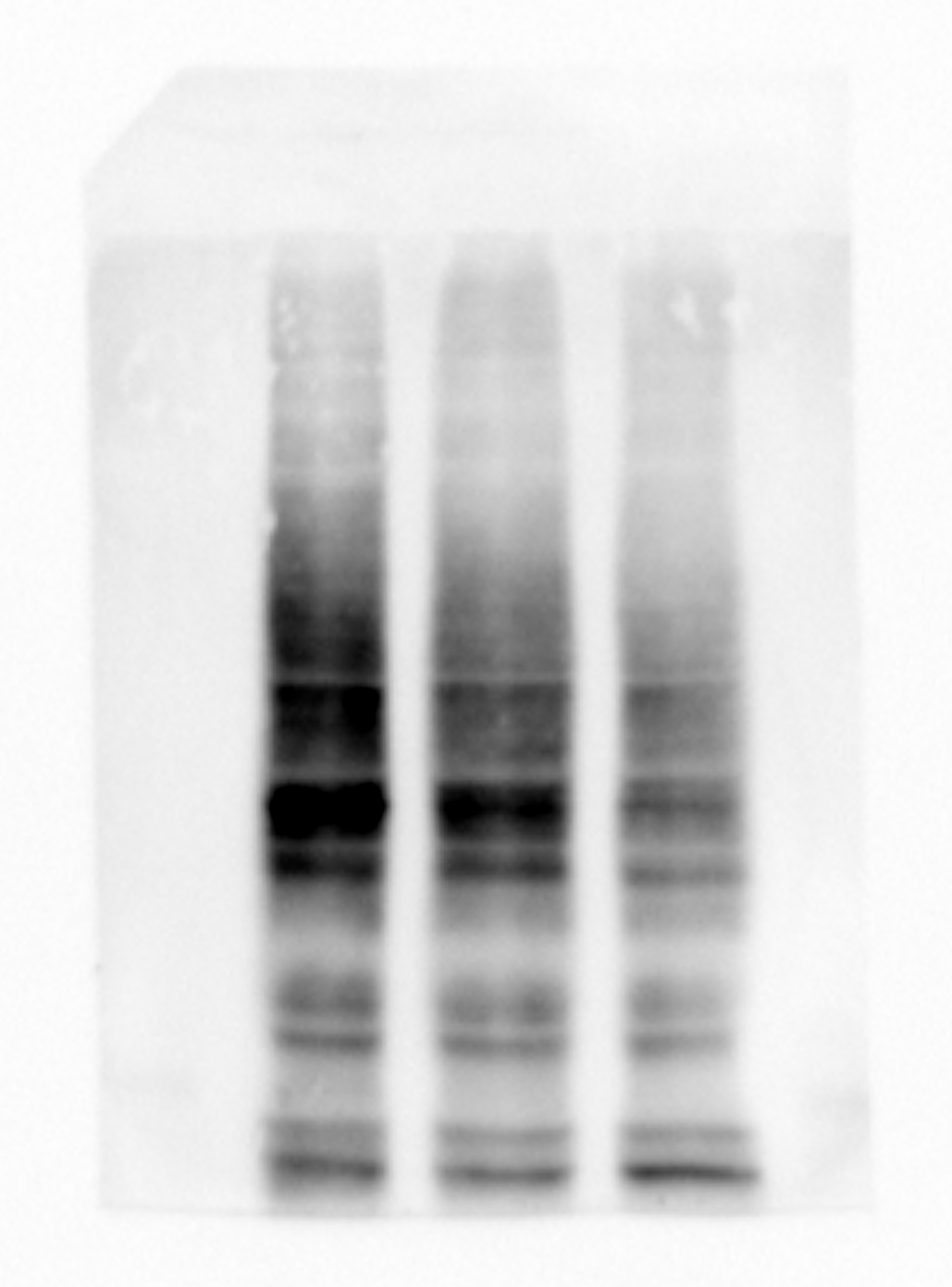

Supplement: Figure 5—source data 3. [file elife-97896-fig5-data3.zip › Figure 5-Source Data 3-21. /Figure 5-Source Data 10. Full raw unedited blot (bead-bound sfGFP-Slp1, blot 4) for Figure 5F.tif]

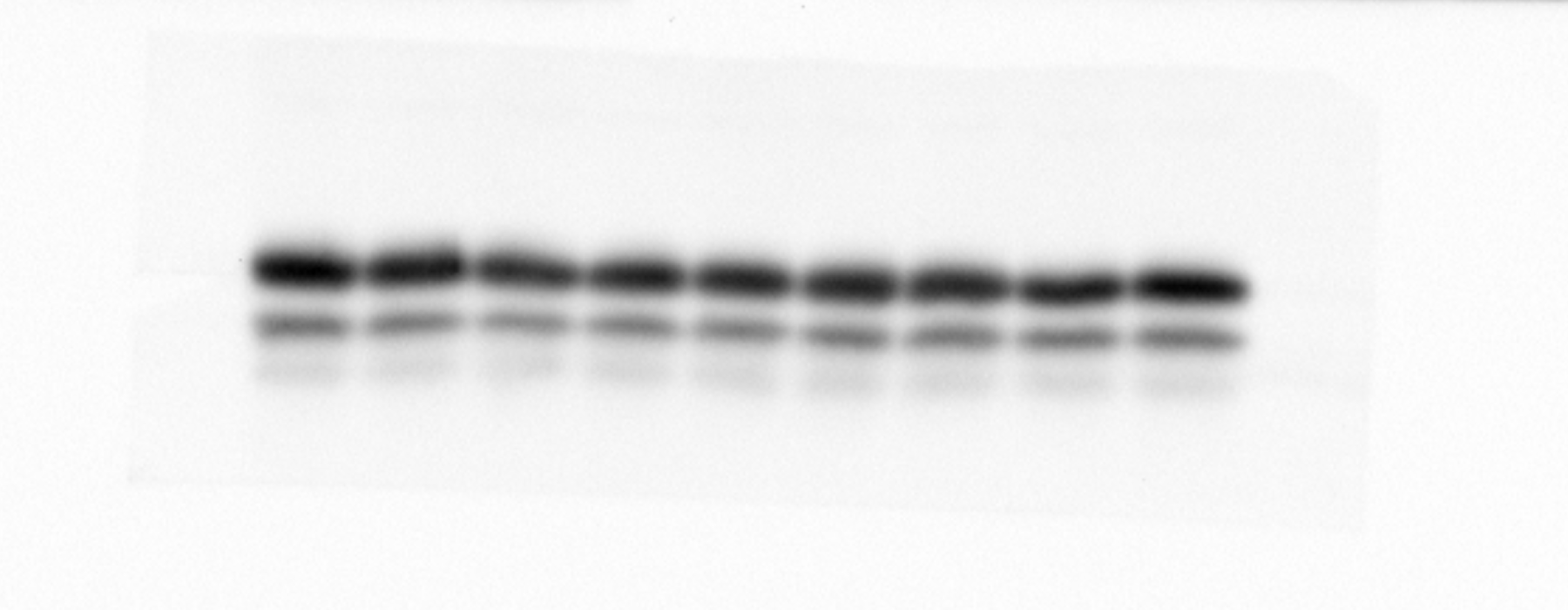

Supplement: Figure 5—source data 3. [file elife-97896-fig5-data3.zip › Figure 5-Source Data 3-21. /Figure 5-Source Data 6. Full raw unedited blot (Cdc2) for Figure 5C.tif]

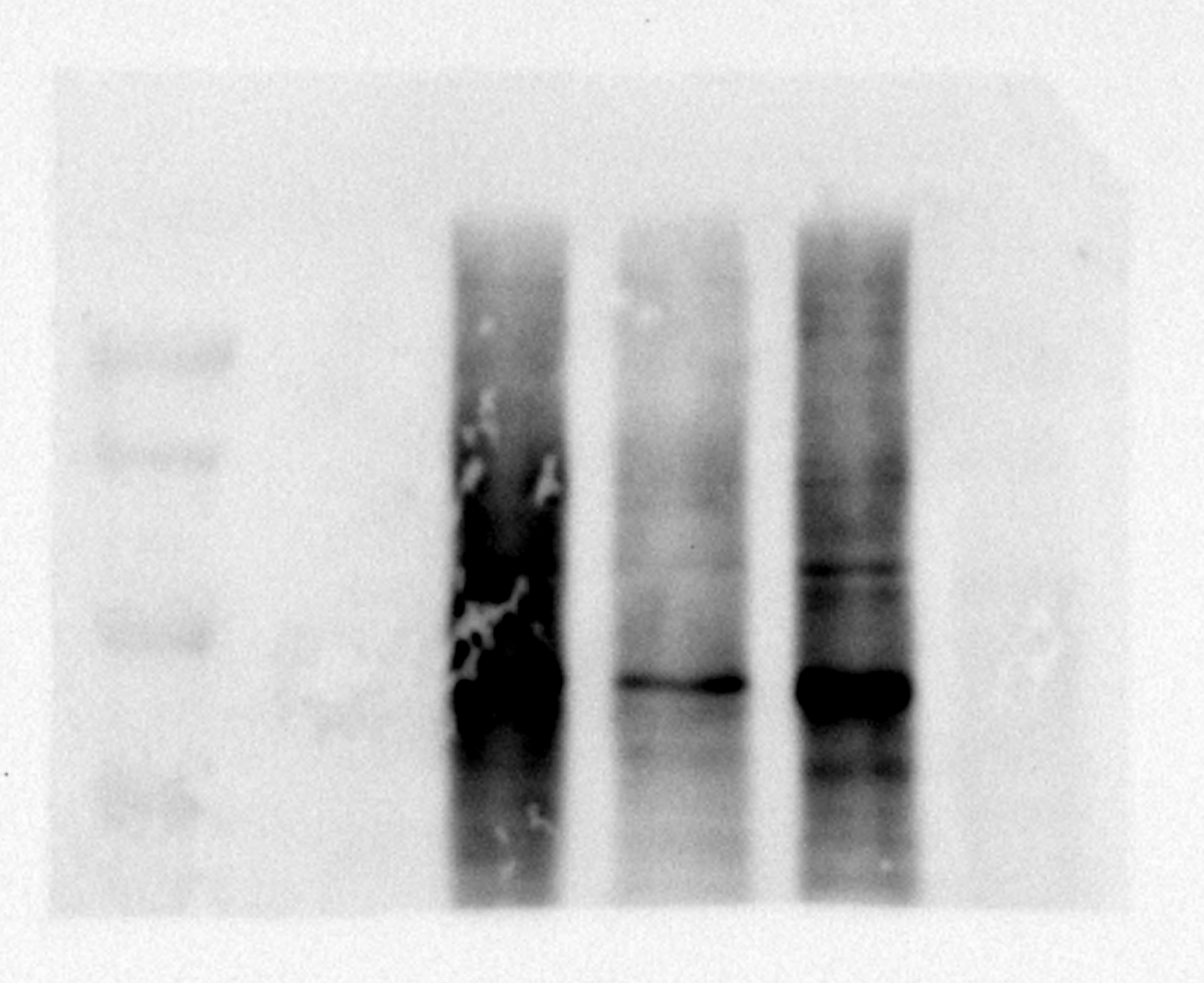

Supplement: Figure 5—source data 3. [file elife-97896-fig5-data3.zip › Figure 5-Source Data 3-21. /Figure 5-Source Data 9. Full raw unedited blot (bead-bound sfGFP-Slp1, blot 3) for Figure 5F.tif]

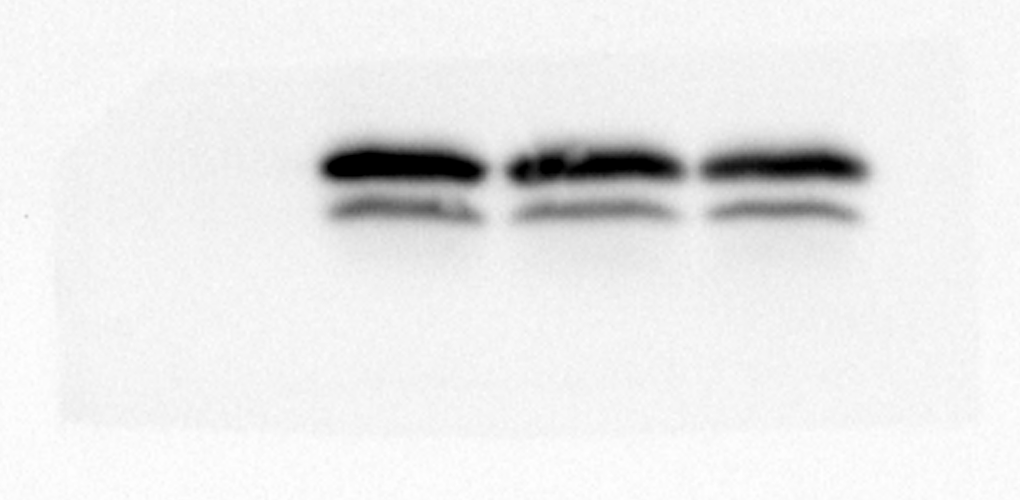

Supplement: Figure 5—source data 3. [file elife-97896-fig5-data3.zip › Figure 5-Source Data 3-21. /Figure 5-Source Data 20. Full raw unedited blot (Cdc2 input, blot 4) for Figure 5F.tif]

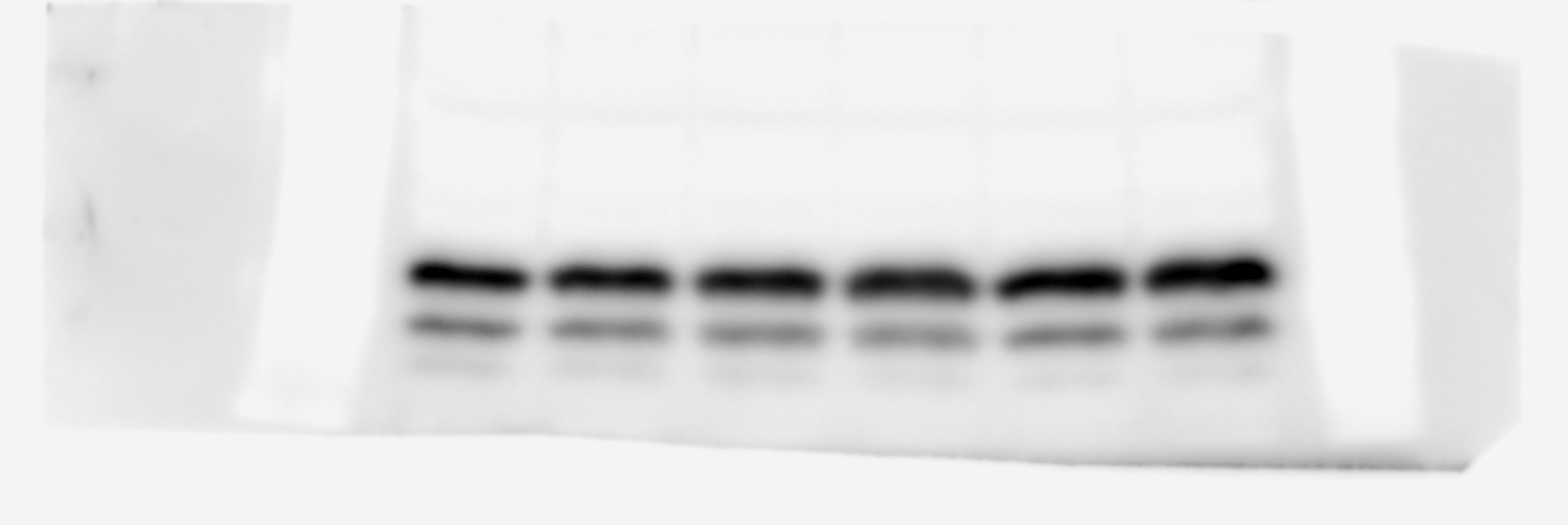

Supplement: Figure 5—source data 3. [file elife-97896-fig5-data3.zip › Figure 5-Source Data 3-21. /Figure 5-Source Data 4. Full raw unedited blot (Cdc2) for Figure 5A.tif]

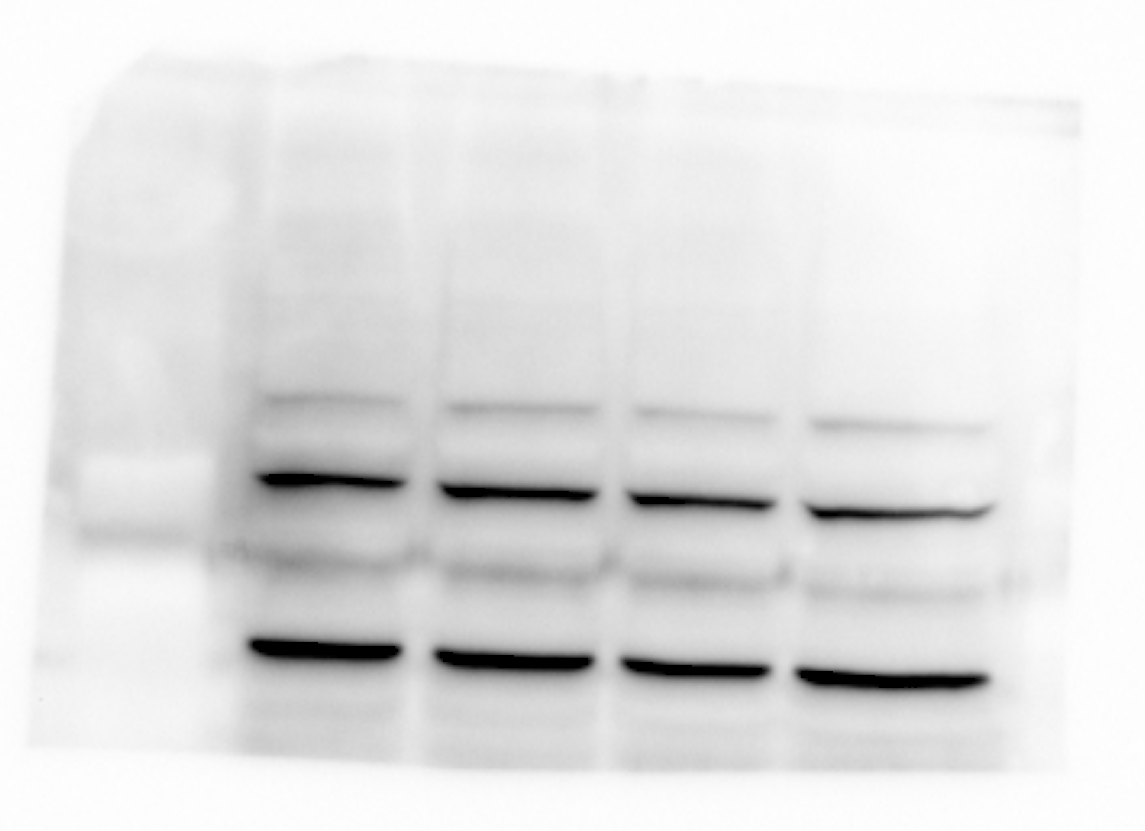

Supplement: Figure 5—source data 3. [file elife-97896-fig5-data3.zip › Figure 5-Source Data 3-21. /Figure 5-Source Data 12. Full raw unedited blot (sfGFP-Slp1 input, blot 1) for Figure 5F.tif]

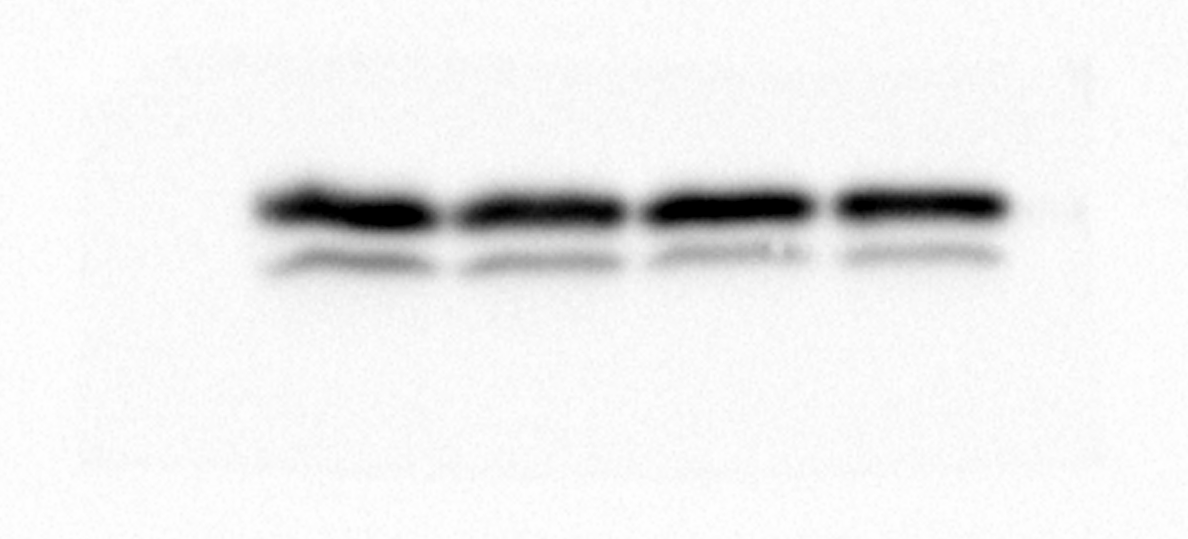

Supplement: Figure 5—source data 3. [file elife-97896-fig5-data3.zip › Figure 5-Source Data 3-21. /Figure 5-Source Data 17. Full raw unedited blot (Cdc2 input, blot 1) for Figure 5F.tif]

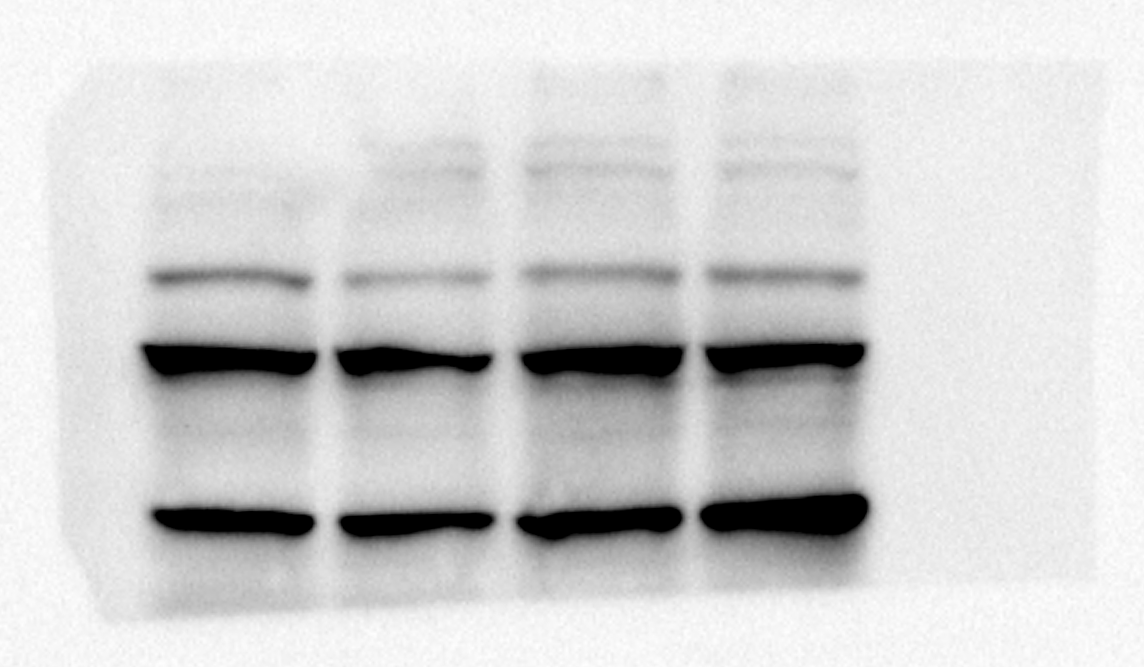

Supplement: Figure 5—source data 3. [file elife-97896-fig5-data3.zip › Figure 5-Source Data 3-21. /Figure 5-Source Data 16. Full raw unedited blot (sfGFP-Slp1 input, blot 5) for Figure 5F.tif]

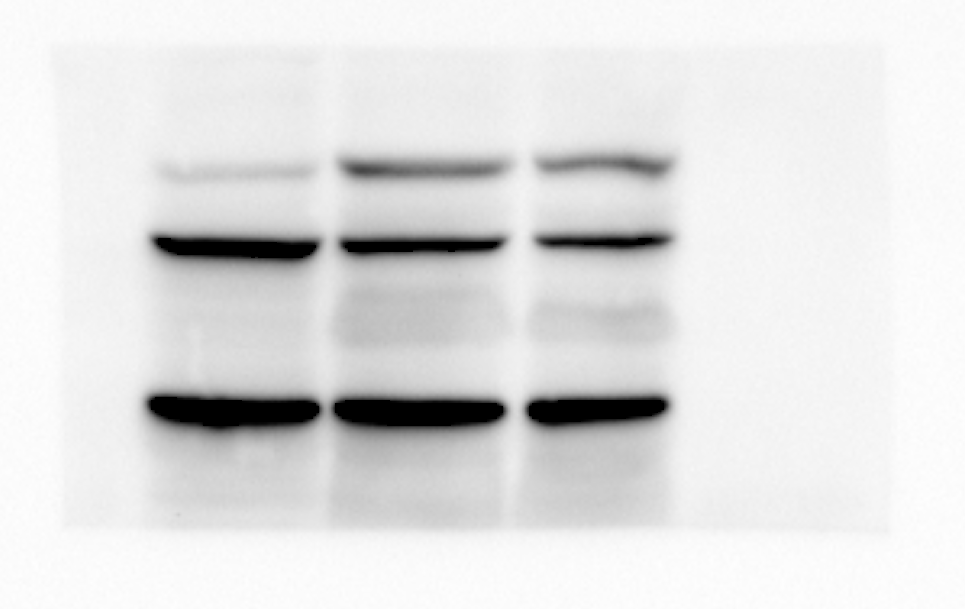

Supplement: Figure 5—source data 3. [file elife-97896-fig5-data3.zip › Figure 5-Source Data 3-21. /Figure 5-Source Data 14. Full raw unedited blot (sfGFP-Slp1 input, blot 3) for Figure 5F.tif]

Figure 5-figure supplement 2.

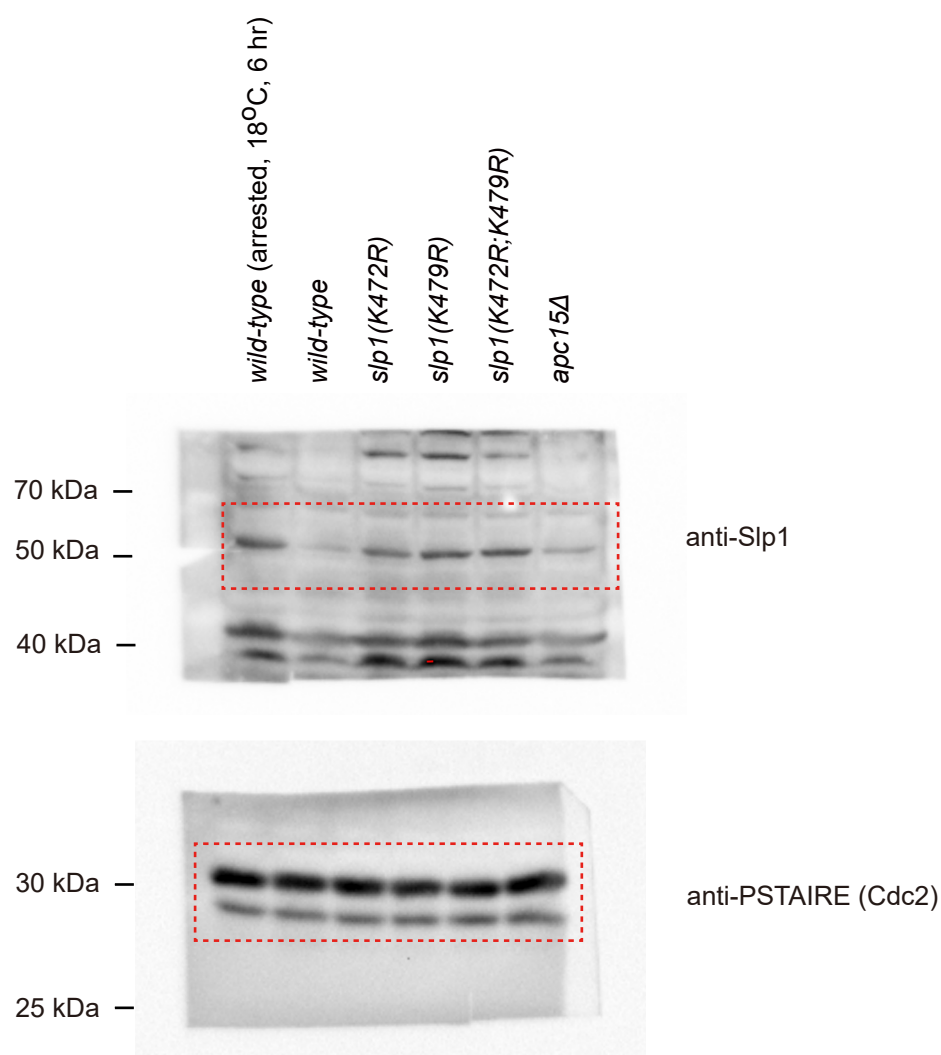

Supplement: Figure 5—figure supplement 2—source data 1. [file elife-97896-fig5-figsupp2-data1.pdf]

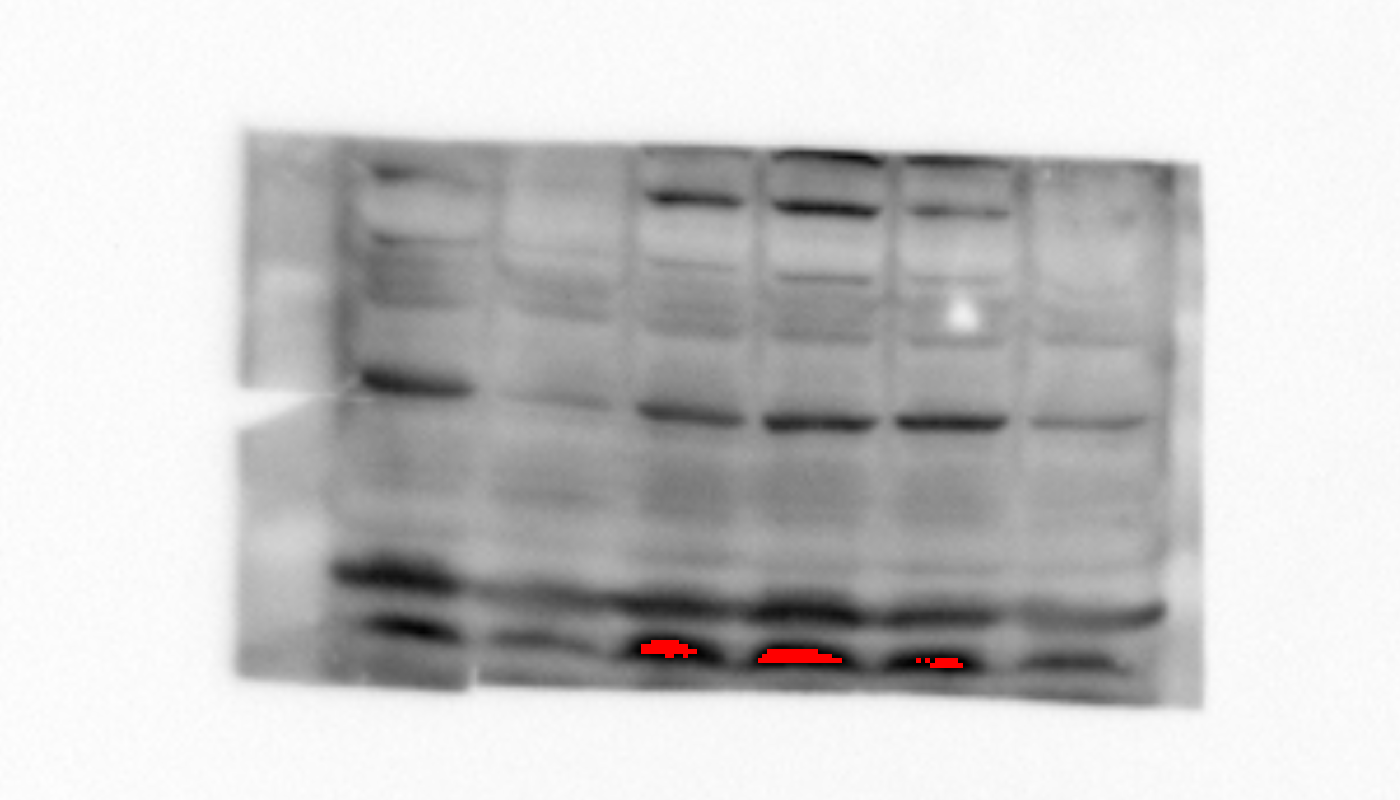

Supplement: Figure 5—figure supplement 2—source data 3. [file elife-97896-fig5-figsupp2-data3.zip › Figure 5-figure supplement 2-Source Data 3. Full raw unedited blot (Slp1) for Figure 5-figure supplement 2.tif]

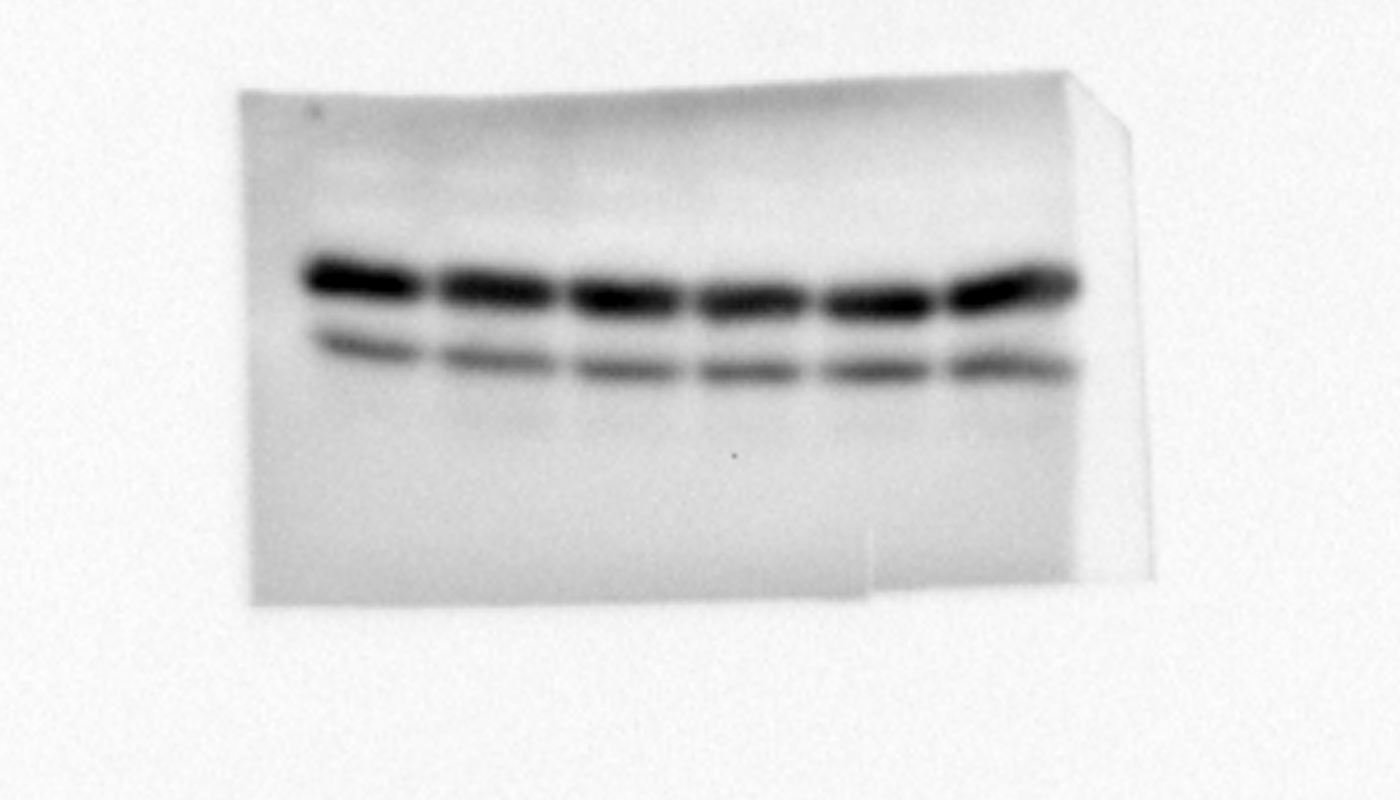

Supplement: Figure 5—figure supplement 2—source data 4. [file elife-97896-fig5-figsupp2-data4.zip › Figure 5-figure supplement 2-Source Data 4. Full raw unedited blot (Cdc2) for Figure 5-figure supplement 2.tif]

Figure 5-figure supplement 5

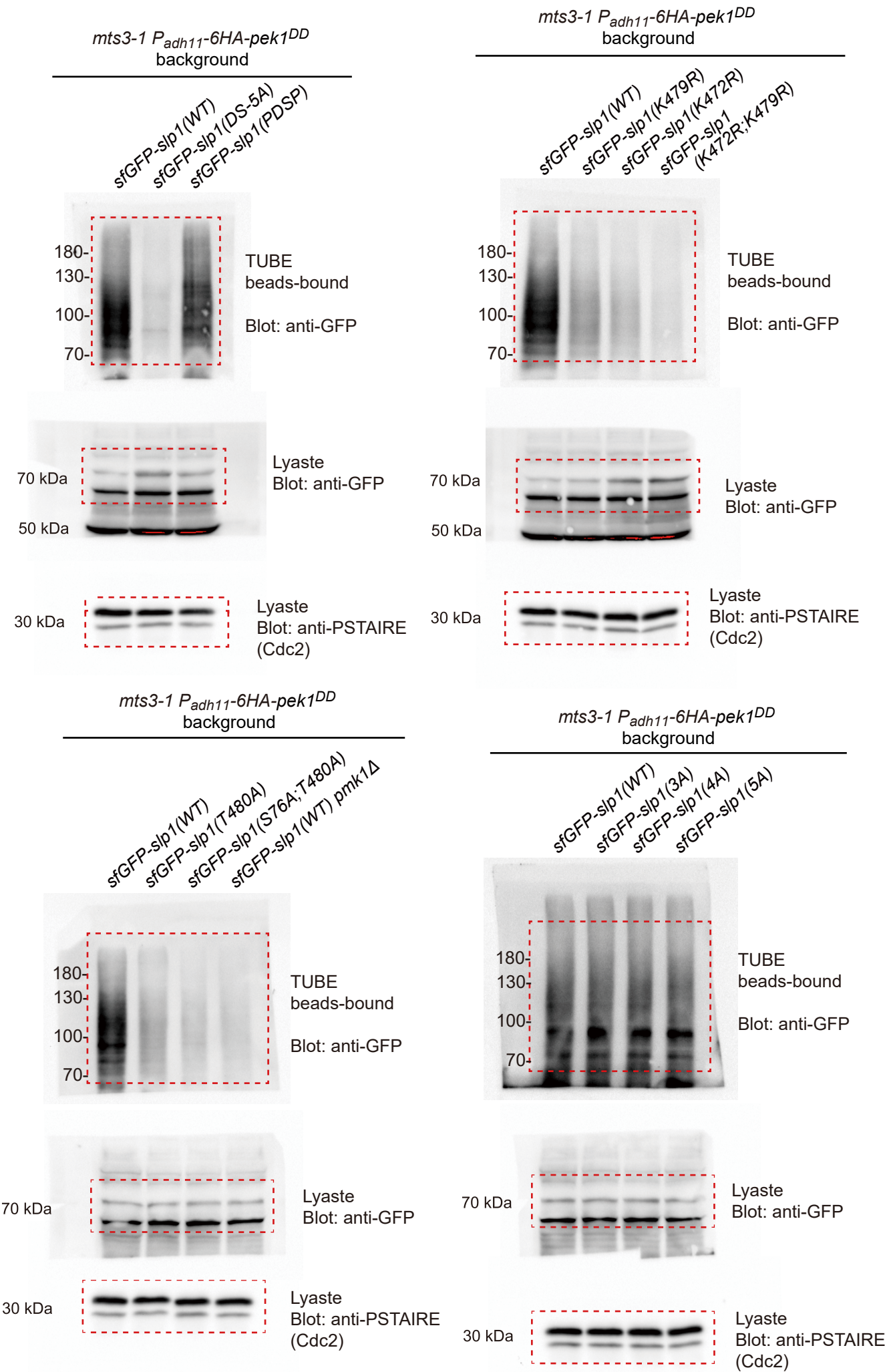

Supplement: Figure 5—figure supplement 5—source data 1. [file elife-97896-fig5-figsupp5-data1.pdf]
