# Supplementary material for: Negative regulation of APC/C activation by MAPK-mediated attenuation of Cdc20Slp1 under stress
Source: eLife. 2024 Oct 16;13:RP97896. doi: 10.7554/eLife.97896 (PMC11483130; doi:10.7554/eLife.97896)
Supplement: Figure 2—source data 3. [file elife-97896-fig2-data3.zip › Source data titles.docx]

Figure 2-Source Data 3. Full raw unedited blot (IPed Lid1-TAP) for Figure 2A.

Figure 2-Source Data 4. Full raw unedited blot (co-IPed Mad2-GFP/Mad3-GFP) for Figure 2A.

Figure 2-Source Data 5. Full raw unedited blot (co-IPed Slp1) for Figure 2A.

Figure 2-Source Data 6. Full raw unedited blot (Lid1-TAP input) for Figure 2A.

Figure 2-Source Data 7. Full raw unedited blot (Mad2-GFP/Mad3-GFP input) for Figure 2A.

Figure 2-Source Data 8. Full raw unedited blot (Slp1 input) for Figure 2A.

Figure 2-Source Data 9. Full raw unedited blot (Cdc2 input) for Figure 2A.

Figure 2-Source Data 10. Full raw unedited blot (phosphorylated Sty1) for Figure 2B.

Figure 2-Source Data 11. Full raw unedited blot (phosphorylated Pmk1) for Figure 2B.

Figure 2-Source Data 12. Full raw unedited blot (Slp1) for Figure 2B.

Figure 2-Source Data 13. Full raw unedited blot (Cdc2) for Figure 2B.

Figure 2-Source Data 14. Full raw unedited blot (Slp1) for Figure 2C.

Figure 2-Source Data 15. Full raw unedited blot (Cdc2) for Figure 2C
