# Supplementary material for: Negative regulation of APC/C activation by MAPK-mediated attenuation of Cdc20Slp1 under stress
Source: eLife. 2024 Oct 16;13:RP97896. doi: 10.7554/eLife.97896 (PMC11483130; doi:10.7554/eLife.97896)
Supplement: Figure 3—source data 3. [file elife-97896-fig3-data3.zip › Source data titles.docx]

Figure 3-Source Data 3. Full raw unedited blot (bead-bound Pmk1-HA) for Figure 3A.

Figure 3-Source Data 4. Full raw unedited blot (Pmk1-HA input) for Figure 3A.

Figure 3-Source Data 5. Full raw unedited blot (Cdc2 input) for Figure 3A.

Figure 3-Source Data 6. Full raw unedited Coomassie gel (MBP & MBP-Slp1) for Figure 3A.

Figure 3-Source Data 7. Full raw unedited Coomassie gel (bead-bound MBP-Slp1 & GST-Pmk1, left) for Figure 3C.

Figure 3-Source Data 8. Full raw unedited Coomassie gel (bead-bound MBP-Slp1 & GST-Pmk1, right) for Figure 3C.

Figure 3-Source Data 9. Full raw unedited Coomassie gel (input, left) for Figure 3C.

Figure 3-Source Data 10. Full raw unedited Coomassie gel (input, right) for Figure 3C.

Figure 3-Source Data 11. Full raw unedited blot (Slp1, left) for Figure 3D.

Figure 3-Source Data 12. Full raw unedited blot (Slp1, right) for Figure 3D.

Figure 3-Source Data 13. Full raw unedited blot (Cdc2, left) for Figure 3D.

Figure 3-Source Data 14. Full raw unedited blot (Cdc2, right) for Figure 3D.
