# Supplementary material for: Negative regulation of APC/C activation by MAPK-mediated attenuation of Cdc20Slp1 under stress
Source: eLife. 2024 Oct 16;13:RP97896. doi: 10.7554/eLife.97896 (PMC11483130; doi:10.7554/eLife.97896)
Supplement: Figure 4—source data 3. [file elife-97896-fig4-data3.zip › Source data titles Figure 4.docx]

Figure 4-Source Data 3. Full raw unedited blot (anti-thioP) for Figure 4A.

Figure 4-Source Data 4. Full raw unedited blot (anti-HA) for Figure 4A.

Figure 4-Source Data 5. Full raw unedited Coomassie gel (GST-Atf1 & GST-Slp1) for Figure 4A.

Figure 4-Source Data 6. Full raw unedited blot (anti-pT480) for Figure 4C. 1802

Figure 4-Source Data 7. Full raw unedited Coomassie gel (GST-fusions) for Figure 4C.

Figure 4-Source Data 8. Full raw unedited blot (bead-bound, anti-pT480) for Figure 4D.

Figure 4-Source Data 9. Full raw unedited blot (bead-bound, anti-GST) for Figure 4D.

Figure 4-Source Data 10. Full raw unedited blot (anti-GST, input) for Figure 4D.

Figure 4-Source Data 11. Full raw unedited blot (Cdc2, input) for Figure 4D.

Figure 4-Source Data 12. Full raw unedited blot (anti-pS28/pT31) for Figure 4E.

Figure 4-Source Data 13. Full raw unedited blot (anti-GFP) for Figure 4E.

Figure 4-Source Data 14. Full raw unedited blot (Cdc2 input) for Figure 4E.

Figure 4-Source Data 15. Full raw unedited Coomassie gel (MBP-Slp1(1-190aa)) for Figure 4E.

Figure 4-Source Data 16. Full raw unedited blot (anti-myc IP, left) for Figure 4F.

Figure 4-Source Data 17. Full raw unedited blot (anti-myc IP, middle) for Figure 4F.

Figure 4-Source Data 18. Full raw unedited blot (anti-myc IP, right) for Figure 4F.

Figure 4-Source Data 19. Full raw unedited blot (anti-pS28/pT31 after anti-myc IP, left) for Figure 4F.

Figure 4-Source Data 20. Full raw unedited blot (anti-pS28/pT31 after anti-myc IP, 58 middle) for Figure 4F.

Figure 4-Source Data 21. Full raw unedited blot (anti-pS28/pT31 after anti-myc IP, right) for Figure 4F.

Figure 4-Source Data 22. Full raw unedited blot (Slp1 input, left) for Figure 4F.

Figure 4-Source Data 23. Full raw unedited blot (Slp1 input, middle) for Figure 4F.

Figure 4-Source Data 24. Full raw unedited blot (Slp1 input, right) for Figure 4F.

Figure 4-Source Data 25. Full raw unedited blot (Slp1, left) for Figure 4G.

Figure 4-Source Data 26. Full raw unedited blot (Slp1, right) for Figure 4G

Figure 4-Source Data 27. Full raw unedited blot (Cdc2, left) for Figure 4G.

Figure 4-Source Data 28. Full raw unedited blot (Cdc2, right) for Figure 4G.
