# Supplementary material for: Negative regulation of APC/C activation by MAPK-mediated attenuation of Cdc20Slp1 under stress
Source: eLife. 2024 Oct 16;13:RP97896. doi: 10.7554/eLife.97896 (PMC11483130; doi:10.7554/eLife.97896)
Supplement: Figure 5—source data 3. [file elife-97896-fig5-data3.zip › Source data titles for figure 5.docx]

Figure 5-Source Data 3. Full raw unedited blot (Slp1) for Figure 5A.

Figure 5-Source Data 4. Full raw unedited blot (Cdc2) for Figure 5A.

Figure 5-Source Data 5. Full raw unedited blot (Slp1) for Figure 5C.

Figure 5-Source Data 6. Full raw unedited blot (Cdc2) for Figure 5C.

Figure 5-Source Data 7. Full raw unedited blot (bead-bound sfGFP-Slp1, blot 1) for Figure 5F.

Figure 5-Source Data 8. Full raw unedited blot (bead-bound sfGFP-Slp1, blot 2) for Figure 5F.

Figure 5-Source Data 9. Full raw unedited blot (bead-bound sfGFP-Slp1, blot 3) for Figure 5F.

Figure 5-Source Data 10. Full raw unedited blot (bead-bound sfGFP-Slp1, blot 4) for Figure 5F.

Figure 5-Source Data 11. Full raw unedited blot (bead-bound sfGFP-Slp1, blot 5) for Figure 5F.

Figure 5-Source Data 12. Full raw unedited blot (sfGFP-Slp1 input, blot 1) for Figure 5F.

Figure 5-Source Data 13. Full raw unedited blot (sfGFP-Slp1 input, blot 2) for Figure 5F.

Figure 5-Source Data 14. Full raw unedited blot (sfGFP-Slp1 input, blot 3) for Figure 5F.

Figure 5-Source Data 15. Full raw unedited blot (sfGFP-Slp1 input, blot 4) for Figure 5F.

Figure 5-Source Data 16. Full raw unedited blot (sfGFP-Slp1 input, blot 5) for Figure 5F.

Figure 5-Source Data 17. Full raw unedited blot (Cdc2 input, blot 1) for Figure 5F.

Figure 5-Source Data 18. Full raw unedited blot (Cdc2 input, blot 2) for Figure 5F.

Figure 5-Source Data 19. Full raw unedited blot (Cdc2 input, blot 3) for Figure 5F.

Figure 5-Source Data 20. Full raw unedited blot (Cdc2 input, blot 4) for Figure 5F.

Figure 5-Source Data 21. Full raw unedited blot (Cdc2 input, blot 5) for Figure 5F.
