# Supplementary figures and images for: Negative regulation of APC/C activation by MAPK-mediated attenuation of Cdc20Slp1 under stress (part 2 of 2)
Source: eLife. 2024 Oct 16;13:RP97896. doi: 10.7554/eLife.97896 (PMC11483130; doi:10.7554/eLife.97896)

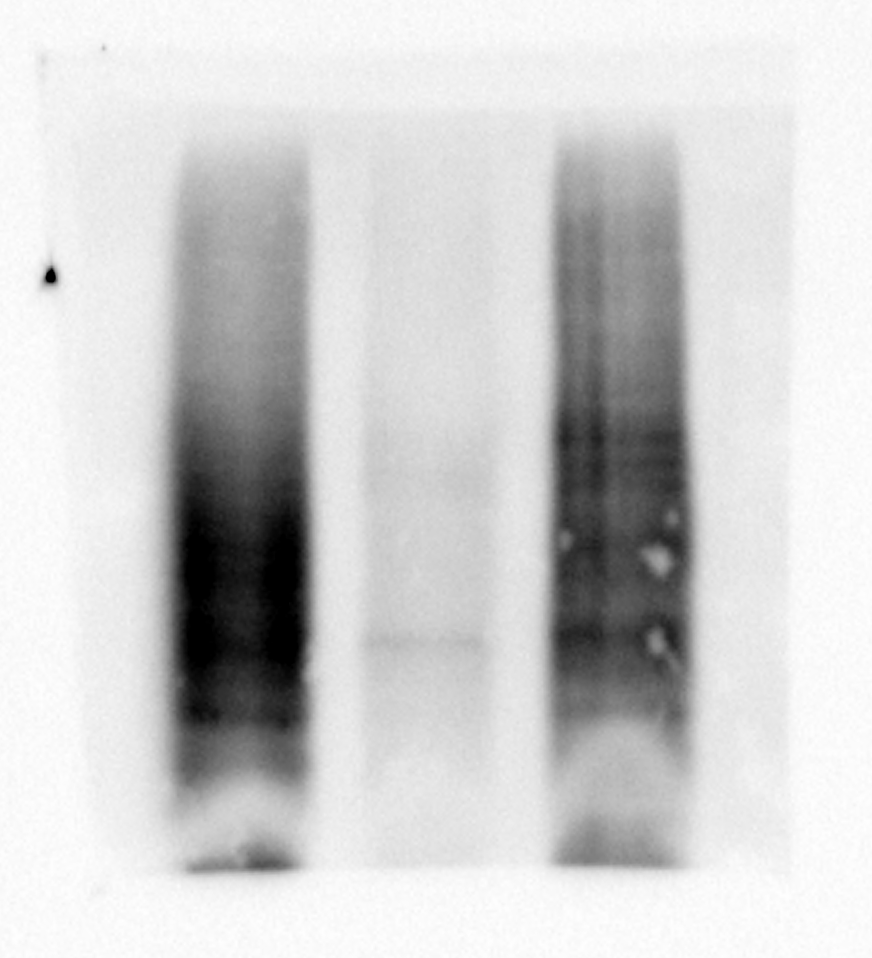

Supplement: Figure 5—figure supplement 5—source data 2. [file elife-97896-fig5-figsupp5-data2.zip › Figure 5-figure supplement 5-Source Data 2. Full raw unedited blot (bead-bound sfGFP-Slp1, blot 1) for Figure 5-figure supplement 5.tif]

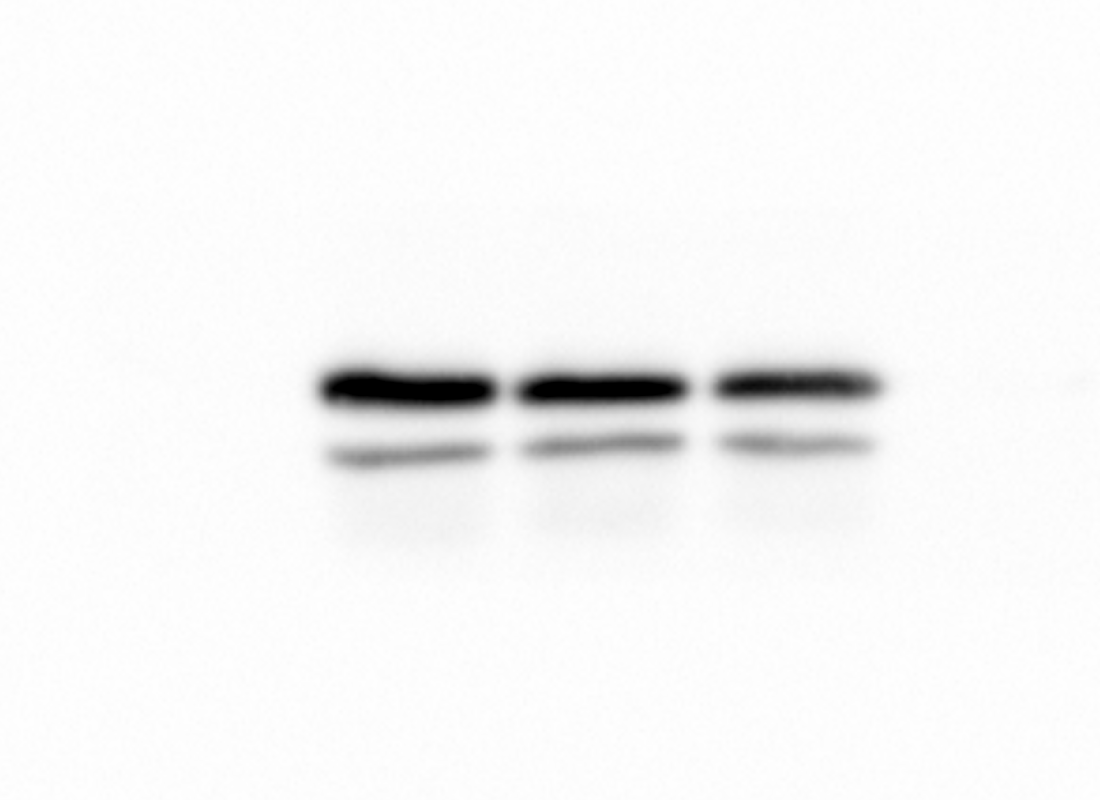

Supplement: Figure 5—figure supplement 5—source data 3. [file elife-97896-fig5-figsupp5-data3.zip › Figure 5-figure supplement 5-Source Data 2-13. /Figure 5-figure supplement 5-Source Data 10. Full raw unedited blot (Cdc2 input, blot 1) for Figure 5-figure supplement 5.tif]

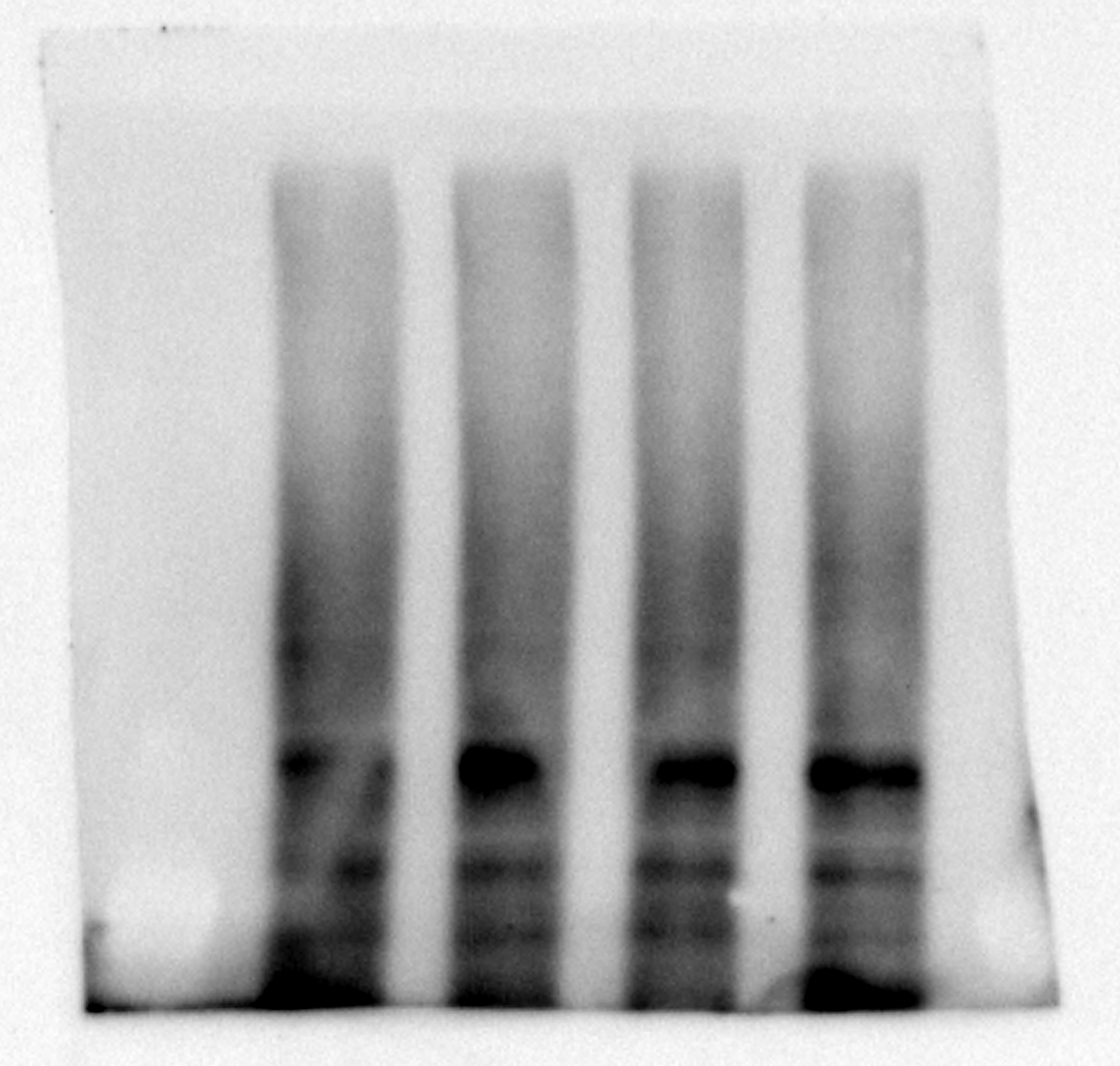

Supplement: Figure 5—figure supplement 5—source data 3. [file elife-97896-fig5-figsupp5-data3.zip › Figure 5-figure supplement 5-Source Data 2-13. /Figure 5-figure supplement 5-Source Data 5. Full raw unedited blot (bead-bound sfGFP-Slp1, blot 4) for Figure 5-figure supplement 5.tif]

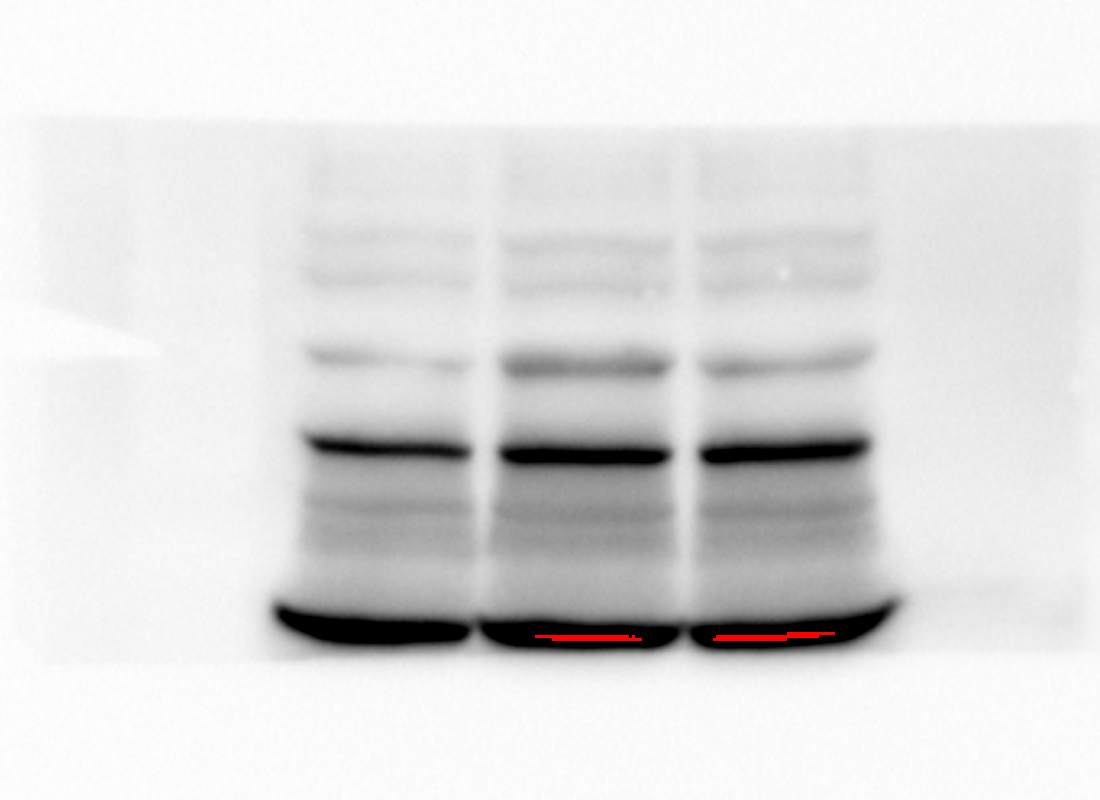

Supplement: Figure 5—figure supplement 5—source data 3. [file elife-97896-fig5-figsupp5-data3.zip › Figure 5-figure supplement 5-Source Data 2-13. /Figure 5-figure supplement 5-Source Data 6. Full raw unedited blot (sfGFP-Slp1 input, blot 1) for Figure 5-figure supplement 5.tif]

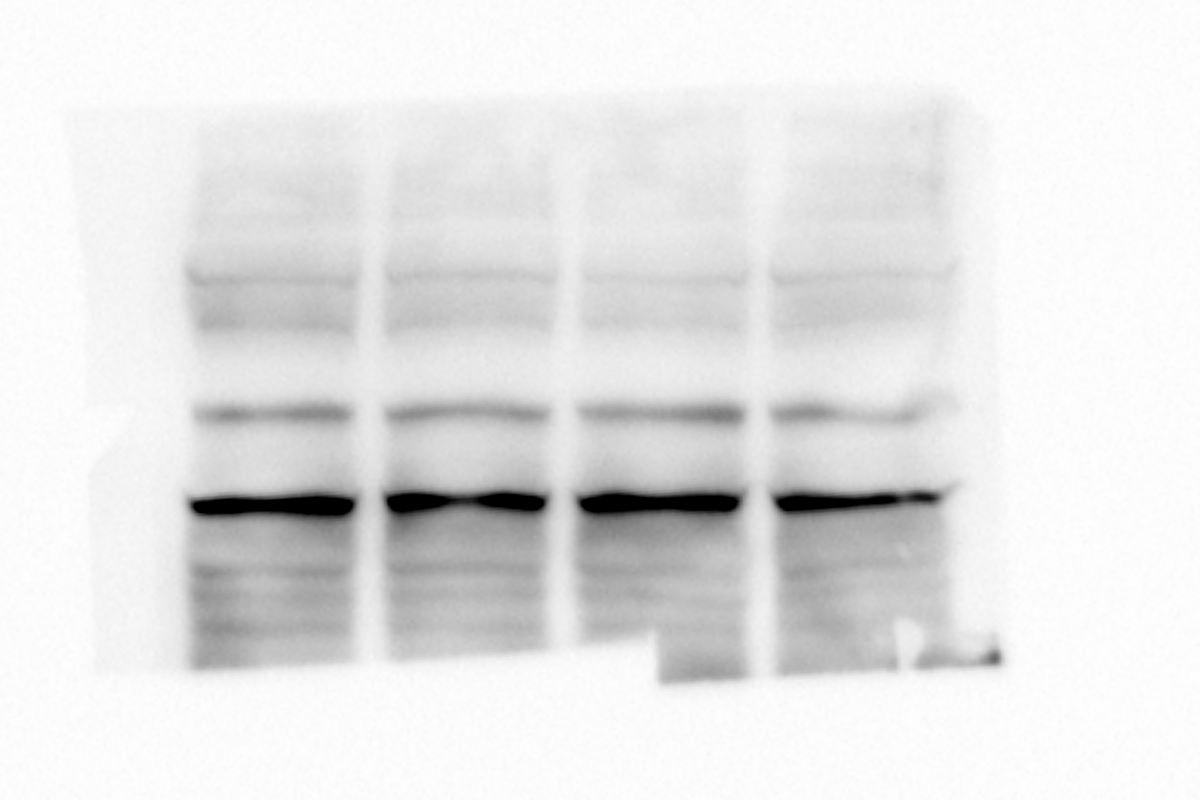

Supplement: Figure 5—figure supplement 5—source data 3. [file elife-97896-fig5-figsupp5-data3.zip › Figure 5-figure supplement 5-Source Data 2-13. /Figure 5-figure supplement 5-Source Data 9. Full raw unedited blot (sfGFP-Slp1 input, blot 4) for Figure 5-figure supplement 5.tif]

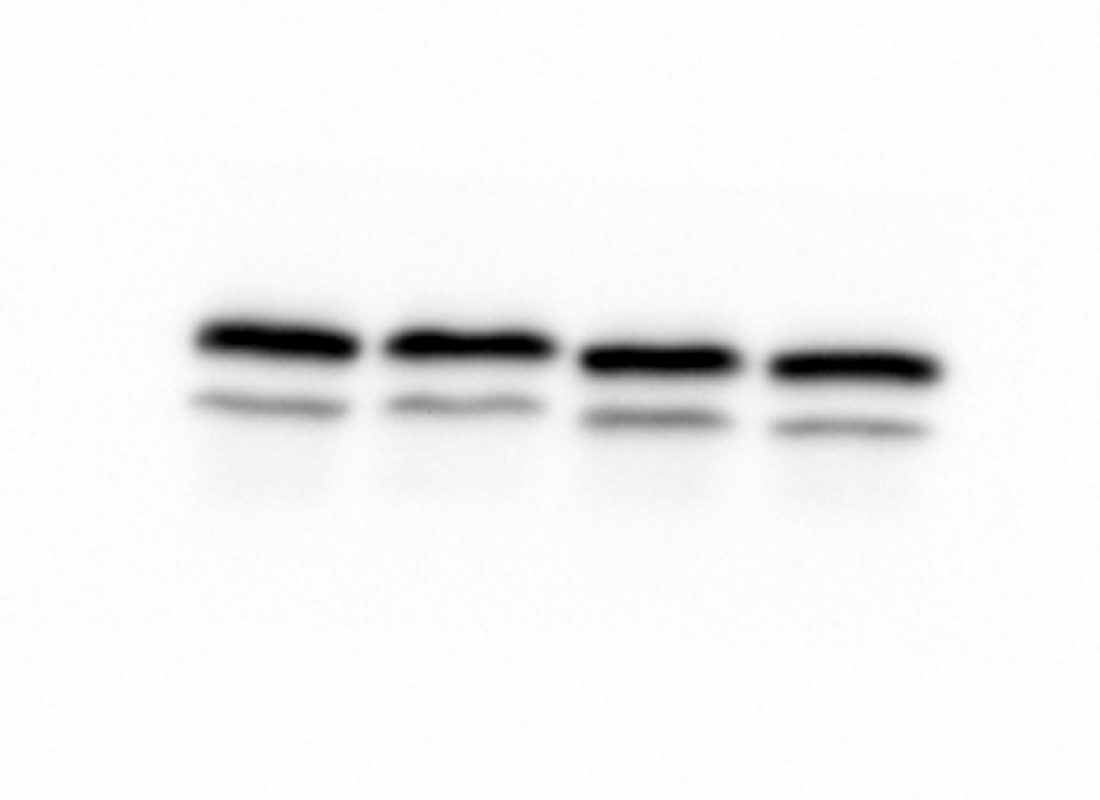

Supplement: Figure 5—figure supplement 5—source data 3. [file elife-97896-fig5-figsupp5-data3.zip › Figure 5-figure supplement 5-Source Data 2-13. /Figure 5-figure supplement 5-Source Data 12. Full raw unedited blot (Cdc2 input, blot 3) for Figure 5-figure supplement 5.tif]

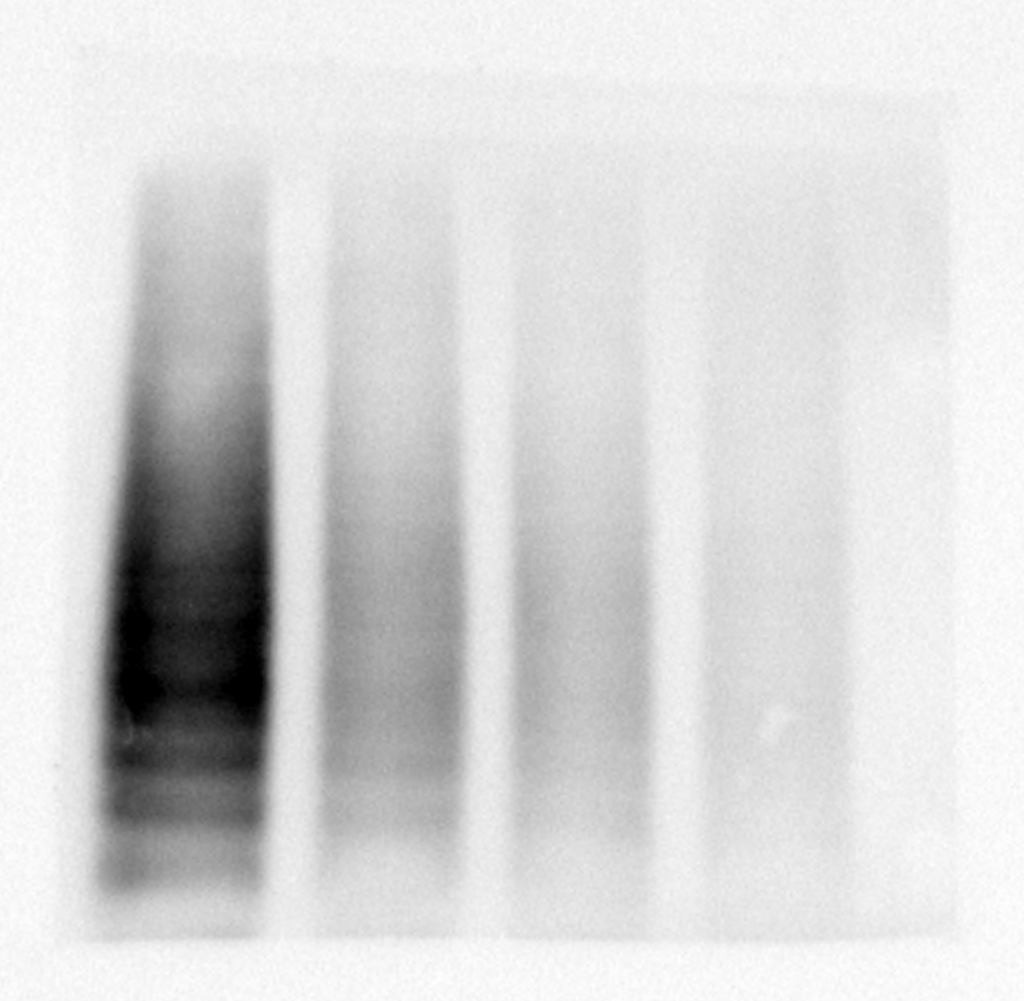

Supplement: Figure 5—figure supplement 5—source data 3. [file elife-97896-fig5-figsupp5-data3.zip › Figure 5-figure supplement 5-Source Data 2-13. /Figure 5-figure supplement 5-Source Data 3. Full raw unedited blot (bead-bound sfGFP-Slp1, blot 2) for Figure 5-figure supplement 5.tif]

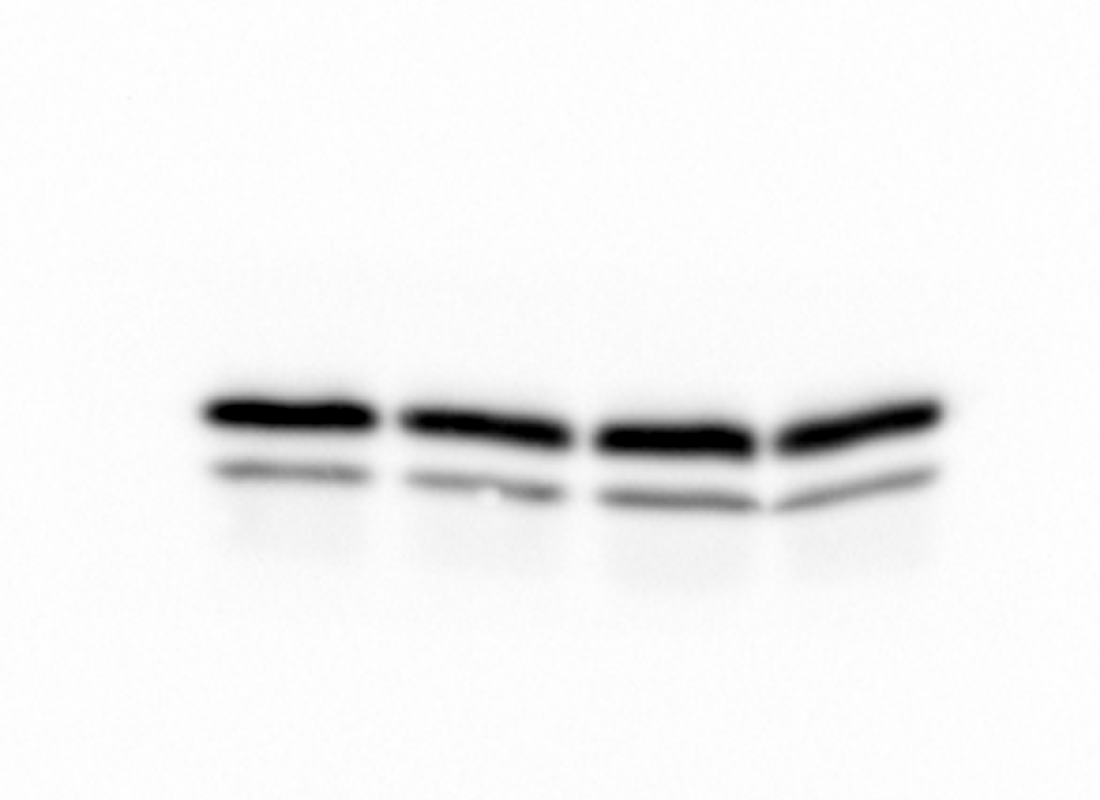

Supplement: Figure 5—figure supplement 5—source data 3. [file elife-97896-fig5-figsupp5-data3.zip › Figure 5-figure supplement 5-Source Data 2-13. /Figure 5-figure supplement 5-Source Data 11. Full raw unedited blot (Cdc2 input, blot 2) for Figure 5-figure supplement 5.tif]

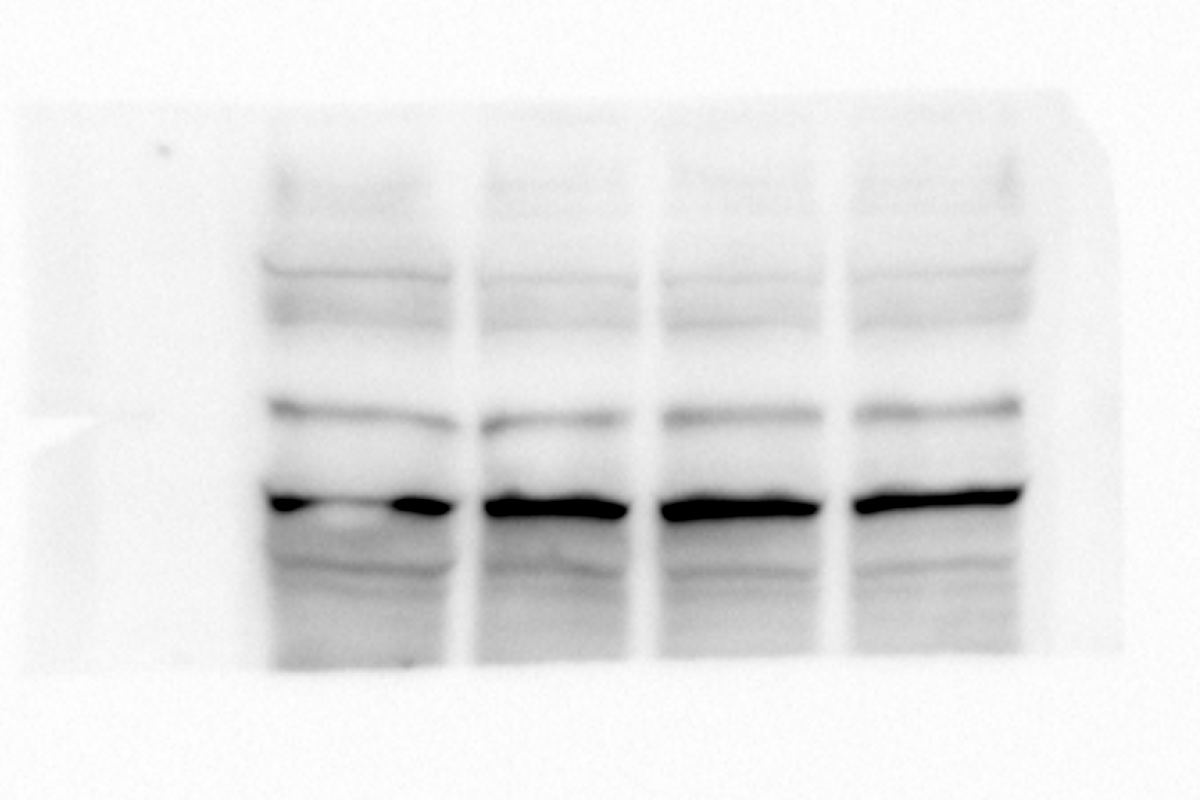

Supplement: Figure 5—figure supplement 5—source data 3. [file elife-97896-fig5-figsupp5-data3.zip › Figure 5-figure supplement 5-Source Data 2-13. /Figure 5-figure supplement 5-Source Data 8. Full raw unedited blot (sfGFP-Slp1 input, blot 3) for Figure 5-figure supplement 5.tif]

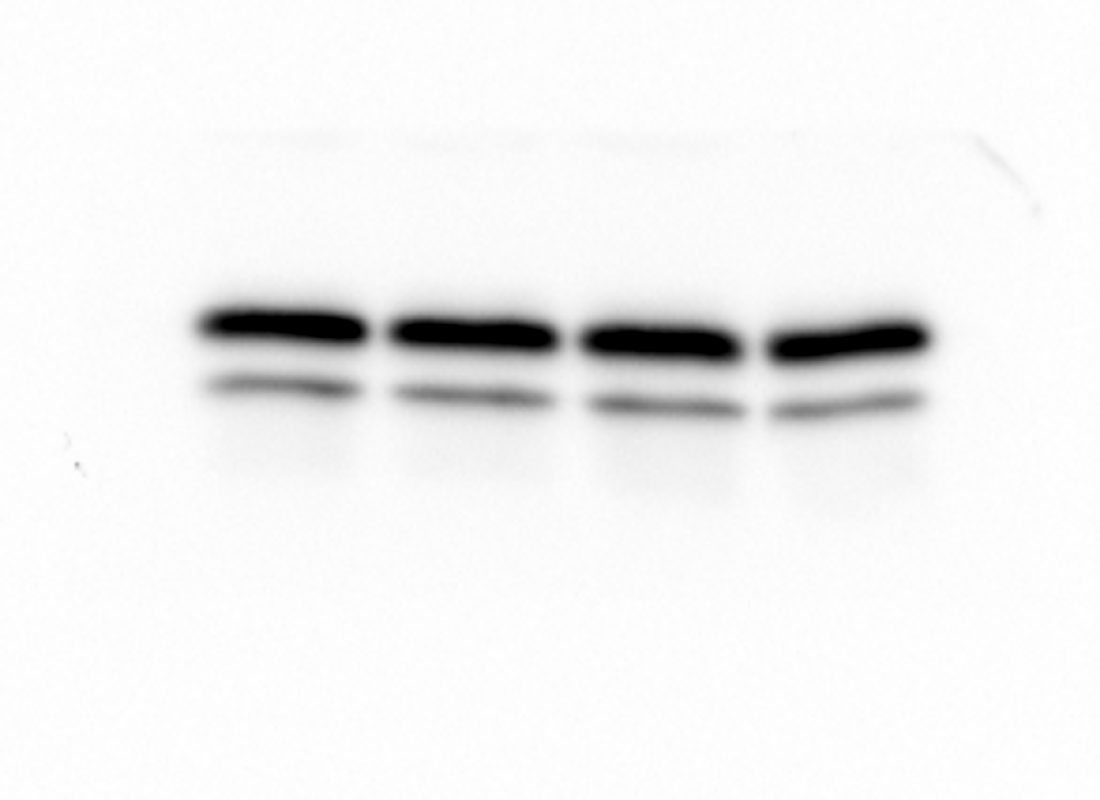

Supplement: Figure 5—figure supplement 5—source data 3. [file elife-97896-fig5-figsupp5-data3.zip › Figure 5-figure supplement 5-Source Data 2-13. /Figure 5-figure supplement 5-Source Data 13. Full raw unedited blot (Cdc2 input, blot 4) for Figure 5-figure supplement 5.tif]

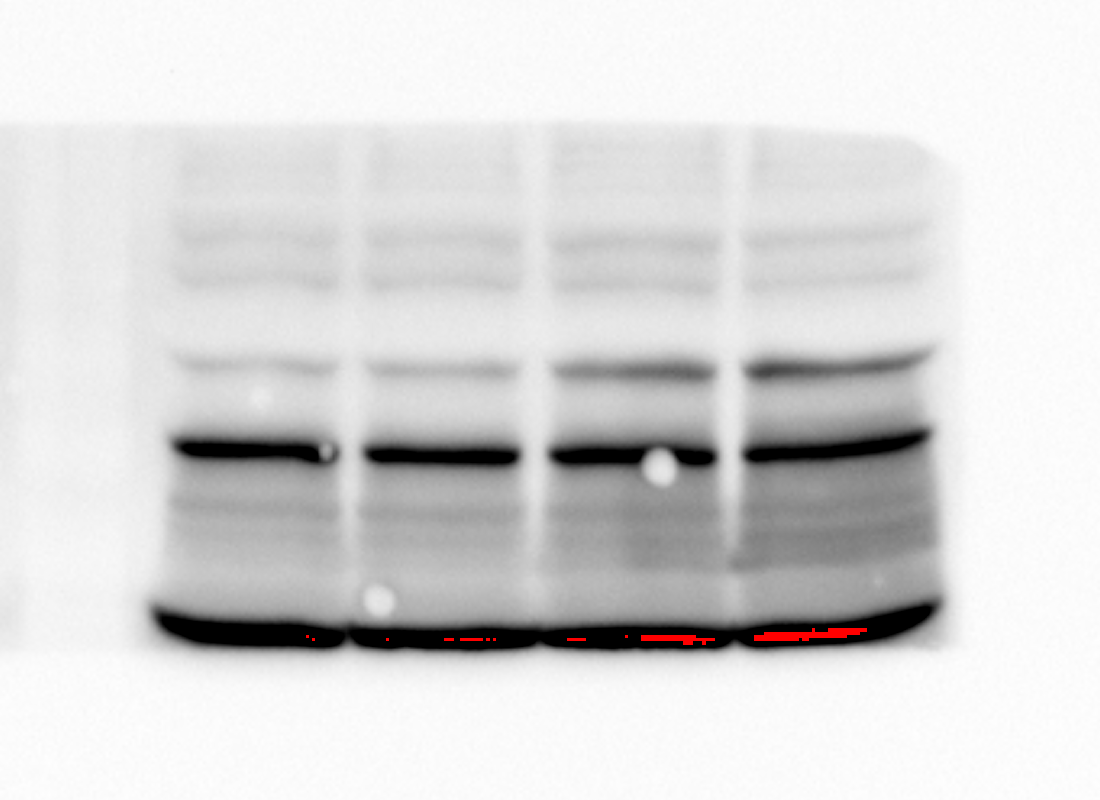

Supplement: Figure 5—figure supplement 5—source data 3. [file elife-97896-fig5-figsupp5-data3.zip › Figure 5-figure supplement 5-Source Data 2-13. /Figure 5-figure supplement 5-Source Data 7. Full raw unedited blot (sfGFP-Slp1 input, blot 2) for Figure 5-figure supplement 5.tif]

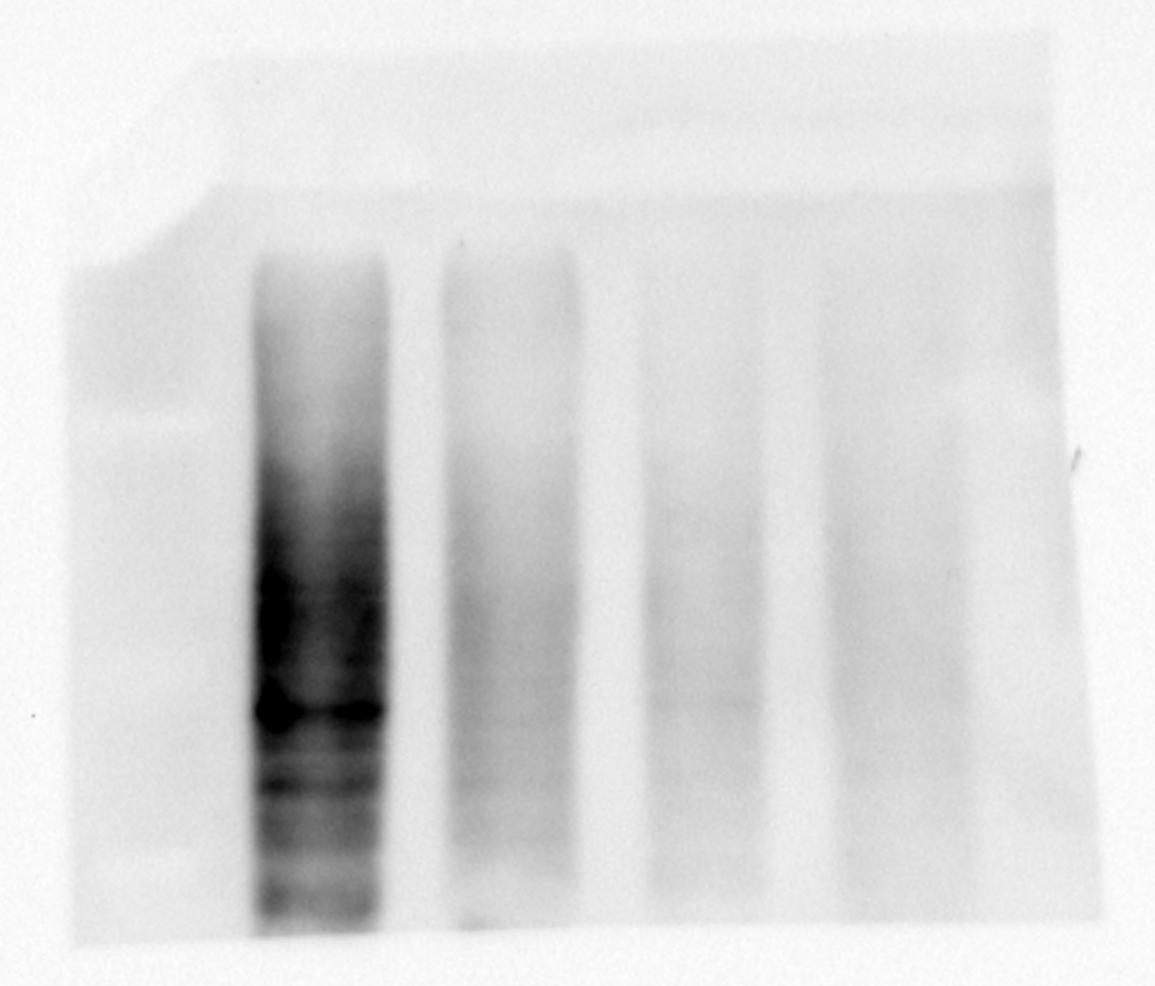

Supplement: Figure 5—figure supplement 5—source data 3. [file elife-97896-fig5-figsupp5-data3.zip › Figure 5-figure supplement 5-Source Data 2-13. /Figure 5-figure supplement 5-Source Data 4. Full raw unedited blot (bead-bound sfGFP-Slp1, blot 3) for Figure 5-figure supplement 5.tif]

Figure 6B.

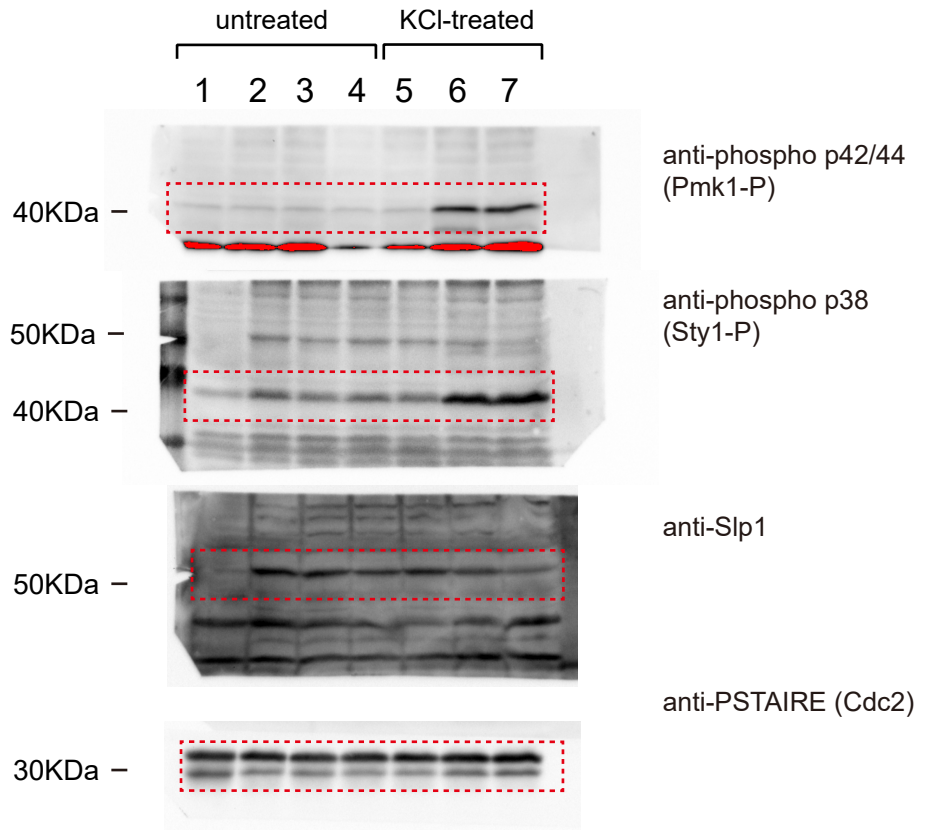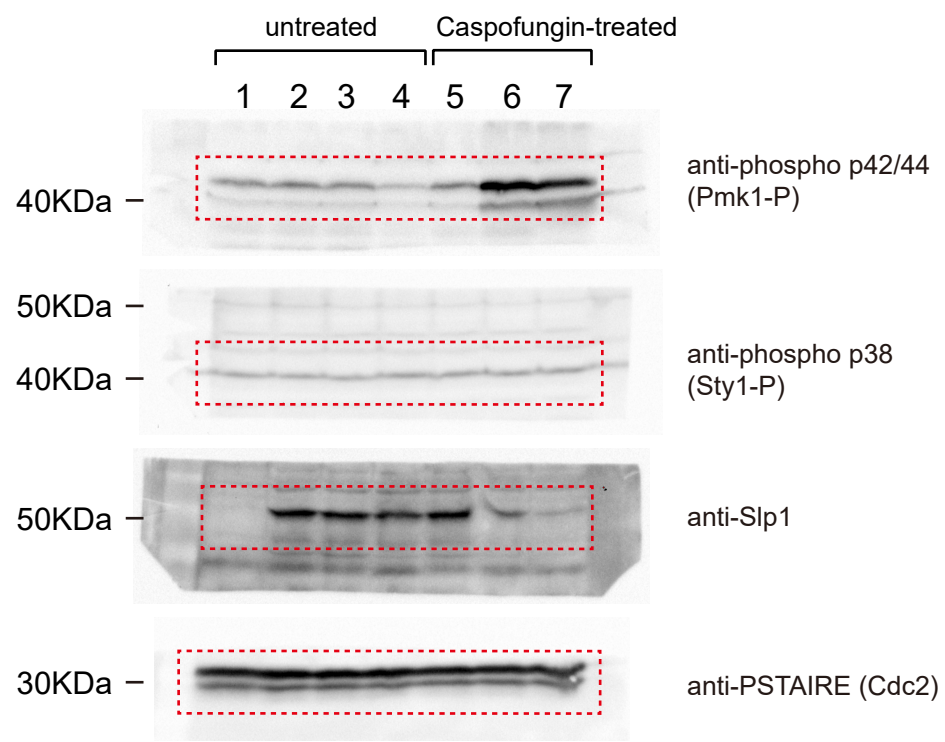

Figure 6C.

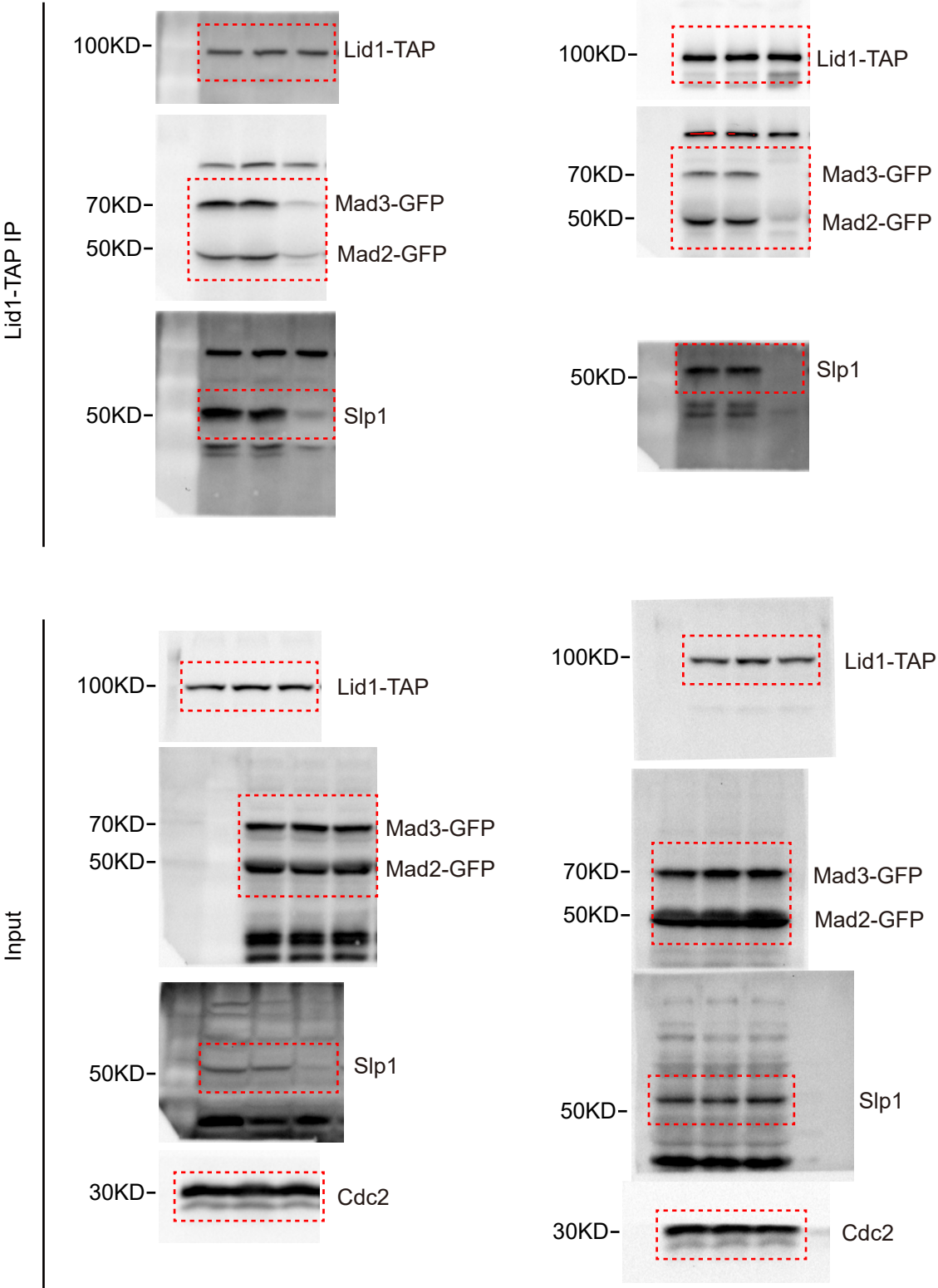

Supplement: Figure 6—source data 1. [file elife-97896-fig6-data1.pdf]

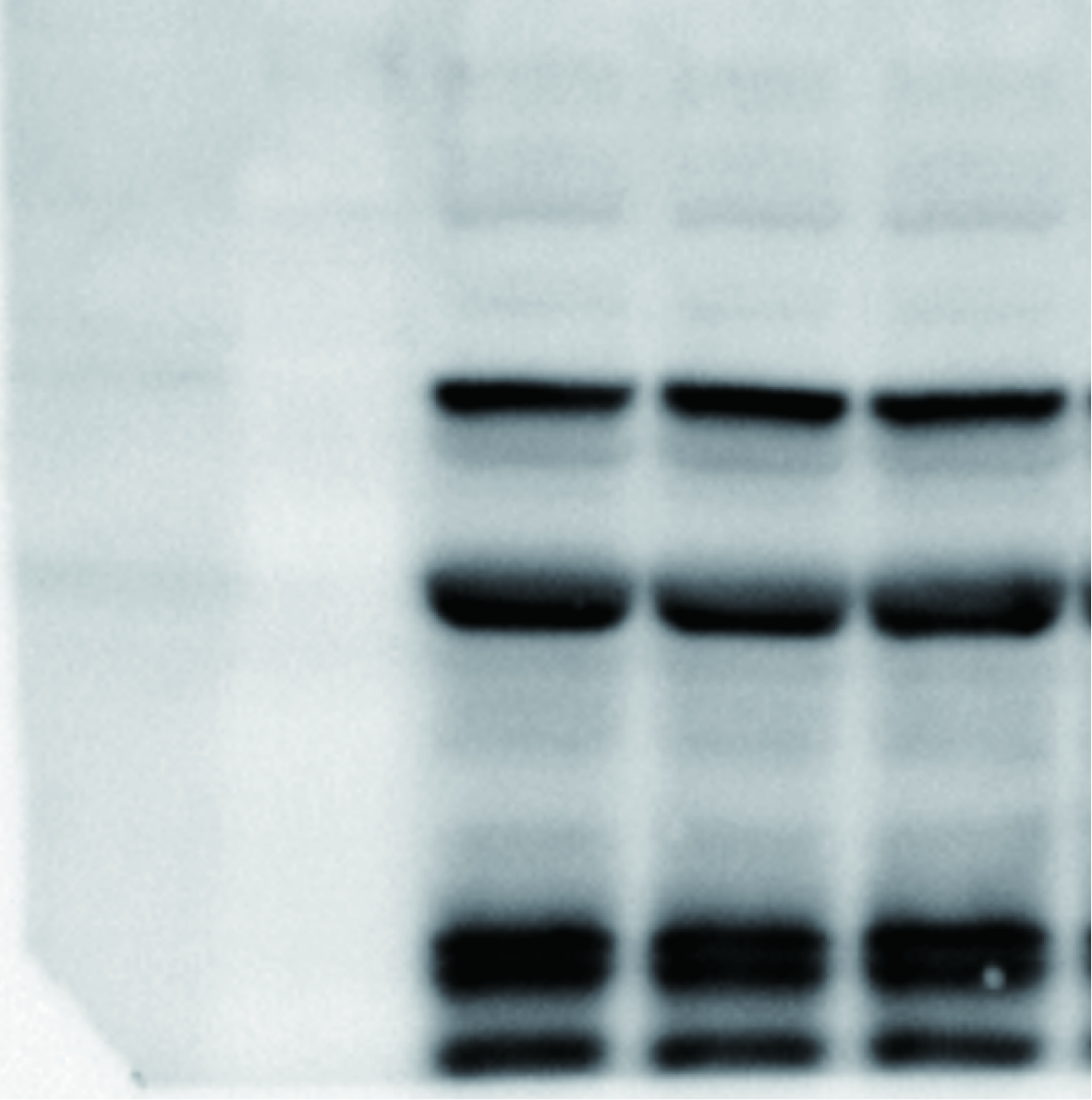

Supplement: Figure 6—source data 3. [file elife-97896-fig6-data3.zip › Figure 6-Source Data 3-24. /Figure 6-Source Data 19. Full raw unedited blot (Mad2-GFP & Mad3-GFP input, left) for Figure 6C.tif]

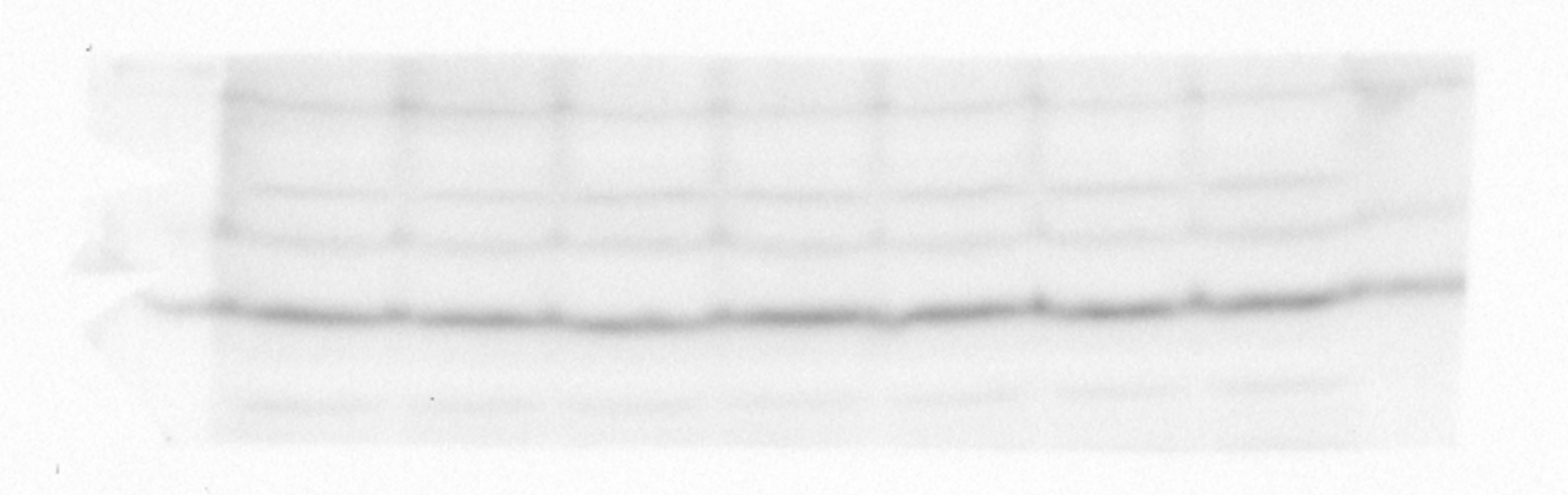

Supplement: Figure 6—source data 3. [file elife-97896-fig6-data3.zip › Figure 6-Source Data 3-24. /Figure 6-Source Data 8. Full raw unedited blot (Sty1-P, Caspofungin-treated group) for Figure 6B.tif]

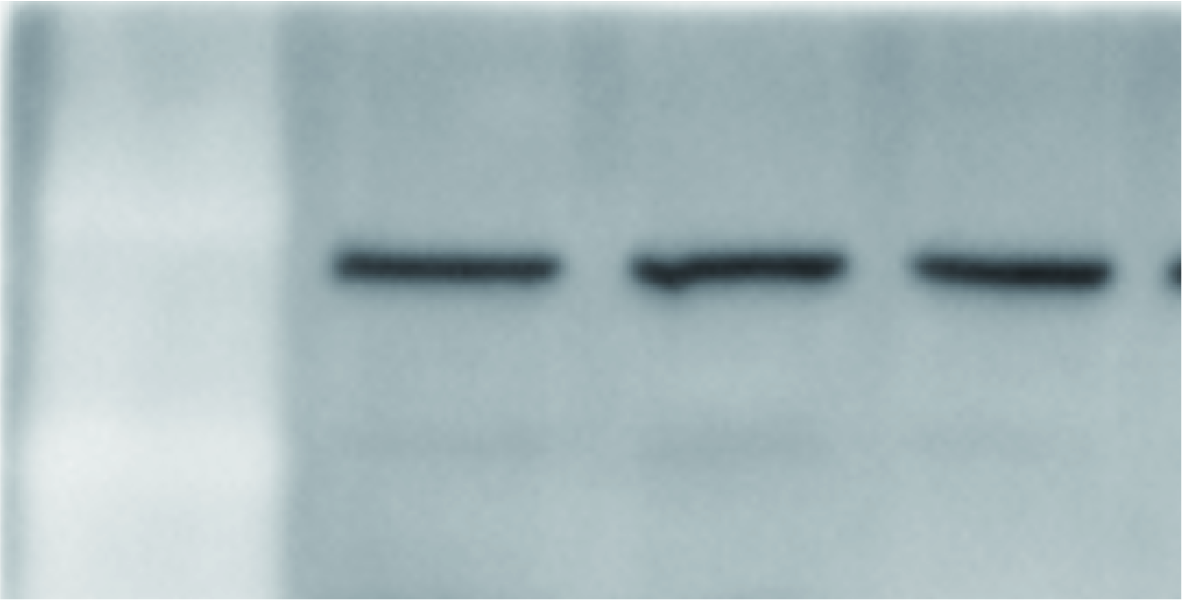

Supplement: Figure 6—source data 3. [file elife-97896-fig6-data3.zip › Figure 6-Source Data 3-24. /Figure 6-Source Data 11. Full raw unedited blot (IPed Lid1-TAP, left) for Figure 6C.tif]

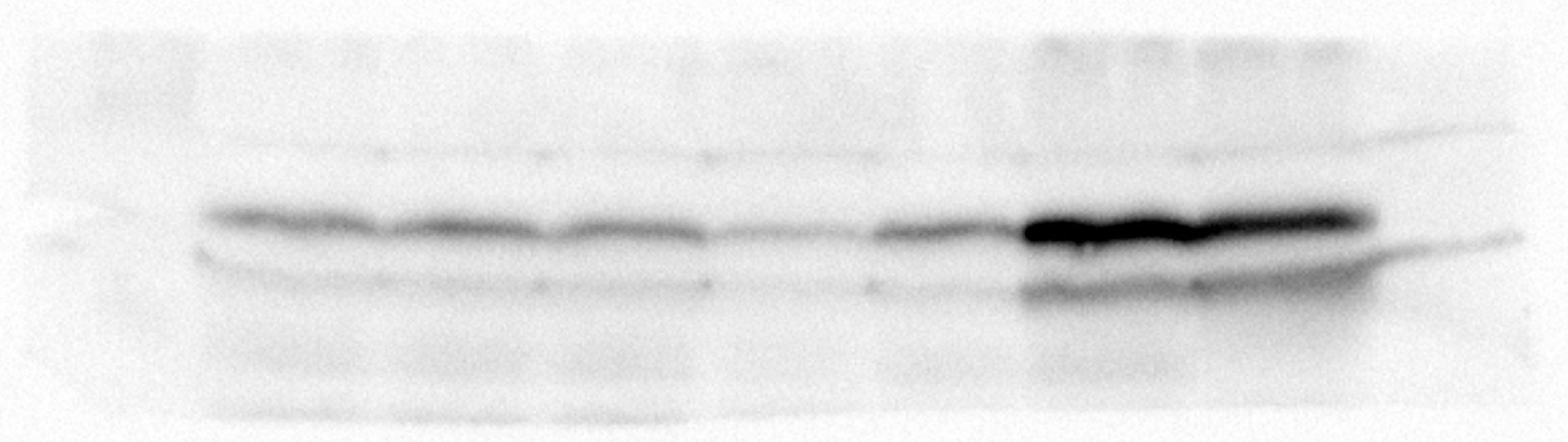

Supplement: Figure 6—source data 3. [file elife-97896-fig6-data3.zip › Figure 6-Source Data 3-24. /Figure 6-Source Data 7. Full raw unedited blot (Pmk1-P, Caspofungin-treated group) for Figure 6B.tif]

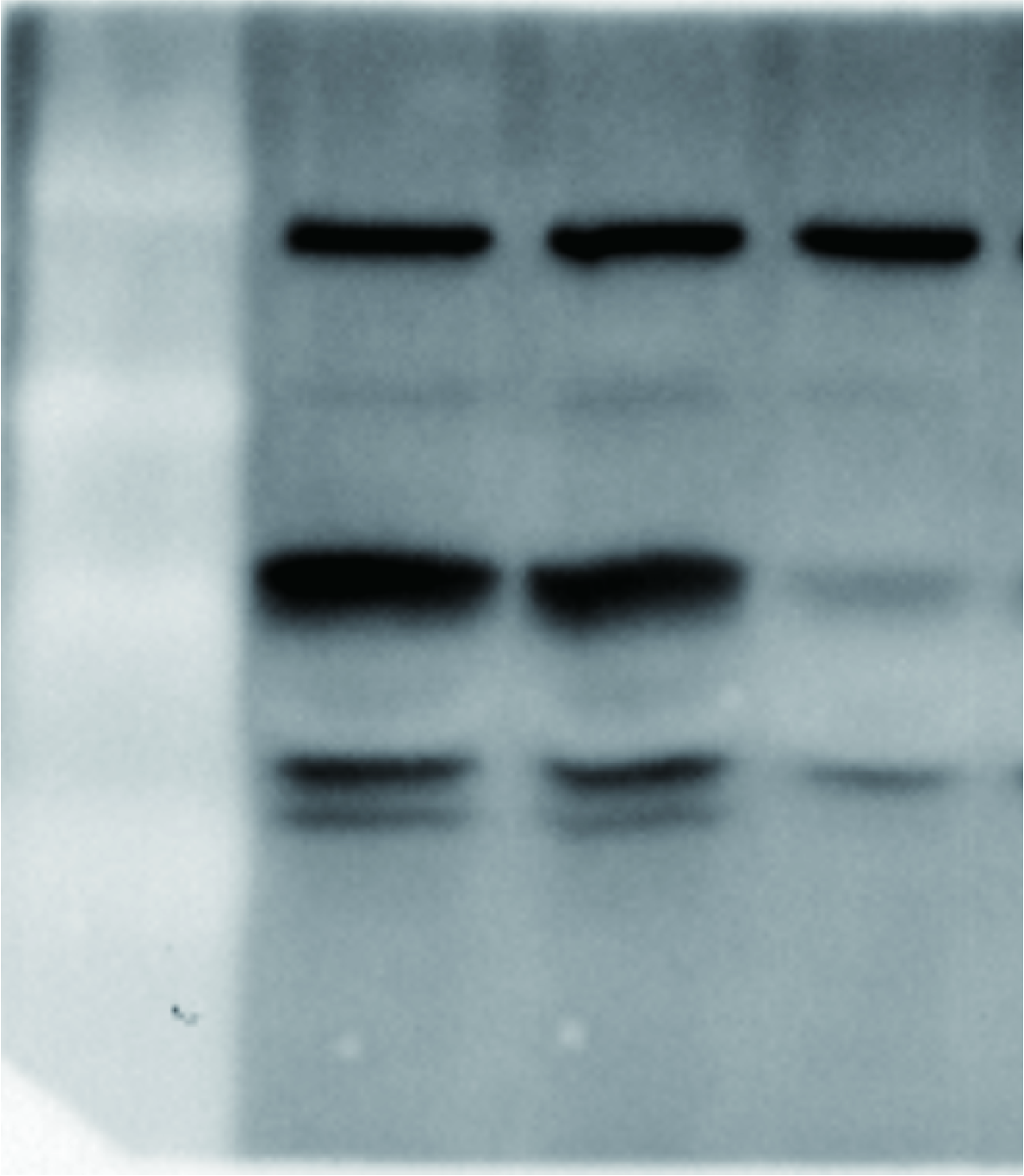

Supplement: Figure 6—source data 3. [file elife-97896-fig6-data3.zip › Figure 6-Source Data 3-24. /Figure 6-Source Data 15. Full raw unedited blot (co-IPed Slp1, left) for Figure 6C.tif]

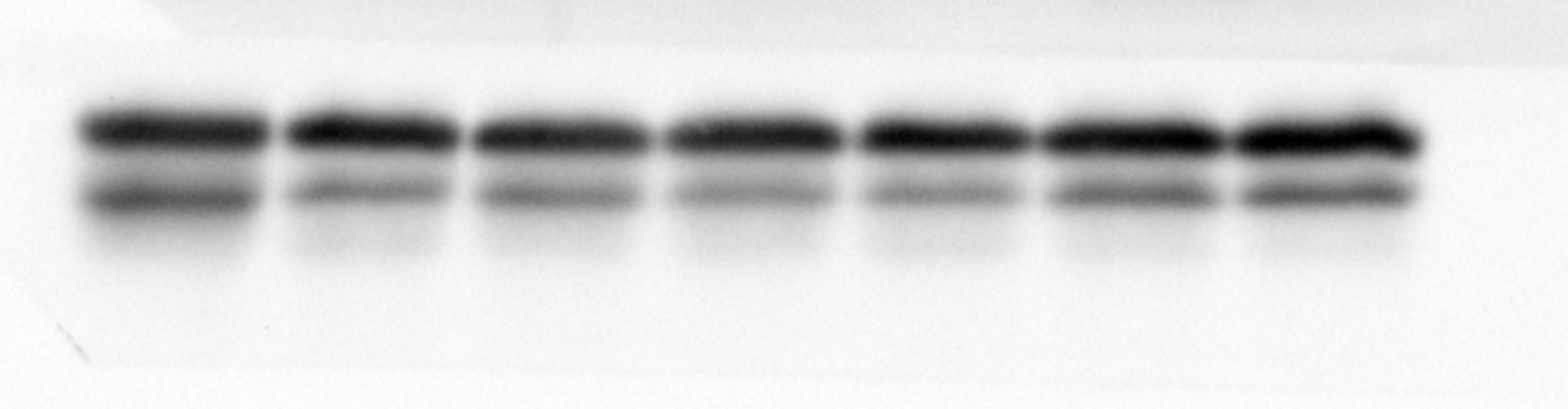

Supplement: Figure 6—source data 3. [file elife-97896-fig6-data3.zip › Figure 6-Source Data 3-24. /Figure 6-Source Data 6. Full raw unedited blot (Cdc2, KCl-treated group) for Figure 6B.tif]

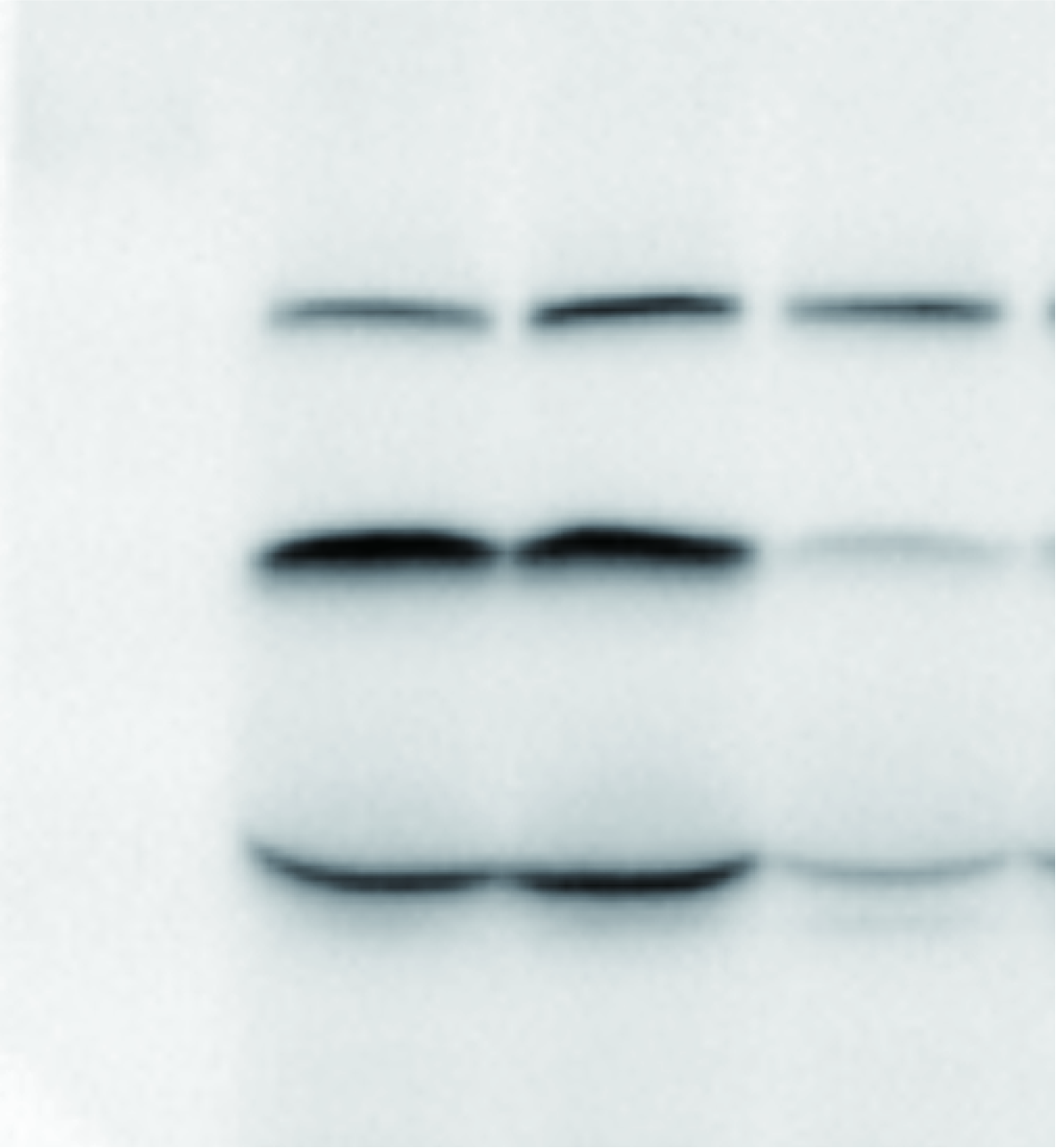

Supplement: Figure 6—source data 3. [file elife-97896-fig6-data3.zip › Figure 6-Source Data 3-24. /Figure 6-Source Data 13. Full raw unedited blot (co-IPed Mad2-GFP & Mad3-GFP, left) for Figure 6C.tif]

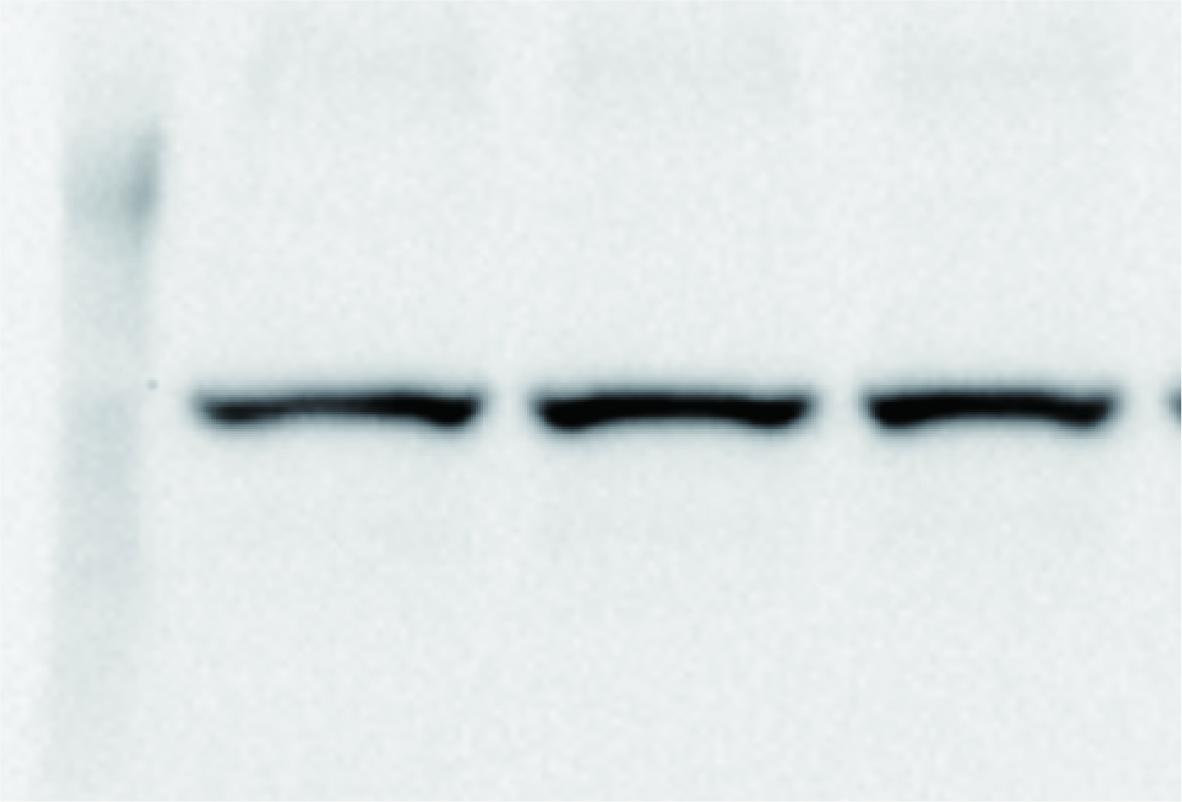

Supplement: Figure 6—source data 3. [file elife-97896-fig6-data3.zip › Figure 6-Source Data 3-24. /Figure 6-Source Data 17. Full raw unedited blot (Lid1-TAP input, left) for Figure 6C.tif]

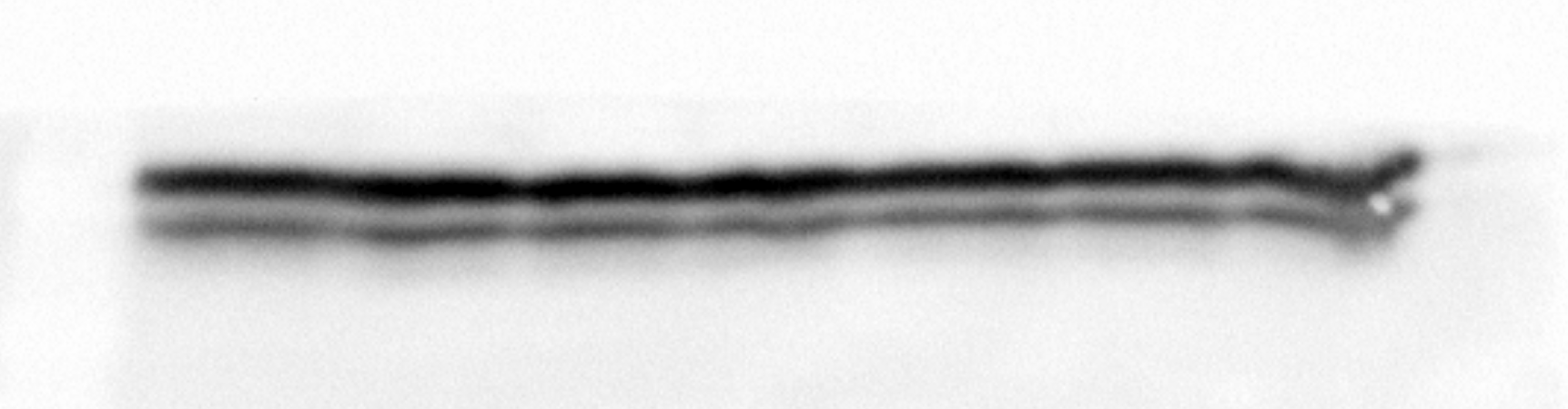

Supplement: Figure 6—source data 3. [file elife-97896-fig6-data3.zip › Figure 6-Source Data 3-24. /Figure 6-Source Data 10. Full raw unedited blot (Cdc2, Caspofungin-treated group) for Figure 6B.tif]

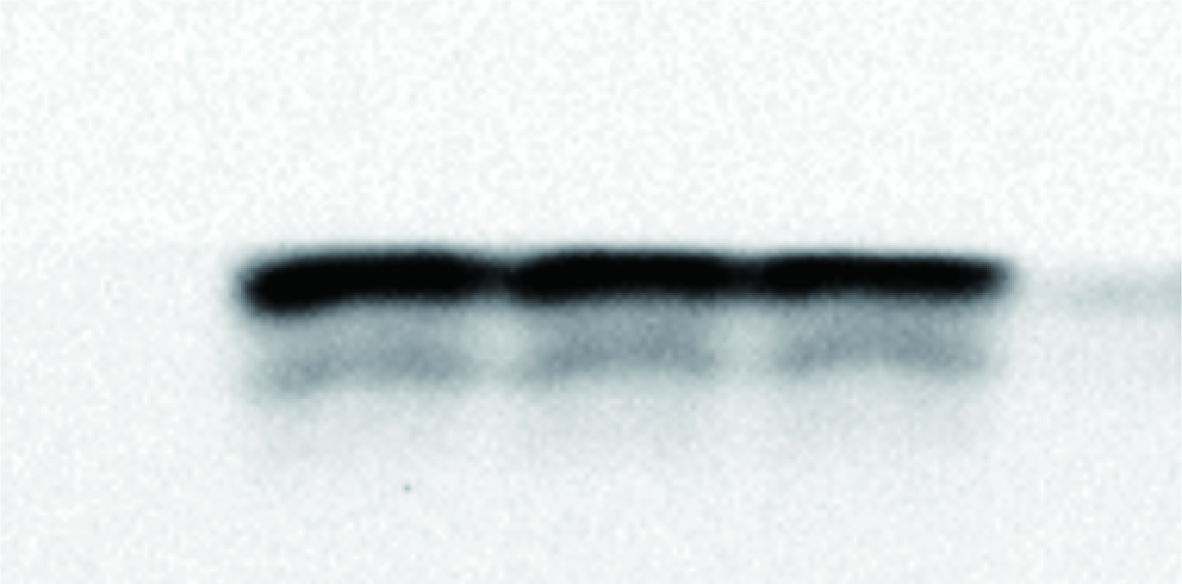

Supplement: Figure 6—source data 3. [file elife-97896-fig6-data3.zip › Figure 6-Source Data 3-24. /Figure 6-Source Data 24. Full raw unedited blot (Cdc2 input, right) for Figure 6C.tif]

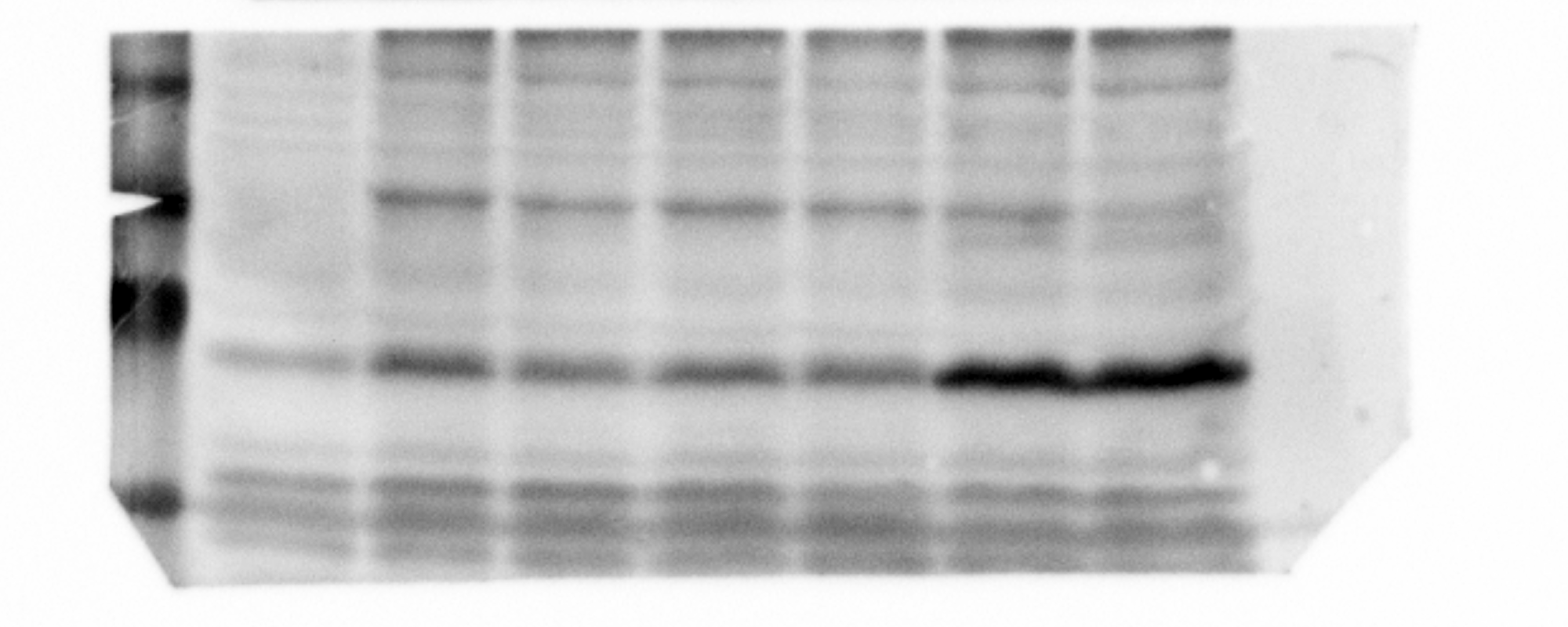

Supplement: Figure 6—source data 3. [file elife-97896-fig6-data3.zip › Figure 6-Source Data 3-24. /Figure 6-Source Data 4. Full raw unedited blot (Sty1-P, KCl-treated group) for Figure 6B.tif]

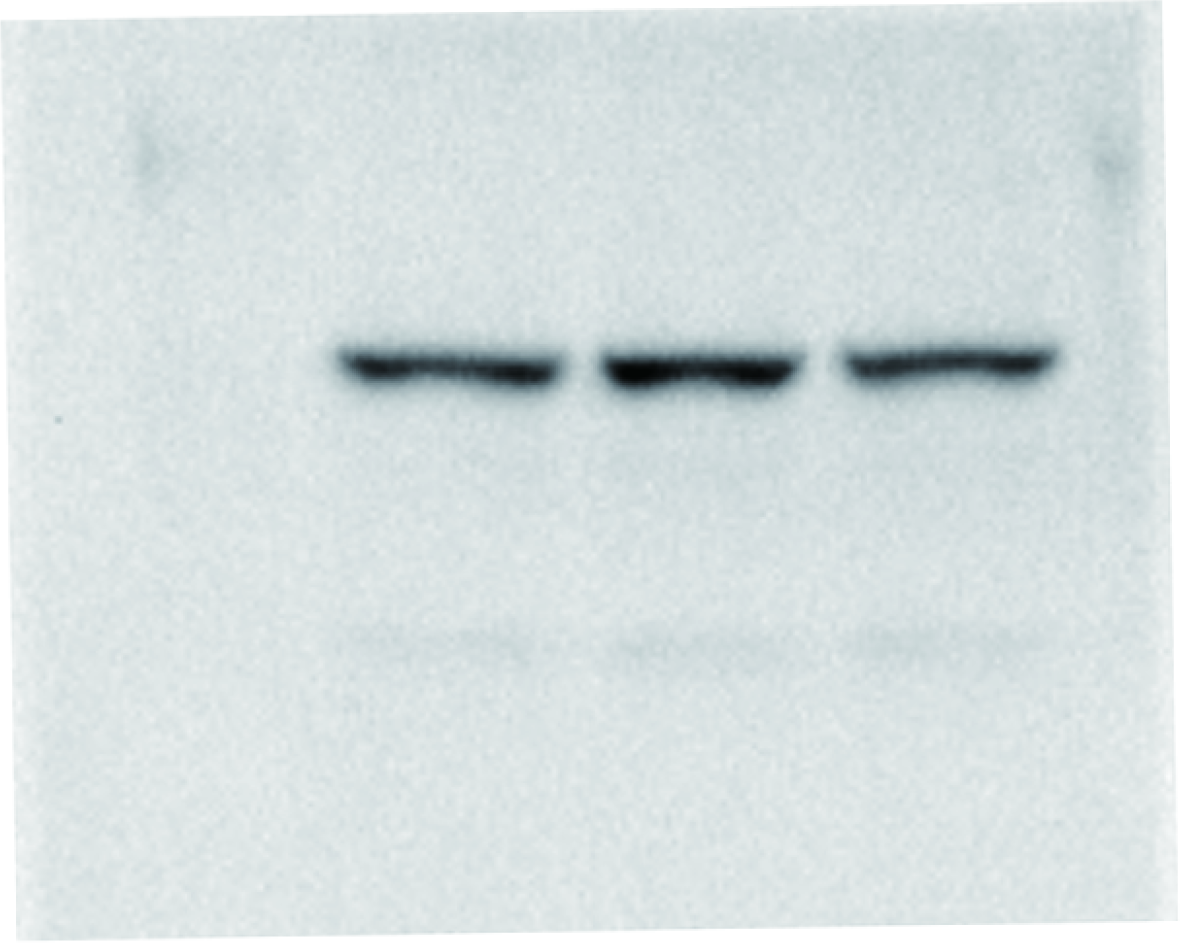

Supplement: Figure 6—source data 3. [file elife-97896-fig6-data3.zip › Figure 6-Source Data 3-24. /Figure 6-Source Data 18. Full raw unedited blot (Lid1-TAP input, right) for Figure 6C.tif]

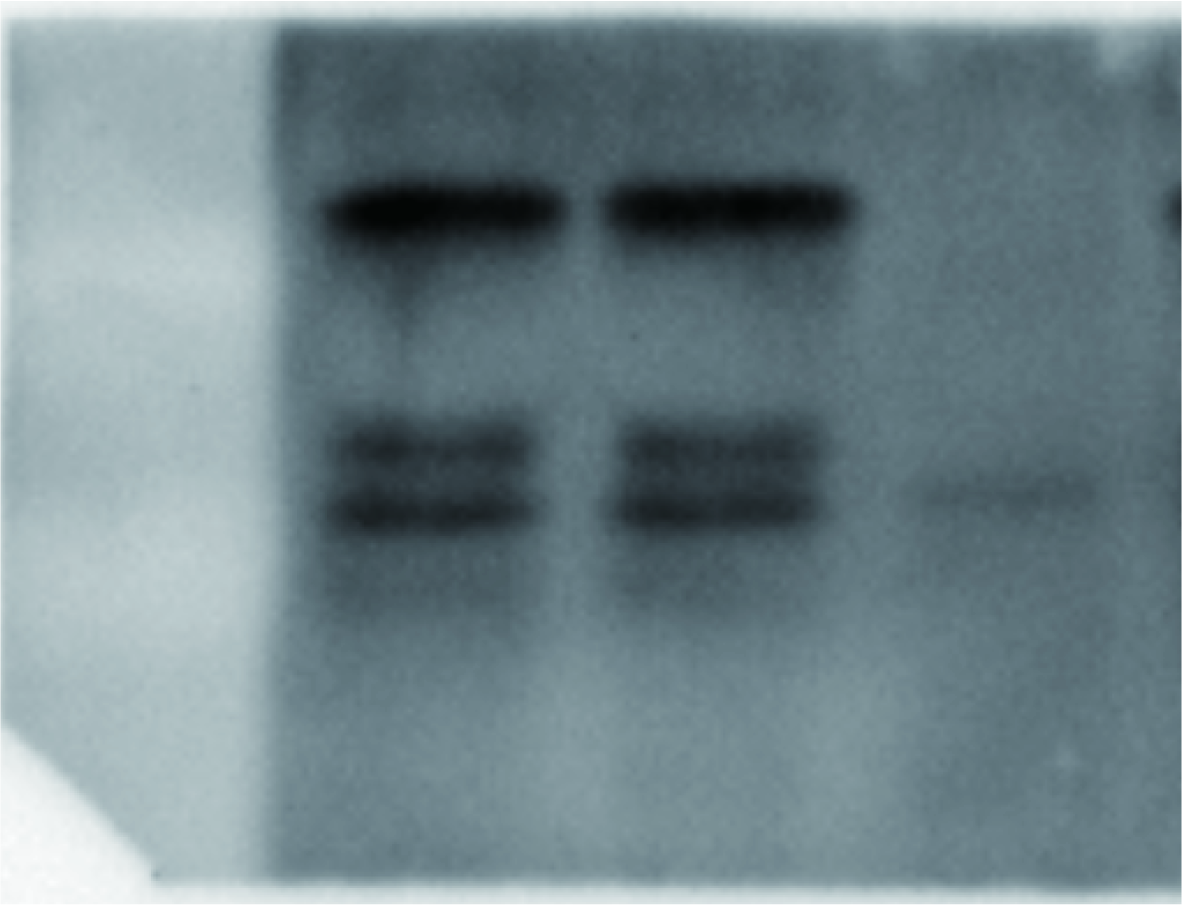

Supplement: Figure 6—source data 3. [file elife-97896-fig6-data3.zip › Figure 6-Source Data 3-24. /Figure 6-Source Data 16. Full raw unedited blot (co-IPed Slp1, right) for Figure 6C.tif]

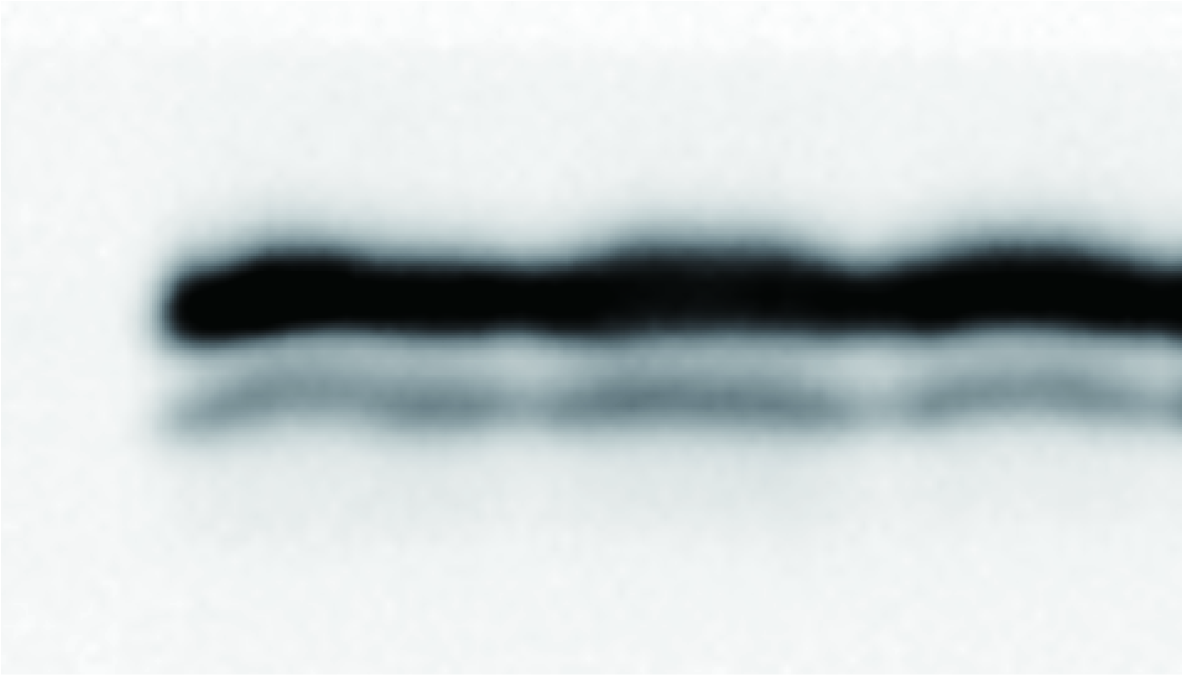

Supplement: Figure 6—source data 3. [file elife-97896-fig6-data3.zip › Figure 6-Source Data 3-24. /Figure 6-Source Data 23. Full raw unedited blot (Cdc2 input, left) for Figure 6C.tif]

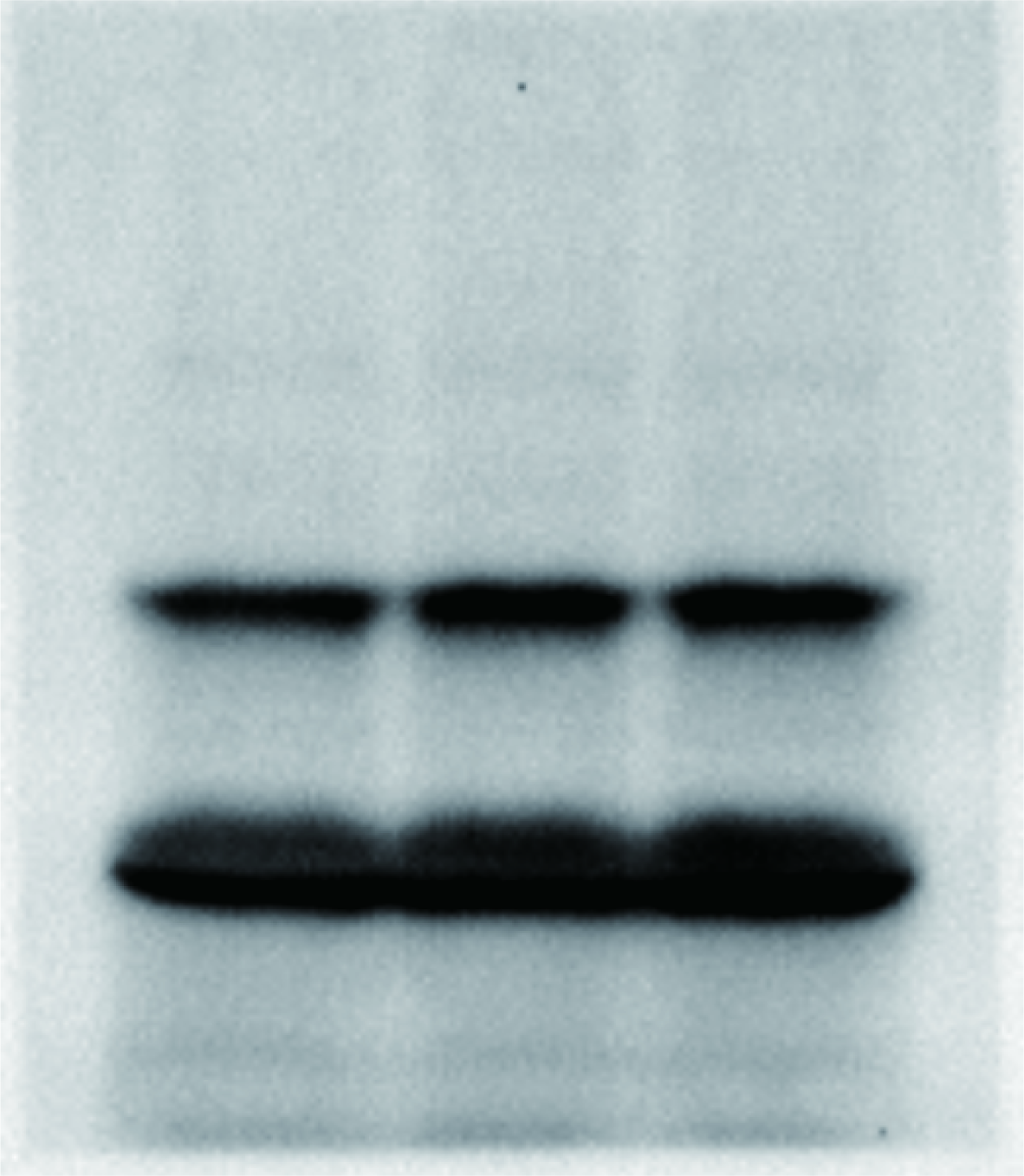

Supplement: Figure 6—source data 3. [file elife-97896-fig6-data3.zip › Figure 6-Source Data 3-24. /Figure 6-Source Data 20. Full raw unedited blot (Mad2-GFP & Mad3-GFP input, right) for Figure 6C.tif]

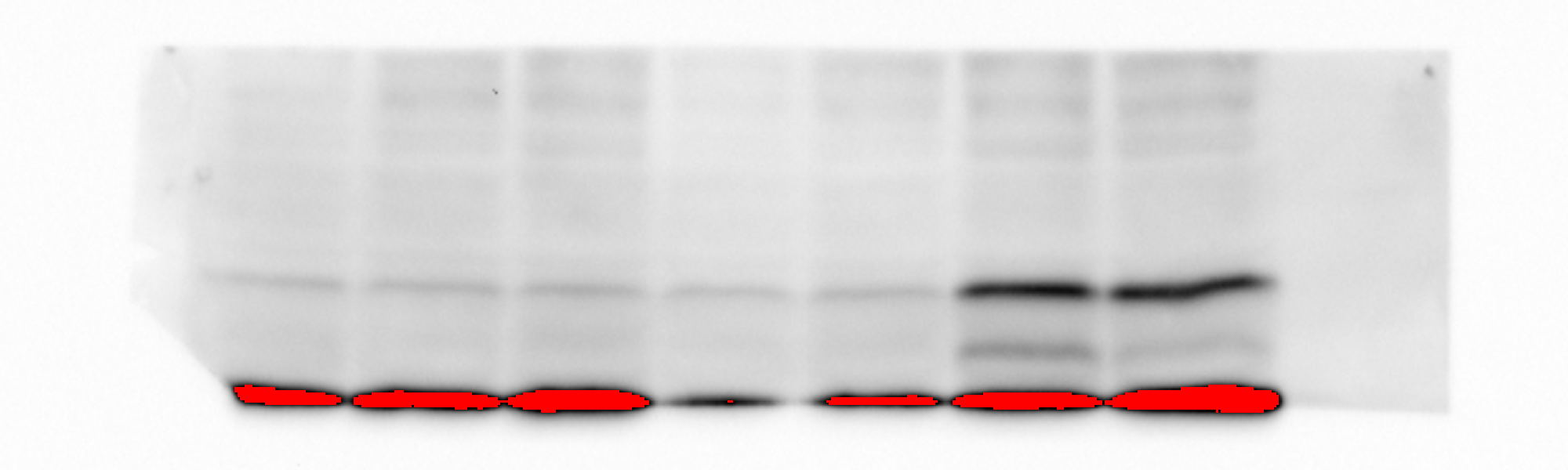

Supplement: Figure 6—source data 3. [file elife-97896-fig6-data3.zip › Figure 6-Source Data 3-24. /Figure 6-Source Data 3. Full raw unedited blot (Pmk1-P, KCl-treated group) for Figure 6B.tif]

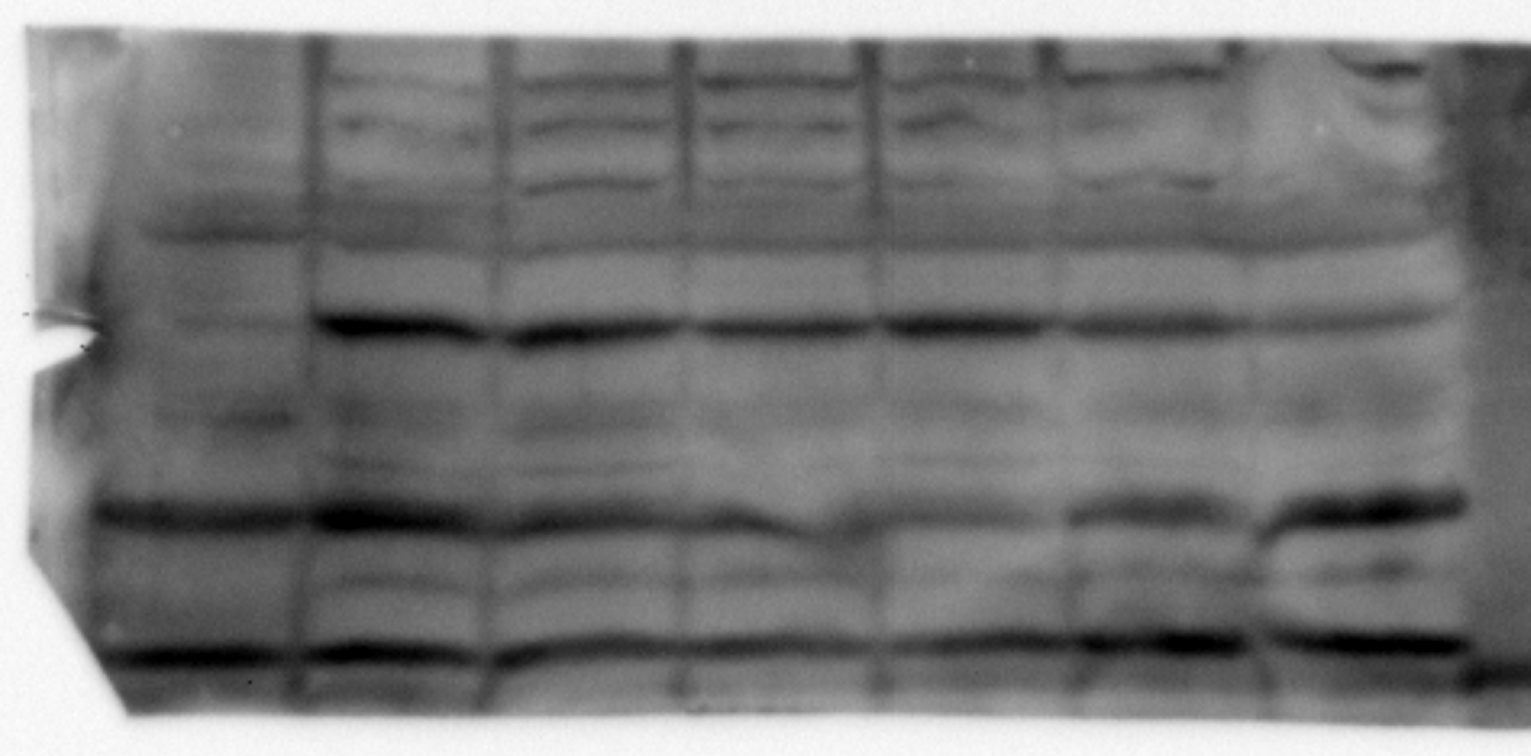

Supplement: Figure 6—source data 3. [file elife-97896-fig6-data3.zip › Figure 6-Source Data 3-24. /Figure 6-Source Data 5. Full raw unedited blot (Slp1, KCl-treated group) for Figure 6B.tif]

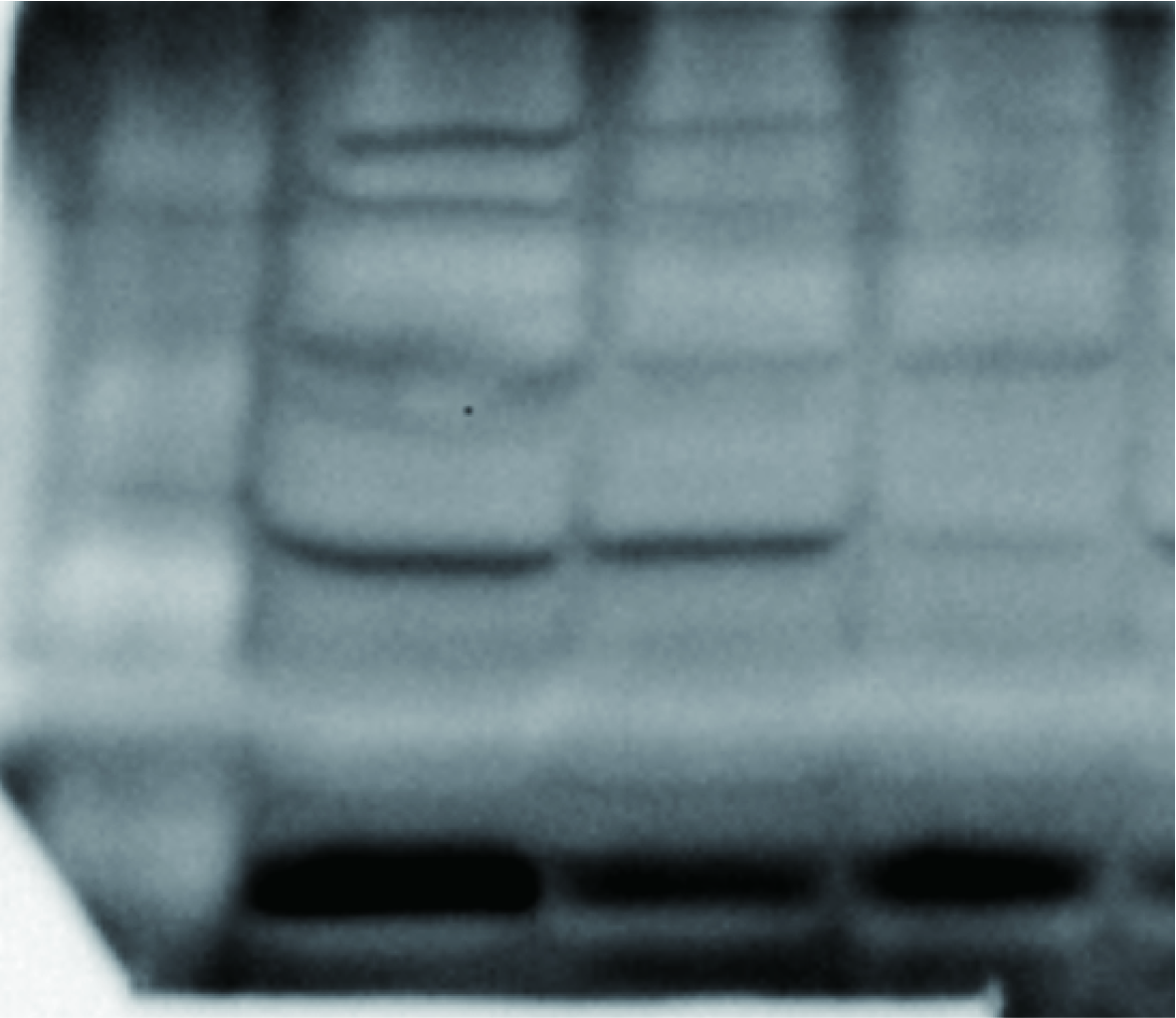

Supplement: Figure 6—source data 3. [file elife-97896-fig6-data3.zip › Figure 6-Source Data 3-24. /Figure 6-Source Data 21. Full raw unedited blot (Slp1 input, left) for Figure 6C.tif]

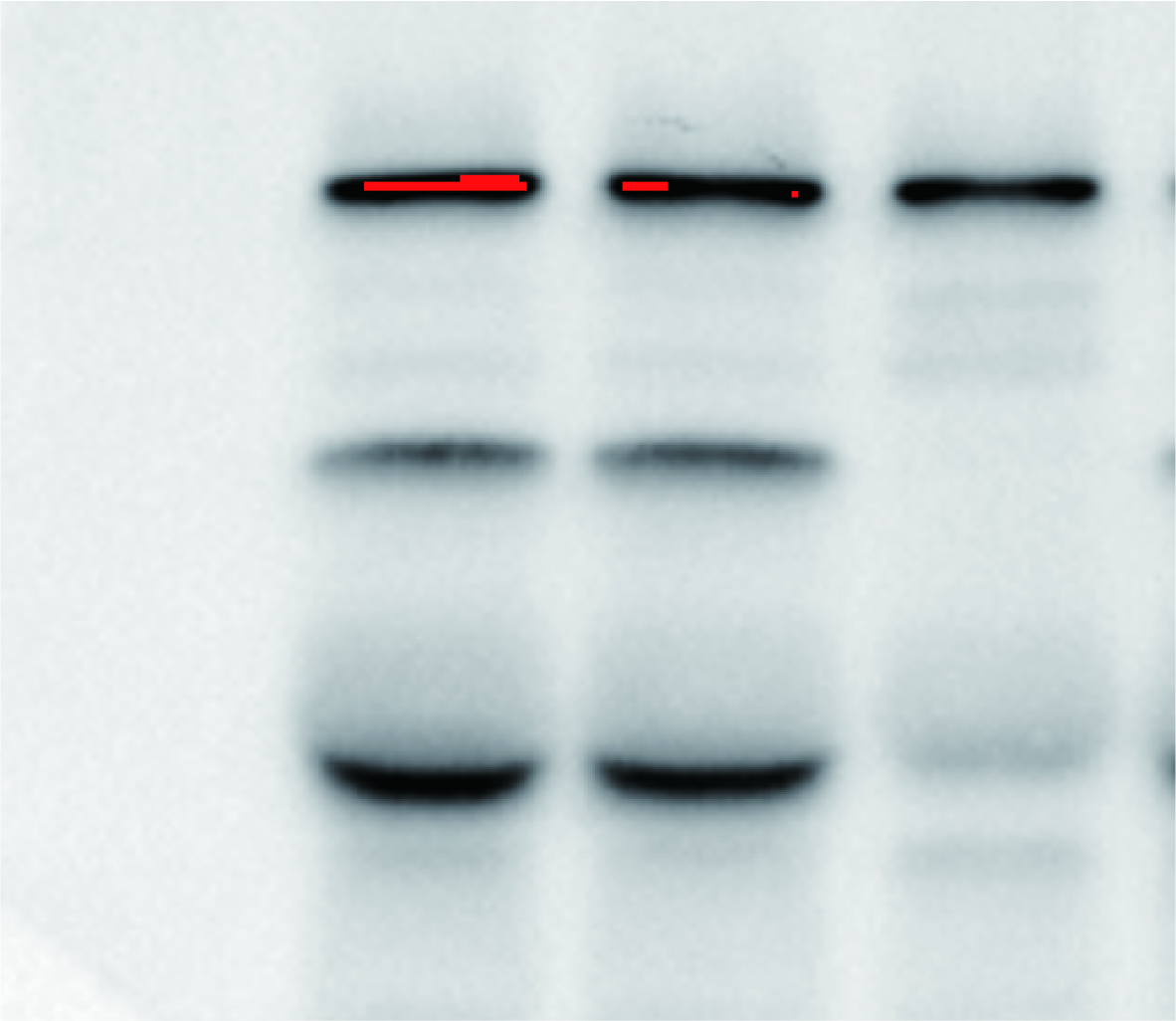

Supplement: Figure 6—source data 3. [file elife-97896-fig6-data3.zip › Figure 6-Source Data 3-24. /Figure 6-Source Data 14. Full raw unedited blot (co-IPed Mad2-GFP & Mad3-GFP, right) for Figure 6C.tif]

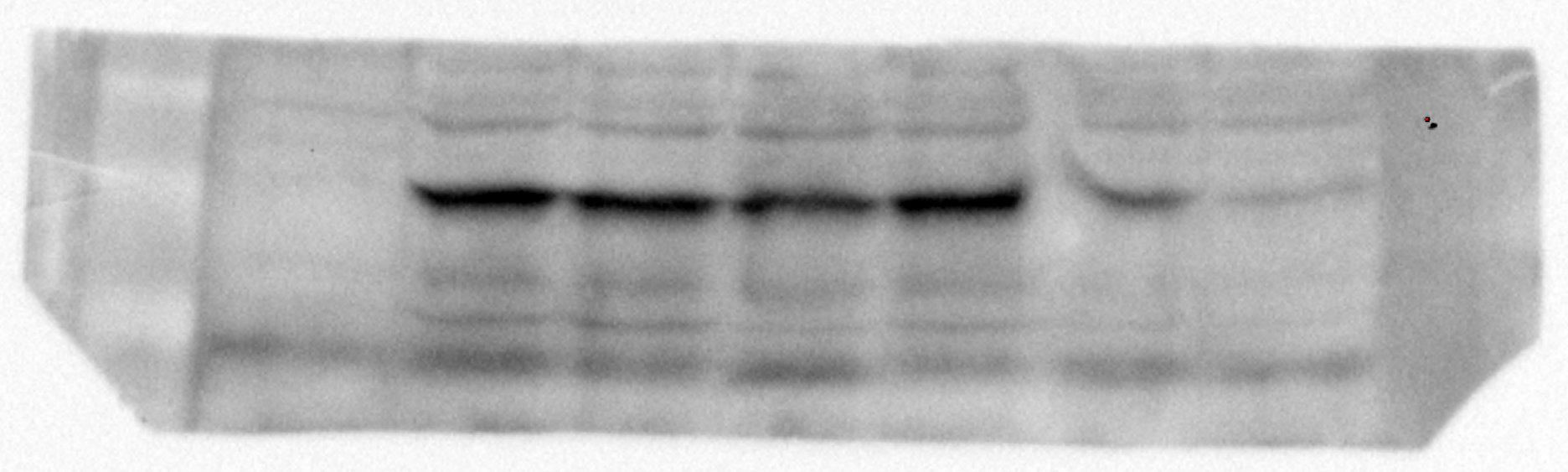

Supplement: Figure 6—source data 3. [file elife-97896-fig6-data3.zip › Figure 6-Source Data 3-24. /Figure 6-Source Data 9. Full raw unedited blot (Slp1, Caspofungin-treated group) for Figure 6B.tif]

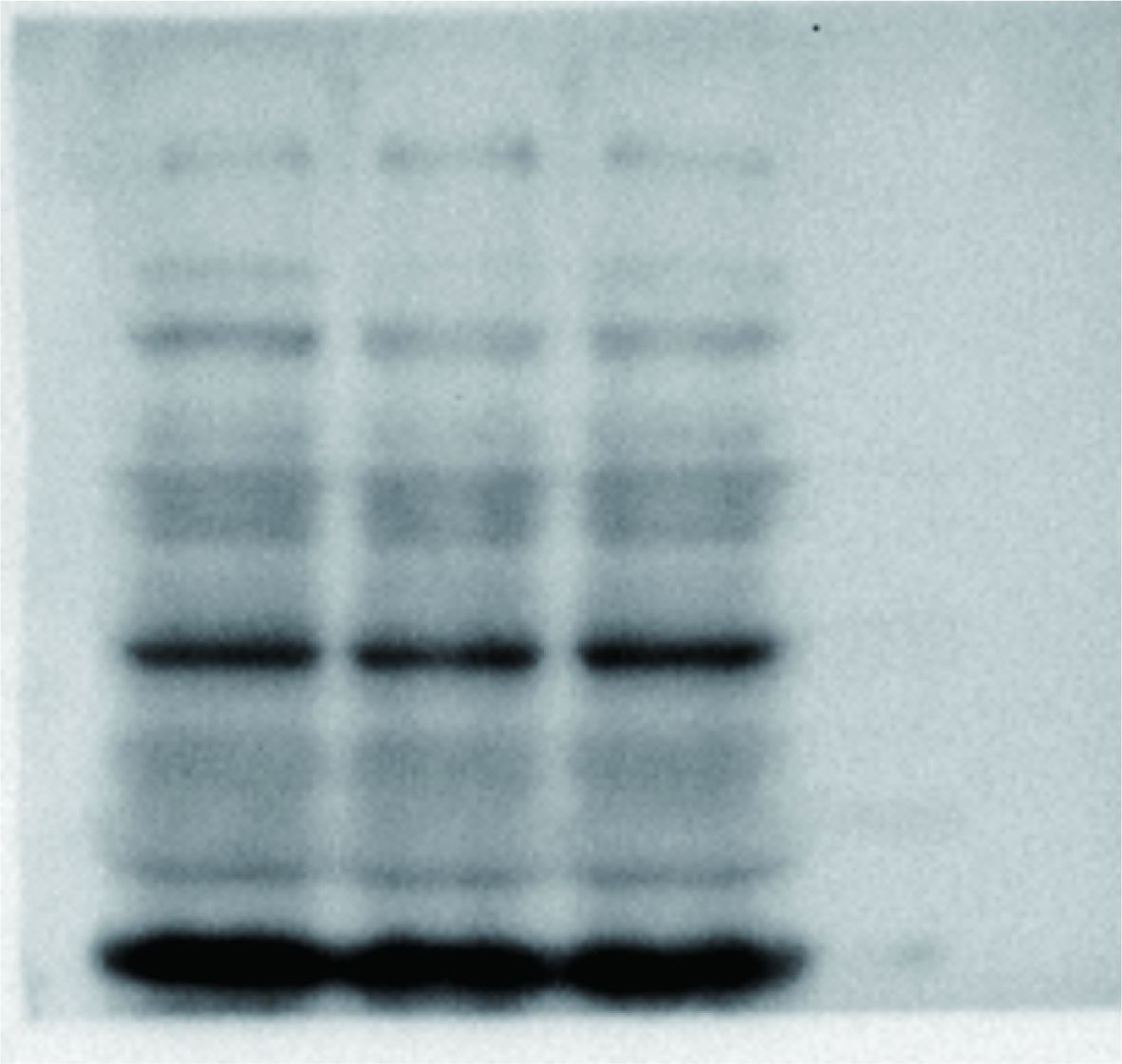

Supplement: Figure 6—source data 3. [file elife-97896-fig6-data3.zip › Figure 6-Source Data 3-24. /Figure 6-Source Data 22. Full raw unedited blot (Slp1 input, right) for Figure 6C.tif]

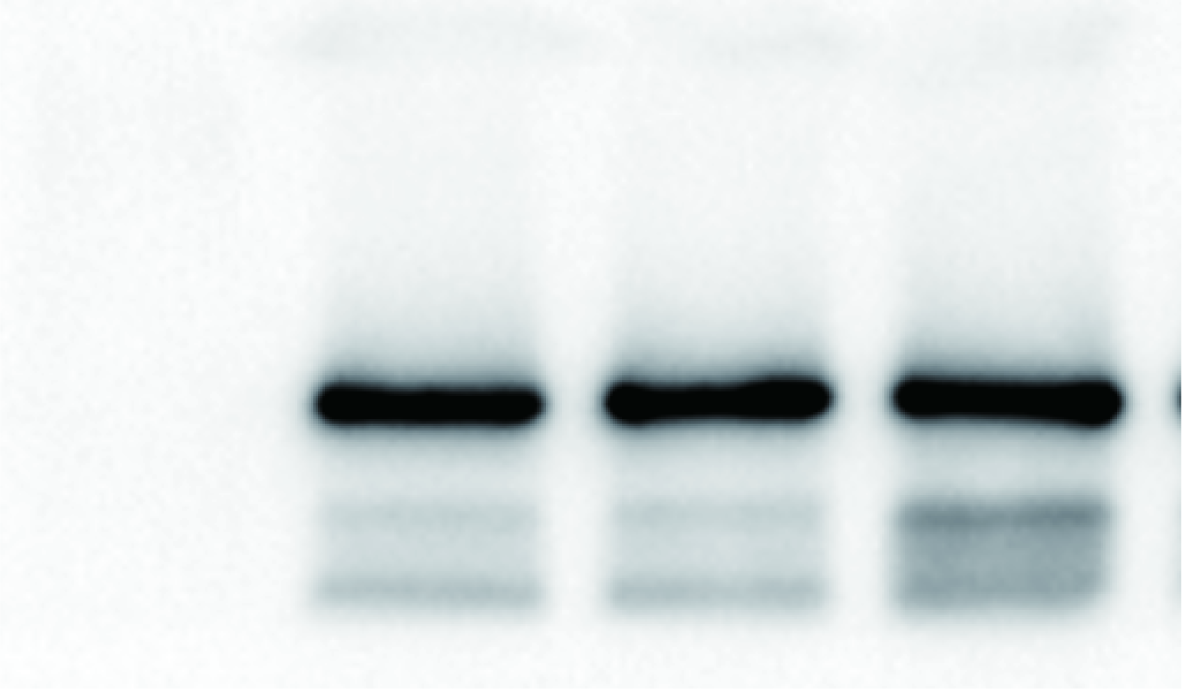

Supplement: Figure 6—source data 3. [file elife-97896-fig6-data3.zip › Figure 6-Source Data 3-24. /Figure 6-Source Data 12. Full raw unedited blot (IPed Lid1-TAP, right) for Figure 6C.tif]

Figure 6-figure supplement 1

|                                                    |   |   |   |   |
|----------------------------------------------------|---|---|---|---|
| KCl                                                | - | + | - | - |
| <i>P<sub>nmt1</sub></i> - <i>mad2</i> <sup>+</sup> | - | - | + | + |
| Thiamine                                           | - | - | + | - |

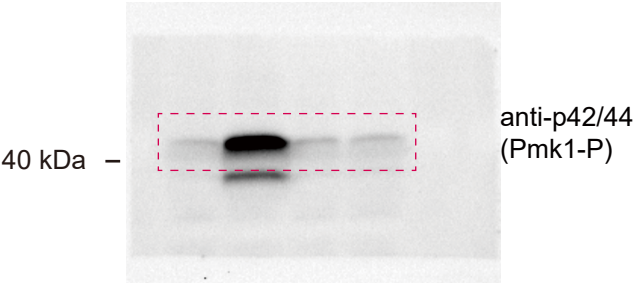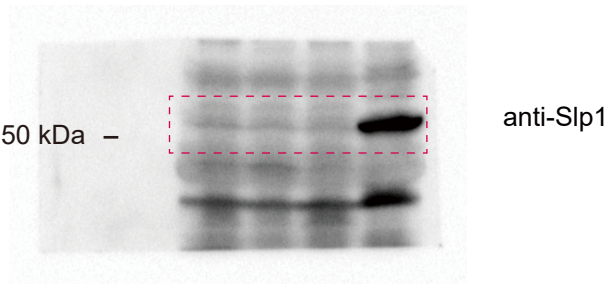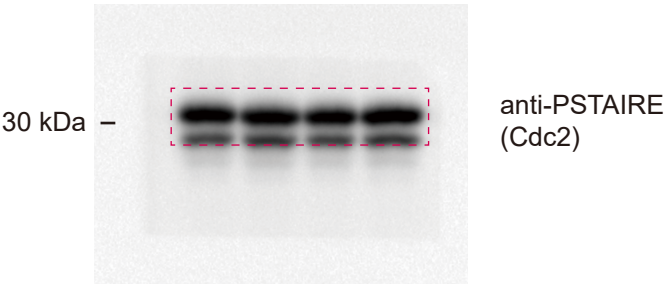

Supplement: Figure 6—figure supplement 1—source data 1. [file elife-97896-fig6-figsupp1-data1.pdf]

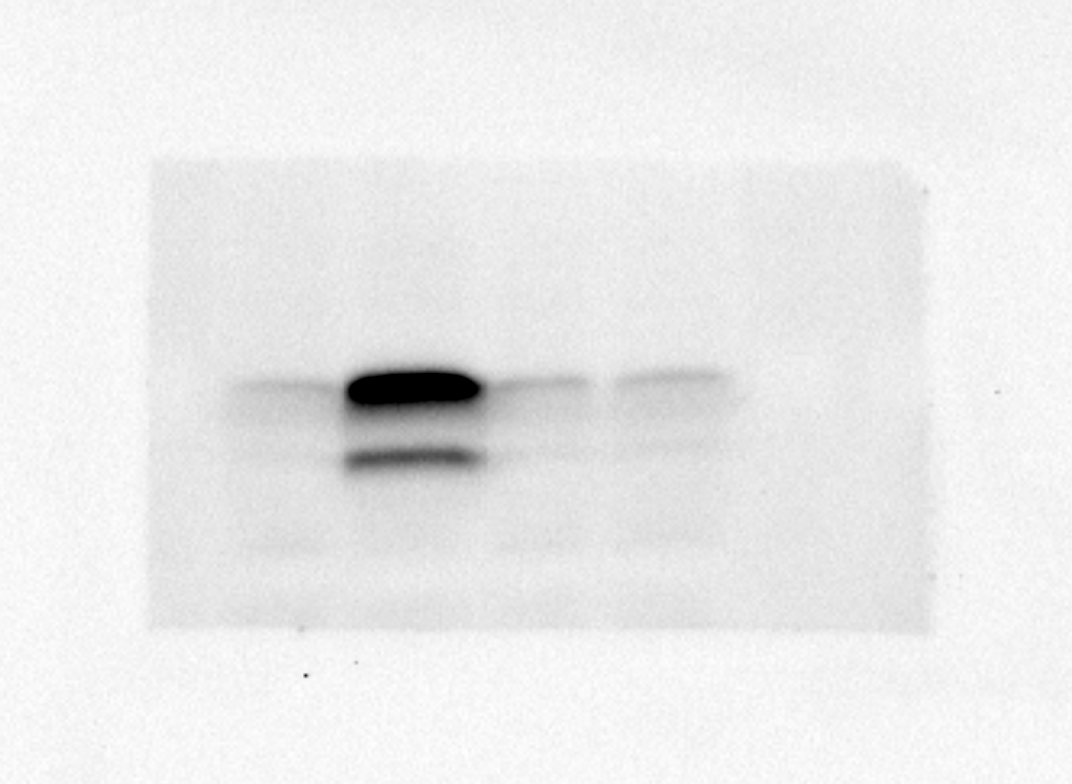

Supplement: Figure 6—figure supplement 1—source data 2. [file elife-97896-fig6-figsupp1-data2.zip › Figure 6-figure supplement 1-Source Data 2. Full raw unedited blot (phosphorylated Pmk1) for Figure 6-figure supplement 1.tif]

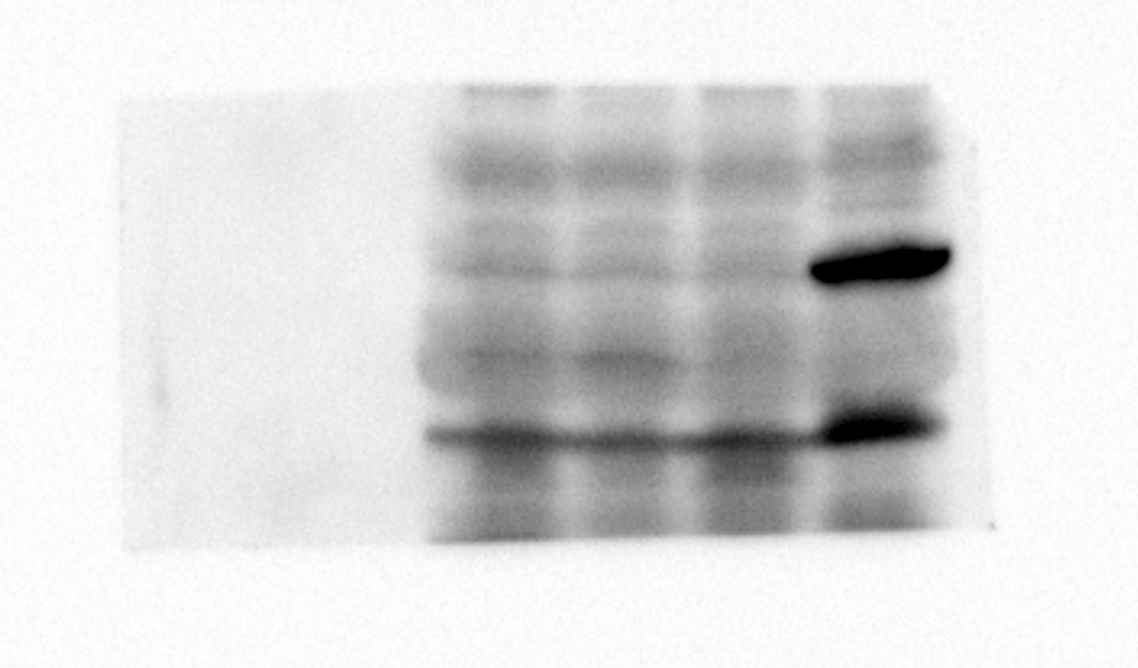

Supplement: Figure 6—figure supplement 1—source data 3. [file elife-97896-fig6-figsupp1-data3.zip › Figure 6-figure supplement 1-Source Data 3. Full raw unedited blot (Slp1) for Figure 6-figure supplement 1.tif]

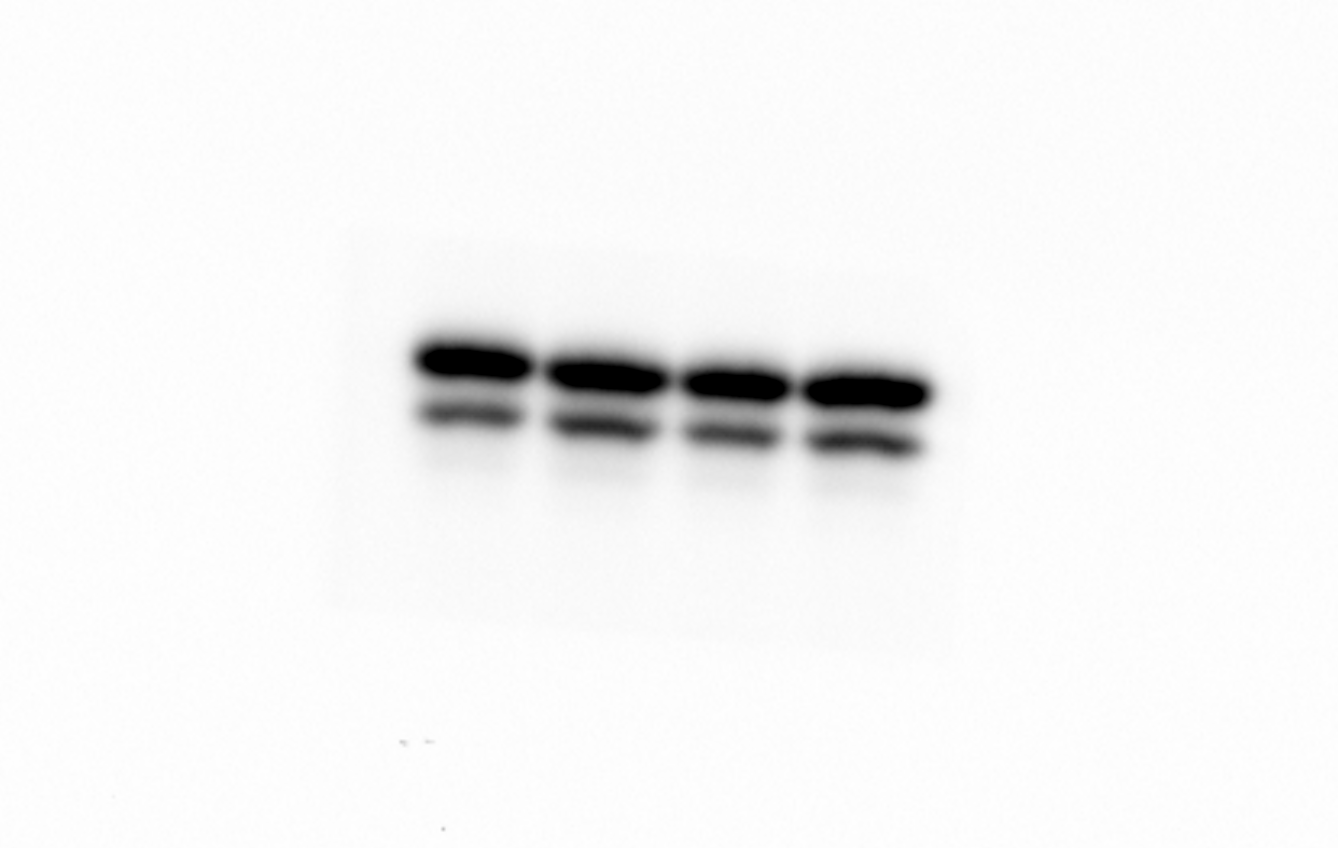

Supplement: Figure 6—figure supplement 1—source data 4. [file elife-97896-fig6-figsupp1-data4.zip › Figure 6-figure supplement 1-Source Data 4. Full raw unedited blot (Cdc2) for Figure 6-figure supplement 1.tif]

Figure 6-figure supplement 3.

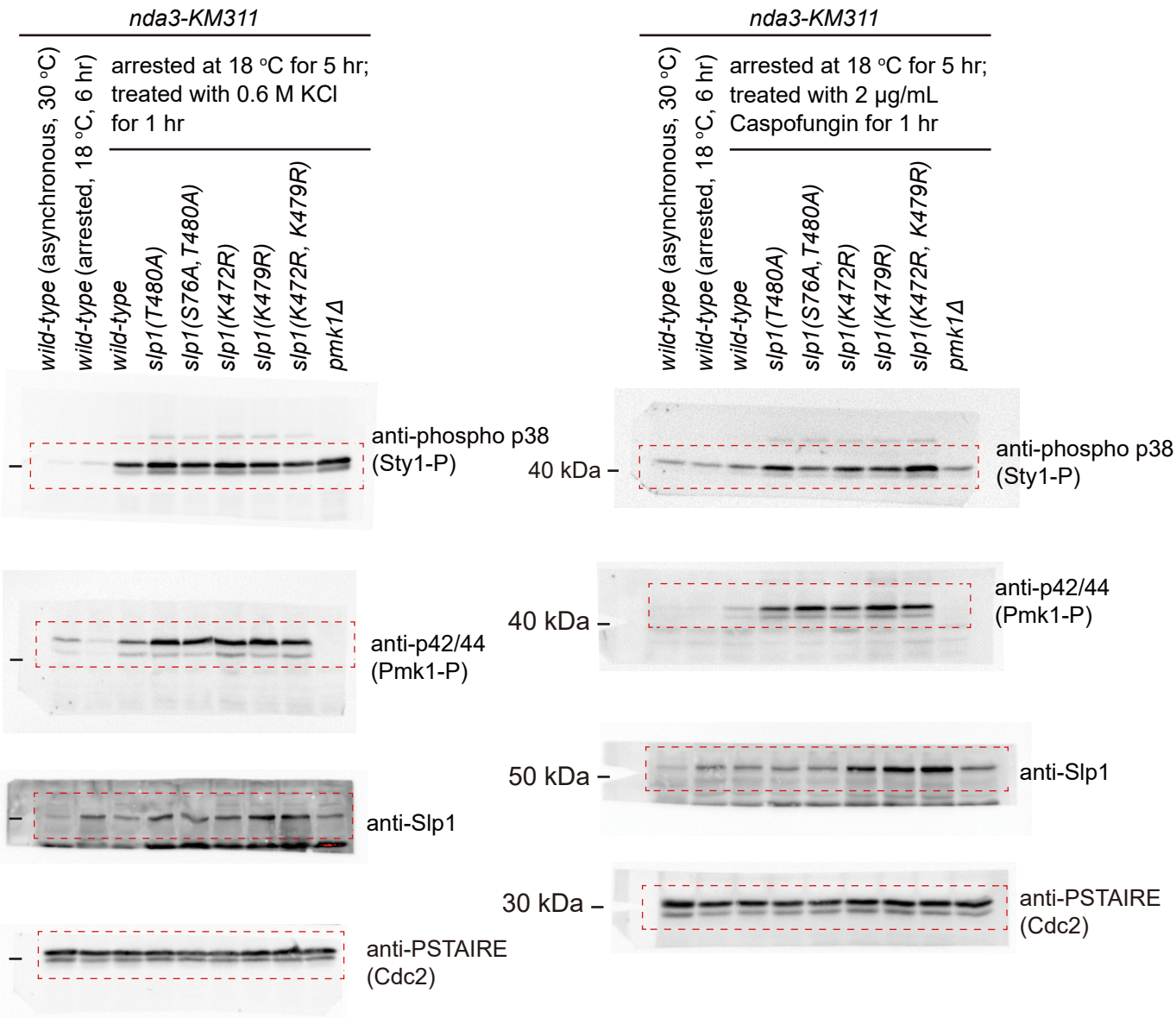

Supplement: Figure 6—figure supplement 3—source data 1. [file elife-97896-fig6-figsupp3-data1.pdf]

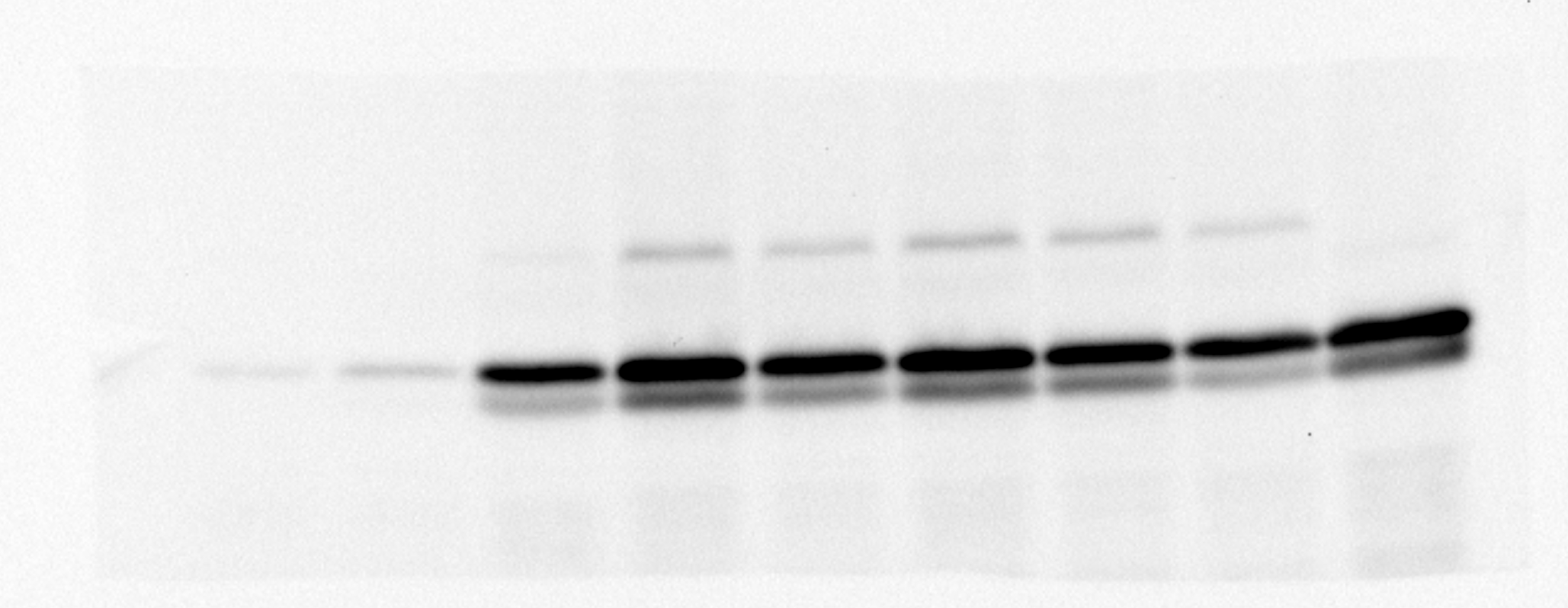

Supplement: Figure 6—figure supplement 3—source data 3. [file elife-97896-fig6-figsupp3-data3.zip › Figure 6-figure supplement 3-Source Data 3-10. /Figure 6-figure supplement 3-Source Data 3. Full raw unedited blot (Sty1-P, KCl) for Fig 6-figure suppl 3.tif]

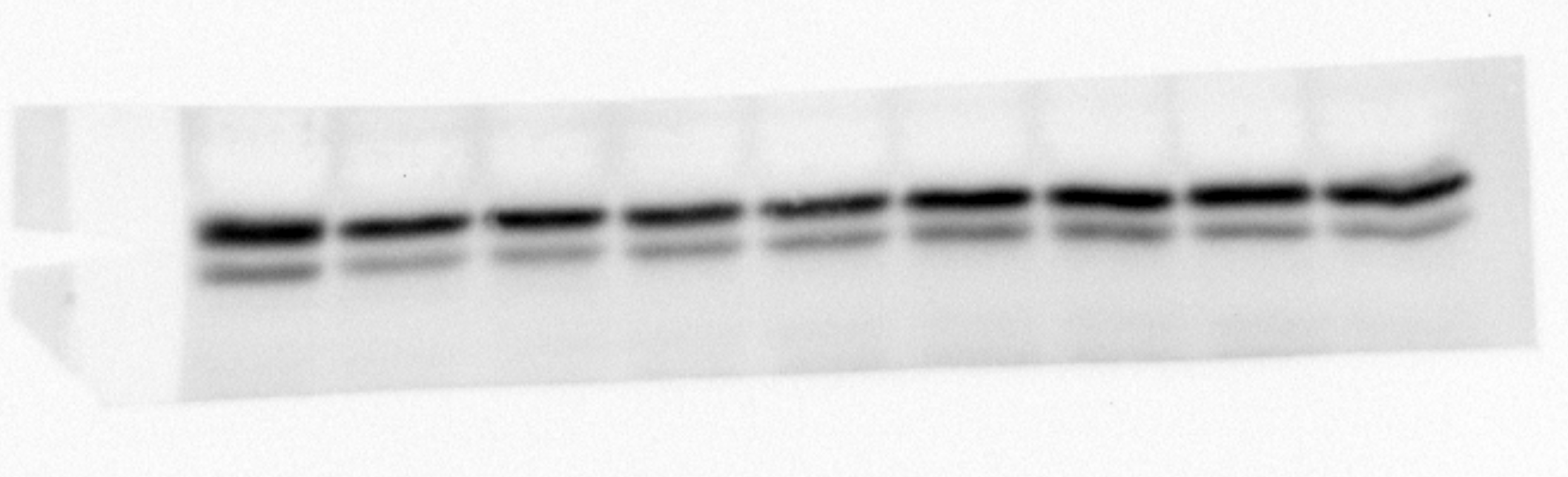

Supplement: Figure 6—figure supplement 3—source data 3. [file elife-97896-fig6-figsupp3-data3.zip › Figure 6-figure supplement 3-Source Data 3-10. /Figure 6-figure supplement 3-Source Data 10. Full raw unedited blot (Cdc2, Casp) for Fig 6-figure suppl 3.tif]

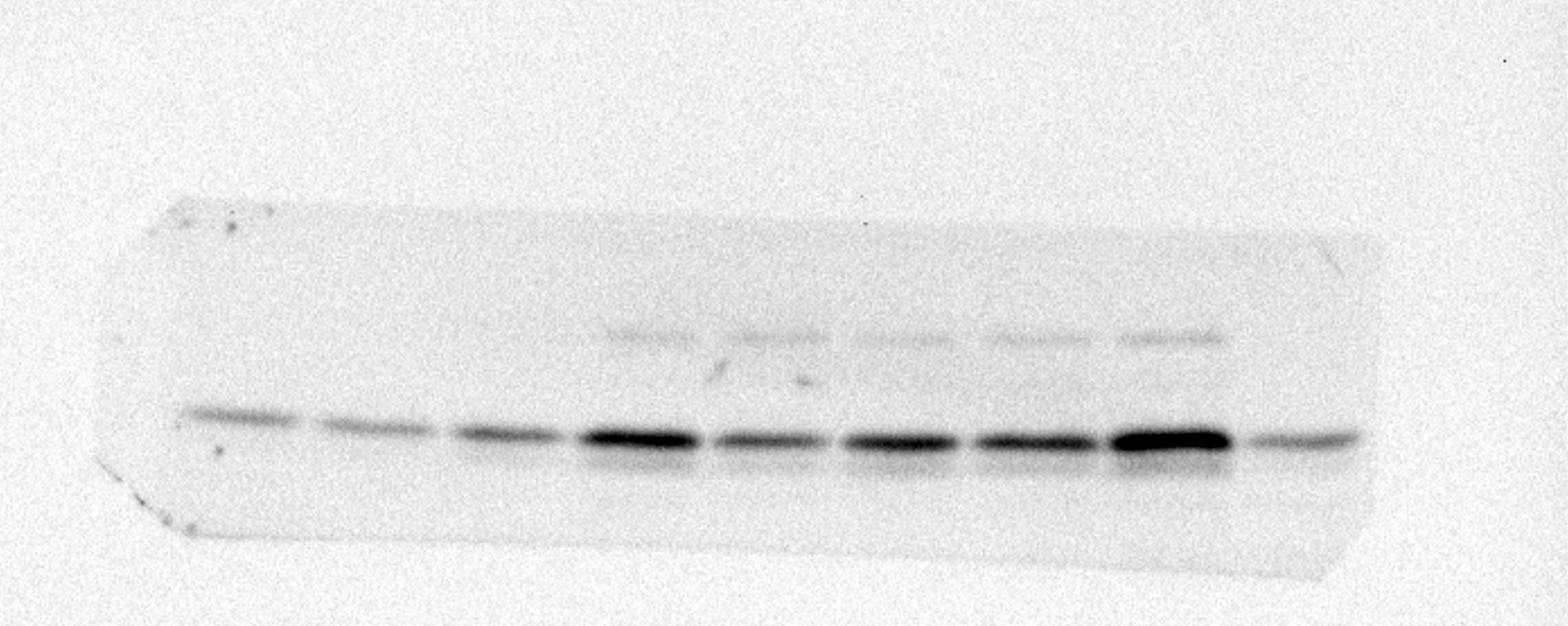

Supplement: Figure 6—figure supplement 3—source data 3. [file elife-97896-fig6-figsupp3-data3.zip › Figure 6-figure supplement 3-Source Data 3-10. /Figure 6-figure supplement 3-Source Data 7. Full raw unedited blot (Sty1-P, Casp) for Fig 6-figure suppl 3.tif]

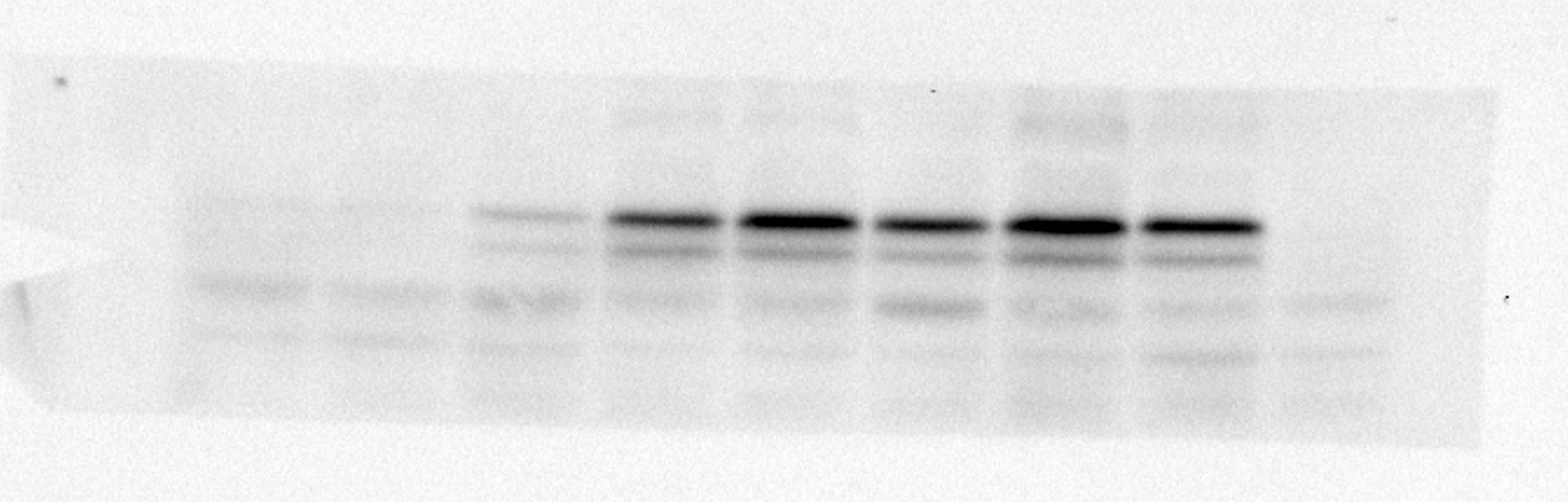

Supplement: Figure 6—figure supplement 3—source data 3. [file elife-97896-fig6-figsupp3-data3.zip › Figure 6-figure supplement 3-Source Data 3-10. /Figure 6-figure supplement 3-Source Data 8. Full raw unedited blot (Pmk1-P, Casp) for Fig 6-figure suppl 3.tif]

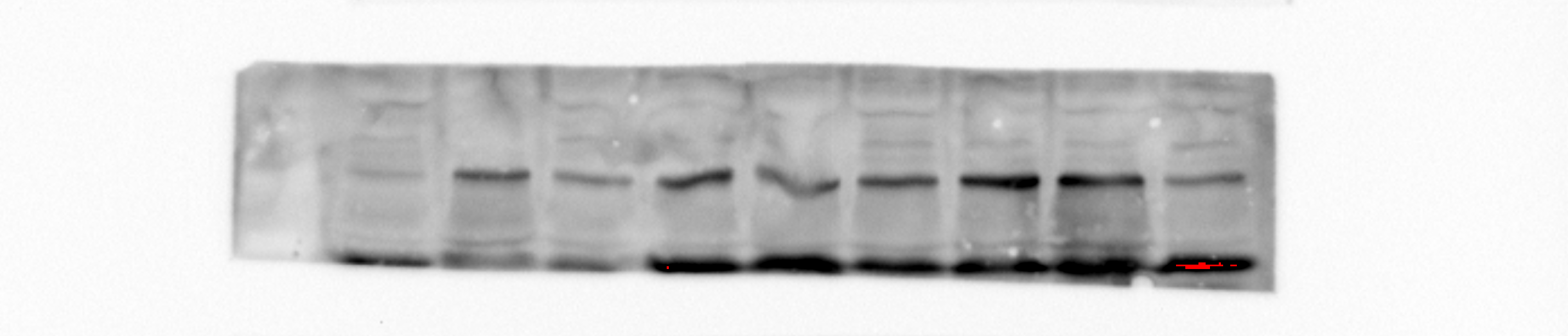

Supplement: Figure 6—figure supplement 3—source data 3. [file elife-97896-fig6-figsupp3-data3.zip › Figure 6-figure supplement 3-Source Data 3-10. /Figure 6-figure supplement 3-Source Data 5. Full raw unedited blot (Slp1, KCl) for Fig 6-figure suppl 3.tif]

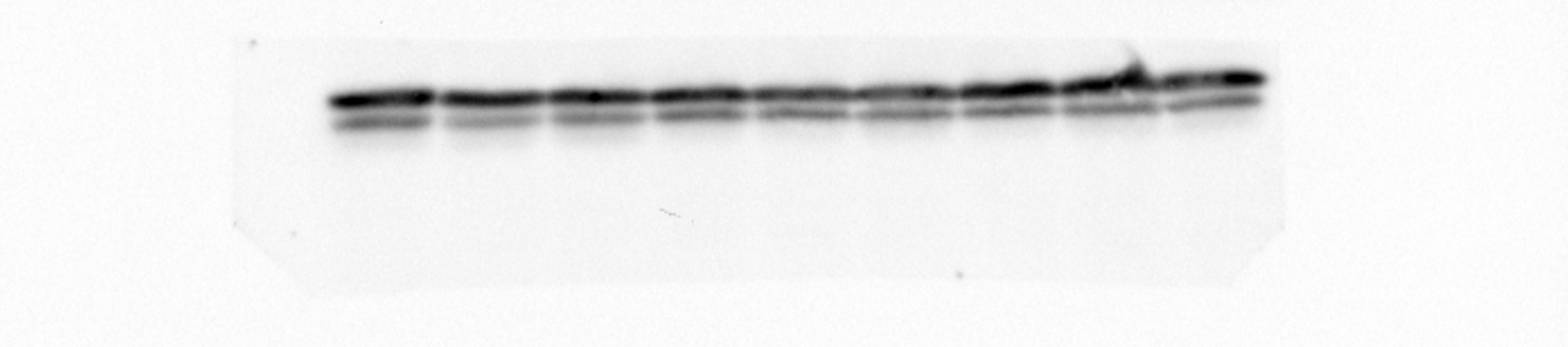

Supplement: Figure 6—figure supplement 3—source data 3. [file elife-97896-fig6-figsupp3-data3.zip › Figure 6-figure supplement 3-Source Data 3-10. /Figure 6-figure supplement 3-Source Data 6. Full raw unedited blot (Cdc2, KCl) for Fig 6-figure suppl 3.tif]

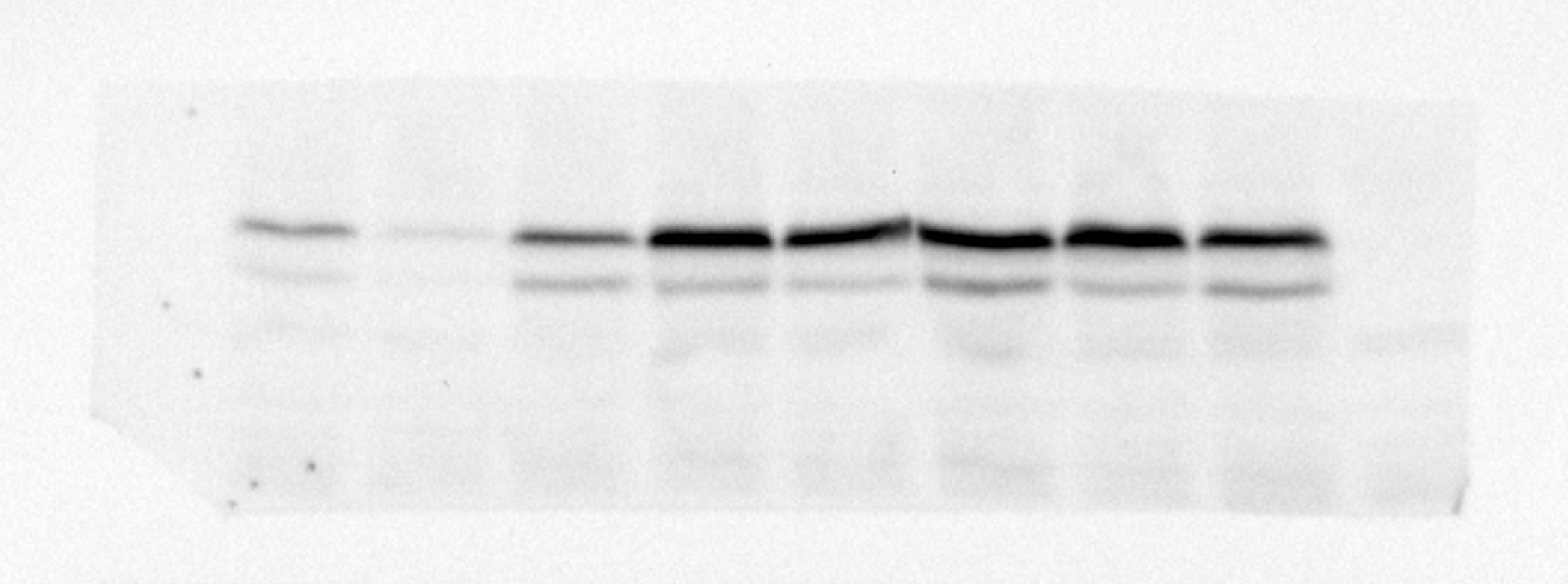

Supplement: Figure 6—figure supplement 3—source data 3. [file elife-97896-fig6-figsupp3-data3.zip › Figure 6-figure supplement 3-Source Data 3-10. /Figure 6-figure supplement 3-Source Data 4. Full raw unedited blot (Pmk1-P, KCl) for Fig 6-figure suppl 3.tif]

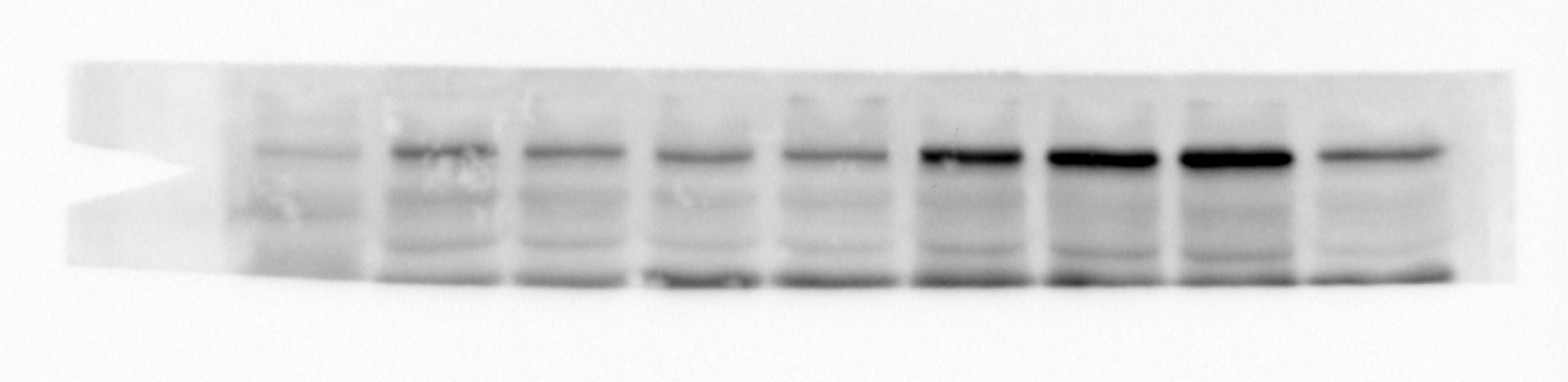

Supplement: Figure 6—figure supplement 3—source data 3. [file elife-97896-fig6-figsupp3-data3.zip › Figure 6-figure supplement 3-Source Data 3-10. /Figure 6-figure supplement 3-Source Data 9. Full raw unedited blot (Slp1, Casp) for Fig 6-figure suppl 3.tif]
