# Supplementary material for: Negative regulation of APC/C activation by MAPK-mediated attenuation of Cdc20Slp1 under stress
Source: eLife. 2024 Oct 16;13:RP97896. doi: 10.7554/eLife.97896 (PMC11483130; doi:10.7554/eLife.97896)
Supplement: Figure 6—source data 3. [file elife-97896-fig6-data3.zip › Figure 6 Source data titles.docx]

Figure 6-Source Data 3. Full raw unedited blot (phosphorylated Pmk1, KCl-treated group) for Figure 6B.

Figure 6-Source Data 4. Full raw unedited blot (phosphorylated Sty1, KCl-treated group) for Figure 6B.

Figure 6-Source Data 5. Full raw unedited blot (Slp1, KCl-treated group) for Figure 6B.

Figure 6-Source Data 6. Full raw unedited blot (Cdc2, KCl-treated group) for Figure 6B.

Figure 6-Source Data 7. Full raw unedited blot (phosphorylated Pmk1, Caspofungin-treated group) for Figure 6B.

Figure 6-Source Data 8. Full raw unedited blot (phosphorylated Sty1, Caspofungin-treated group) for Figure 6B.

Figure 6-Source Data 9. Full raw unedited blot (Slp1, Caspofungin-treated group) for Figure 6B.

Figure 6-Source Data 10. Full raw unedited blot (Cdc2, Caspofungin-treated group) for Figure 6B.

Figure 6-Source Data 11. Full raw unedited blot (IPed Lid1-TAP, left) for Figure 6C.

Figure 6-Source Data 12. Full raw unedited blot (IPed Lid1-TAP, right) for Figure 6C.

Figure 6-Source Data 13. Full raw unedited blot (co-IPed Mad2-GFP/Mad3-GFP, left) for Figure 6C.

Figure 6-Source Data 14. Full raw unedited blot (co-IPed Mad2-GFP/Mad3-GFP, right) for Figure 6C.

Figure 6-Source Data 15. Full raw unedited blot (co-IPed Slp1, left) for Figure 6C.

Figure 6-Source Data 16. Full raw unedited blot (co-IPed Slp1, right) for Figure 6C.

Figure 6-Source Data 17. Full raw unedited blot (Lid1-TAP input, left) for Figure 6C.

Figure 6-Source Data 18. Full raw unedited blot (Lid1-TAP input, right) for Figure 6C.

Figure 6-Source Data 19. Full raw unedited blot (Mad2-GFP/Mad3-GFP input, left) for Figure 6C.

Figure 6-Source Data 20. Full raw unedited blot (Mad2-GFP/Mad3-GFP input, right) for Figure 6C.

Figure 6-Source Data 21. Full raw unedited blot (Slp1 input, left) for Figure 6C.

Figure 6-Source Data 22. Full raw unedited blot (Slp1 input, right) for Figure 6C.

Figure 6-Source Data 23. Full raw unedited blot (Cdc2 input, left) for Figure 6C.

Figure 6-Source Data 24. Full raw unedited blot (Cdc2 input, right) for Figure 6C.
