# Supplementary material for: Negative regulation of APC/C activation by MAPK-mediated attenuation of Cdc20Slp1 under stress
Source: eLife. 2024 Oct 16;13:RP97896. doi: 10.7554/eLife.97896 (PMC11483130; doi:10.7554/eLife.97896)
Supplement: Figure 6—figure supplement 3—source data 3. [file elife-97896-fig6-figsupp3-data3.zip › Figure 6 figure supplement 3 source data titles.docx]

Figure 6-figure supplement 3-Source Data 2. Raw data of Slp1 level measurement for Figure 6-figure supplement 3.

Figure 6-figure supplement 3-Source Data 3. Full raw unedited blot (phosphorylated Sty1, KCl-treated group) for Figure 6-figure supplement 3.

Figure 6-figure supplement 3-Source Data 4. Full raw unedited blot (phosphorylated Pmk1, KCl-treated group) for Figure 6-figure supplement.

Figure 6-figure supplement 3-Source Data 5. Full raw unedited blot (Slp1, 2027 KCl-treated group) for Figure 6-figure supplement 3.

Figure 6-figure supplement 3-Source Data 6. Full raw unedited blot (Cdc2, 2029 KCl-treated group) for Figure 6-figure supplement 3.

Figure 6-figure supplement 3-Source Data 7. Full raw unedited blot (phosphorylated Sty1, Caspofungin-treated group) for Figure 6-figure supplement 3.

Figure 6-figure supplement 3-Source Data 8. Full raw unedited blot(phosphorylated Pmk1, Caspofungin-treated group) for Figure 6-figure supplement 3.

Figure 6-figure supplement 3-Source Data 9. Full raw unedited blot (Slp1, Caspofungin-treated group) for Figure 6-figure supplement 3.

Figure 6-figure supplement 3-Source Data 10. Full raw unedited blot (Cdc2, Caspofungin-treated group) for Figure 6-figure supplement 3.
